# The evolution of new lipoprotein subunits of the bacterial outer membrane BAM complex

**DOI:** 10.1111/j.1365-2958.2012.08059.x

**Published:** 2012-04-23

**Authors:** Khatira Anwari, Chaille T Webb, Sebastian Poggio, Andrew J Perry, Matthew Belousoff, Nermin Celik, Georg Ramm, Andrew Lovering, R Elizabeth Sockett, John Smit, Christine Jacobs-Wagner, Trevor Lithgow

**Affiliations:** 1 Department of Biochemistry & Molecular Biology, Monash UniversityMelbourne 3800, Australia; 2 Department of Molecular, Cellular and Developmental Biology and Microbial Pathogenesis Section, Howard Hughes Medical Institute, Yale UniversityNew Haven, CT 06520, USA; 3 School of Biosciences, University of BirminghamBirmingham, UK; 4 Centre for Genetics and Genomics, School of Biology, University of Nottingham, Medical School, QMCNottingham, UK; 5 Department of Microbiology & Immunology, University of British ColumbiaVancouver, B.C. V6T1Z3, Canada

## Abstract

The β-barrel assembly machine (BAM) complex is an essential feature of all bacteria with an outer membrane. The core subunit of the BAM complex is BamA and, in *Escherichia coli*, four lipoprotein subunits: BamB, BamC, BamD and BamE, also function in the BAM complex. Hidden Markov model analysis was used to comprehensively assess the distribution of subunits of the BAM lipoproteins across all subclasses of proteobacteria. A patchwork distribution was detected which is readily reconciled with the evolution of the α-, β-, γ-, δ- and ε-proteobacteria. Our findings lead to a proposal that the ancestral BAM complex was composed of two subunits: BamA and BamD, and that BamB, BamC and BamE evolved later in a distinct sequence of events. Furthermore, in some lineages novel lipoproteins have evolved instead of the lipoproteins found in *E. coli*. As an example of this concept, we show that no known species of α-proteobacteria has a homologue of BamC. However, purification of the BAM complex from the model α-proteobacterium *Caulobacter crescentus* identified a novel subunit we refer to as BamF, which has a conserved sequence motif related to sequences found in BamC. BamF and BamD can be eluted from the BAM complex under similar conditions, mirroring the BamC:D module seen in the BAM complex of γ-proteobacteria such as *E. coli*.

## Introduction

Current models for the evolution of the kingdom *Bacteria* suggest that some of the earliest bacteria had outer membranes as protection against small molecule toxins, radiation and desiccation (Cavalier-Smith, 2006). The assembly of proteins into the outer membrane assembly is essential for cell viability, with protein precursors first transiting across the inner membrane and periplasmic space. The Sec machinery mediates protein translocation across the inner membrane, and periplasmic transport is facilitated by molecular chaperones such as DegP, Skp and SurA. A protein complex in the outer membrane, known as the β-barrel assembly machine (BAM) complex, captures and inserts the protein substrates from these chaperones for assembly into the lipid phase of the outer membrane (Ruiz *et al*., 2006; Bos *et al*.*,* 2007; Knowles *et al*., 2009; Hagan *et al*., 2010; Ricci and Silhavy, 2011).

The BAM complex is a multimeric machine. The core subunit of the BAM complex, BamA, is a member of the Omp85 family of proteins (Gentle *et al*., 2004; Bos *et al*.*,* 2007; Gatsos *et al*., 2008; Knowles *et al*., 2009). Homologues of BamA are found in all bacteria with outer membranes (Gentle *et al*., 2004; Voulhoux and Tommassen, 2004), leading to the suggestion that BamA is an extremely ancient feature of the kingdom *Bacteria* (Cavalier-Smith, 2006). In *Escherichia coli*, four lipoprotein partners of BamA have been identified: BamB, BamC, BamD and BamE (Bos *et al*., 2007; Knowles *et al*., 2009; Hagan *et al*., 2010; Ricci and Silhavy, 2011). Recent successes in solving crystal structures of the lipoprotein partners in the BAM complex of *E. coli* have inspired models for the architecture and function of the BAM complex. BamB has a beta-propeller fold (Gatsos *et al*., 2008; Kim and Paetzel, 2010; Noinaj *et al*., 2011), a common protein–protein scaffolding topology (Chen *et al*., 2011). BamC has two domains each with ‘helix-grip’ architecture, but interacts with BamD via an N-terminal linker region which is intrinsically disordered in NMR experiments (Warner *et al*., 2011) and ill-defined in crystal structures (Kim *et al*., 2011). The BamD subunit has multiple tetratricopeptide repeats (TPR), a 34-residue segment which packs into a helix–turn–helix with defined symmetry; the TPR segments stack into a super-helical arrangement and are often found in the substrate-binding subunits of protein transport systems (D’Andrea and Regan, 2003; Gatsos *et al*., 2008). BamE is a small lipoprotein of ∼ 10 kDa with the potential to dimerize and also to associate with the essential protein BamD (Albrecht and Zeth, 2011; Kim *et al*., 2011; Rigel *et al*., 2012).

As *E. coli* is a γ-proteobacterium, comparative analyses of the BAM complex in this model organism and more distant proteobacterial species would offer insight into the evolution of the outer membrane assembly machinery. The Proteobacteria are divided into subclasses called the α-, β-, γ-, δ- and ε-proteobacteria, with current models of evolution suggesting that the γ-proteobacterial lineages was one of the last to arise (Woese, 1987; Olsen *et al*., 1994; Klenk *et al*., 1999; Gupta, 2000). Understanding the evolution of the BAM complex in turn may offer new ways to approach the question of mechanism in how outer membrane proteins are assembled by this remarkable molecular machine.

Here we used hidden Markov model (HMM) analysis to comprehensively assess the distribution of the BAM lipoproteins, and find a patchwork distribution that can be readily reconciled with the evolution of the α-, β-, γ-, δ- and ε-proteobacteria. We propose that the ancestral BAM complex was composed of two subunits: BamA and BamD, and that BamB, BamC and BamE evolved later in a distinct sequence of events. Of the 149 species of α-proteobacteria that have yielded complete genome sequences, none encodes a homologue of BamC. However, purification of the BAM complex from the model α-proteobacterium *Caulobacter crescentus* identified a novel subunit, with sequence characteristics suggesting it to be an outer membrane lipoprotein. This BamF subunit is predicted to have an intrinsically disordered N-terminal domain with a conserved sequence motif, related to sequences found in BamC. We suggest that while BamB, BamC, BamD and BamE constitute the lipoprotein components of the BAM complex in β- and γ-proteobacteria, other bacterial lineages have independently evolved to have distinct lipoproteins docked into the BAM complex to ensure its function of assembling β-barrel proteins into the bacterial outer membrane.

## Results

### A patchwork distribution of the four BAM complex lipoproteins

We used HMM analysis to comprehensively assess the distribution of the components of the BAM complex. The results are summarized in Table 1.

**Table 1:** HMM detection of BAM complex subunits

Class (number of genomes)	BamA	BamB	BamC	BamD	BamE
α-Proteobacteria (149)	244	280	0	151	110
β-Proteobacteria (99)	180	192	121	118	116
γ-Proteobacteria (293)	550	461	187	340	422
δ-Proteobacteria (41)	80	32^a^	0	85	6^c^
ε-Proteobacteria (37)	39	5^b^	0	36	1^c^

a. As detailed in the text, only four of these proteins have *E*-values of < e-08 suggesting that they may be genuine BamB sequences, which might have been acquired by lateral gene transfer (Fig. S2).

b. All have *E*-values between e-08 and e-05.

c. The few δ-proteobacteria (*Bdellovibrio bacteriovorus* HD100, *Desulfobulbus propionicus* DSM 2032, *Geobacter bemidjiensis* Bem, *Geobacter* sp. FRC-32, *Pelobacter carbinolicus* DSM 2380, *Pelobacter propionicus* DSM 2379) and the only ε-proteobacteria (*Sulfurospirillum deleyianum* DSM 6946) species that have hits would score as OsmE-like lipoproteins (‘BamE’*E*-values between e-08 and e-05).

In order to capture even distantly related sequences, including those of ancestrally related, non-homologous proteins, the scan was carried out with a non-conservative cut-off *E*-value of 10−5 (see *Experimental procedures*). Grey shading denotes an absence of genuine BAM subunits, as defined in the text.

As previously reported (Gentle *et al*., 2004; Voulhoux and Tommassen, 2004), BamA is encoded in the genomes of all bacteria with an outer membrane. Using a ‘relaxed’ cut-off *E*-value of e-05, the HMM for detecting BamA sequences also picks up a distinct class of Omp85 protein called YtfM/TamA. For example, *E. coli* K-12 substr. MG1655 has a BamA with a perfect *E*-value score ‘0’ (NP_414719.1) and a second form with an *E*-value score 9.3 e-07 (NP_418641.1). With this in mind, we applied a relaxed *E*-value cut-off of e-05 to the other HMM searches to detect both homologues (defined as those with *E*-value scores < e-08) and more distantly related proteins of other protein families (with *E*-value scores between e-05 and e-08).

**Fig. 1:**
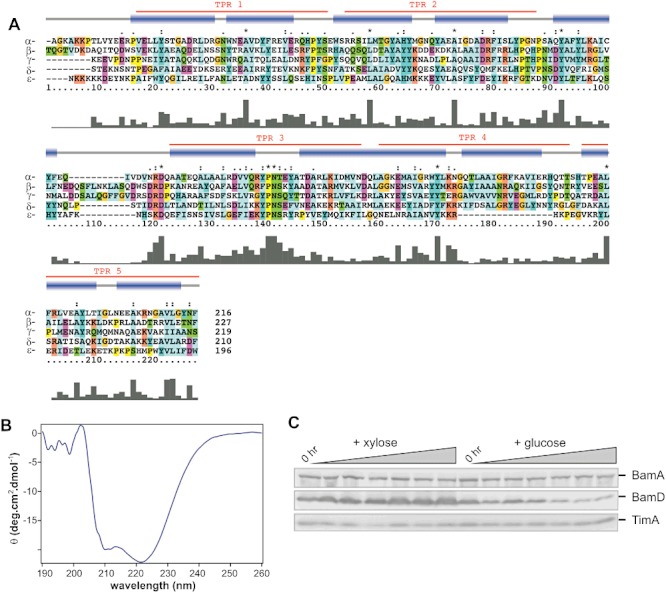
BamD is an essential component of the BAM complex in *C. crescentus*. A. clustalw sequence alignment combined with both secondary structure and TPR prediction of the BamD sequences from proteobacteria of the α- (*C. crescentus*), β- (*N. meningitidis*), γ- (*E. coli*), δ- (*B. bacteriovorus*) and ε- (*Helicobacter pylori*) subclasses is shown. Red denotes TPR prediction, blue boxes define areas that are predicted to be α-helical, and the histogram depicts sequence conservation across the alignment. Amino acid residues are coloured according to side-chain properties: e.g. light blue = hydrophobic, magenta = acidic. *N.B.* All lipoprotein sequences lack the N-terminal signal sequence, and a 60 residue C-terminal extension from the α-proteobacterial BamD is not shown. B. Recombinant BamD was purified and analysed by circular dichroism, the spectra clearly indicates a predominately α-helical structure (for more details refer to Table S1). C. A BamD-depletion strain of *C. crescentus* was cultured overnight in growth medium containing xylose and a ‘0 h’ sample removed from the culture. Equal volumes of cells were then resuspended in growth medium containing either 0.03% (w/v) xylose or 0.2% (w/v) glucose. Lanes labelled ‘0 hr’ correspond to the initial samples. At hourly time points up to 6 h, an equivalent volume of culture was prepared for analysis by SDS-PAGE and immunoblotting with antisera recognizing BamD and the control proteins BamA (Anwari *et al*., 2010) and TimA (Clements *et al*., 2009). No viable colonies could be cultured after growth on glucose-containing medium for 16 h.

Using these search parameters, the only other component of the BAM complex to be detected ubiquitously in proteobacteria is BamD (Table 1). In several species of proteobacteria, YbgF, a TPR protein that docks to the Tol–Pal system (Krachler *et al*., 2010), is also detected by our HMM suggesting that this protein has some limited similarity to BamD. In some species (e.g. *Pseudomonas aeruginosa*) there are two genes encoding YbgF-like proteins (NP_249665.1 and NP_254189.1) in addition to the genuine BamD (NP_253235.1). The three-dimensional structure of a BamC–BamD module from the BAM complex of *E. coli* shows that specific contacts are made through an N-terminal sequence of BamC across the TPR domains of BamD (Kim *et al*., 2011). In the α-proteobacterium *C. crescentus*, the putative BamD protein corresponding to the high-scoring sequence detected with the HMM (NP_420791.1) has been co-purified with the BAM complex (Anwari *et al*., 2010), and is predicted to contain TPR sequences that can be aligned reliably to known structural homologues from *E. coli* and *Rhodothermus marinus* (Sandoval *et al*., 2011; Albrecht and Zeth, 2011) (Fig. 1A). The *C. crescentus* BamD was expressed in *E. coli* and the purified BamD shows a circular dichroism profile characteristic of an alpha-helical TPR structure (Fig. 1B). This protein is essential for viability in the α-proteobacterium *C. crescentus*, as has been observed for BamD from a species of β-proteobacteria (Volokhina *et al*., 2009) and γ-proteobacteria (Malinverni *et al*., 2006): in a strain of *C. crescentus* with the gene encoding BamD under the control of a xylose-inducible promoter, growth in the absence of xylose led to a substantial depletion of BamD within 6 h (Fig. 1C), and after 16 h resulted in loss of cell viability (see *Experimental procedures*) and outer membrane blebbing as judged by scanning electron microscopy (SEM) (Fig. S1).

In the case of BamB and BamE also, there are related proteins that score *E*-values > e-08 (Table 1). The BamB HMM detects cytoplasmic dehydrogenases with low significance; e.g. the BamB sequence of *Azospirillum* sp. B510 (YP_003448892.1) has a perfect score of ‘0’, but scores that range from e-06 to e-08 are given for three proteins annotated as ‘alcohol dehydrogenase’ (YP_003450601.1 and YP_003451663.1) and ‘quinoprotein glucose dehydrogenase’ (YP_003453045.1). Similarly, with a relaxed cut-off score, the BamE HMM detects the osmotic-sensitive lipoprotein OsmE (at ∼e-05). In some species (e.g. *P. aeruginosa*) the OsmE form is represented by two genes (YP_001351160.1 and YP_001350807.1), in addition to the genuine BamE (YP_001350807.1).

We find no evidence that BamB, BamC or BamE are components of the BAM complex in δ- or ε-proteobacteria (Table 1). A few species of δ-proteobacteria: *Bdellovibrio bacteriovorus*, *Haliangium ochraceum, Myxococcus* sp. and *Anaeromyxobacter* sp., encode putative BamB sequences (NP_968885.1, YP_003268123.1, YP_004668368.1 and YP_001379339.1 respectively) with *E*-value scores of < 10^−8^, an observation that is perhaps best explained by a lateral gene transfer event – in the case of *B. bacteriovorus* the GC content at the locus encoding NP_968885.1 is consistent with this idea (Fig. S2). The most striking finding from the HMM searches is the complete absence of any BamC-related sequences from α-, δ- and ε-proteobacteria (Table 1).

### Identification of BamF

While it is not essential for cell viability, BamC is found in all species of β- and γ-proteobacteria. Why then are no sequences related to BamC detected in any species of α-, δ- or ε-proteobacteria? This absence of BamC raised the question of whether or not these organisms might have substituted proteins with analogous function, but with a non-conserved primary structure, into the BAM complex. To address this question we used the model organism *C. crescentus* and purified the BAM complex by immunoprecipitation.

**Fig. 2:**
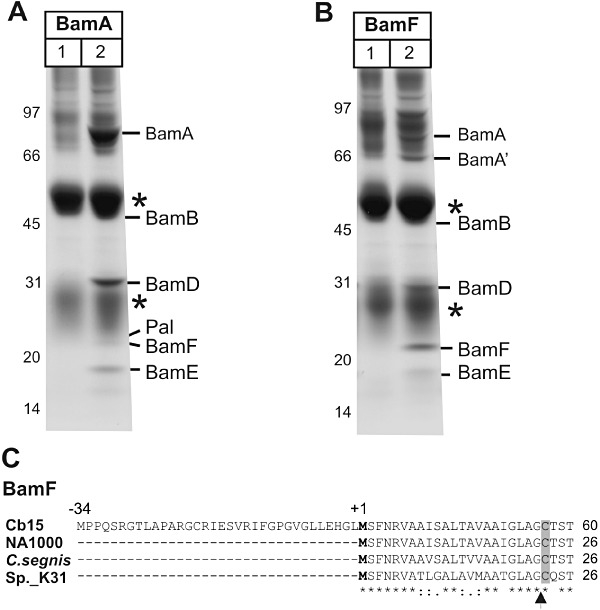
BamF is a subunit of the BAM complex in *C. crescentus*. A. Outer membrane vesicles were solubilized with 0.75% DDM and subject to immunoprecipitation using pre-immune serum (lane 1) or using BamA anti-serum (lane 2), and analysed by SDS-PAGE and Coomassie blue staining. Asterisks indicate the IgG heavy and light chains. Mass spectrometry was used to identify the indicated components of the BAM complex, and the novel protein BamF. B. Outer membrane vesicles were solubilized with 0.75% DDM and subject to immunoprecipitation using pre-immune serum (lane 1) or using BamF anti-serum (lane 2), and analysed as described above. BamA′ designates a 66 kDa protein fragment of BamA. C. The BamF sequences from *C. crescentus* strains CB15 and NA1000, *Caulobacter segnis* and *Caulobacter* sp. 31 are shown. The genome sequence from CB15 is unique in having an in-frame ATG codon (corresponding to Met ‘−34’) upstream of the predicted start site in all other *Caulobacter* genomes. The predicted processing sites for Signal peptidase II is indicated with an arrow-head. Asterisks indicate sequence identity across the alignment and the cysteine residue predicted to be lipid-modified is shaded grey.

Purified outer membranes from *C. crescentus* were solubilized in 0.75% (w/v) dodecylmaltoside, and the solubilized membrane proteins were incubated with the BamA antiserum. After immunoprecipitation, the core components BamA, BamB, BamD and BamE were identified by mass spectrometry (Anwari *et al*., 2010; Fig. 2A). A novel protein encoded by the *cc0699* gene co-purifies with the BAM complex, and we refer to it hereafter as BamF. On SDS-PAGE, BamF migrates at ∼ 22 kDa, the same size as the BAM complex ancillary factor Pal/YiaD (Fig. 2A).

Antibodies raised to BamF immunoprecipitate the subunits of the core BAM complex (Fig. 2B), validating the interaction between BamF and the BAM complex. A 66 kDa fragment of BamA is more prevalent in the immunoprecipitations where BamF is the binding site for the antibody, compare Fig. 2B and A. The reason for this is not known, but we note that in *E. coli* BamA has been observed to exist in two distinct functional states: one more protease sensitive than the other (Rigel *et al*., 2012). The 66 kDa fragment of BamA has affinity enough for BamF so as to co-immunoprecipitate.

The BamF protein has the characteristic sequence features of a lipoprotein: LipoP predicts processing by Signal peptidase II, and the threonine residue at position 2 from the processing site would allow BamF to be targeted to the outer membrane by the Lol system (Tokuda and Matsuyama, 2004; Lewenza *et al*.*,* 2008). After processing at the conserved cysteine residue (Fig. 2C), the predicted molecular size of BamF (17.5 kDa) is similar to that of the processed lipoprotein Pal (17.4 kDa). The aberrant, but consistent, migration of both BamF and Pal proteins at ∼ 22 kDa is perhaps due to the lipid modifications on the lipoproteins. In the genome data from *C. crescentus* strain CB15, a longer open reading frame has been annotated (Fig. 2C). Whether or not this appendage to the signal sequence of BamF exists in that strain, it is not coded for in the genomes of *C. crescentus* strain NA1000 or in *Caulobacter segnis* (ATCC 21756) or *Caulobacter* sp. K31. In the CB15 genome sequence, a consensus Shine–Dalgarno sequence (5′-GGAGCA-3′) sits upstream of the codon for the methionine residue indicated as ‘+1’ in Fig. 2C. In all four BamF sequences, the N-terminal ∼ 50 residues of the processed lipoprotein (i.e. C^23^-E^68^) are predicted to be intrinsically disordered, and the BamF sequence conforms to a conserved Pfam domain (DUF3035) of unknown function, which is found only in α-proteobacteria. In terms of gene synteny, the *cc0699* gene encoding BamF sits immediately downstream of the gene (*cc0700*) encoding Signal peptidase II.

### BamF is an outer membrane protein

**Fig. 3:**
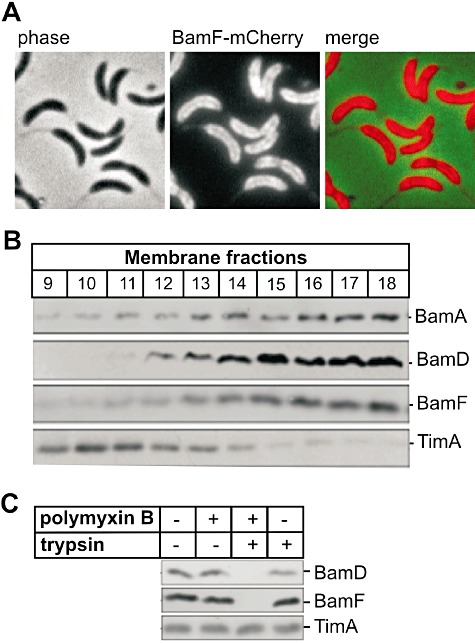
BamF is an outer membrane protein with a periplasmic domain. A. Micrographs of *C. crescentus* cells (CJW3125) producing a BamF–mCherry fusion in place of wild-type BamF. B. Outer membrane vesicles were solubilized with 0.75% DDM and subject to sucrose gradient fractionation. The gradients were fractionated and membrane proteins analysed by SDS-PAGE and immunoblotting with antisera to BamF, the outer membrane proteins BamA and BamD and the inner membrane protein TimA. C. Protease shaving of *C. crescentus* JS1014 cells. Cells were incubated with (lanes 2, 3) and without (lanes 1, 4) polymyxin B followed by addition of trypsin (lanes 3, 4). TCA-precipitated samples were then analysed by SDS-PAGE and immunoblotting with antibodies against BamF, BamD and TimA.

We constructed strain CJW3125 of *C. crescentus* to express a BamF–mCherry fusion in place of wild-type BamF. Fluorescence microscopy revealed a membrane-localization pattern (Fig. 3A). To demonstrate that BamF localizes to the outer membrane (as opposed to the inner membrane), membranes were prepared from wild-type *C. crescentus* CB15N and separated on sucrose density gradients: BamF co-migrates on the gradients with BamA and BamD, distinct from the inner membrane protein TimA (Clements *et al*., 2009) (Fig. 3B). To determine the topology of BamF, a trypsin shaving assay was used. Wild-type *C. crescentus* CB15N has a surface-coating proteinaceous ‘S-layer’ that, along with the smooth LPS that attaches the S-layer could potentially block trypsin access to outer membrane proteins. We therefore made use of strain JS1014 which lacks this S-layer, as well as the smooth LPS (Ford *et al*., 2007). Cells were incubated in the presence of trypsin, with or without polymyxin B. Polymyxin B permeates the outer membrane allowing trypsin access to the periplasmic space (Clements *et al*., 2009): only in the presence of polymyxin B did trypsin degrade BamD and BamF, demonstrating that both proteins are accessible in the periplasm (Fig. 3C).

### BamF is found exclusively in α-proteobacteria

**Fig. 4:**
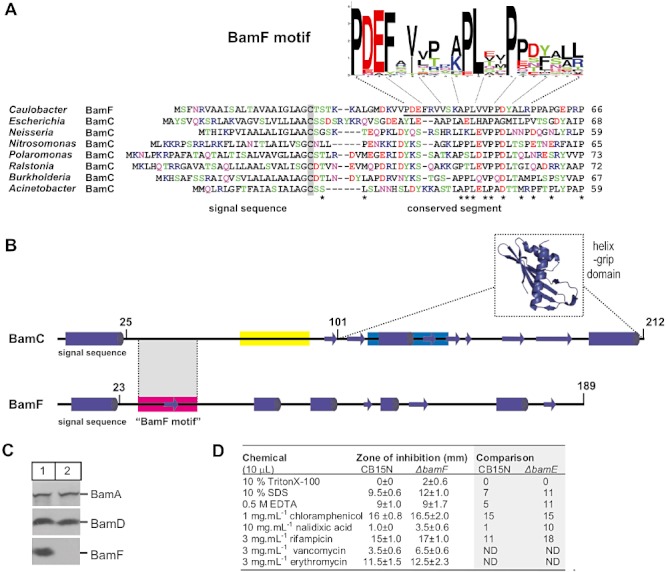
Conserved sequence in the N-terminal, disordered regions of BamF and BamC. A. clustalw alignment of the N-terminal sequences of BamF from *C. crescentus* and BamC homologues from indicated species of β- and γ-proteobacteria. The sequence corresponding to the ‘BamF motif’ is underlined. The BamF motif is presented as a Sequence Logo: the height of the letter representing each amino acid residue is proportional to how well conserved each residue is across the collection of 51 BamF sequences (see Fig. S2). Amino acid residues are coloured according to chemical properties (basic = blue; acidic = red; hydroxylated = green, relatively hydrophobic = black). Grey shading indicates the presumed N-terminal lipidated cysteine residue after processing of the signal peptide, asterisks highlight positions at which there is some conservation of sequence across the aligned sequences. B. The sequence of BamC from *E. coli* and BamF from *C. crescentus* were each submitted to JPred (http://www.compbio.dundee.ac.uk/www-jpred/) for secondary structure prediction, and beta-strands (arrows) and alpha-helices (cylinders) are shown accordingly. The second helix-grip domain of BamC has been deleted from this representation, with the structure of the first helix-grip domain (which corresponds to residues 101–212 of BamC; pdb 2LAF) shown as an inset. The position of BamF motif and two motifs that are conserved only within the BamC family of proteins (shown in yellow and blue) are coloured consistently with the full data presented in Fig. S3. C. The *bamF* gene was deleted in a CB15N strain background (*Experimental procedures*). *C. crescentus* CB15N and Δ*bamF* strains were grown in culture and cell extracts prepared for SDS-PAGE and immunoblotting. D. *C. crescentus* CB15N and the isogenic Δ*bamF* mutant were grown on solid medium in the presence of the indicated detergents or antibiotics and the zone of inhibition was measured between the edge of the disc and the bacterial lawn. The results are the average and standard deviation error of three independent experiments and the data are compared with that seen with a Δ*bamE* mutant in a previous study (Ryan *et al*., 2010).

BamF has homologues in all species of α-proteobacteria, with no homologues detected in other bacterial lineages. However, blast searches identified a short region at the N-terminus of BamF with similarity to an equivalent region in BamC (Fig. 4A). To determine whether a conserved motif might be present in this region of BamF proteins, MEME analysis (Bailey *et al*., 2006) was undertaken allowing for ‘zero or one motif’ in each of the 90 BamC and 51 BamF sequences (see *Experimental procedures*). A BamF motif (Fig. 4A) was detected in the N-terminal region, and the same motif was found in several β-proteobacterial BamC homologues (Fig. S3). In addition to the sequence similarity evident in comparison with BamF (Fig. 4A), the γ-proteobacterial BamC proteins, such as that found in *E. coli*, contain a distinct, dominant motif in the adjoining N-terminal region and several motifs that describe features of the ‘helix-grip’ domains (Fig. S3). These BamC-specific regions are highlighted in Fig. 4B, with the predicted secondary structure of both BamC from *E. coli* and BamF from *C. crescentus* shown. Since there is neither conservation in the motifs nor the secondary structure predictions, there is as yet no evidence to suggest that BamF and BamC have a similar fold apart from the unstructured N-terminal regions found in both proteins.

After processing of the signal sequence and lipid modification of the N-terminal cysteine residue, the conserved, N-terminal region of the BamC and BamF proteins would be proximal to the outer membrane, and recent structural analysis demonstrated that this N-terminal region of BamC from *E. coli* is responsible for its interaction with the BamD subunit (Kim *et al*., 2011). A ‘control’ experiment using MEME to find common motifs from within a set of 98 predicted *C. crescentus* outer membrane lipoproteins (see *Experimental procedures*) showed no common motifs, indicating that the conserved N-terminal region in BamC and BamF is not simply a common signature in the N-terminal regions of outer membrane lipoproteins. We suggest that proteins of the BamC- and the BamF-protein families each contain a short, membrane proximal sequence motif to facilitate their interaction with the BAM complex.

Cellular sensitivity to various detergents and antibiotics was tested as a means to judge membrane integrity of a *C. crescentus* strain lacking BamF*.* The Δ*bamF* mutant strain is viable and has similar steady-state levels of BamA and BamD as the wild-type strain (Fig. 4C). Compared with wild-type CB15N, the Δ*bamF* strain displayed minor increases in susceptibility to antibiotics and detergents, suggesting mild defects in outer membrane permeability (Fig. 4D). In a previous study, deletion of the gene encoding BamE in *C. crescentus* CB15N strain resulted in changes to chemical sensitivity and hence BamE was implicated in the stabilization of the BAM complex and changes in outer membrane permeability (Ryan *et al*., 2010). These values are noted in Fig. 4D by way of comparison.

### Modules comprising the BAM complex

**Fig. 5:**
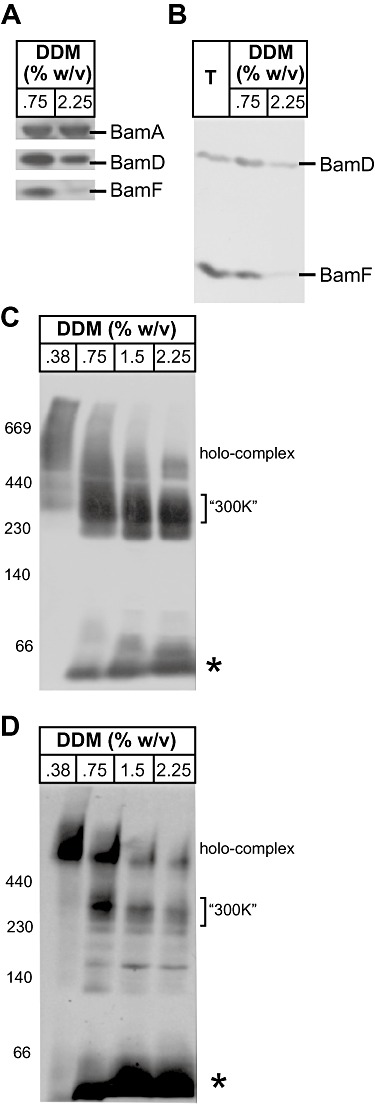
BamF is in a modular complex and can be displaced from the BAM complex. A. Outer membrane vesicles were solubilized with DDM and subject to immunoprecipitation with antibodies recognizing BamA. The samples were analysed by SDS-PAGE and immunoblotting. B. Outer membrane vesicles (equivalent to 4 µg total membrane protein, shown as ‘T’) were solubilized with either ‘low’ DDM (0.75% w/v) or ‘high’ DDM (2.25% w/v) and subject to immunoprecipitation with antibodies recognizing BamA. The samples were analysed by SDS-PAGE and immunoblotting, probed with antibodies recognizing BamD and BamF, and subject to phosphorimage analysis. C and D. Outer membrane protein complexes were solubilized with the indicated concentrations of DDM and separated by BN-PAGE and: (C) immunoblot analysis using anti-serum against BamD, or (D) immunoblot analysis using anti-serum against BamF. The electrophoretic mobility of the ‘∼ 500K’ holo-complex and ‘300K’ core complex (Anwari *et al*., 2010) is indicated. Asterisk notes the position of BamD and BamF co-migrating in a species of ∼ 50 kDa.

We previously demonstrated the modular construction of the BAM complex, using detergent titration of membranes analysed by blue native polyacrylamide gel electrophoresis (BN-PAGE) (Anwari *et al*., 2010). With antibodies to BamD and BamF available, we sought to better characterize the modular structure of the BAM complex. Immunoprecipitation with antiserum recognizing BamA showed (i) that all of the BamF present in the outer membrane is present in the BAM complex and (ii) that in samples solubilized under either ‘low’ (0.75%) dodecylmaltoside (DDM) or ‘high’ (2.25%) DDM, there is a displacement of much of BamD (∼ 70 %) and BamF (∼ 90%) from the core BAM complex (Fig. 5A and B). This shows that in low DDM, both BamD and BamF are docked in the BAM complex. When outer membranes were titrated with increasing amounts of DDM and separated by BN-PAGE, immunoblotting revealed that BamD was present in the holo-complex of ∼ 500 kDa (Fig. 5C). Increasing the detergent concentration at or above 0.75% DDM disrupts the holo-complex, resulting in a ∼ 300 kDa core complex as the major species as well as faster migrating forms of BamD (Fig. 5C). In equivalent experiments focused on BamA, a ∼ 150 kDa module of BamA was detected (Anwari *et al*., 2010), but the 150 kDa form does not contain BamD (Fig. 5C). When antiserum recognizing BamF was used to probe the same samples, the same BAM complex modules were identified as seen for BamD (Fig. 5D). Taken together, the immunoprecipitation and BN-PAGE results suggest that BamF is released from the BAM complex under similar conditions that promote release of BamD.

## Discussion

The HMM data enable a clear generalization to be made about distribution of subunits of the BAM complex across each proteobacterial class (Table 1). There are a few exceptions such as the presence of BamB in *B. bacteriovorus* HD100 and *H. ochraceum* DSM 14365. However, given the propensity for lateral gene transfer across species of proteobacteria (Gupta and Griffiths, 2002; Bapteste *et al*., 2004), the rarity of these observed exceptions serves to validate the generalization and help formulate a model for BAM complex evolution.

For a few species secondary losses are also noteworthy. For example, BamB has previously been noted as absent from the BAM complex in the β-proteobacterium *Neisseria meningitidis* (Volokhina *et al*., 2009; Tellez and Misra, 2012). Our comprehensive assessment of β-proteobacterial species shows that BamB is selectively missing from all sequenced strains of *N. meningitidis, N. gonorrhoeae* and *N. lactamica*; but that all other β-proteobacterial genomes encode a homologue of BamB. The reasons for such reduction in function in the genus *Neisseria* is not clear but, again, it is a rare exception to the rule. Secondary acquisition through duplication can also be found, with all sequenced species of the β-proteobacterial genus *Burkholderia* having two genes encoding BamC; *Burkholderia* is notable in its capacity for gene duplication as recently documented for the RND family of proteins (Perrin *et al*., 2010), and is the only genus determined to have two BamC-coding genes.

The cut-off scores used for the HMM were designed to capture both genuine homologues of the Bam lipoproteins and ‘related’ proteins. Related proteins were defined as those with *E*-value scores in the range e-05 to e-08, with this arbitrary definition potentially detecting the following: (i) proteins of homologous function but for which sequence divergence is very great, and (ii) proteins with a distinct function, which might share some evolutionary relationship to the Bam lipoprotein. For example, the HMM analysis suggests that the BamB lipoprotein was derived after the divergence of the δ-/ε- and α-/β-/γ-proteobacterial lineages, and may have evolved from cytoplasmic dehydrogenases: a set of these are detected by the BamB HMM in the broadest range of proteobacterial species. The beta-propeller fold shared by these proteins is a common framework requiring very little evolutionary tinkering to convert enzymatic functions to scaffolding functions (Chen *et al*., 2011). The specific role of BamB in the BAM complex is not entirely clear, but it may provide a scaffolding function, to assist in substrate folding (Noinaj *et al*., 2011).

### A model for the evolution of the BAM complex

**Fig. 6:**
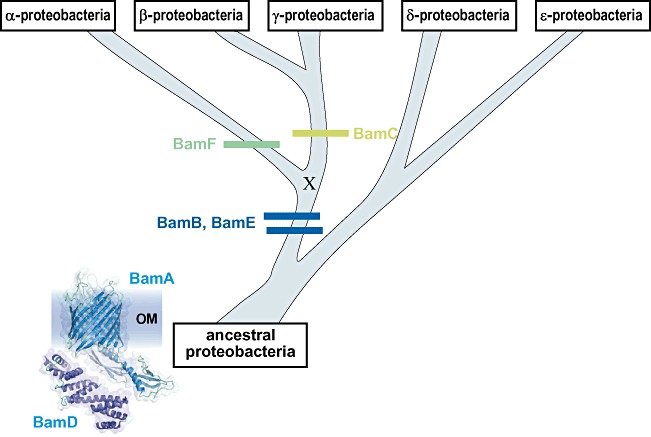
The evolution of the BAM complex. The order of divergence for each of the five subclasses of proteobacteria is based on rRNA and RNA polymerase gene phylogenies (Klenk *et al*., 1999; Gupta, 2000). As BamA and BamD are ubiquitous in all five subclasses of proteobacteria, and found also in non-proteobacteria, it can be assumed they were both present in the ancestral proteobacterial species. It cannot be known whether other proteins assisted this ancestral, core BAM complex. The order of acquisition for BamB, BamC and BamE is according to their distribution in Table 1. A potential common ancestor for BamC and BamF has been indicated with an ‘X’ and is discussed in the text. However, given the available evidence, the most parsimonious model would be for an independent acquisition of BamF and BamC in the two lineages. In either case, the BamF- and BamC-protein families arise as indicated in green.

Bacterial rRNA and RNA polymerase gene phylogenies suggest that the most ancient subclasses are the δ- and ε-proteobacteria, from which evolved the α-, then β-, and most recently the γ-proteobacterial lineages (Woese, 1987; Olsen *et al*., 1994; Klenk *et al*., 1999; Gupta, 2000). The distribution of lipoprotein subunits from the BAM complex is consistent with a very simple model for the evolution of the BAM machinery, with a BamA–BamD ancestral complex in the earliest proteobacteria (Fig. 6). This model shows the acquisition of BamB and BamE before the divergence of the lineage that gave rise to α-proteobacteria, and thereafter the evolution of BamC in the lineage that gave rise to the β- and γ-proteobacteria.

The only lipoprotein subunit of the BAM complex to be detected ubiquitously in proteobacteria is BamD. This is in keeping with the experimental observation that the *bamD* gene is essential for viability in γ-proteobacteria (Malinverni *et al*., 2006), β-proteobacteria (Volokhina *et al*., 2009) and α-proteobacteria (this study), and with the identification of BamD homologues in non-proteobacterial groups of bacteria such as the Bacteriodetes/Chlorobi group (Sandoval *et al*., 2011).

An uncertainty in the evolutionary model concerns the placement of BamC and BamF. We suggest here that only after the divergence of the α- and β-/γ-proteobacterial lineages did the BamC lipoprotein evolve. This proposition is evidenced by its exclusive distribution in species of β- and γ-proteobacteria and its complete absence from δ-, ε- and α-proteobacteria. According to this model in the α-proteobacterial lineage, instead, evolved BamF. Until the function of both BamC and BamF are better understood, the proposition that BamF and BamC might fulfil similar functions cannot be directly tested.

An alternative model is possible in which a common ancestor gave rise to both BamC and BamF. This hypothetical ancestor is indicated with an ‘X’ in Fig. 6. While we cannot ignore this possibility, two points make this model less attractive: (i) the HMM analysis used here is sufficiently sensitive to find relatedness such as that for BamB lipoproteins and dehydrogenases, and also BamD/YbgF lipoproteins and BamE/OsmE lipoproteins, yet finds no relatedness between the BamF- and BamC-protein families and (ii) the structure of BamC reveals two characteristic ‘helix-grip’ domains (Albrecht and Zeth, 2011; Kim *et al*.*,* 2011; Warner *et al*.*,* 2011), but secondary structure predictions on BamF sequences suggest a very different architecture. In confirmation of this, MEME analysis of BamC sequences shows that right across the species of γ- and β-protein bacterial species, motifs defining elements in the helix-grip domains are conserved, and yet never found in any α-proteobacterial BamF sequence (Fig. S3). A common ancestor can never be ruled out in a situation such as this, but we believe that the most parsimonious explanation in this case is that BamC and BamF evolved independently.

Our discovery of BamF in the α-proteobacterial BAM complex is of further significance because it suggests that additional proteins can evolve to ‘fill’ a role in the BAM complex of a lineage. Thus, while the ancestral δ-/ε-proteobacterial lineage may have simply had only BamA and BamD, extant species of δ- and ε-proteobacteria may well have evolved additional proteins to fulfil the roles played by BamB, BamC/BamF and BamE. Biochemical analysis of model species of δ- and ε-proteobacteria therefore promises new knowledge on the assembly of bacterial outer membranes.

## Experimental procedures

### HMM analyses

Hidden Markov model analyses were carried out as previously described (Likic *et al*., 2010). Specifically, (i) bacterial genome sequences were downloaded (all.faa.tar.gz) from NCBI ftp site http://ftp://ftp.ncbi.nlm.nih.gov/genomes/Bacteria/ on 28 November 2011, (ii) complete genome sequences were selected and their taxonomy determined via lproks_0.txt (lproks_0.txt file is available from the NCBI ftp site), and (iii) the number of genomes in each class of proteobacteria was tabulated. HMMs describing each subunit of the BAM complex were built with HMMER-2.3.2 version 11 (http://hmmer.janelia.org/; Eddy, 1998) using sequences as previously described (Gatsos *et al*., 2008). Bacterial genomes were scanned using HMMER-2.3.2 version 11. The scans were carried out with a non-conservative cut off *E*-value of 10^−5^ (Likic *et al*., 2010), with ‘perfect’*E*-value scores stated as ‘0’ (corresponding to < e −300). All detected protein sequences were extracted using Yabby-0.1 (http://code.google.com/p/yabby/). Full sequence data are provided as Table S2.

### Strains and growth

For growth of *C. crescentus*, peptone yeast extract (PYE) medium was prepared as previously described (Poindexter, 1964). Unless otherwise indicated, *C. crescentus*, cultures were grown under aerobic conditions at 30°C shaking at 120 r.p.m. in 5 l baffled flasks to an optical density (OD)_600_ of 0.7.

All plasmids and strains used are documented in Table 2, and the wild-type strain of *C. crescentus* used in this study is CB15N.

**Table 2:** Plasmids and strains used in this study

	Description	Reference
**Plasmid**		
pRXMCS	pMB1 replicon with *oriT* and *xylXp* promoter	Thanbichler *et al.* (2007)
pNPTS138	Kan^r^; *sacB*-containing integration vector	Spratt *et al*. (1986)
pBOR	2 kb EcoRI fragment from pHP45Ω[vector carrying a Spec^R^/Strep^R^ cassette (Ω)] cloned into EcoRI-digested pBluescript	Collier *et al*. (2007)
pCHYC4	pMB1 replicon with *oriT* and *mCherry*	Thanbichler *et al.* (2007)
pBgent	GentR variant of pBGS18T	Matroule *et al*. (2004)
**Strain**		
CB15N	*C. crescentus* wild-type strain, synchronizable variant strain of CB15; also known as NA1000	Evinger and Agabian (1977)
JS1014	*C. crescentus* strain lacking S-layer: has deletions of *rsaA*, the gene responsible for S-layer biosynthesis and *manB*, a gene in a biosynthetic pathway common to both LPS O-side chain and exopolysaccharide synthesis	Ford *et al.* (2007)
KA3	*C. crescentus* strain in which the *bamD* gene is under the control of a xylose-inducible promoter	This study
KA4	*C. crescentus* strain in which the *bamF* gene has been deleted	This study
CJW3125	*C. crescentus* strain expressing a BamF–mCherry fusion protein under the control of the *bamF* promoter	This study
S17-1	*E. coli* strain (ATCC47055): RP4-2 *Tc*::*Mu KM*-Tn*7*, for plasmid mobilization into *C. crescentus*	Simon *et al*. (1983)
BL21(DE3)	*E. coli* strain: F−*ompT hsdS*B (rB− mB−) *dcm gal* (DE3), recombinant expression of his-tagged BamF and BamD for antibody production and biochemical analysis	Invitrogen

The *C. crescentus* strain CJW3125 was engineered by amplifying the last 381 bp from the 3′-end of the *cc0699* gene with primers CC0699F (5′-CAGGTACCGAGCCGCGCCCGCAGGAACTG-3′) and CC0699R (5′-CAGAATTCCCCAGGCCCGGGAGCTTGATGCC-3′). The PCR product was then digested with EcoRI and KpnI and cloned into pCHYC4 (Thanbichler *et al*., 2007). The resulting plasmid was electroporated into CB15N cells. Integration of the plasmid by single cross-over with the chromosomal copy of the *cc0699* gene (renamed *bamF*) was then selected by antibiotic resistance. As a result, strain CJW3125 produces a BamF–mCherry protein fusion under the control of the native promoter.

The *C. crescentus* strain KA4 was engineered by amplifying a region of the *cc0699* gene with primers BamFKOForXbaI (5′-GCCGTCTAGAAGTTTCAACCGGGT-3′) and BamFKORevHindIII (5′-ATCGAAGCTTAGGGTCTTCAGGCG-3′) and the PCR product was digested with XbaI and HindIII and cloned into pBgent. The resulting plasmid was electroporated into CB15N cells. Integration of the plasmid by single cross-over with the chromosomal copy of the *cc0699* gene (renamed *bamF*) was then selected by gentamicin resistance.

KA3 is a BamD-depletion strain of *C. crescentus* and was generated by double-homologous recombination with a pNPBamD^SpecR^ construct, while *bamD* was expressed from a complementing *bamD-*pRXMCS plasmid. To form the pNPBamD^SpecR^ construct, regions upstream and downstream of *bamD* were amplified by PCR with the following primers: UpstreamForHindIII: 5′-TACGAAGCTTCTGCGCGCCATCGGTCT-3′, UpstreamRevEcoRI: 5′-GCGCGAATTCACTTCGCGGAAATAGTC-3′, DownstreamForEcoRI: 5′-AGTCGAATTCACTTCCCGGGCG-3′, and DownstreamRevNheI: 5′-ACTAGCTAGCCGAACGACCGTC-3′. These fragments were ligated into pNPTS138 to form pNPBamD. A cassette carrying spectinomycin resistance was amplified from pBOR using PCR with primers: SpectinomycinFor: 5′-CGGCCTGCAGAGTGGATCCCCCGGGCTGCA-3′ and SpectinomycinRev: 5′-CGGCGCTAGCGGTATCGATAAGCTTGATAT-3′. The PCR product was digested with EcoRI and ligated into pNPBamD. When double recombination did not occur due to gene essentiality, single recombinants were mated with S17-1 cells harbouring the replicating plasmids encoding BamD (*bamD-*pRXMCS). These transformed single recombinants were spread onto plates supplemented with 3% (w/v) sucrose and 0.03% (w/v) xylose. Resultant colonies were used to seed cultures containing 0.03% (w/v) xylose to maintain *bamD* gene expression from the pRXMCS vector. Clones were verified by antibiotic selectivity, and by immunoblot analysis of BamD after growing cells in glucose (0.2%) or xylose (0.03%) to repress or induce the gene promoter respectively. After 16 h growth on glucose-containing medium, no viable colonies were recovered on plates containing xylose.

### Subcellular fractionation of *C. crescentus*

We previously developed a method to purify outer and inner membranes from *C. crescentus* that involved fractionation on a six-step sucrose gradient [35:40:45:50:55:60% (w/v) sucrose in 5 mM EDTA, pH 7.5] by centrifugation (Anwari *et al*., 2010). All steps were performed at 4°C and outer membranes were stored at −80°C.

### Protease accessibility

Cultures of *C. crescentus* were grown and harvested at mid-log growth phase and washed twice with 50 mM Tris-HCl, pH 8.0. Cells were incubated with either polymyxin B (final concentration 2 mg ml^−1^) or 50 mM Tris-HCl, pH 8.0 (control) for 10 min. Cells were digested with trypsin (final concentration 0.1 mg ml^−1^) for 30 min at 4°C. Proteolysis was stopped by adding soybean trypsin inhibitor (STBI) to a final concentration of 0.5 mg ml^−1^. Prior to analysis by SDS-PAGE, 2× SDS sample buffer was added and samples were heated at 95°C for 5 min.

### Immunological methods, electrophoresis and mass spectrometry

Antibody production, immunoprecipitation analysis, SDS-PAGE and BN-PAGE were carried out as previously described (Anwari *et al*., 2010). Protein-containing bands from SDS gels were manipulated and analysed by liquid chromatography-tandem MS (LC-MS/MS) using a Shimadzu Prominence nano flow HPLC and an Applied Biosystems Q-STAR ELITE mass spectrometer as previously described (Purcell *et al*., 2001; Anwari *et al*., 2010).

### Microscopy

Fluorescence microscopy images were obtained using NIKON E1000 equipped with a Hamamatsu ORCA ER camera. Cells were grown to an OD_660_ between 0.2 and 0.3 in minimal (M2G) medium and immobilized on an agarose-padded slide containing the same growth medium. Images were taken and processed by Metamorph 7.1.4 software and processed with ImageJ software.

For SEM, the BamD-depletion strain of *C. crescentus* was grown at 30°C for 16 h in the presence of 0.03% (w/v) xylose or 0.2% (w/v) glucose to an OD_600_ of 0.15, harvested by centrifugation at 10 000 *g* for 10 min at 4°C and rinsed with 1× PBS. The cell pellet was resuspended in 3% (v/v) gluteraldehyde solution in 0.1 M phosphate buffer, pH 7.2 and fixed for 24 h. Cells were placed on sterile Thermanox plastic coverslips (Pro SciTech) for 10 min, then the coverslips were washed twice with 0.1 M sodium cacodylate, pH 7.2. Cells were dehydrated into ethanol in 10 min steps, using 70%, 90% and 100% (absolute) ethanol solutions. These samples were then treated with hexamethyldisilazane in 100% (absolute) ethanol for 10 min each, starting with a 25% solution continuing through 50%, 75% and 100% solutions, and then coated with gold palladium. Observation and photomicrographs were then carried out with a Hitachi S-3400 N SEM (Hitachi Instrument, Japan). Image J was used to analyse the SEM images.

### Circular dichroism

BamD from *R. marinus* was previously expressed in recombinant form in *E. coli* to generate a crystal structure for the protein (Sandoval *et al*.*,* 2011), we used the same strategy for the expression of BamD from *C. crescentus.* Briefly, BamD was truncated to remove the signal sequence and conserved Cys residue and was expressed in *E. coli* with a C-terminal hexahistidine tag to aid purification by nickel-affinity chromatography and gel filtration. CD analyses were performed using a Jasco J-815 spectrometer. Far-UV CD spectra from 190 to 260 nm were acquired at 20°C in a 1 mm path-length cuvette, with a 1 nm bandwidth, 1 s response time and 100 nm min^−1^ scan rate. The protein concentration of the sample was 0.288 mg ml^−1^ BamD in 1.5 M Urea, 50 mM Tris-HCl pH 7.0. The protein concentration was determined using the calculated extinction coefficient *ε*_M_ = 48.82 × 10^−3^ M^−1^ cm^−1^ and the molecular weight 32 853.1 Da of purified BamD. The spectrum, representing the average of three scans, was baseline corrected by subtracting the spectral attributes of the buffer. Spectra Manager (Version 2.08.02) was used to smooth the data using the Savitzky-Golay method for smoothing with a convolution width of 25. Raw data (in millidegrees) sere converted to ellipticity (degrees cm^2^ dmol^−1^) by calculating the mean residue weight using the molecular weight of BamD as 32 853.1 Da and the number of amino acids of BamD as 289. The CDPro program (Sreerama and Woody, 2004) was utilized to assess the secondary structure contents of the proteins from the spectrum.

### Chemical sensitivity assays

Disc diffusion assays were performed as previously described (Ryan *et al*., 2010) with the following exceptions. Overnight cultures of CB15N and Δ*bamF::gent* grown in PYE medium or PYE medium supplemented with 0.5 µg ml^−1^ gentamicin respectively were diluted to OD_600_ 0.7–0.8 units ml^−1^, combined with 4 ml of molten PYE medium agar and poured over PYE medium agar plates. The zone of inhibition was measured between the edge of the 6 mm disc and the bacterial lawn after 24 h incubation at 30°C. Each experiment was repeated at least three times.

### Signal sequence and secondary structure predictions

Lipoprotein signal sequence features (Juncker *et al*.*,* 2003) were predicted using the LipoP v1.0 server (http://www.cbs.dtu.dk/services/LipoP/). The scores for BamF indicate a high probability of processing by Signal peptidase SpII and location to the outer membrane (score = 18.5495, margin = 13.34608 cleavage = 22–23 Pos+2 = T). For structure-based comparisons of BamD, representative sequences were first analysed for an outer membrane localization signal sequence using LipoP v1,0. These signal sequences were deleted, and the ‘mature’ sequences then aligned using clustalx (Larkin *et al*.*,* 2007). Secondary structure for each sequence was predicted using the Strap software package (http://www.bioinformatics.org/strap) and TPR elements were located using the TPRPred online webserver (http://toolkit.tuebingen.mpg.de/tprpred).

### Motif analyses

To define the regions of BamC and BamF that have remained conserved during evolution, motif analysis using MEME was employed. First, a set of BamC homologues was compiled by searching the NCBI nr database (15-10-2008) with HHsenser, starting with *E. coli* BamC (P0A903) as a query sequence. A set of BamF sequences was compiled in the same way, using *C. crescentus* BamF as a query sequence. To prevent inadvertent bias in motif detection, sequences from metagenomic studies and other truncated sequences were discarded, and the collected sequences were manipulated to remove the N-terminal signal sequence and lipid attachment Cys, MEME (v4.6.1) was employed for motif discovery in ‘zoops’ (zero or one motif per sequence) and ‘oops’ (one motif per sequence) mode (-maxsize 1000000 -maxw 80 -nmotifs 5), to find conserved motifs within the total set of BamC and BamF sequences. For control experiments, a collection of 98 putative outer membrane lipoprotein sequences was generated, using LipoP to detect the sequences and manually removing those with the canonical (aspartate) outer membrane avoidance signal.

## References

[b1] Albrecht R, Zeth K (2011). Structural basis of outer membrane protein biogenesis in bacteria. J Biol Chem.

[b2] Anwari K, Poggio S, Perry A, Gatsos X, Ramarathinam SH, Williamson NA (2010). A modular BAM complex in the outer membrane of the alpha-proteobacterium *Caulobacter crescentus*. PLoS ONE.

[b3] Bailey TL, Williams N, Misleh C, Li WW (2006). MEME: discovering and analyzing DNA and protein sequence motifs. Nucleic Acids Res.

[b4] Bapteste E, Boucher Y, Leigh J, Doolittle WF (2004). Phylogenetic reconstruction and lateral gene transfer. Trends Microbiol.

[b5] Bos MP, Robert V, Tommassen J (2007). Functioning of outer membrane protein assembly factor Omp85 requires a single POTRA domain. EMBO Rep.

[b6] Cavalier-Smith T (2006). Rooting the tree of life by transition analyses. Biol Direct.

[b7] Chen CK, Chan NL, Wang AH (2011). The many blades of the β-propeller proteins: conserved but versatile. Trends Biochem Sci.

[b8] Clements A, Bursac D, Gatsos X, Perry AJ, Civciristov S, Celik N (2009). The reducible complexity of a mitochondrial molecular machine. Proc Natl Acad Sci USA.

[b9] Collier J, McAdams HH, Shapiro L (2007). A DNA methylation ratchet governs progression through a bacterial cell cycle. Proc Natl Acad Sci USA.

[b10] D’Andrea LD, Regan L (2003). TPR proteins: the versatile helix. Trends Biochem Sci.

[b11] Eddy SR (1998). Profile hidden Markov models. Bioinformatics.

[b12] Evinger M, Agabian N (1977). Envelope-associated nucleoid from *Caulobacter crescentus* stalked and swarmer cells. J Bacteriol.

[b13] Ford MJ, Nomellini JF, Smit J (2007). S-layer anchoring and localization of an S-layer-associated protease in *Caulobacter crescentus*. J Bacteriol.

[b14] Gatsos X, Perry AJ, Anwari K, Dolezal P, Wolynec PP, Likic VA (2008). Protein secretion and outer membrane assembly in Alphaproteobacteria. FEMS Microbiol Rev.

[b15] Gentle IE, Gabriel K, Beech P, Waller R, Lithgow T (2004). The Omp85 family of proteins is essential for outer membrane biogenesis in mitochondria and bacteria. J Cell Biol.

[b16] Gupta RS (2000). The phylogeny of proteobacteria: relationships to other eubacterial phyla and eukaryotes. FEMS Microbiol Rev.

[b17] Gupta RS, Griffiths E (2002). Critical issues in bacterial phylogeny. Theor Popul Biol.

[b18] Hagan CL, Kim S, Kahne D (2010). Reconstitution of outer membrane protein assembly from purified components. Science.

[b19] Juncker AS, Willenbrock H, von Heijne G, Nielsen H, Brunak S, Krogh A (2003). Prediction of lipoprotein signal peptides in Gram-negative bacteria. Protein Sci.

[b21] Kim KH, Paetzel M (2010). Crystal structure of *Escherichia coli* BamB, a lipoprotein component of the β-barrel assembly machinery complex. J Mol Biol.

[b20] Kim KH, Aulakh S, Paetzel M (2011). Crystal structure of β-barrel assembly machinery BamCD protein complex. J Biol Chem.

[b22] Klenk HP, Meier TD, Durovic P, Schwass V, Lottspeich F, Dennis PP, Zillig W (1999). RNA polymerase of *Aquifex pyrophilus*: implications for the evolution of the bacterial rpoBC operon and extremely thermophilic bacteria. J Mol Evol.

[b23] Knowles TJ, Scott-Tucker A, Overduin M, Henderson IR (2009). Membrane protein architects: the role of the BAM complex in outer membrane protein assembly. Nat Rev Microbiol.

[b24] Krachler AM, Sharma A, Cauldwell A, Papadakos G, Kleanthous C (2010). TolA modulates the oligomeric status of YbgF in the bacterial periplasm. J Mol Biol.

[b26] Larkin MA, Blackshields G, Brown NP, Chenna R, McGettigan PA, McWilliam H (2007). Clustal W and Clustal X version 2.0. Bioinformatics.

[b27] Lewenza S, Mhlanga MM, Pugsley AP (2008). Novel inner membrane retention signals in *Pseudomonas aeruginosa* lipoproteins. J Bacteriol.

[b28] Likic VA, Dolezal P, Celik N, Dagley M, Lithgow T (2010). Using hidden Markov models to discover new protein transport machines. Methods Mol Biol.

[b29] Malinverni JC, Werner J, Kim S, Sklar JG, Kahne D, Misra R, Silhavy TJ (2006). YfiO stabilizes the YaeT complex and is essential for outer membrane protein assembly in *Escherichia coli*. Mol Microbiol.

[b30] Matroule JY, Lam H, Burnette DT, Jacobs-Wagner C (2004). Cytokinesis monitoring during development; rapid pole-to-pole shuttling of a signaling protein by localized kinase and phosphatase in *Caulobacter*. Cell.

[b31] Noinaj N, Fairman JW, Buchanan SK (2011). The crystal structure of BamB suggests interactions with BamA and its role within the BAM complex. J Mol Biol.

[b32] Olsen GJ, Woese CR, Overbeek R (1994). The winds of (evolutionary) change: breathing new life into microbiology. J Bacteriol.

[b33] Perrin E, Fondi M, Papaleo MC, Maida I, Buroni S, Pasca MR (2010). Exploring the HME and HAE1 efflux systems in the genus *Burkholderia*. BMC Evol Biol.

[b34] Poindexter JS (1964). Biological properties and classification of the *Caulobacter* group. Bacteriol Rev.

[b35] Purcell AW, Gorman JJ, Garcia-Peydró M, Paradela A, Burrows SR, Talbo GH (2001). Quantitative and qualitative influences of tapasin on the class I peptide repertoire. J Immunol.

[b36] Ricci DP, Silhavy TJ (2011). The BAM machine: a molecular cooper. Biochim Biophys Acta.

[b37] Rigel NW, Schwalm J, Ricci DP, Silhavy TJ (2012). BamE modulates the *Escherichia coli* beta-barrel assembly machine component BamA. J Bacteriol.

[b38] Ruiz N, Kahne D, Silhavy TJ (2006). Advances in understanding bacterial outer-membrane biogenesis. Nat Rev Microbiol.

[b39] Ryan KR, Taylor JA, Bowers LM (2010). The BAM complex subunit BamE (SmpA) is required for membrane integrity, stalk growth and normal levels of outer membrane {beta}-barrel proteins in *Caulobacter crescentus*. Microbiology.

[b40] Sandoval CM, Baker SL, Jansen K, Metzner SI, Sousa MC (2011). Crystal structure of BamD: an essential component of the β-Barrel assembly machinery of gram-negative bacteria. J Mol Biol.

[b41] Simon R, Prieffer U, Puhler A (1983). A broad host range mobilization system for *in vivo* genetic engineering: transposon mutagenesis in gram-negative bacteria. Biotechnology (N Y).

[b42] Spratt BG, Hedge PJ, te Heesen S, Edelman A, Broome-Smith JK (1986). Kanamycin-resistant vectors that are analogues of plasmids pUC8, pUC9, pEMBL8 and pEMBL9. Gene.

[b43] Sreerama N, Woody RW (2004). On the analysis of membrane protein circular dichroism spectra. Protein Sci.

[b45] Tellez R, Misra R (2012). Substitutions in the BamA β-barrel domain overcome the conditional lethal phenotype of a ΔbamB ΔbamE strain of *Escherichia coli*. J Bacteriol.

[b46] Thanbichler M, Iniesta AA, Shapiro L (2007). A comprehensive set of plasmids for vanillate- and xylose-inducible gene expression in *Caulobacter crescentus*. Nucleic Acids Res.

[b47] Tokuda H, Matsuyama S (2004). Sorting of lipoproteins to the outer membrane in *E. coli*. Biochim Biophys Acta.

[b48] Volokhina EB, Beckers F, Tommassen J, Bos MP (2009). The beta-barrel outer membrane protein assembly complex of *Neisseria meningitidis*. J Bacteriol.

[b49] Voulhoux R, Tommassen J (2004). Omp85, an evolutionarily conserved bacterial protein involved in outer-membrane-protein assembly. Res Microbiol.

[b50] Warner LR, Varga K, Lange OF, Baker SL, Baker D, Sousa MC, Pardi A (2011). Structure of the BamC two-domain protein obtained by Rosetta with a limited NMR data set. J Mol Biol.

[b51] Woese CR (1987). Bacterial evolution. Microbiol Rev.

