# Supplementary material for: The evolution of new lipoprotein subunits of the bacterial outer membrane BAM complex
Source: Mol Microbiol. 2012 Apr 23;84(5):832–44. doi: 10.1111/j.1365-2958.2012.08059.x (PMC3359395; doi:10.1111/j.1365-2958.2012.08059.x)
Supplement: Supplementary file 1 [file mmi0084-0832-SD1.pdf]

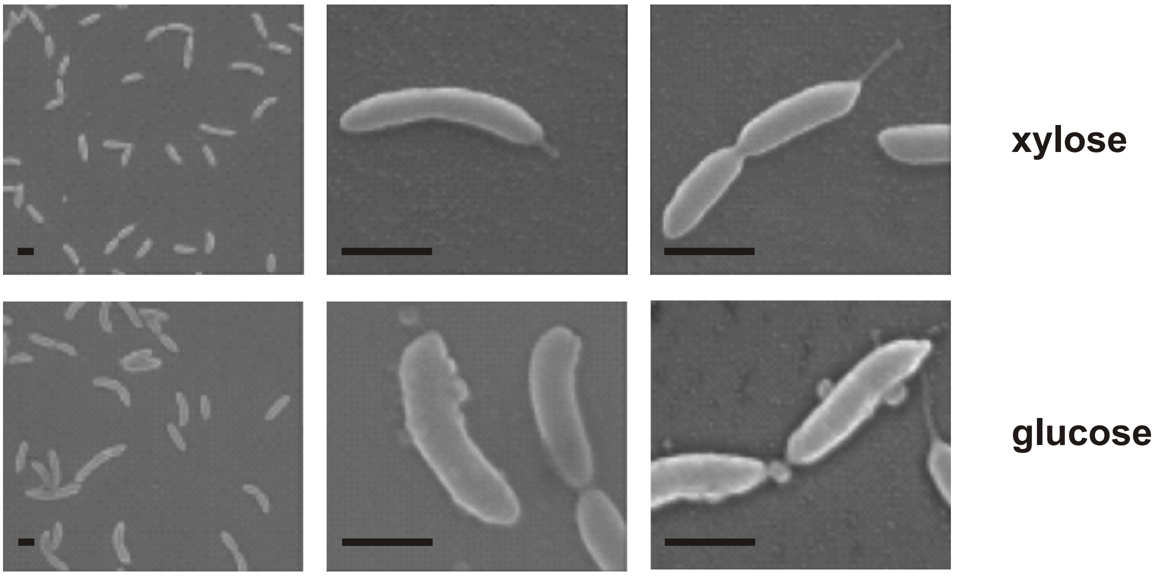

Figure S1: Scanning electron microscopy of *Caulobacter crescentus* after shut-down of BamD expression. To assess the phenotype of *C. crescentus* after depletion of BamD, we constructed a strain with BamD under the control of a xylose-inducible promoter (see Methods). The strain is viable on medium containing xylose, but inviable when xylose is absent from the growth medium, demonstrating that the *bamD* gene is essential in *C. crescentus*. Scanning electron microscopy was used to monitor the depletion of BamD after shifting the strain from growth medium containing 0.03% (w/v) xylose to medium containing 0.2% (w/v) glucose: depletion of BamD resulted in drastic changes to the surface of *C. crescentus*. Scale bars represent 1 μm.

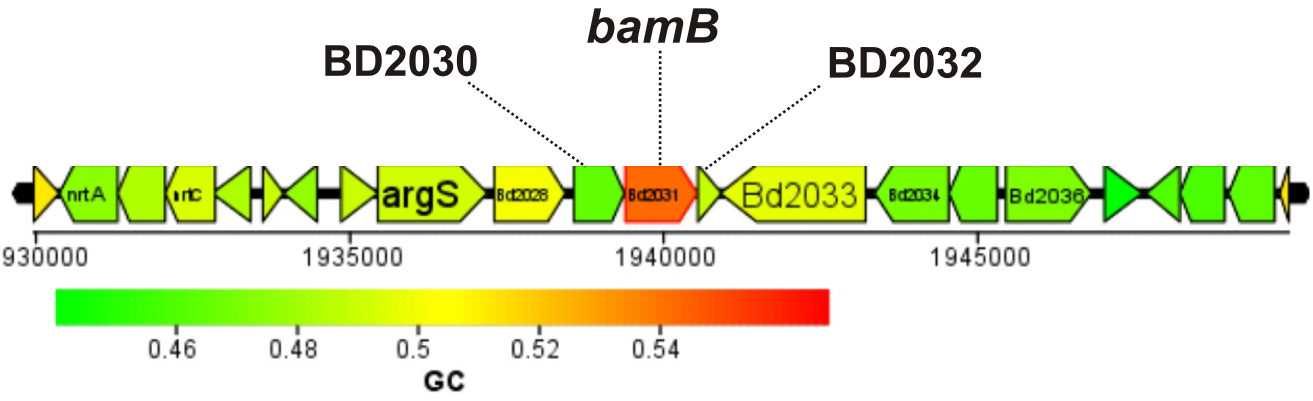

Figure S2: Gene context of the predicted *bamB* gene in the genome of *Bdellovibrio bacteriovorus* HD100. The GC content of the genome of str. Hd100 was mapped across the chromosomal locus encompassing the gene BD2031 (*bamB*) using XBASE (Chaudhuri, R.R., Loman, N.J., Snyder, L.A., Bailey, C.M., Stekel, D.J., Pallen M.J. (2008) *Nucleic Acids Res*. 36(Database issue):D543-546). The upstream gene BD2030 encodes a protein of unknown function conserved only in other δ-proteobacteria. Together with characterized genes such as *argS*, it defines this chromosomal locus. The down-stream sequence BD2032 is not conserved in other δ-proteobacteria, but is highly similar (40-50% sequence identity) to sequences in the genomes of various bacteria (closest relatives *Neisseria weaverii* and *Kingella oralis*), and the Conserved Domain Architecture Tool suggests sequence similarity with L-413C-like integrases. Evidence that the BD2031(*bamB*) gene is functional in *B. bacteriovorus* comes from an observed 6-fold induction of gene expression on the switch from (non replicating) predatory hunter form to axenic grower on peptone rich media (Lambert, C., Chang, C.Y., Capeness, M.J., Sockett, R.E. (2010) *PLoS One* 5:e8599).

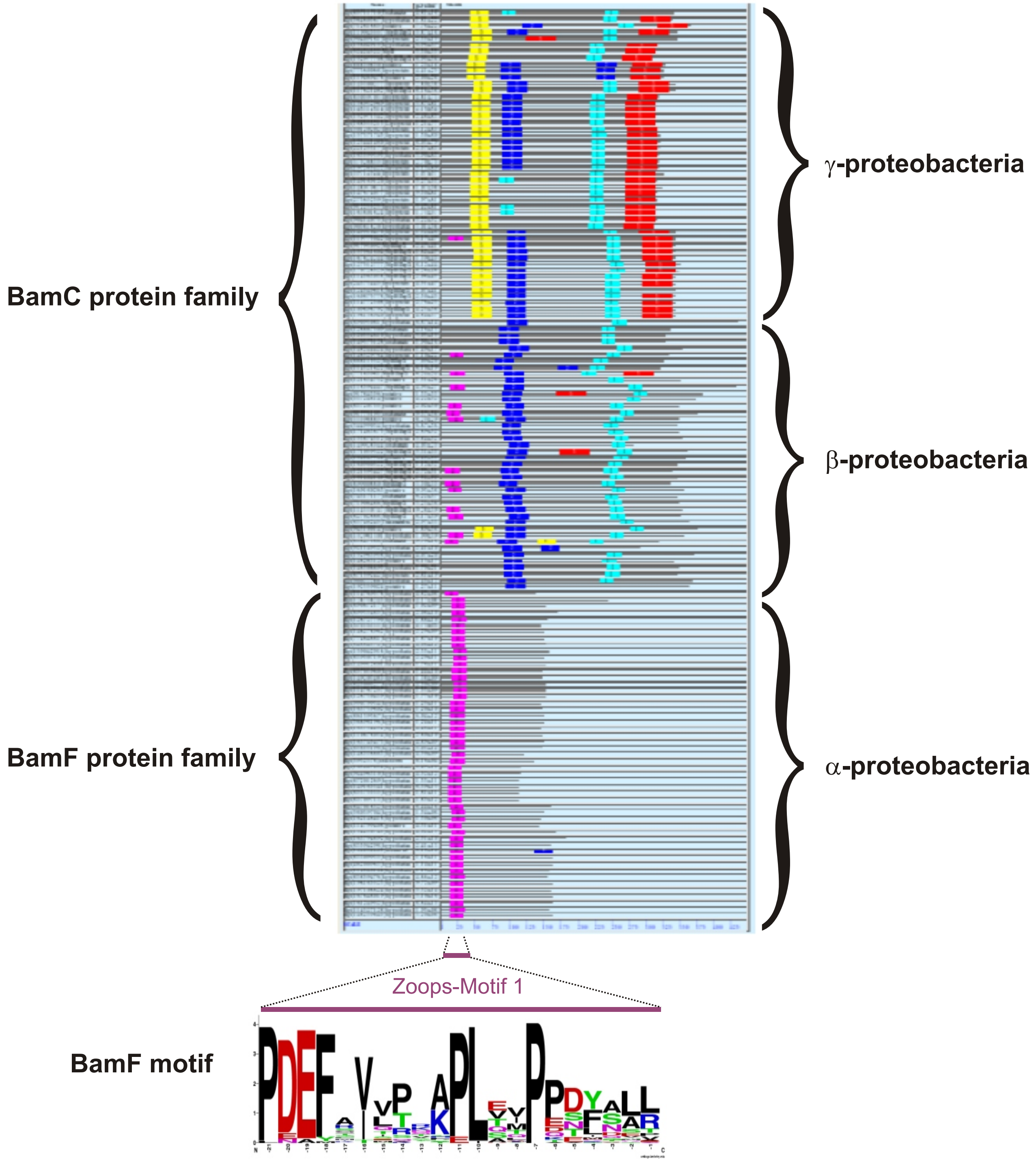

Figure S3. Motif analysis of the BamC-family of proteins and the BamF-family of proteins. Each of the protein sequences analysed is represented as a single line, and these have been sorted into species of γ-proteobacteria, β-proteobacteria and α-proteobacteria. The common motifs are shown as colored boxes on each protein sequence: "Zoops motif 1" (colored in pink) corresponds to the sequence logo shown as the BamF motif. It is found in all α-proteobacterial BamF proteins and also in many β-proteobacterial BamC proteins. Although the motif is not strictly conserved in γ-proteobacteria, the corresponding region in all BamC sequences shows some sequence similarity (documented in Figure 4). There are three strongly-conserved motifs within the structured domains of γ-proteobacterial and β-proteobacterial BamC sequences (the motifs are shown in blue, cyan and red) that define elements of the 'helix-grip' fold. In addition, a short conserved motif ($E=2.5x10^{-156}$; colored in yellow) was found in the N-terminal 'unstructured' region: it sits between the helix-grip domains and the region of sequence conserved across the BamF- and BamC-protein families. In the *E. coli* BamC sequence, this "motif 2" appears

CDPro Algorithm	α - helix	β - sheet	turns	unordered
CONTIN	65	4	12	19
SELCON3	67	4	11	18
CDSSTR	82	5	6	7

Table S1. Calculation of secondary structure components (%) from the CD spectra of BamD. Calculations were performed using the CDPro software package (Sreerama N & Woody RW, 2004).

Supplementary Table 2

Acetobacter pasteurianus IFO 3283-01	5.10E-021	BamB	YP_003188239.1	dehydrogenase
Acetobacter pasteurianus IFO 3283-01	2.50E-007	BamB	YP_003188704.1	alcohol dehydrogenase large subunit
Acetobacter pasteurianus IFO 3283-01	7.00E-007	BamB	YP_003186624.1	alcohol dehydrogenase large subunit
Acetobacter pasteurianus IFO 3283-01	8.70E-005	BamB	YP_003188649.1	glucose dehydrogenase, methanol dehydrogenase subunit 1
Acetobacter pasteurianus IFO 3283-01	1.00E-019	BamD	YP_003188411.1	hypothetical protein APA01_19070
Acetobacter pasteurianus IFO 3283-01	9.30E-013	BamE	YP_003188212.1	lipoprotein SmpA/OmlA
Acetobacter pasteurianus IFO 3283-01	9.10E-005	BamE	YP_003188097.1	hypothetical protein APA01_15790
Acetobacter pasteurianus IFO 3283-01	2.40E-111	Omp85	YP_003187185.1	outer membrane protein
Acetobacter pasteurianus IFO 3283-01	2.90E-006	Omp85	YP_003187610.1	outer membrane protein
Acidiphilium cryptum JF-5	7.20E-022	BamB	YP_001233890.1	Pyrrolo-quinoline quinone
Acidiphilium cryptum JF-5	1.40E-008	BamB	YP_001234864.1	Pyrrolo-quinoline quinone
Acidiphilium cryptum JF-5	6.50E-007	BamB	YP_001234695.1	Pyrrolo-quinoline quinone
Acidiphilium cryptum JF-5	1.70E-006	BamB	YP_001235907.1	Pyrrolo-quinoline quinone
Acidiphilium cryptum JF-5	2.30E-005	BamB	YP_001235900.1	Pyrrolo-quinoline quinone
Acidiphilium cryptum JF-5	4.20E-019	BamD	YP_001233894.1	DNA uptake lipoprotein-like protein
Acidiphilium cryptum JF-5	3.30E-009	BamE	YP_001233419.1	SmpA/OmlA domain-containing protein
Acidiphilium cryptum JF-5	6.60E-006	BamE	YP_001234501.1	hypothetical protein Acry_1371
Acidiphilium cryptum JF-5	6.40E-005	BamE	YP_001235487.1	hypothetical protein Acry_2374
Acidiphilium cryptum JF-5	2.10E-115	Omp85	YP_001235556.1	surface antigen (D15)
Acidiphilium cryptum JF-5	5.80E-007	Omp85	YP_001233312.1	surface antigen (D15)
Acidiphilium multivorum AIU301	7.20E-022	BamB	YP_004283350.1	pyrrolo-quinoline quinone
Acidiphilium multivorum AIU301	1.50E-008	BamB	YP_004284015.1	putative quinoprotein ethanol dehydrogenase
Acidiphilium multivorum AIU301	6.50E-007	BamB	YP_004283842.1	putative pyrrolo-quinoline quinone
Acidiphilium multivorum AIU301	1.60E-006	BamB	YP_004285358.1	alcohol dehydrogenase
Acidiphilium multivorum AIU301	2.30E-005	BamB	YP_004285351.1	PQQ-dependent dehydrogenase
Acidiphilium multivorum AIU301	4.20E-019	BamD	YP_004283346.1	putative lipoprotein
Acidiphilium multivorum AIU301	3.30E-009	BamE	YP_004282529.1	hypothetical protein ACMV_03000
Acidiphilium multivorum AIU301	5.00E-006	BamE	YP_004283647.1	hypothetical protein ACMV_14180
Acidiphilium multivorum AIU301	6.40E-005	BamE	YP_004284929.1	hypothetical protein ACMV_27000
Acidiphilium multivorum AIU301	3.80E-112	Omp85	YP_004284991.1	hypothetical protein ACMV_27620
Acidiphilium multivorum AIU301	6.30E-007	Omp85	YP_004282419.1	putative outer membrane protein
Agrobacterium radiobacter K84	4.10E-006	BamB	YP_002541776.1	glucose dehydrogenase (pyrroloquinoline-quinone) protein
Agrobacterium radiobacter K84	9.30E-006	BamB	YP_002540870.1	Protein kinase: pyrrolo-quinoline quinone
Agrobacterium radiobacter K84	6.70E-005	BamB	YP_002540305.1	glucose dehydrogenase (pyrroloquinoline-quinone) protein
Agrobacterium radiobacter K84	7.20E-020	BamD	YP_002544968.1	hypothetical protein Arad_2982
Agrobacterium radiobacter K84	2.00E-030	BamE	YP_002544048.1	hypothetical protein Arad_1765
Agrobacterium radiobacter K84	4.00E-005	BamE	YP_002543299.1	glycine betaine ABC transporter

Agrobacterium radiobacter K84	1.30E-107	Omp85	YP_002544395.1	outer membrane lipoprotein
Agrobacterium radiobacter K84	8.60E-007	Omp85	YP_002545946.1	outer membrane protein
Agrobacterium sp. H13-3	2.30E-005	BamB	YP_004444323.1	Glucose dehydrogenase
Agrobacterium sp. H13-3	4.40E-021	BamD	YP_004279319.1	lipoprotein
Agrobacterium sp. H13-3	3.30E-030	BamE	YP_004278413.1	hypothetical protein AGROH133_05409
Agrobacterium sp. H13-3	2.70E-117	Omp85	YP_004278608.1	group 1 outer membrane protein precursor
Agrobacterium sp. H13-3	8.00E-007	Omp85	YP_004279849.1	Outer membrane protein
Agrobacterium tumefaciens str. C58	2.20E-005	BamB	NP_356512.1	glucose dehydrogenase
Agrobacterium tumefaciens str. C58	1.60E-020	BamD	NP_355049.2	hypothetical protein Atu2084
Agrobacterium tumefaciens str. C58	1.40E-030	BamE	NP_354193.2	hypothetical protein Atu1175
Agrobacterium tumefaciens str. C58	8.60E-117	Omp85	NP_354388.1	hypothetical protein Atu1381
Agrobacterium tumefaciens str. C58	7.20E-007	Omp85	NP_355552.1	hypothetical protein Atu2615
Agrobacterium vitis S4	9.40E-006	BamB	YP_002550056.1	glucose dehydrogenase
Agrobacterium vitis S4	1.40E-022	BamD	YP_002550084.1	hypothetical protein Avi_2880
Agrobacterium vitis S4	1.00E-031	BamE	YP_002549169.1	hypothetical protein Avi_1624
Agrobacterium vitis S4	3.00E-124	Omp85	YP_002549812.1	group 1 outer membrane protein precursor
Agrobacterium vitis S4	7.10E-007	Omp85	YP_002550968.1	hypothetical protein Avi_4088
Anaplasma centrale str. Israel	3.90E-010	BamB	YP_003328463.1	hypothetical protein ACIS_00541
Anaplasma centrale str. Israel	5.70E-016	BamD	YP_003328305.1	DNA uptake lipoprotein ComL
Anaplasma centrale str. Israel	3.10E-006	BamE	YP_003328540.1	hypothetical protein ACIS_00646
Anaplasma centrale str. Israel	1.20E-054	Omp85	YP_003328252.1	hypothetical protein ACIS_00268
Anaplasma marginale str. Florida	1.90E-010	BamB	YP_002563693.1	hypothetical protein AMF_592
Anaplasma marginale str. Florida	2.30E-015	BamD	YP_002563857.1	hypothetical protein AMF_769
Anaplasma marginale str. Florida	2.80E-006	BamE	YP_002563605.1	hypothetical protein AMF_493
Anaplasma marginale str. Florida	2.90E-060	Omp85	YP_002563915.1	outer membrane protein
Anaplasma marginale str. St. Maries	1.90E-010	BamB	YP_153984.1	hypothetical protein AM798
Anaplasma marginale str. St. Maries	2.30E-015	BamD	YP_154148.1	hypothetical protein AM1010
Anaplasma marginale str. St. Maries	9.00E-060	Omp85	YP_154207.1	outer membrane protein
Anaplasma phagocytophilum HZ	1.20E-011	BamB	YP_504997.1	PQQ repeat-containing protein
Anaplasma phagocytophilum HZ	1.00E-014	BamD	YP_505635.1	putative competence lipoprotein ComL
Anaplasma phagocytophilum HZ	8.00E-055	Omp85	YP_505741.1	OMP85 family outer membrane protein
Asticcacaulis excentricus CB 48	1.60E-117	BamB	YP_004088487.1	pyrrolo-quinoline quinone beta-propeller repeat
Asticcacaulis excentricus CB 48	8.60E-020	BamD	YP_004087487.1	outer membrane assembly lipoprotein yfio
Asticcacaulis excentricus CB 48	2.00E-018	BamE	YP_004088721.1	smpa/omla domain-containing protein
Asticcacaulis excentricus CB 48	2.70E-007	Omp85	YP_004087507.1	surface antigen (d15)
Asticcacaulis excentricus CB 48	7.80E-124	Omp85	YP_004088640.1	outer membrane protein assembly complex, yaet protein
Azorhizobium caulinodans ORS 571	6.70E-018	BamD	YP_001527483.1	lipoprotein precursor
Azorhizobium caulinodans ORS 571	1.30E-026	BamE	YP_001525214.1	SmpA protein
Azorhizobium caulinodans ORS 571	1.00E-143	Omp85	YP_001524617.1	outer membrane protein precursor
Azorhizobium caulinodans ORS 571	5.80E-006	Omp85	YP_001523673.1	hypothetical protein AZC_0757

Azospirillum sp. B510	2.20E-080	BamB	YP_003448892.1	pyrrolo-quinoline quinone
Azospirillum sp. B510	1.10E-007	BamB	YP_003450601.1	alcohol dehydrogenase (acceptor)
Azospirillum sp. B510	7.60E-008	BamB	YP_003451663.1	alcohol dehydrogenase (acceptor)
Azospirillum sp. B510	2.00E-006	BamB	YP_003453045.1	quinoprotein glucose dehydrogenase
Azospirillum sp. B510	6.40E-033	BamD	YP_003449141.1	lipoprotein
Azospirillum sp. B510	5.50E-038	BamE	YP_003448201.1	SmpA/OmlA domain-containing protein
Azospirillum sp. B510	5.40E-147	Omp85	YP_003448800.1	outer membrane protein
Azospirillum sp. B510	1.10E-007	Omp85	YP_003448434.1	surface antigen
Bartonella bacilliformis KC583	2.00E-017	BamD	YP_989220.1	putative lipoprotein
Bartonella bacilliformis KC583	3.50E-017	BamE	YP_988939.1	SmpA/OmlA family outer membrane protein
Bartonella bacilliformis KC583	5.40E-107	Omp85	YP_988897.1	OMP85 family outer membrane protein
Bartonella bacilliformis KC583	4.00E-006	Omp85	YP_989591.1	OMP85 family outer membrane protein
Bartonella clarridgeiae 73	4.10E-018	BamD	YP_004159242.1	competence lipoprotein precursor
Bartonella clarridgeiae 73	9.00E-028	BamE	YP_004159081.1	Small protein A
Bartonella clarridgeiae 73	1.20E-107	Omp85	YP_004159130.1	outer membrane protein
Bartonella clarridgeiae 73	2.10E-005	Omp85	YP_004158328.1	hypothetical protein BARCL_0049
Bartonella grahamii as4aup	6.90E-019	BamD	YP_002972284.1	competence lipoprotein ComL precursor
Bartonella grahamii as4aup	1.50E-032	BamE	YP_002972086.1	small protein A, outer membrane lipoprotein
Bartonella grahamii as4aup	8.00E-104	Omp85	YP_002971703.1	outer membrane protein
Bartonella grahamii as4aup	8.00E-006	Omp85	YP_002971110.1	putative outer membrane protein
Bartonella henselae str. Houston-1	1.10E-017	BamD	YP_033888.1	competence lipoprotein comL precursor
Bartonella henselae str. Houston-1	5.10E-033	BamE	YP_033572.1	hypothetical protein BH07600
Bartonella henselae str. Houston-1	5.40E-113	Omp85	YP_033457.1	Outer membrane protein
Bartonella henselae str. Houston-1	4.40E-006	Omp85	YP_032911.1	hypothetical protein BH00450
Bartonella quintana str. Toulouse	1.80E-018	BamD	YP_032490.1	competence lipoprotein comL precursor
Bartonella quintana str. Toulouse	5.70E-031	BamE	YP_032207.1	hypothetical protein BQ05450
Bartonella quintana str. Toulouse	1.40E-105	Omp85	YP_032332.1	Outer membrane protein
Bartonella quintana str. Toulouse	2.90E-006	Omp85	YP_031766.1	hypothetical protein BQ00400
Bartonella tribocorum CIP 105476	2.00E-018	BamD	YP_001609917.1	competence lipoprotein precursor
Bartonella tribocorum CIP 105476	4.60E-031	BamE	YP_001609671.1	small protein A
Bartonella tribocorum CIP 105476	2.00E-103	Omp85	YP_001609311.1	outer membrane protein
Bartonella tribocorum CIP 105476	5.70E-006	Omp85	YP_001608542.1	putative outer membrane protein
Beijerinckia indica subsp. indica ATCC 9039	6.40E-007	BamB	YP_001832332.1	Pyrrolo-quinoline quinone
Beijerinckia indica subsp. indica ATCC 9039	9.50E-007	BamB	YP_001832102.1	Pyrrolo-quinoline quinone
Beijerinckia indica subsp. indica ATCC 9039	2.20E-006	BamB	YP_001834680.1	methanol/ethanol family PQQ-dependent dehydrogenase
Beijerinckia indica subsp. indica ATCC 9039	2.00E-020	BamD	YP_001831838.1	DNA uptake lipoprotein-like protein
Beijerinckia indica subsp. indica ATCC 9039	2.50E-018	BamE	YP_001834429.1	SmpA/OmlA domain-containing protein
Beijerinckia indica subsp. indica ATCC 9039	7.50E-133	Omp85	YP_001831438.1	outer membrane protein assembly complex, YaeT protein
Beijerinckia indica subsp. indica ATCC 9039	4.10E-006	Omp85	YP_001832835.1	surface antigen (D15)
Bradyrhizobium japonicum USDA 110	8.80E-008	BamB	NP_772860.1	alcohol dehydrogenase

alpha_e-5

Bradyrhizobium japonicum USDA 110	1.10E-007	BamB	NP_766973.1	alcohol dehydrogenase
Bradyrhizobium japonicum USDA 110	1.10E-007	BamB	NP_772847.1	quinoprotein ethanol dehydrogenase
Bradyrhizobium japonicum USDA 110	1.60E-007	BamB	NP_774279.1	alcohol dehydrogenase
Bradyrhizobium japonicum USDA 110	2.70E-006	BamB	NP_772144.1	polyvinyl-alcohol dehydrogenase
Bradyrhizobium japonicum USDA 110	5.10E-005	BamB	NP_772853.1	methanol dehydrogenase large subunit-like protein
Bradyrhizobium japonicum USDA 110	2.00E-017	BamD	NP_773234.1	hypothetical protein bll6594
Bradyrhizobium japonicum USDA 110	2.00E-023	BamE	NP_771664.1	hypothetical protein blr5024
Bradyrhizobium japonicum USDA 110	9.40E-113	Omp85	NP_770352.1	outer membrane protein
Bradyrhizobium japonicum USDA 110	4.30E-104	Omp85	NP_771493.1	outer membrane protein
Bradyrhizobium sp. BTAi1	1.60E-007	BamB	YP_001238404.1	putative quinoprotein
Bradyrhizobium sp. BTAi1	1.90E-007	BamB	YP_001241809.1	putative quinoprotein ethanol dehydrogenase
Bradyrhizobium sp. BTAi1	4.90E-007	BamB	YP_001241369.1	putative alcohol dehydrogenase
Bradyrhizobium sp. BTAi1	1.30E-006	BamB	YP_001239913.1	putative alcohol dehydrogenase
Bradyrhizobium sp. BTAi1	1.60E-006	BamB	YP_001238114.1	putative quinoprotein ethanol dehydrogenase
Bradyrhizobium sp. BTAi1	5.20E-006	BamB	YP_001241813.1	methanol dehydrogenase large subunit
Bradyrhizobium sp. BTAi1	2.30E-005	BamB	YP_001243334.1	quinoprotein glucose dehydrogenase
Bradyrhizobium sp. BTAi1	3.70E-019	BamD	YP_001241994.1	hypothetical protein BBta_6164
Bradyrhizobium sp. BTAi1	5.10E-020	BamE	YP_001240569.1	small protein A domain-containing protein
Bradyrhizobium sp. BTAi1	4.80E-106	Omp85	YP_001240447.1	surface antigen domain-containing protein
Bradyrhizobium sp. BTAi1	2.00E-103	Omp85	YP_001236335.1	hypothetical protein BBta_0131
Bradyrhizobium sp. BTAi1	2.70E-087	Omp85	YP_001241677.1	putative protective surface antigen
Bradyrhizobium sp. ORS 278	1.20E-007	BamB	YP_001202344.1	quinoprotein ethanol dehydrogenase
Bradyrhizobium sp. ORS 278	1.20E-007	BamB	YP_001207373.1	quinoprotein ethanol dehydrogenase
Bradyrhizobium sp. ORS 278	1.60E-007	BamB	YP_001203590.1	quinoprotein ethanol dehydrogenase
Bradyrhizobium sp. ORS 278	2.20E-006	BamB	YP_001203821.1	quinoprotein ethanol dehydrogenase
Bradyrhizobium sp. ORS 278	3.40E-006	BamB	YP_001207377.1	methanol dehydrogenase large subunit-like protein
Bradyrhizobium sp. ORS 278	4.10E-006	BamB	YP_001205882.1	alcohol dehydrogenase (acceptor)
Bradyrhizobium sp. ORS 278	6.20E-005	BamB	YP_001202777.1	quinoprotein glucose dehydrogenase
Bradyrhizobium sp. ORS 278	3.10E-019	BamD	YP_001207538.1	TPR repeat-containing protein
Bradyrhizobium sp. ORS 278	1.50E-020	BamE	YP_001206385.1	hypothetical protein BRADO4421
Bradyrhizobium sp. ORS 278	2.50E-104	Omp85	YP_001206109.1	hypothetical protein BRADO4132
Bradyrhizobium sp. ORS 278	2.60E-100	Omp85	YP_001202441.1	outer membrane protein
Bradyrhizobium sp. ORS 278	1.80E-081	Omp85	YP_001207233.1	protective surface antigen
Brevundimonas subvibrioides ATCC 15264	1.30E-106	BamB	YP_003819467.1	pyrrolo-quinoline quinone beta-propeller repeat protein
Brevundimonas subvibrioides ATCC 15264	2.60E-019	BamD	YP_003819592.1	outer membrane assembly lipoprotein YfiO
Brevundimonas subvibrioides ATCC 15264	2.10E-030	BamE	YP_003818827.1	SmpA/OmlA domain-containing protein
Brevundimonas subvibrioides ATCC 15264	2.10E-136	Omp85	YP_003819423.1	outer membrane protein assembly complex, YaeT protein
Brevundimonas subvibrioides ATCC 15264	3.70E-005	Omp85	YP_003818283.1	surface antigen (D15)
Brucella abortus bv. 1 str. 9-941	2.60E-025	BamD	YP_222108.1	competence protein ComL
Brucella abortus bv. 1 str. 9-941	3.70E-059	BamE	YP_221517.1	hypothetical protein BruAb1_0788

Brucella abortus bv. 1 str. 9-941	2.60E-118	Omp85	YP_221860.1	surface antigen
Brucella abortus bv. 1 str. 9-941	4.90E-006	Omp85	YP_220827.1	hypothetical protein BruAb1_0048
Brucella abortus S19	2.60E-025	BamD	YP_001935317.1	TPR repeat-containing protein
Brucella abortus S19	3.70E-059	BamE	YP_001934741.1	SmpA/OmlA
Brucella abortus S19	2.60E-118	Omp85	YP_001935075.1	surface antigen
Brucella abortus S19	4.40E-006	Omp85	YP_001934077.1	surface antigen
Brucella canis ATCC 23365	2.60E-025	BamD	YP_001593257.1	hypothetical protein BCAN_A1455
Brucella canis ATCC 23365	1.20E-060	BamE	YP_001592626.1	SmpA/OmlA domain-containing protein
Brucella canis ATCC 23365	2.30E-118	Omp85	YP_001592993.1	Outer membrane protein assembly factor yaeT precursor
Brucella canis ATCC 23365	4.80E-006	Omp85	YP_001591924.1	surface antigen (D15)
Brucella melitensis ATCC 23457	2.60E-025	BamD	YP_002733137.1	outer membrane assembly lipoprotein YfiO
Brucella melitensis ATCC 23457	1.20E-060	BamE	YP_002732521.1	SmpA/OmlA domain-containing protein
Brucella melitensis ATCC 23457	8.70E-118	Omp85	YP_002732885.1	outer membrane protein assembly complex protein YaeT
Brucella melitensis ATCC 23457	4.90E-006	Omp85	YP_002731823.1	surface antigen D15
Brucella melitensis biovar Abortus 2308	2.60E-025	BamD	YP_414813.1	TPR repeat-containing protein
Brucella melitensis biovar Abortus 2308	3.70E-059	BamE	YP_414227.1	SmpA/OmlA
Brucella melitensis biovar Abortus 2308	2.60E-118	Omp85	YP_414567.1	surface antigen
Brucella melitensis biovar Abortus 2308	4.90E-006	Omp85	YP_413545.1	surface antigen
Brucella melitensis bv. 1 str. 16M	2.60E-025	BamD	NP_539504.1	COML, competence lipoprotein
Brucella melitensis bv. 1 str. 16M	1.20E-060	BamE	NP_540101.1	small protein A
Brucella melitensis bv. 1 str. 16M	8.70E-118	Omp85	NP_539747.1	outer membrane protein
Brucella melitensis bv. 1 str. 16M	1.20E-005	Omp85	NP_540812.1	outer membrane protein
Brucella microti CCM 4915	2.60E-025	BamD	YP_003107349.1	competence protein ComL
Brucella microti CCM 4915	1.20E-060	BamE	YP_003106712.1	SmpA/OmlA domain-containing protein
Brucella microti CCM 4915	2.30E-118	Omp85	YP_003107089.1	surface antigen
Brucella microti CCM 4915	5.00E-006	Omp85	YP_003106020.1	surface antigen
Brucella ovis ATCC 25840	1.70E-025	BamD	YP_001259314.1	putative competence protein ComL
Brucella ovis ATCC 25840	1.30E-060	BamE	YP_001258751.1	hypothetical protein BOV_0765
Brucella ovis ATCC 25840	3.30E-123	Omp85	YP_001259073.1	outer membrane protein assembly complex protein YaeT
Brucella ovis ATCC 25840	4.20E-006	Omp85	YP_001258093.1	hypothetical protein BOV_0047
Brucella suis 1330	2.60E-025	BamD	NP_698420.1	competence protein ComL
Brucella suis 1330	1.20E-060	BamE	NP_697786.1	hypothetical protein BR0772
Brucella suis 1330	9.10E-118	Omp85	NP_698159.1	surface antigen
Brucella suis 1330	4.90E-006	Omp85	NP_697090.1	hypothetical protein BR0048
Brucella suis ATCC 23445	2.60E-025	BamD	YP_001628081.1	hypothetical protein BSUIS_A1473
Brucella suis ATCC 23445	1.20E-060	BamE	YP_001627447.1	SmpA/OmlA domain-containing protein
Brucella suis ATCC 23445	7.30E-118	Omp85	YP_001627823.1	Outer membrane protein assembly factor yaeT precursor
Brucella suis ATCC 23445	5.60E-006	Omp85	YP_001626728.1	surface antigen (D15)
Candidatus Liberibacter asiaticus str. psy62	1.90E-015	BamD	YP_003065587.1	outer membrane assembly lipoprotein YfiO
Candidatus Liberibacter asiaticus str. psy62	3.20E-007	BamE	YP_003064913.1	hypothetical protein CLIBASIA_01935

Candidatus Liberibacter asiaticus str. psy62	2.50E-062	Omp85	YP_003065185.1	surface antigen (D15)
Candidatus Liberibacter solanacearum CLso-ZC1	4.10E-017	BamD	YP_004063058.1	outer membrane assembly lipoprotein YfiO
Candidatus Liberibacter solanacearum CLso-ZC1	1.00E-008	BamE	YP_004062802.1	hypothetical protein CKC_02825
Candidatus Liberibacter solanacearum CLso-ZC1	5.90E-021	Omp85	YP_004062478.1	surface antigen (D15)
Candidatus Midichloria mitochondrii IricVA	6.20E-013	BamB	YP_004680139.1	PQQ enzyme repeat family protein
Candidatus Midichloria mitochondrii IricVA	1.60E-018	BamD	YP_004679950.1	competence lipoprotein ComL
Candidatus Midichloria mitochondrii IricVA	1.20E-008	BamE	YP_004679823.1	hypothetical protein midi_00849
Candidatus Midichloria mitochondrii IricVA	1.50E-010	Omp85	YP_004679182.1	surface antigen
Candidatus Pelagibacter sp. IMCC9063	1.60E-007	BamB	YP_004358135.1	PQQ enzyme repeat family protein
Candidatus Pelagibacter sp. IMCC9063	8.90E-015	BamD	YP_004357981.1	putative competence lipoprotein ComL
Candidatus Pelagibacter sp. IMCC9063	7.60E-011	Omp85	YP_004358253.1	outer membrane protein assembly factor YaeT precursor
Candidatus Pelagibacter ubique HTCC1062	4.00E-009	BamB	YP_266730.1	PQQ repeat-containing protein
Candidatus Pelagibacter ubique HTCC1062	1.10E-014	BamD	YP_265445.1	competence lipoprotein ComL
Candidatus Pelagibacter ubique HTCC1062	3.50E-009	BamE	YP_266449.1	hypothetical protein SAR11_1039
Candidatus Pelagibacter ubique HTCC1062	4.80E-011	Omp85	YP_266329.1	outer membrane protein omp1
Candidatus Puniceispirillum marinum IMCC1322	3.00E-033	BamB	YP_003552827.1	pyrrolo-quinoline quinone
Candidatus Puniceispirillum marinum IMCC1322	8.70E-027	BamD	YP_003552653.1	competence lipoprotein ComL
Candidatus Puniceispirillum marinum IMCC1322	1.70E-009	BamE	YP_003550495.1	SmpA/OmlA
Candidatus Puniceispirillum marinum IMCC1322	4.20E-108	Omp85	YP_003550838.1	surface antigen D15
Caulobacter crescentus CB15	3.20E-291	BamB	NP_420463.1	PQQ repeat-containing protein
Caulobacter crescentus CB15	2.80E-020	BamD	NP_420791.1	competence lipoprotein ComL
Caulobacter crescentus CB15	1.20E-052	BamE	NP_420178.1	hypothetical protein CC_1365
Caulobacter crescentus CB15	2.80E-138	Omp85	NP_420722.1	outer membrane protein
Caulobacter crescentus CB15	9.60E-006	Omp85	NP_420414.1	hypothetical protein CC_1603
Caulobacter crescentus NA1000	3.20E-291	BamB	YP_002517098.1	PQQ enzyme repeat family protein
Caulobacter crescentus NA1000	2.80E-020	BamD	YP_002517436.1	ComL family lipoprotein
Caulobacter crescentus NA1000	3.80E-052	BamE	YP_002516800.1	SmpA/OmlA family lipoprotein
Caulobacter crescentus NA1000	9.10E-144	Omp85	YP_002517365.1	outer membrane protein assembly factor
Caulobacter crescentus NA1000	1.70E-005	Omp85	YP_002517048.1	outer membrane protein
Caulobacter segnis ATCC 21756	1.80E-251	BamB	YP_003593401.1	PQQ enzyme repeat family protein
Caulobacter segnis ATCC 21756	1.50E-022	BamD	YP_003592551.1	outer membrane assembly lipoprotein YfiO
Caulobacter segnis ATCC 21756	1.70E-047	BamE	YP_003592768.1	SmpA/OmlA domain-containing protein
Caulobacter segnis ATCC 21756	1.90E-144	Omp85	YP_003593283.1	outer membrane protein assembly complex YaeT protein
Caulobacter segnis ATCC 21756	2.10E-005	Omp85	YP_003593674.1	surface antigen (D15)
Caulobacter sp. K31	4.90E-211	BamB	YP_001684122.1	Pyrrolo-quinoline quinone
Caulobacter sp. K31	2.50E-019	BamD	YP_001684725.1	outer membrane assembly lipoprotein YfiO
Caulobacter sp. K31	8.80E-040	BamE	YP_001684662.1	SmpA/OmlA domain-containing protein
Caulobacter sp. K31	6.10E-132	Omp85	YP_001684422.1	outer membrane protein assembly complex, YaeT protein
Caulobacter sp. K31	6.50E-006	Omp85	YP_001683825.1	surface antigen (D15)
Dinoroseobacter shibae DFL 12	4.30E-016	BamB	YP_001532508.1	pyrrolo-quinoline quinone

Dinoroseobacter shibae DFL 12	2.50E-008	BamB	YP_001534007.1	quinoprotein ethanol dehydrogenase
Dinoroseobacter shibae DFL 12	4.90E-005	BamB	YP_001531825.1	quinoprotein glucose dehydrogenase
Dinoroseobacter shibae DFL 12	1.60E-019	BamD	YP_001533749.1	hypothetical protein Dshi_2414
Dinoroseobacter shibae DFL 12	3.80E-010	BamE	YP_001533059.1	hypothetical protein Dshi_1716
Dinoroseobacter shibae DFL 12	3.90E-148	Omp85	YP_001532842.1	hypothetical protein Dshi_1499
Ehrlichia canis str. Jake	7.00E-012	BamB	YP_303156.1	quino protein
Ehrlichia canis str. Jake	9.80E-019	BamD	YP_303441.1	hypothetical protein Ecaj_0812
Ehrlichia canis str. Jake	9.30E-009	BamE	YP_303055.1	hypothetical protein Ecaj_0414
Ehrlichia canis str. Jake	3.80E-049	Omp85	YP_303492.1	surface antigen (D15):surface antigen variable number
Ehrlichia chaffeensis str. Arkansas	1.30E-010	BamB	YP_507323.1	PQQ repeat-containing protein
Ehrlichia chaffeensis str. Arkansas	3.70E-018	BamD	YP_507792.1	putative competence protein ComL
Ehrlichia chaffeensis str. Arkansas	2.40E-008	BamE	YP_507435.1	putative lipoprotein
Ehrlichia chaffeensis str. Arkansas	4.50E-056	Omp85	YP_507856.1	OMP85 family outer membrane protein
Ehrlichia ruminantium str. Gardel	2.00E-011	BamB	YP_196455.1	hypothetical protein ERGA_CDS_05290
Ehrlichia ruminantium str. Gardel	4.80E-017	BamD	YP_196736.1	hypothetical protein ERGA_CDS_08100
Ehrlichia ruminantium str. Gardel	9.30E-010	BamE	YP_196360.1	hypothetical protein ERGA_CDS_04340
Ehrlichia ruminantium str. Gardel	5.20E-051	Omp85	YP_196792.1	Outer membrane protein omp1
Ehrlichia ruminantium str. Welgevonden	1.30E-011	BamB	YP_180377.1	hypothetical protein Erum5140
Ehrlichia ruminantium str. Welgevonden	1.30E-011	BamB	YP_197415.1	hypothetical protein ERWE_CDS_05390
Ehrlichia ruminantium str. Welgevonden	3.30E-017	BamD	YP_180639.1	hypothetical protein Erum7760
Ehrlichia ruminantium str. Welgevonden	3.30E-017	BamD	YP_197696.1	hypothetical protein ERWE_CDS_08200
Ehrlichia ruminantium str. Welgevonden	9.30E-010	BamE	YP_180289.1	hypothetical protein Erum4230
Ehrlichia ruminantium str. Welgevonden	9.30E-010	BamE	YP_197316.1	hypothetical protein ERWE_CDS_04400
Ehrlichia ruminantium str. Welgevonden	3.80E-051	Omp85	YP_180689.1	Outer membrane protein omp1
Ehrlichia ruminantium str. Welgevonden	3.80E-051	Omp85	YP_197751.1	Outer membrane protein omp1
Erythrobacter litoralis HTCC2594	8.30E-053	BamB	YP_457468.1	hypothetical protein ELI_02895
Erythrobacter litoralis HTCC2594	9.80E-009	BamB	YP_459803.1	alcohol dehydrogenase large subunit
Erythrobacter litoralis HTCC2594	2.40E-018	BamD	YP_458335.1	DNA uptake lipoprotein
Erythrobacter litoralis HTCC2594	7.90E-010	BamE	YP_456931.1	tmRNA-binding small protein A
Erythrobacter litoralis HTCC2594	8.90E-118	Omp85	YP_457652.1	outer membrane protein
Erythrobacter litoralis HTCC2594	1.80E-007	Omp85	YP_457217.1	hypothetical protein ELI_01640
Gluconacetobacter diazotrophicus PAl 5	2.20E-026	BamB	YP_002277450.1	Pyrrolo-quinoline quinone
Gluconacetobacter diazotrophicus PAl 5	6.60E-007	BamB	YP_002274674.1	PQQ-dependent dehydrogenase, methanol/ethanol family
Gluconacetobacter diazotrophicus PAl 5	2.90E-005	BamB	YP_002277434.1	membrane-bound PQQ-dependent dehydrogenase, glucose/quinate/shikimate family
Gluconacetobacter diazotrophicus PAl 5	1.70E-026	BamB	YP_001603491.1	Pyrrolo-quinoline quinone
Gluconacetobacter diazotrophicus PAl 5	6.60E-007	BamB	YP_001602285.1	alcohol dehydrogenase
Gluconacetobacter diazotrophicus PAl 5	2.90E-005	BamB	YP_001603508.1	quinoprotein glucose dehydrogenase
Gluconacetobacter diazotrophicus PAl 5	1.60E-019	BamD	YP_002277507.1	outer membrane assembly lipoprotein YfiO
Gluconacetobacter diazotrophicus PAl 5	1.50E-019	BamD	YP_001603429.1	hypothetical protein GDI_3198
Gluconacetobacter diazotrophicus PAl 5	1.90E-016	BamE	YP_002277631.1	SmpA/OmlA domain-containing protein

alpha_e-5

Gluconacetobacter diazotrophicus PAl 5	1.90E-016	BamE	YP_001603310.1	SmpA/OmlA family lipoprotein
Gluconacetobacter diazotrophicus PAl 5	3.10E-101	Omp85	YP_002274776.1	outer membrane protein assembly complex, YaeT protein
Gluconacetobacter diazotrophicus PAl 5	1.10E-005	Omp85	YP_002274488.1	surface antigen (D15)
Gluconacetobacter diazotrophicus PAl 5	1.50E-088	Omp85	YP_001602390.1	outer membrane protein assembly factor yaeT
Gluconacetobacter diazotrophicus PAl 5	3.00E-006	Omp85	YP_001602089.1	surface antigen
Gluconobacter oxydans 621H	6.10E-027	BamB	YP_192361.1	PQQ-containing dehydrogenase 2
Gluconobacter oxydans 621H	3.60E-007	BamB	YP_191493.1	alcohol dehydrogenase large subunit
Gluconobacter oxydans 621H	5.50E-005	BamB	YP_192251.1	PQQ-containing dehydrogenase 1
Gluconobacter oxydans 621H	7.70E-019	BamD	YP_190615.1	lipoprotein
Gluconobacter oxydans 621H	1.40E-017	BamE	YP_190569.1	hypothetical protein GOX0119
Gluconobacter oxydans 621H	1.30E-093	Omp85	YP_192213.1	Outer membrane protein
Gluconobacter oxydans 621H	8.30E-007	Omp85	YP_192403.1	Outer membrane protein
Granulibacter bethesdensis CGDNIH1	3.00E-045	BamB	YP_746115.1	PQQ repeat-containing protein
Granulibacter bethesdensis CGDNIH1	2.10E-006	BamB	YP_745743.1	methanol dehydrogenase subunit 1
Granulibacter bethesdensis CGDNIH1	8.80E-006	BamB	YP_744165.1	methanol dehydrogenase subunit 1
Granulibacter bethesdensis CGDNIH1	1.10E-005	BamB	YP_744472.1	methanol dehydrogenase subunit 1
Granulibacter bethesdensis CGDNIH1	3.40E-005	BamB	YP_743943.1	methanol dehydrogenase subunit 1
Granulibacter bethesdensis CGDNIH1	7.50E-026	BamD	YP_744242.1	ComL family lipoprotein
Granulibacter bethesdensis CGDNIH1	2.20E-016	BamE	YP_744831.1	SmpA/OmlA family lipoprotein
Granulibacter bethesdensis CGDNIH1	5.40E-005	BamE	YP_745011.1	hypothetical protein GbCGDNIH1_1190
Granulibacter bethesdensis CGDNIH1	6.00E-134	Omp85	YP_744761.1	outer membrane protein assembly factor yaeT
Granulibacter bethesdensis CGDNIH1	4.20E-007	Omp85	YP_743941.1	outer membrane protein
Hirschia baltica ATCC 49814	1.40E-053	BamB	YP_003060335.1	pyrrolo-quinoline quinone
Hirschia baltica ATCC 49814	6.10E-019	BamD	YP_003058807.1	outer membrane assembly lipoprotein YfiO
Hirschia baltica ATCC 49814	2.60E-005	BamD	YP_003060775.1	tol-pal system protein YbgF
Hirschia baltica ATCC 49814	5.10E-019	BamE	YP_003059770.1	SmpA/OmlA domain-containing protein
Hirschia baltica ATCC 49814	4.10E-112	Omp85	YP_003060094.1	outer membrane protein assembly complex, YaeT protein
Hirschia baltica ATCC 49814	1.50E-006	Omp85	YP_003059159.1	surface antigen (D15)
Hyphomicrobium denitrificans ATCC 51888	5.50E-044	BamB	YP_003754995.1	pyrrolo-quinoline quinone
Hyphomicrobium denitrificans ATCC 51888	1.30E-007	BamB	YP_003754935.1	methanol/ethanol family PQQ-dependent dehydrogenase
Hyphomicrobium denitrificans ATCC 51888	2.20E-007	BamB	YP_003754922.1	PQQ-dependent enzyme-like protein
Hyphomicrobium denitrificans ATCC 51888	3.60E-007	BamB	YP_003755456.1	methanol/ethanol family PQQ-dependent dehydrogenase
Hyphomicrobium denitrificans ATCC 51888	7.20E-006	BamB	YP_003754486.1	methanol/ethanol family PQQ-dependent dehydrogenase
Hyphomicrobium denitrificans ATCC 51888	2.40E-005	BamB	YP_003756965.1	methanol/ethanol family PQQ-dependent dehydrogenase
Hyphomicrobium denitrificans ATCC 51888	4.40E-020	BamD	YP_003754397.1	outer membrane assembly lipoprotein YfiO
Hyphomicrobium denitrificans ATCC 51888	1.90E-016	BamE	YP_003755421.1	SmpA/OmlA domain-containing protein
Hyphomicrobium denitrificans ATCC 51888	7.40E-135	Omp85	YP_003756032.1	outer membrane protein assembly complex, YaeT protein
Hyphomicrobium sp. MC1	4.00E-019	BamD	YP_004674155.1	putative lipoprotein UPF0169, hypothetical protein
Hyphomicrobium sp. MC1	1.50E-048	BamB	YP_004676961.1	Pyrrolo-quinoline quinone
Hyphomicrobium sp. MC1	1.40E-006	BamB	YP_004674295.1	methanol dehydrogenase subunit alpha

alpha_e-5

Hyphomicrobium sp. MC1	2.20E-006	BamB	YP_004677526.1	putative PQQ enzyme repeat protein
Hyphomicrobium sp. MC1	3.20E-006	BamB	YP_004675301.1	Pyrrolo-quinoline quinone
Hyphomicrobium sp. MC1	4.00E-006	BamB	YP_004678633.1	alcohol dehydrogenase
Hyphomicrobium sp. MC1	7.00E-006	BamB	YP_004677389.1	putative PQQ-linked dehydrogenase
Hyphomicrobium sp. MC1	8.90E-006	BamB	YP_004677895.1	putative PQQ-linked dehydrogenase
Hyphomicrobium sp. MC1	3.50E-005	BamB	YP_004674607.1	putative PQQ-linked dehydrogenase
Hyphomicrobium sp. MC1	2.10E-020	BamE	YP_004676446.1	hypothetical protein HYPMC_2661
Hyphomicrobium sp. MC1	1.20E-139	Omp85	YP_004676649.1	putative outer membrane protein assembly factor
Hyphomonas neptunium ATCC 15444	9.50E-068	BamB	YP_759501.1	PQQ repeat-containing protein
Hyphomonas neptunium ATCC 15444	3.20E-007	BamB	YP_758863.1	alcohol dehydrogenase (acceptor)
Hyphomonas neptunium ATCC 15444	1.00E-019	BamD	YP_759117.1	putative competence lipoprotein ComL
Hyphomonas neptunium ATCC 15444	9.10E-019	BamE	YP_759878.1	SmpA/OmlA family protein
Hyphomonas neptunium ATCC 15444	1.40E-109	Omp85	YP_760480.1	OMP85 family outer membrane protein
Hyphomonas neptunium ATCC 15444	4.10E-006	Omp85	YP_760558.1	OMP85 family outer membrane protein
Jannaschia sp. CCS1	9.00E-034	BamB	YP_510141.1	Pyrrolo-quinoline quinone
Jannaschia sp. CCS1	1.50E-018	BamD	YP_510690.1	competence lipoprotein ComL
Jannaschia sp. CCS1	1.60E-017	BamE	YP_509730.1	SmpA/OmlA
Jannaschia sp. CCS1	9.50E-152	Omp85	YP_510395.1	surface antigen (D15)
Ketogulonicigenium vulgare Y25	7.80E-023	BamB	YP_003963799.1	pyrrolo-quinoline quinone
Ketogulonicigenium vulgare Y25	1.60E-005	BamB	YP_003964325.1	sorbitol dehydrogenase
Ketogulonicigenium vulgare Y25	2.30E-005	BamB	YP_003963113.1	sorbose dehydrogenase
Ketogulonicigenium vulgare Y25	3.60E-005	BamB	YP_003965035.1	sorbose dehydrogenase
Ketogulonicigenium vulgare Y25	5.40E-005	BamB	YP_003965013.1	sorbose dehydrogenase
Ketogulonicigenium vulgare Y25	5.80E-006	BamB	YP_003965268.1	pyrrolo-quinoline quinone
Ketogulonicigenium vulgare Y25	1.70E-016	BamD	YP_003962855.1	tetratricopeptide TPR_2 repeat protein
Ketogulonicigenium vulgare Y25	9.30E-011	BamE	YP_003963821.1	lipoprotein, SmpA/OmlA family
Ketogulonicigenium vulgare Y25	7.50E-136	Omp85	YP_003963990.1	outer membrane protein
Ketogulonicigenium vulgare Y25	1.80E-005	Omp85	YP_003964196.1	outer membrane protein
Magnetospirillum magneticum AMB-1	1.30E-071	BamB	YP_420706.1	WD-40 repeat-containing protein
Magnetospirillum magneticum AMB-1	1.20E-008	BamB	YP_421058.1	ankyrin repeat-containing protein
Magnetospirillum magneticum AMB-1	1.10E-019	BamD	YP_423219.1	DNA uptake lipoprotein
Magnetospirillum magneticum AMB-1	9.20E-138	Omp85	YP_421853.1	Outer membrane protein/protective antigen OMA87
Maricaulis maris MCS10	1.30E-087	BamB	YP_756433.1	Pyrrolo-quinoline quinone
Maricaulis maris MCS10	2.90E-025	BamD	YP_757298.1	DNA uptake lipoprotein-like protein
Maricaulis maris MCS10	1.30E-018	BamE	YP_756756.1	hypothetical protein Mmar10_1526
Maricaulis maris MCS10	8.30E-128	Omp85	YP_756618.1	surface antigen (D15)
Maricaulis maris MCS10	2.10E-005	Omp85	YP_757868.1	surface antigen (D15)
Mesorhizobium ciceri biovar biserrulae WSM1271	4.20E-005	BamB	YP_004134372.1	pqq-dependent dehydrogenase, methanol/ethanol family
Mesorhizobium ciceri biovar biserrulae WSM1271	1.90E-005	BamB	YP_004142365.1	glucose/quinate/shikimate family membrane-bound PQQ-dependent dehydrogenase
Mesorhizobium ciceri biovar biserrulae WSM1271	2.30E-005	BamB	YP_004143457.1	methanol/ethanol family PQQ-dependent dehydrogenase

Mesorhizobium ciceri biovar biserrulae WSM1271	1.10E-019	BamD	YP_004142332.1	outer membrane assembly lipoprotein YfiO
Mesorhizobium ciceri biovar biserrulae WSM1271	6.20E-035	BamE	YP_004143625.1	SmpA/OmlA domain-containing protein
Mesorhizobium ciceri biovar biserrulae WSM1271	2.60E-112	Omp85	YP_004143140.1	outer membrane protein assembly complex, YaeT protein
Mesorhizobium ciceri biovar biserrulae WSM1271	1.50E-006	Omp85	YP_004142247.1	surface antigen (D15)
Mesorhizobium loti MAFF303099	7.30E-005	BamB	NP_103072.1	glucose dehydrogenase
Mesorhizobium loti MAFF303099	8.20E-020	BamD	NP_103107.1	hypothetical protein mll1543
Mesorhizobium loti MAFF303099	3.40E-034	BamE	NP_108518.1	hypothetical protein mll8419
Mesorhizobium loti MAFF303099	8.80E-005	BamE	NP_106001.1	hypothetical protein mlr5313
Mesorhizobium loti MAFF303099	2.40E-110	Omp85	NP_102404.1	outer membrane protein
Mesorhizobium loti MAFF303099	4.50E-006	Omp85	NP_103198.1	hypothetical protein mll1662
Mesorhizobium opportunistum WSM2075	9.60E-006	BamB	YP_004612117.1	membrane-bound PQQ-dependent dehydrogenase, glucose/quinate/shikimate family
Mesorhizobium opportunistum WSM2075	5.50E-005	BamB	YP_004614925.1	membrane-bound PQQ-dependent dehydrogenase, glucose/quinate/shikimate family
Mesorhizobium opportunistum WSM2075	7.60E-005	BamB	YP_004612889.1	PQQ-dependent dehydrogenase, methanol/ethanol family
Mesorhizobium opportunistum WSM2075	1.80E-019	BamD	YP_004612085.1	outer membrane assembly lipoprotein YfiO
Mesorhizobium opportunistum WSM2075	2.30E-036	BamE	YP_004613427.1	SmpA/OmlA domain-containing protein
Mesorhizobium opportunistum WSM2075	9.40E-005	BamE	YP_004608786.1	ErfK/YbiS/YcfS/YnhG family protein
Mesorhizobium opportunistum WSM2075	1.20E-105	Omp85	YP_004612788.1	outer membrane protein assembly complex, YaeT protein
Mesorhizobium opportunistum WSM2075	1.40E-006	Omp85	YP_004611995.1	surface antigen (D15)
Mesorhizobium sp. BNC1	5.70E-007	BamB	YP_675152.1	Pyrrolo-quinoline quinone
Mesorhizobium sp. BNC1	7.60E-007	BamB	YP_675153.1	Pyrrolo-quinoline quinone
Mesorhizobium sp. BNC1	2.00E-006	BamB	YP_675431.1	Pyrrolo-quinoline quinone
Mesorhizobium sp. BNC1	4.50E-005	BamB	YP_676061.1	quinoprotein glucose dehydrogenase
Mesorhizobium sp. BNC1	1.40E-022	BamD	YP_674557.1	putative lipoprotein
Mesorhizobium sp. BNC1	1.30E-005	BamD	YP_672684.1	tetratricopeptide TPR_2
Mesorhizobium sp. BNC1	1.10E-037	BamE	YP_673703.1	SmpA/OmlA
Mesorhizobium sp. BNC1	1.80E-118	Omp85	YP_673949.1	surface antigen (D15)
Mesorhizobium sp. BNC1	7.30E-006	Omp85	YP_674082.1	surface antigen (D15)
Methylobacterium chloromethanicum CM4	1.60E-007	BamB	YP_002423677.1	pyrrolo-quinoline quinone
Methylobacterium chloromethanicum CM4	3.60E-007	BamB	YP_002423222.1	methanol/ethanol family PQQ-dependent dehydrogenase
Methylobacterium chloromethanicum CM4	4.40E-006	BamB	YP_002420334.1	methanol/ethanol family PQQ-dependent dehydrogenase
Methylobacterium chloromethanicum CM4	5.00E-006	BamB	YP_002424324.1	pyrrolo-quinoline quinone
Methylobacterium chloromethanicum CM4	5.00E-021	BamD	YP_002421944.1	outer membrane assembly lipoprotein YfiO
Methylobacterium chloromethanicum CM4	1.30E-027	BamE	YP_002421098.1	SmpA/OmlA domain-containing protein
Methylobacterium chloromethanicum CM4	2.90E-086	Omp85	YP_002421128.1	outer membrane protein assembly complex, YaeT protein
Methylobacterium chloromethanicum CM4	7.00E-084	Omp85	YP_002423485.1	outer membrane protein assembly complex, YaeT protein
Methylobacterium chloromethanicum CM4	1.90E-006	Omp85	YP_002421758.1	surface antigen (D15)
Methylobacterium extorquens AM1	9.60E-021	BamD	YP_002964181.1	lipoprotein UPF0169, exported protein
Methylobacterium extorquens AM1	1.60E-007	BamB	YP_002965854.1	quinoprotein alcohol dehydrogenase
Methylobacterium extorquens AM1	1.40E-006	BamB	YP_002965446.1	methanol dehydrogenase subunit 1 precursor (MDH large alpha subunit) (MEDH)

Methylobacterium extorquens AM1	4.40E-006	BamB	YP_002962288.1	quinoprotein ethanol dehydrogenase precursor (QEDH)
Methylobacterium extorquens AM1	1.30E-027	BamE	YP_002963111.1	hypothetical protein MexAM1_META1p2032
Methylobacterium extorquens AM1	2.90E-086	Omp85	YP_002963144.1	outer membrane protein assembly factor
Methylobacterium extorquens AM1	2.20E-006	Omp85	YP_002963991.1	hypothetical protein MexAM1_META1p2962
Methylobacterium extorquens DM4	5.00E-021	BamD	YP_003069211.1	lipoprotein UPF0169, exported protein
Methylobacterium extorquens DM4	1.60E-007	BamB	YP_003070985.1	quinoprotein alcohol dehydrogenase
Methylobacterium extorquens DM4	1.40E-006	BamB	YP_003070571.1	methanol dehydrogenase subunit 1
Methylobacterium extorquens DM4	4.40E-006	BamB	YP_003067544.1	quinoprotein ethanol dehydrogenase
Methylobacterium extorquens DM4	1.30E-027	BamE	YP_003068332.1	hypothetical protein METDI2815
Methylobacterium extorquens DM4	2.90E-086	Omp85	YP_003068363.1	outer membrane protein assembly factor
Methylobacterium extorquens DM4	2.00E-006	Omp85	YP_003069025.1	hypothetical protein METDI3531
Methylobacterium extorquens PA1	1.80E-007	BamB	YP_001641963.1	Pyrrolo-quinoline quinone
Methylobacterium extorquens PA1	3.60E-007	BamB	YP_001641590.1	methanol/ethanol family PQQ-dependent dehydrogenase
Methylobacterium extorquens PA1	4.40E-006	BamB	YP_001638811.1	methanol/ethanol family PQQ-dependent dehydrogenase
Methylobacterium extorquens PA1	1.90E-005	BamB	YP_001637598.1	methanol/ethanol family PQQ-dependent dehydrogenase
Methylobacterium extorquens PA1	5.00E-021	BamD	YP_001640411.1	putative lipoprotein
Methylobacterium extorquens PA1	1.30E-027	BamE	YP_001639517.1	SmpA/OmlA domain-containing protein
Methylobacterium extorquens PA1	6.30E-088	Omp85	YP_001639548.1	outer membrane protein assembly complex, YaeT protein
Methylobacterium extorquens PA1	1.90E-006	Omp85	YP_001640227.1	surface antigen (D15)
Methylobacterium nodulans ORS 2060	3.30E-007	BamB	YP_002490201.1	PQQ-dependent dehydrogenase, methanol/ethanol family
Methylobacterium nodulans ORS 2060	6.80E-005	BamB	YP_002497622.1	PQQ-dependent dehydrogenase
Methylobacterium nodulans ORS 2060	2.40E-021	BamD	YP_002502474.1	outer membrane assembly lipoprotein YfiO
Methylobacterium nodulans ORS 2060	5.80E-025	BamE	YP_002502122.1	SmpA/OmlA domain-containing protein
Methylobacterium nodulans ORS 2060	3.50E-080	Omp85	YP_002496826.1	outer membrane protein assembly complex, YaeT protein
Methylobacterium nodulans ORS 2060	1.20E-007	Omp85	YP_002501840.1	surface antigen (D15)
Methylobacterium populi BJ001	1.00E-007	BamB	YP_001927668.1	pyrrolo-quinoline quinone
Methylobacterium populi BJ001	2.30E-007	BamB	YP_001927264.1	PQQ-dependent dehydrogenase, methanol/ethanol family
Methylobacterium populi BJ001	4.20E-006	BamB	YP_001927504.1	PQQ-dependent dehydrogenase, methanol/ethanol family
Methylobacterium populi BJ001	5.10E-024	BamD	YP_001925822.1	lipoprotein
Methylobacterium populi BJ001	2.50E-028	BamE	YP_001924709.1	SmpA/OmlA domain-containing protein
Methylobacterium populi BJ001	4.20E-087	Omp85	YP_001924739.1	outer membrane protein assembly complex, YaeT protein
Methylobacterium populi BJ001	1.90E-006	Omp85	YP_001925578.1	surface antigen (D15)
Methylobacterium radiotolerans JCM 2831	7.30E-008	BamB	YP_001753502.1	Pyrrolo-quinoline quinone
Methylobacterium radiotolerans JCM 2831	3.20E-007	BamB	YP_001756860.1	methanol/ethanol family PQQ-dependent dehydrogenase
Methylobacterium radiotolerans JCM 2831	4.70E-006	BamB	YP_001755087.1	methanol/ethanol family PQQ-dependent dehydrogenase
Methylobacterium radiotolerans JCM 2831	9.20E-006	BamB	YP_001754610.1	methanol/ethanol family PQQ-dependent dehydrogenase
Methylobacterium radiotolerans JCM 2831	5.70E-027	BamD	YP_001755026.1	putative lipoprotein
Methylobacterium radiotolerans JCM 2831	5.60E-024	BamE	YP_001756629.1	SmpA/OmlA domain-containing protein
Methylobacterium radiotolerans JCM 2831	1.60E-079	Omp85	YP_001756099.1	outer membrane protein assembly complex, YaeT protein
Methylobacterium radiotolerans JCM 2831	1.40E-006	Omp85	YP_001755067.1	surface antigen (D15)

Methylobacterium sp. 4-46	8.10E-006	BamB	YP_001770439.1	methanol/ethanol family PQQ-dependent dehydrogenase
Methylobacterium sp. 4-46	3.90E-005	BamB	YP_001769840.1	methanol/ethanol family PQQ-dependent dehydrogenase
Methylobacterium sp. 4-46	8.70E-005	BamB	YP_001768979.1	methanol/ethanol family PQQ-dependent dehydrogenase
Methylobacterium sp. 4-46	9.90E-005	BamB	YP_001772474.1	methanol/ethanol family PQQ-dependent dehydrogenase
Methylobacterium sp. 4-46	1.50E-021	BamD	YP_001773380.1	putative lipoprotein
Methylobacterium sp. 4-46	2.10E-024	BamE	YP_001772927.1	SmpA/OmlA domain-containing protein
Methylobacterium sp. 4-46	5.90E-084	Omp85	YP_001767633.1	outer membrane protein assembly complex, YaeT protein
Methylobacterium sp. 4-46	4.60E-007	Omp85	YP_001773084.1	surface antigen (D15)
Methylocella silvestris BL2	4.90E-007	BamB	YP_002363977.1	Pyrrolo-quinoline quinone
Methylocella silvestris BL2	8.50E-007	BamB	YP_002363146.1	Pyrrolo-quinoline quinone
Methylocella silvestris BL2	1.20E-006	BamB	YP_002360806.1	PQQ-dependent dehydrogenase
Methylocella silvestris BL2	2.40E-006	BamB	YP_002362556.1	PQQ-dependent dehydrogenase
Methylocella silvestris BL2	3.90E-006	BamB	YP_002361898.1	PQQ-dependent dehydrogenase
Methylocella silvestris BL2	6.70E-006	BamB	YP_002363420.1	PQQ-dependent dehydrogenase
Methylocella silvestris BL2	2.60E-005	BamB	YP_002363652.1	PQQ-dependent dehydrogenase
Methylocella silvestris BL2	6.10E-019	BamD	YP_002363747.1	outer membrane assembly lipoprotein YfiO
Methylocella silvestris BL2	3.00E-026	BamE	YP_002360990.1	SmpA/OmlA domain-containing protein
Methylocella silvestris BL2	1.70E-124	Omp85	YP_002364081.1	outer membrane protein assembly complex, YaeT protein
Methylocella silvestris BL2	8.30E-007	Omp85	YP_002362901.1	surface antigen (D15)
Neorickettsia risticii str. Illinois	1.30E-012	BamD	YP_003082089.1	competence protein ComL
Neorickettsia risticii str. Illinois	4.10E-042	Omp85	YP_003081906.1	outer membrane protein assembly complex, YaeT protein
Neorickettsia sennetsu str. Miyayama	2.20E-011	BamD	YP_506787.1	putative competence protein ComL
Neorickettsia sennetsu str. Miyayama	2.00E-007	BamE	YP_506092.1	putative lipoprotein
Neorickettsia sennetsu str. Miyayama	2.70E-039	Omp85	YP_506594.1	OMP85 family outer membrane protein
Nitrobacter hamburgensis X14	3.70E-019	BamD	YP_576573.1	putative lipoprotein
Nitrobacter hamburgensis X14	7.00E-006	BamD	YP_577425.1	tetratricopeptide TPR_2
Nitrobacter hamburgensis X14	4.40E-022	BamE	YP_576863.1	SmpA/OmlA
Nitrobacter hamburgensis X14	6.50E-106	Omp85	YP_576978.1	surface antigen (D15)
Nitrobacter winogradskyi Nb-255	2.30E-019	BamD	YP_317674.1	putative lipoprotein
Nitrobacter winogradskyi Nb-255	1.80E-005	BamD	YP_318355.1	hypothetical protein Nwi_1742
Nitrobacter winogradskyi Nb-255	3.60E-022	BamE	YP_318016.1	SmpA/OmlA
Nitrobacter winogradskyi Nb-255	1.40E-103	Omp85	YP_318463.1	Outer membrane protein
Nitrobacter winogradskyi Nb-255	1.40E-006	Omp85	YP_317491.1	surface antigen (D15)
Novosphingobium aromaticivorans DSM 12444	3.30E-068	BamB	YP_496223.1	Pyrrolo-quinoline quinone
Novosphingobium aromaticivorans DSM 12444	5.10E-009	BamB	YP_498148.1	Pyrrolo-quinoline quinone
Novosphingobium aromaticivorans DSM 12444	1.40E-007	BamB	YP_498140.1	Pyrrolo-quinoline quinone
Novosphingobium aromaticivorans DSM 12444	9.30E-019	BamD	YP_497006.1	DNA uptake lipoprotein
Novosphingobium aromaticivorans DSM 12444	8.20E-014	BamE	YP_497906.1	SmpA/OmlA
Novosphingobium aromaticivorans DSM 12444	6.70E-006	BamE	YP_498100.1	hypothetical protein Saro_2830
Novosphingobium aromaticivorans DSM 12444	2.00E-136	Omp85	YP_496655.1	surface antigen (D15)

Novosphingobium aromaticivorans DSM 12444	1.80E-007	Omp85	YP_496381.1	surface antigen (D15)
Novosphingobium sp. PP1Y	9.60E-069	BamB	YP_004533285.1	pyrrolo-quinoline quinone
Novosphingobium sp. PP1Y	7.80E-009	BamB	YP_004533882.1	pyrrolo-quinoline quinone
Novosphingobium sp. PP1Y	1.50E-007	BamB	YP_004533356.1	pyrrolo-quinoline quinone
Novosphingobium sp. PP1Y	1.10E-006	BamB	YP_004533894.1	alcohol dehydrogenase large subunit
Novosphingobium sp. PP1Y	2.60E-006	BamB	YP_004538410.1	alcohol dehydrogenase
Novosphingobium sp. PP1Y	1.80E-005	BamB	YP_004538555.1	pyrrolo-quinoline quinone
Novosphingobium sp. PP1Y	7.80E-020	BamD	YP_004535654.1	DNA uptake lipoprotein
Novosphingobium sp. PP1Y	7.00E-011	BamE	YP_004533951.1	SmpA/OmlA protein
Novosphingobium sp. PP1Y	6.70E-135	Omp85	YP_004534135.1	surface antigen (D15)
Novosphingobium sp. PP1Y	7.00E-007	Omp85	YP_004535118.1	surface antigen (D15)
Ochrobactrum anthropi ATCC 49188	4.70E-006	BamB	YP_001368854.1	Pyrrolo-quinoline quinone
Ochrobactrum anthropi ATCC 49188	4.40E-024	BamD	YP_001370297.1	competence protein ComL
Ochrobactrum anthropi ATCC 49188	2.90E-056	BamE	YP_001371065.1	SmpA/OmlA domain-containing protein
Ochrobactrum anthropi ATCC 49188	2.00E-120	Omp85	YP_001370581.1	surface antigen (D15)
Ochrobactrum anthropi ATCC 49188	1.90E-006	Omp85	YP_001368613.1	surface antigen (D15)
Oligotropha carboxidovorans OM5	3.90E-019	BamD	YP_002288249.1	coml, competence lipoprotein
Oligotropha carboxidovorans OM5	5.00E-025	BamE	YP_002288862.1	SmpA/OmlA
Oligotropha carboxidovorans OM5	6.30E-005	BamE	YP_002287554.1	hypothetical protein OCAR_4544
Oligotropha carboxidovorans OM5	3.50E-108	Omp85	YP_002288949.1	outer membrane protein assembly complex
Orientia tsutsugamushi str. Boryong	3.00E-013	BamB	YP_001248882.1	hypothetical protein OTBS_1467
Orientia tsutsugamushi str. Boryong	2.60E-012	BamD	YP_001249195.1	TPR repeat-containing protein
Orientia tsutsugamushi str. Boryong	8.70E-008	BamE	YP_001248357.1	outer membrane lipoprotein
Orientia tsutsugamushi str. Boryong	3.40E-023	Omp85	YP_001248342.1	outer membrane protein
Orientia tsutsugamushi str. Ikeda	2.10E-014	BamB	YP_001937474.1	hypothetical protein OTT_0782
Orientia tsutsugamushi str. Ikeda	1.50E-012	BamD	YP_001936862.1	ComL-like lipoprotein
Orientia tsutsugamushi str. Ikeda	1.10E-007	BamE	YP_001936707.1	hypothetical protein homologous to outer membrane lipoprotein
Orientia tsutsugamushi str. Ikeda	3.30E-017	Omp85	YP_001936722.1	outer membrane protein Omp1
Paracoccus denitrificans PD1222	2.00E-023	BamB	YP_916209.1	Pyrrolo-quinoline quinone
Paracoccus denitrificans PD1222	7.00E-006	BamB	YP_913833.1	Pyrrolo-quinoline quinone
Paracoccus denitrificans PD1222	1.40E-005	BamB	YP_916772.1	Pyrrolo-quinoline quinone
Paracoccus denitrificans PD1222	2.30E-018	BamD	YP_918245.1	putative ComL lipoprotein
Paracoccus denitrificans PD1222	4.10E-011	BamE	YP_915685.1	hypothetical protein Pden_1894
Paracoccus denitrificans PD1222	6.30E-005	Omp85	YP_916462.1	surface antigen (D15)
Paracoccus denitrificans PD1222	3.90E-159	Omp85	YP_917761.1	surface antigen (D15)
Parvibaculum lavamentivorans DS-1	7.80E-083	BamB	YP_001414749.1	Pyrrolo-quinoline quinone
Parvibaculum lavamentivorans DS-1	6.20E-008	BamB	YP_001412583.1	Pyrrolo-quinoline quinone
Parvibaculum lavamentivorans DS-1	3.40E-031	BamD	YP_001413696.1	DNA uptake lipoprotein
Parvibaculum lavamentivorans DS-1	3.10E-027	BamE	YP_001414183.1	SmpA/OmlA domain-containing protein
Parvibaculum lavamentivorans DS-1	3.40E-136	Omp85	YP_001414451.1	surface antigen (D15)

Parvibaculum lavamentivorans DS-1	6.60E-007	Omp85	YP_001411664.1	surface antigen (D15)
Parvularcula bermudensis HTCC2503	1.40E-057	BamB	YP_003855715.1	PQQ enzyme repeat family protein
Parvularcula bermudensis HTCC2503	3.10E-023	BamD	YP_003855280.1	competence lipoprotein ComL
Parvularcula bermudensis HTCC2503	3.80E-006	BamE	YP_003854845.1	lipoprotein, SmpA/OmlA family protein
Parvularcula bermudensis HTCC2503	3.40E-103	Omp85	YP_003853707.1	outer membrane protein
Parvularcula bermudensis HTCC2503	2.90E-006	Omp85	YP_003855793.1	hypothetical protein PB2503_13069
Phenylobacterium zucineum HLK1	1.10E-139	BamB	YP_002130375.1	PQQ enzyme repeat domain protein
Phenylobacterium zucineum HLK1	8.00E-008	BamB	YP_002131950.1	glucose dehydrogenase
Phenylobacterium zucineum HLK1	4.90E-006	BamB	YP_002128900.1	glucose dehydrogenase
Phenylobacterium zucineum HLK1	4.20E-005	BamB	YP_002130431.1	glucose dehydrogenase
Phenylobacterium zucineum HLK1	1.50E-020	BamD	YP_002131150.1	DNA uptake lipoprotein
Phenylobacterium zucineum HLK1	2.70E-028	BamE	YP_002128768.1	small protein A (tmRNA-binding)
Phenylobacterium zucineum HLK1	3.20E-031	BamE	YP_002130503.1	small protein A (tmRNA-binding)
Phenylobacterium zucineum HLK1	7.50E-005	BamE	YP_002132202.1	hypothetical protein PHZ_c3364
Phenylobacterium zucineum HLK1	1.20E-135	Omp85	YP_002130612.1	outer membrane protein
Phenylobacterium zucineum HLK1	6.90E-007	Omp85	YP_002130250.1	hypothetical protein PHZ_c1407
Polymorphum gilvum SL003B-26A1	4.20E-038	BamB	YP_004304144.1	PQQ enzyme repeat domain-containing protein
Polymorphum gilvum SL003B-26A1	2.20E-005	BamB	YP_004302745.1	methanol dehydrogenase large subunit-like protein
Polymorphum gilvum SL003B-26A1	1.70E-020	BamD	YP_004303083.1	DNA uptake lipoprotein-like protein
Polymorphum gilvum SL003B-26A1	2.60E-025	BamE	YP_004303653.1	SmpA/OmlA protein
Polymorphum gilvum SL003B-26A1	1.50E-150	Omp85	YP_004303828.1	Outer membrane protein assembly complex, YaeT protein
Polymorphum gilvum SL003B-26A1	1.90E-006	Omp85	YP_004304522.1	Outer membrane protein, OMP85 family
Rhizobium etli CFN 42	2.30E-005	BamB	YP_468754.1	glucose dehydrogenase (pyrroloquinoline-quinone) protein
Rhizobium etli CFN 42	7.20E-020	BamD	YP_470333.1	hypothetical protein RHE_CH02838
Rhizobium etli CFN 42	5.30E-032	BamE	YP_469058.1	hypothetical protein RHE_CH01528
Rhizobium etli CFN 42	1.50E-122	Omp85	YP_469433.1	outer membrane lipoprotein
Rhizobium etli CFN 42	9.50E-007	Omp85	YP_471322.1	outer membrane protein, protective antigen
Rhizobium etli CIAT 652	4.60E-005	BamB	YP_001977468.1	glucose dehydrogenase (pyrroloquinoline-quinone) protein
Rhizobium etli CIAT 652	1.90E-019	BamD	YP_001979115.1	competence lipoprotein protein
Rhizobium etli CIAT 652	1.10E-031	BamE	YP_001977755.1	outer membrane protein
Rhizobium etli CIAT 652	5.80E-122	Omp85	YP_001978156.1	outer membrane lipoprotein
Rhizobium etli CIAT 652	7.10E-007	Omp85	YP_001980236.1	outer membrane protein, protective antigen
Rhizobium leguminosarum bv. trifolii WSM1325	4.90E-006	BamB	YP_002974814.1	membrane-bound PQQ-dependent dehydrogenase, glucose/quinate/shikimate family
Rhizobium leguminosarum bv. trifolii WSM1325	1.50E-019	BamD	YP_002976642.1	outer membrane assembly lipoprotein YfiO
Rhizobium leguminosarum bv. trifolii WSM1325	3.20E-032	BamE	YP_002975113.1	SmpA/OmlA domain protein
Rhizobium leguminosarum bv. trifolii WSM1325	8.20E-121	Omp85	YP_002975607.1	outer membrane protein assembly complex, YaeT protein
Rhizobium leguminosarum bv. trifolii WSM1325	1.10E-006	Omp85	YP_002977695.1	surface antigen (D15)
Rhizobium leguminosarum bv. trifolii WSM2304	1.90E-005	BamB	YP_002280376.1	membrane-bound PQQ-dependent dehydrogenase, glucose/quinate/shikimate family
Rhizobium leguminosarum bv. trifolii WSM2304	1.60E-019	BamD	YP_002282081.1	outer membrane assembly lipoprotein YfiO

Rhizobium leguminosarum bv. trifolii WSM2304	2.80E-029	BamE	YP_002280713.1	SmpA/OmlA domain-containing protein
Rhizobium leguminosarum bv. trifolii WSM2304	4.80E-122	Omp85	YP_002281106.1	outer membrane protein assembly complex, YaeT protein
Rhizobium leguminosarum bv. trifolii WSM2304	8.60E-007	Omp85	YP_002283117.1	hypothetical protein Rleg2_3626
Rhizobium leguminosarum bv. viciae 3841	6.00E-006	BamB	YP_766960.1	quinoprotein glucose dehydrogenase
Rhizobium leguminosarum bv. viciae 3841	1.10E-019	BamD	YP_768875.1	competence lipoprotein ComL protein
Rhizobium leguminosarum bv. viciae 3841	1.20E-031	BamE	YP_767239.1	outer membrane protein
Rhizobium leguminosarum bv. viciae 3841	4.40E-120	Omp85	YP_767822.1	outer membrane protein
Rhizobium leguminosarum bv. viciae 3841	5.50E-007	Omp85	YP_769952.1	cell surface protein
Rhodobacter capsulatus SB 1003	5.10E-029	BamB	YP_003578440.1	PQQ enzyme repeat family protein
Rhodobacter capsulatus SB 1003	8.70E-008	BamB	YP_003577809.1	quinoprotein ethanol dehydrogenase
Rhodobacter capsulatus SB 1003	2.60E-007	BamB	YP_003577549.1	quinoprotein ethanol dehydrogenase
Rhodobacter capsulatus SB 1003	2.00E-017	BamD	YP_003577000.1	competence lipoprotein ComL
Rhodobacter capsulatus SB 1003	4.70E-017	BamE	YP_003578059.1	SmpA/OmlA domain-containing protein
Rhodobacter capsulatus SB 1003	4.20E-149	Omp85	YP_003577784.1	outer membrane protein assembly factor YaeT
Rhodobacter capsulatus SB 1003	3.40E-005	Omp85	YP_003576199.1	surface antigen
Rhodobacter sphaeroides 2.4.1	3.80E-036	BamB	YP_352753.1	putative quinoprotein
Rhodobacter sphaeroides 2.4.1	3.60E-006	BamB	YP_352728.1	quinoprotein glucose dehydrogenase
Rhodobacter sphaeroides 2.4.1	2.40E-005	BamB	YP_352636.1	putative PQQ dehydrogenase protein
Rhodobacter sphaeroides 2.4.1	6.90E-018	BamD	YP_352172.1	putative ComL lipoprotein
Rhodobacter sphaeroides 2.4.1	9.30E-012	BamE	YP_352674.1	hypothetical protein RSP_2616
Rhodobacter sphaeroides 2.4.1	3.50E-169	Omp85	YP_352767.1	putative outer membrane protein
Rhodobacter sphaeroides 2.4.1	4.10E-005	Omp85	YP_354267.1	putative outer membrane protein
Rhodobacter sphaeroides ATCC 17025	7.40E-030	BamB	YP_001168356.1	Pyrrolo-quinoline quinone
Rhodobacter sphaeroides ATCC 17025	4.00E-005	BamB	YP_001167417.1	quinoprotein glucose dehydrogenase
Rhodobacter sphaeroides ATCC 17025	5.10E-005	BamB	YP_001168141.1	Pyrrolo-quinoline quinone
Rhodobacter sphaeroides ATCC 17025	5.80E-018	BamD	YP_001166913.1	TPR repeat-containing protein
Rhodobacter sphaeroides ATCC 17025	9.90E-013	BamE	YP_001168104.1	SmpA/OmlA domain-containing protein
Rhodobacter sphaeroides ATCC 17025	1.70E-169	Omp85	YP_001168341.1	surface antigen (D15)
Rhodobacter sphaeroides ATCC 17025	4.90E-005	Omp85	YP_001168813.1	surface antigen (D15)
Rhodobacter sphaeroides ATCC 17029	4.20E-035	BamB	YP_001043237.1	Pyrrolo-quinoline quinone
Rhodobacter sphaeroides ATCC 17029	6.20E-006	BamB	YP_001043212.1	quinoprotein glucose dehydrogenase
Rhodobacter sphaeroides ATCC 17029	2.30E-005	BamB	YP_001043118.1	Pyrrolo-quinoline quinone
Rhodobacter sphaeroides ATCC 17029	4.80E-018	BamD	YP_001042675.1	TPR repeat-containing protein
Rhodobacter sphaeroides ATCC 17029	1.80E-012	BamE	YP_001043156.1	SmpA/OmlA domain-containing protein
Rhodobacter sphaeroides ATCC 17029	2.80E-169	Omp85	YP_001043251.1	surface antigen (D15)
Rhodobacter sphaeroides ATCC 17029	3.50E-005	Omp85	YP_001044718.1	surface antigen (D15)
Rhodobacter sphaeroides KD131	1.70E-034	BamB	YP_002525374.1	Pyrrolo-quinoline quinone
Rhodobacter sphaeroides KD131	4.50E-006	BamB	YP_002525348.1	Quinoprotein glucose dehydrogenase
Rhodobacter sphaeroides KD131	2.40E-005	BamB	YP_002525251.1	Pyrrolo-quinoline quinone
Rhodobacter sphaeroides KD131	4.80E-018	BamD	YP_002524788.1	hypothetical protein RSKD131_0427

alpha_e-5

Rhodobacter sphaeroides KD131	1.30E-009	BamE	YP_002525289.1	SmpA/OmlA domain-containing protein
Rhodobacter sphaeroides KD131	1.50E-166	Omp85	YP_002525389.1	Surface antigen (D15)
Rhodobacter sphaeroides KD131	3.00E-005	Omp85	YP_002526941.1	Surface antigen (D15)
Rhodomicrobium vannielii ATCC 17100	1.20E-043	BamB	YP_004012494.1	pyrrolo-quinoline quinone repeat-containing protein
Rhodomicrobium vannielii ATCC 17100	2.70E-006	BamB	YP_004012163.1	PQQ-dependent dehydrogenase
Rhodomicrobium vannielii ATCC 17100	4.10E-022	BamD	YP_004012432.1	outer membrane assembly lipoprotein YfiO
Rhodomicrobium vannielii ATCC 17100	7.50E-028	BamE	YP_004011666.1	SmpA/OmlA domain-containing protein
Rhodomicrobium vannielii ATCC 17100	4.40E-116	Omp85	YP_004012337.1	outer membrane protein assembly complex, YaeT protein
Rhodomicrobium vannielii ATCC 17100	3.30E-096	Omp85	YP_004013249.1	outer membrane protein assembly complex, YaeT protein
Rhodopseudomonas palustris BisA53	3.30E-007	BamB	YP_782055.1	Pyrrolo-quinoline quinone
Rhodopseudomonas palustris BisA53	3.80E-007	BamB	YP_782435.1	Pyrrolo-quinoline quinone
Rhodopseudomonas palustris BisA53	1.70E-006	BamB	YP_779787.1	Pyrrolo-quinoline quinone
Rhodopseudomonas palustris BisA53	1.60E-018	BamD	YP_781042.1	putative lipoprotein
Rhodopseudomonas palustris BisA53	1.20E-024	BamE	YP_781739.1	SmpA/OmlA domain-containing protein
Rhodopseudomonas palustris BisA53	2.40E-100	Omp85	YP_781479.1	surface antigen (D15)
Rhodopseudomonas palustris BisB18	1.10E-007	BamB	YP_531783.1	Pyrrolo-quinoline quinone
Rhodopseudomonas palustris BisB18	2.30E-018	BamD	YP_533157.1	putative lipoprotein
Rhodopseudomonas palustris BisB18	4.20E-020	BamE	YP_532540.1	SmpA/OmlA
Rhodopseudomonas palustris BisB18	3.70E-114	Omp85	YP_532314.1	surface antigen (D15)
Rhodopseudomonas palustris BisB5	3.30E-008	BamB	YP_570237.1	Pyrrolo-quinoline quinone
Rhodopseudomonas palustris BisB5	5.60E-018	BamD	YP_570509.1	putative lipoprotein
Rhodopseudomonas palustris BisB5	3.20E-019	BamE	YP_569812.1	SmpA/OmlA
Rhodopseudomonas palustris BisB5	1.10E-104	Omp85	YP_569978.1	surface antigen (D15)
Rhodopseudomonas palustris CGA009	6.80E-008	BamB	NP_948527.1	alcohol dehydrogenase
Rhodopseudomonas palustris CGA009	3.30E-018	BamD	NP_948858.1	putative lipoprotein
Rhodopseudomonas palustris CGA009	2.00E-022	BamE	NP_948080.1	SmpA/OmlA
Rhodopseudomonas palustris CGA009	4.60E-107	Omp85	NP_948255.1	putative outer membrane protein
Rhodopseudomonas palustris DX-1	5.70E-008	BamB	YP_004108567.1	PQQ-dependent dehydrogenase
Rhodopseudomonas palustris DX-1	6.00E-019	BamD	YP_004108179.1	outer membrane assembly lipoprotein YfiO
Rhodopseudomonas palustris DX-1	2.00E-022	BamE	YP_004109084.1	SmpA/OmlA domain-containing protein
Rhodopseudomonas palustris DX-1	2.70E-106	Omp85	YP_004108911.1	outer membrane protein assembly complex, YaeT protein
Rhodopseudomonas palustris HaA2	6.00E-008	BamB	YP_485971.1	Pyrrolo-quinoline quinone
Rhodopseudomonas palustris HaA2	2.00E-018	BamD	YP_485625.1	putative lipoprotein
Rhodopseudomonas palustris HaA2	3.30E-023	BamE	YP_486259.1	SmpA/OmlA
Rhodopseudomonas palustris HaA2	2.90E-105	Omp85	YP_486433.1	surface antigen (D15)
Rhodopseudomonas palustris HaA2	1.00E-097	Omp85	YP_484373.1	surface antigen
Rhodopseudomonas palustris TIE-1	6.30E-008	BamB	YP_001992576.1	PQQ-dependent dehydrogenase, methanol/ethanol family
Rhodopseudomonas palustris TIE-1	2.70E-018	BamD	YP_001993009.1	outer membrane assembly lipoprotein YfiO
Rhodopseudomonas palustris TIE-1	3.40E-023	BamE	YP_001991997.1	SmpA/OmlA domain-containing protein
Rhodopseudomonas palustris TIE-1	4.60E-107	Omp85	YP_001992237.1	outer membrane protein assembly complex, YaeT protein

Rhodospirillum centenum SW	5.00E-083	BamB	YP_002297619.1	PQQ enzyme repeat family protein
Rhodospirillum centenum SW	3.30E-005	BamB	YP_002300042.1	quinoprotein glucose dehydrogenase
Rhodospirillum centenum SW	6.40E-020	BamD	YP_002296853.1	tetratricopeptide repeat family protein
Rhodospirillum centenum SW	2.60E-029	BamE	YP_002297549.1	SmpA
Rhodospirillum centenum SW	1.90E-146	Omp85	YP_002297427.1	outer membrane protein, putative
Rhodospirillum centenum SW	1.80E-007	Omp85	YP_002300080.1	outer membrane protein, OMP85 family
Rhodospirillum rubrum ATCC 11170	2.10E-064	BamB	YP_428167.1	Pyrrolo-quinoline quinone
Rhodospirillum rubrum ATCC 11170	1.70E-032	BamD	YP_426029.1	competence lipoprotein ComL
Rhodospirillum rubrum ATCC 11170	5.50E-005	BamD	YP_427440.1	tetratricopeptide TPR_4
Rhodospirillum rubrum ATCC 11170	1.30E-056	BamE	YP_426749.1	smpA/OmlA
Rhodospirillum rubrum ATCC 11170	1.60E-141	Omp85	YP_426681.1	surface antigen D15
Rhodospirillum rubrum ATCC 11170	4.40E-006	Omp85	YP_427186.1	surface antigen
Rickettsia africae ESF-5	3.30E-219	BamB	YP_002844981.1	WD40-like repeat protein
Rickettsia africae ESF-5	1.50E-017	BamD	YP_002844927.1	DNA uptake lipoprotein
Rickettsia africae ESF-5	8.70E-010	BamE	YP_002845638.1	tmRNA-binding protein
Rickettsia africae ESF-5	1.00E-081	Omp85	YP_002844903.1	Outer membrane protein omp1
Rickettsia akari str. Hartford	5.90E-201	BamB	YP_001493161.1	WD-40 repeat-containing protein
Rickettsia akari str. Hartford	1.30E-017	BamD	YP_001493089.1	hypothetical protein A1C_01315
Rickettsia akari str. Hartford	9.80E-010	BamE	YP_001493946.1	hypothetical protein A1C_06025
Rickettsia akari str. Hartford	2.60E-083	Omp85	YP_001493063.1	Outer membrane protein omp1
Rickettsia bellii OSU 85-389	2.60E-153	BamB	YP_001496675.1	WD-40 repeat-containing protein
Rickettsia bellii OSU 85-389	1.90E-018	BamD	YP_001495786.1	DNA uptake lipoprotein
Rickettsia bellii OSU 85-389	2.00E-009	BamE	YP_001496795.1	tmRNA-binding protein
Rickettsia bellii OSU 85-389	9.20E-064	Omp85	YP_001495771.1	Outer membrane protein omp1
Rickettsia bellii RML369-C	2.60E-153	BamB	YP_537395.1	WD-40 repeat-containing protein
Rickettsia bellii RML369-C	1.90E-018	BamD	YP_538272.1	DNA uptake lipoprotein
Rickettsia bellii RML369-C	2.00E-009	BamE	YP_537296.1	tmRNA-binding protein
Rickettsia bellii RML369-C	8.50E-062	Omp85	YP_538287.1	Outer membrane protein omp1
Rickettsia canadensis str. McKiel	5.80E-188	BamB	YP_001491998.1	DNA topoisomerase IV subunit B
Rickettsia canadensis str. McKiel	1.50E-016	BamD	YP_001491916.1	hypothetical protein A1E_00915
Rickettsia canadensis str. McKiel	8.40E-009	BamE	YP_001492693.1	hypothetical protein A1E_04960
Rickettsia canadensis str. McKiel	2.80E-073	Omp85	YP_001491896.1	Outer membrane protein omp1
Rickettsia conorii str. Malish 7	5.80E-222	BamB	NP_359951.1	hypothetical protein RC0314
Rickettsia conorii str. Malish 7	2.70E-017	BamD	NP_359867.1	hypothetical protein RC0230
Rickettsia conorii str. Malish 7	8.70E-010	BamE	NP_360841.1	hypothetical protein RC1204
Rickettsia conorii str. Malish 7	1.10E-078	Omp85	NP_359839.1	outer membrane protein omp1
Rickettsia felis URRWXCal2	9.10E-212	BamB	YP_247009.1	WD-40 repeat-containing protein
Rickettsia felis URRWXCal2	3.60E-017	BamD	YP_247104.1	hypothetical protein RF_1088
Rickettsia felis URRWXCal2	3.20E-010	BamE	YP_247253.1	hypothetical protein RF_1237
Rickettsia felis URRWXCal2	1.60E-083	Omp85	YP_247137.1	Outer membrane protein omp1

alpha_e-5

Rickettsia heilongjiangensis 054	1.80E-217	BamB	YP_004764033.1	WD-40 repeat-containing protein
Rickettsia heilongjiangensis 054	2.20E-017	BamD	YP_004763949.1	DNA uptake lipoprotein
Rickettsia heilongjiangensis 054	9.60E-010	BamE	YP_004764847.1	tmRNA-binding protein
Rickettsia heilongjiangensis 054	3.10E-080	Omp85	YP_004763921.1	outer membrane protein omp1
Rickettsia massiliae MTU5	1.20E-216	BamB	YP_001499123.1	WD-40 repeat-containing protein
Rickettsia massiliae MTU5	6.60E-018	BamD	YP_001499072.1	DNA uptake lipoprotein
Rickettsia massiliae MTU5	5.00E-010	BamE	YP_001499748.1	tmRNA-binding protein
Rickettsia massiliae MTU5	3.20E-080	Omp85	YP_001499051.1	Outer membrane protein omp1
Rickettsia peacockii str. Rustic	2.30E-217	BamB	YP_002916258.1	PQQ repeat-containing protein
Rickettsia peacockii str. Rustic	2.20E-017	BamD	YP_002916311.1	ComL family lipoprotein
Rickettsia peacockii str. Rustic	1.30E-009	BamE	YP_002916439.1	SmpA/OmlA family lipoprotein
Rickettsia peacockii str. Rustic	3.10E-080	Omp85	YP_002916206.1	outer membrane protein assembly factor
Rickettsia prowazekii str. Madrid E	6.50E-168	BamB	NP_220618.1	hypothetical protein RP232
Rickettsia prowazekii str. Madrid E	1.70E-017	BamD	NP_220572.1	hypothetical protein RP183
Rickettsia prowazekii str. Madrid E	8.30E-009	BamE	NP_221125.1	hypothetical protein RP774
Rickettsia prowazekii str. Madrid E	1.20E-082	Omp85	NP_220550.1	outer membrane protein OMP1 (omp1)
Rickettsia rickettsii str. 'Sheila Smith'	6.30E-217	BamB	YP_001494432.1	hypothetical protein A1G_01790
Rickettsia rickettsii str. 'Sheila Smith'	3.20E-017	BamD	YP_001494351.1	hypothetical protein A1G_01315
Rickettsia rickettsii str. 'Sheila Smith'	8.70E-010	BamE	YP_001495277.1	hypothetical protein A1G_06580
Rickettsia rickettsii str. 'Sheila Smith'	1.00E-079	Omp85	YP_001494320.1	outer membrane protein omp1
Rickettsia rickettsii str. Iowa	6.30E-217	BamB	YP_001649681.1	PQQ enzyme repeat family protein
Rickettsia rickettsii str. Iowa	3.20E-017	BamD	YP_001649600.1	ComL family lipoprotein
Rickettsia rickettsii str. Iowa	8.70E-010	BamE	YP_001650548.1	SmpA/OmlA family lipoprotein
Rickettsia rickettsii str. Iowa	1.00E-079	Omp85	YP_001649567.1	outer membrane protein assembly factor
Rickettsia typhi str. Wilmington	3.50E-166	BamB	YP_067188.1	hypothetical protein RT0224
Rickettsia typhi str. Wilmington	6.50E-018	BamD	YP_067140.1	lipoprotein
Rickettsia typhi str. Wilmington	6.60E-009	BamE	YP_067699.1	hypothetical protein RT0761
Rickettsia typhi str. Wilmington	2.80E-084	Omp85	YP_067116.1	outer membrane protein Omp1
Roseobacter denitrificans OCh 114	9.60E-021	BamB	YP_682086.1	PQQ repeat-containing protein
Roseobacter denitrificans OCh 114	4.00E-005	BamB	YP_681244.1	methanol dehydrogenase, large subunit
Roseobacter denitrificans OCh 114	5.80E-017	BamD	YP_683530.1	competence lipoprotein ComL, putative
Roseobacter denitrificans OCh 114	4.70E-011	BamE	YP_683345.1	putative lipoprotein
Roseobacter denitrificans OCh 114	1.30E-179	Omp85	YP_682830.1	outer membrane protein, putative
Roseobacter denitrificans OCh 114	7.80E-005	Omp85	YP_682591.1	outer membrane protein, putative
Roseobacter litoralis Och 149	1.60E-022	BamB	YP_004691856.1	quinoprotein
Roseobacter litoralis Och 149	8.90E-005	BamB	YP_004692646.1	PQQ-dependent dehydrogenase, methanol/ethanol family
Roseobacter litoralis Och 149	6.80E-017	BamD	YP_004691493.1	outer membrane assembly lipoprotein
Roseobacter litoralis Och 149	7.80E-011	BamE	YP_004691313.1	lipoprotein-like protein
Roseobacter litoralis Och 149	9.70E-182	Omp85	YP_004690914.1	outer membrane assembly factor
Roseobacter litoralis Och 149	7.80E-005	Omp85	YP_004691108.1	surface antigen-like protein

alpha_e-5

Ruegeria pomeroyi DSS-3	1.90E-020	BamB	YP_167552.1	PQQ repeat-containing protein
Ruegeria pomeroyi DSS-3	3.30E-008	BamB	YP_166749.1	quinoprotein ethanol dehydrogenase
Ruegeria pomeroyi DSS-3	7.70E-017	BamD	YP_166453.1	competence lipoprotein ComL, putative
Ruegeria pomeroyi DSS-3	2.50E-018	BamE	YP_167706.1	SmpA/OmlA family lipoprotein
Ruegeria pomeroyi DSS-3	0.00E+000	Omp85	YP_166909.1	OMP85 family outer membrane protein
Ruegeria sp. TM1040	3.80E-026	BamB	YP_612985.1	Pyrrolo-quinoline quinone
Ruegeria sp. TM1040	9.90E-017	BamD	YP_612686.1	competence lipoprotein ComL, putative
Ruegeria sp. TM1040	1.10E-011	BamE	YP_612913.1	SmpA/OmlA family lipoprotein
Ruegeria sp. TM1040	9.80E-193	Omp85	YP_613402.1	surface antigen (D15)
Sinorhizobium fredii NGR234	4.50E-008	BamB	YP_002822542.1	alcohol dehydrogenase
Sinorhizobium fredii NGR234	3.60E-005	BamB	YP_002825284.1	glucose dehydrogenase (pyrroloquinoline-quinone) protein
Sinorhizobium fredii NGR234	3.50E-021	BamD	YP_002826615.1	competence lipoprotein ComL
Sinorhizobium fredii NGR234	1.50E-034	BamE	YP_002825511.1	putative outer membrane protein
Sinorhizobium fredii NGR234	1.30E-130	Omp85	YP_002825874.1	group 1 outer membrane protein precursor
Sinorhizobium fredii NGR234	5.10E-007	Omp85	YP_002827566.1	putative cell surface protein
Sinorhizobium medicae WSM419	3.10E-005	BamB	YP_001312686.1	Pyrrolo-quinoline quinone
Sinorhizobium medicae WSM419	7.90E-006	BamB	YP_001314876.1	Pyrrolo-quinoline quinone
Sinorhizobium medicae WSM419	1.50E-005	BamB	YP_001326286.1	quinoprotein glucose dehydrogenase
Sinorhizobium medicae WSM419	2.90E-020	BamD	YP_001327740.1	hypothetical protein Smed_2072
Sinorhizobium medicae WSM419	3.10E-035	BamE	YP_001326516.1	SmpA/OmlA domain-containing protein
Sinorhizobium medicae WSM419	5.90E-128	Omp85	YP_001326824.1	surface antigen (D15)
Sinorhizobium medicae WSM419	1.10E-006	Omp85	YP_001328552.1	surface antigen (D15)
Sinorhizobium meliloti 1021	1.10E-005	BamB	NP_385087.1	glucose dehydrogenase
Sinorhizobium meliloti 1021	5.20E-005	BamB	NP_436713.1	methanol dehydrogenase, large subunit
Sinorhizobium meliloti 1021	3.20E-020	BamD	NP_386272.1	hypothetical protein SMc01876
Sinorhizobium meliloti 1021	1.90E-035	BamE	NP_385325.1	hypothetical protein SMc01781
Sinorhizobium meliloti 1021	2.20E-122	Omp85	NP_385608.1	outer membrane transmembrane protein
Sinorhizobium meliloti 1021	1.90E-006	Omp85	NP_387102.1	hypothetical protein SMc03097
Sinorhizobium meliloti AK83	1.10E-005	BamB	YP_004548064.1	glucose/quinate/shikimate family membrane-bound PQQ-dependent dehydrogenase
Sinorhizobium meliloti AK83	5.20E-005	BamB	YP_004556573.1	PQQ-dependent dehydrogenase methanol/ethanol family protein
Sinorhizobium meliloti AK83	3.20E-020	BamD	YP_004549480.1	outer membrane assembly lipoprotein YfiO
Sinorhizobium meliloti AK83	1.90E-035	BamE	YP_004548382.1	SmpA/OmlA domain-containing protein
Sinorhizobium meliloti AK83	2.20E-122	Omp85	YP_004548666.1	outer membrane protein assembly complex YaeT protein
Sinorhizobium meliloti AK83	8.40E-007	Omp85	YP_004550371.1	surface antigen (D15)
Sphingobium chlorophenolicum L-1	3.50E-068	BamB	YP_004552265.1	pyrrolo-quinoline quinone repeat-containing protein
Sphingobium chlorophenolicum L-1	3.30E-008	BamB	YP_004552804.1	PQQ-dependent dehydrogenase methanol/ethanol family protein
Sphingobium chlorophenolicum L-1	8.30E-008	BamB	YP_004552793.1	alcohol dehydrogenase (acceptor)
Sphingobium chlorophenolicum L-1	4.70E-006	BamB	YP_004554303.1	PQQ-dependent dehydrogenase methanol/ethanol family protein

Sphingobium chlorophenolicum L-1	2.40E-005	BamB	YP_004555392.1	glucose/quinate/shikimate family membrane-bound PQQ-dependent dehydrogenase
Sphingobium chlorophenolicum L-1	4.80E-005	BamB	YP_004555381.1	glucose/quinate/shikimate family membrane-bound PQQ-dependent dehydrogenase
Sphingobium chlorophenolicum L-1	2.70E-019	BamD	YP_004553440.1	outer membrane assembly lipoprotein YfiO
Sphingobium chlorophenolicum L-1	3.10E-015	BamE	YP_004554342.1	SmpA/OmlA domain-containing protein
Sphingobium chlorophenolicum L-1	8.80E-005	BamE	YP_004554582.1	putative lipoprotein
Sphingobium chlorophenolicum L-1	1.80E-120	Omp85	YP_004553525.1	outer membrane protein assembly complex YaeT protein
Sphingobium chlorophenolicum L-1	2.10E-007	Omp85	YP_004552458.1	surface antigen (D15)
Sphingobium chlorophenolicum L-1	2.60E-006	Omp85	YP_004553975.1	surface antigen (D15)
Sphingobium japonicum UT26S	1.40E-066	BamB	YP_003545944.1	putative PQQ enzyme
Sphingobium japonicum UT26S	4.00E-009	BamB	YP_003543647.1	alcohol dehydrogenase large subunit
Sphingobium japonicum UT26S	5.60E-007	BamB	YP_003543653.1	quinohemoprotein alcohol dehydrogenase
Sphingobium japonicum UT26S	1.30E-007	BamB	YP_003559668.1	putative PQQ-linked alcohol dehydrogenase
Sphingobium japonicum UT26S	1.30E-007	BamB	YP_003559495.1	putative alcohol dehydrogenase
Sphingobium japonicum UT26S	4.20E-006	BamB	YP_003559193.1	quinoprotein glucose dehydrogenase
Sphingobium japonicum UT26S	3.60E-005	BamB	YP_003559189.1	glucose dehydrogenase
Sphingobium japonicum UT26S	9.50E-020	BamD	YP_003545074.1	putative lipoprotein
Sphingobium japonicum UT26S	5.90E-015	BamE	YP_003546914.1	putative small protein A
Sphingobium japonicum UT26S	2.30E-123	Omp85	YP_003545854.1	outer membrane protein YaeT
Sphingobium japonicum UT26S	2.30E-007	Omp85	YP_003544724.1	outer membrane protein YtfM
Sphingobium japonicum UT26S	9.90E-007	Omp85	YP_003546660.1	putative outer membrane protein
Sphingomonas wittichii RW1	2.30E-007	BamB	YP_001260014.1	Pyrrolo-quinoline quinone
Sphingomonas wittichii RW1	2.10E-071	BamB	YP_001263612.1	pyrrolo-quinoline quinone
Sphingomonas wittichii RW1	7.80E-010	BamB	YP_001261506.1	pyrrolo-quinoline quinone
Sphingomonas wittichii RW1	2.00E-008	BamB	YP_001261198.1	pyrrolo-quinoline quinone
Sphingomonas wittichii RW1	2.00E-007	BamB	YP_001264638.1	pyrrolo-quinoline quinone
Sphingomonas wittichii RW1	2.10E-007	BamB	YP_001261226.1	pyrrolo-quinoline quinone
Sphingomonas wittichii RW1	6.10E-007	BamB	YP_001262724.1	pyrrolo-quinoline quinone
Sphingomonas wittichii RW1	1.00E-006	BamB	YP_001261826.1	pyrrolo-quinoline quinone
Sphingomonas wittichii RW1	3.80E-006	BamB	YP_001261943.1	pyrrolo-quinoline quinone
Sphingomonas wittichii RW1	7.40E-006	BamB	YP_001262398.1	quinoprotein glucose dehydrogenase
Sphingomonas wittichii RW1	9.30E-006	BamB	YP_001264733.1	pyrrolo-quinoline quinone
Sphingomonas wittichii RW1	4.80E-005	BamB	YP_001262521.1	quinoprotein glucose dehydrogenase
Sphingomonas wittichii RW1	2.20E-019	BamD	YP_001261959.1	DNA uptake lipoprotein-like protein
Sphingomonas wittichii RW1	1.70E-016	BamE	YP_001262429.1	SmpA/OmlA domain-containing protein
Sphingomonas wittichii RW1	7.00E-106	Omp85	YP_001260974.1	surface antigen (D15)
Sphingomonas wittichii RW1	1.20E-007	Omp85	YP_001265302.1	surface antigen (D15)
Sphingopyxis alaskensis RB2256	2.60E-066	BamB	YP_617246.1	Pyrrolo-quinoline quinone
Sphingopyxis alaskensis RB2256	3.10E-008	BamB	YP_615435.1	Pyrrolo-quinoline quinone
Sphingopyxis alaskensis RB2256	1.90E-020	BamD	YP_615599.1	DNA uptake lipoprotein

Sphingopyxis alaskensis RB2256	1.90E-012	BamE	YP_617831.1	tmRNA-binding small protein A
Sphingopyxis alaskensis RB2256	2.50E-116	Omp85	YP_616997.1	surface antigen (D15)
Sphingopyxis alaskensis RB2256	4.30E-007	Omp85	YP_616608.1	surface antigen (D15)
Starkeya novella DSM 506	3.20E-006	BamB	YP_003696076.1	PQQ-dependent dehydrogenase
Starkeya novella DSM 506	7.00E-018	BamD	YP_003695029.1	outer membrane assembly lipoprotein YfiO
Starkeya novella DSM 506	1.40E-005	BamD	YP_003694931.1	tol-pal system protein YbgF
Starkeya novella DSM 506	2.80E-027	BamE	YP_003693585.1	SmpA/OmlA domain-containing protein
Starkeya novella DSM 506	1.20E-123	Omp85	YP_003693754.1	outer membrane protein assembly complex, YaeT protein
Starkeya novella DSM 506	6.80E-007	Omp85	YP_003692773.1	surface antigen (D15)
Wolbachia endosymbiont of Culex quinquefasciatus Pel	4.40E-013	BamB	YP_001974919.1	PQQ enzyme repeat family protein
Wolbachia endosymbiont of Culex quinquefasciatus Pel	1.20E-010	BamD	YP_001975948.1	competence lipoprotein ComL, putative
Wolbachia endosymbiont of Culex quinquefasciatus Pel	1.20E-010	BamE	YP_001974855.1	Putative lipoprotein
Wolbachia endosymbiont of Culex quinquefasciatus Pel	3.20E-031	Omp85	YP_001975782.1	surface antigen
Wolbachia endosymbiont of Drosophila melanogaster	3.50E-013	BamB	NP_966506.1	PQQ repeat-containing protein
Wolbachia endosymbiont of Drosophila melanogaster	2.00E-011	BamD	NP_966268.1	competence lipoprotein ComL, putative
Wolbachia endosymbiont of Drosophila melanogaster	2.50E-011	BamE	NP_966024.1	putative lipoprotein
Wolbachia endosymbiont of Drosophila melanogaster	6.20E-046	Omp85	NP_966806.1	surface antigen
Wolbachia endosymbiont strain TRS of Brugia malayi	4.40E-013	BamD	YP_198501.1	DNA uptake lipoprotein
Wolbachia endosymbiont strain TRS of Brugia malayi	8.80E-010	BamE	YP_198099.1	small protein A, tmRNA-binding
Wolbachia endosymbiont strain TRS of Brugia malayi	9.70E-034	Omp85	YP_197888.1	Outer membrane protein/protective antigen OMA87
Wolbachia sp. wRi	2.80E-013	BamB	YP_002727270.1	PQQ enzyme repeat family protein
Wolbachia sp. wRi	5.00E-009	BamB	YP_002727727.1	PQQ enzyme repeat family protein
Wolbachia sp. wRi	1.20E-010	BamD	YP_002726874.1	competence lipoprotein ComL, putative
Wolbachia sp. wRi	2.50E-011	BamE	YP_002726828.1	small protein A, tmRNA-binding
Wolbachia sp. wRi	5.90E-049	Omp85	YP_002727606.1	surface antigen
Xanthobacter autotrophicus Py2	3.50E-005	BamB	YP_001419563.1	Pyrrolo-quinoline quinone
Xanthobacter autotrophicus Py2	5.50E-005	BamB	YP_001416683.1	Pyrrolo-quinoline quinone
Xanthobacter autotrophicus Py2	2.00E-017	BamD	YP_001415245.1	putative lipoprotein
Xanthobacter autotrophicus Py2	1.80E-030	BamE	YP_001419259.1	SmpA/OmlA domain-containing protein
Xanthobacter autotrophicus Py2	8.40E-115	Omp85	YP_001419309.1	surface antigen (D15)
Xanthobacter autotrophicus Py2	1.00E-006	Omp85	YP_001419102.1	surface antigen (D15)
Zymomonas mobilis subsp. mobilis NCIMB 11163	4.40E-047	BamB	YP_003225928.1	pyrrolo-quinoline quinone
Zymomonas mobilis subsp. mobilis NCIMB 11163	3.00E-018	BamD	YP_003226588.1	outer membrane assembly lipoprotein YfiO
Zymomonas mobilis subsp. mobilis NCIMB 11163	1.10E-015	BamE	YP_003226362.1	SmpA/OmlA domain-containing protein
Zymomonas mobilis subsp. mobilis NCIMB 11163	1.80E-135	Omp85	YP_003225316.1	outer membrane protein assembly complex, YaeT protein
Zymomonas mobilis subsp. mobilis NCIMB 11163	5.70E-007	Omp85	YP_003225292.1	surface antigen (D15)
Zymomonas mobilis subsp. mobilis NCIMB 11163	1.70E-006	Omp85	YP_003225889.1	surface antigen (D15)

Achromobacter xylosoxidans A8	3.10E-092	BamB	YP_003978215.1	outer membrane assembly lipoprotein YfgL
Achromobacter xylosoxidans A8	3.80E-007	BamC	YP_003980625.1	NlpBDapX lipoprotein
Achromobacter xylosoxidans A8	4.00E-094	BamD	YP_003977715.1	competence lipoprotein ComL
Achromobacter xylosoxidans A8	2.30E-017	BamE	YP_003977219.1	outer membrane lipoprotein OmlA domain-containing protein
Achromobacter xylosoxidans A8	1.30E-005	BamE	YP_003980911.1	hypothetical protein AXYL_04888
Achromobacter xylosoxidans A8	2.60E-182	Omp85	YP_003978844.1	outer membrane protein assembly complex, YaeT protein
Achromobacter xylosoxidans A8	6.40E-005	Omp85	YP_003981661.1	surface antigen family protein
Acidovorax avenae subsp. avenae ATCC 19860	3.20E-067	BamB	YP_004233955.1	outer membrane assembly lipoprotein YfgL
Acidovorax avenae subsp. avenae ATCC 19860	4.40E-006	BamB	YP_004232962.1	membrane-bound PQQ-dependent dehydrogenase
Acidovorax avenae subsp. avenae ATCC 19860	2.70E-007	BamC	YP_004233841.1	NlpBDapX family lipoprotein
Acidovorax avenae subsp. avenae ATCC 19860	6.00E-084	BamD	YP_004234488.1	outer membrane assembly lipoprotein YfiO
Acidovorax avenae subsp. avenae ATCC 19860	1.90E-010	BamE	YP_004236924.1	SmpA/OmlA domain-containing protein
Acidovorax avenae subsp. avenae ATCC 19860	3.80E-005	BamE	YP_004237075.1	putative lipoprotein transmembrane
Acidovorax avenae subsp. avenae ATCC 19860	2.40E-177	Omp85	YP_004234253.1	outer membrane protein assembly complex, YaeT protein
Acidovorax avenae subsp. avenae ATCC 19860	2.60E-005	Omp85	YP_004236311.1	surface antigen (D15)
Acidovorax citrulli AAC00-1	1.10E-066	BamB	YP_969792.1	Pyrrolo-quinoline quinone
Acidovorax citrulli AAC00-1	9.30E-006	BamB	YP_968792.1	quinoprotein glucose dehydrogenase
Acidovorax citrulli AAC00-1	5.60E-008	BamC	YP_969701.1	NlpB/DapX family lipoprotein
Acidovorax citrulli AAC00-1	4.60E-083	BamD	YP_971577.1	hypothetical protein Aave_3241
Acidovorax citrulli AAC00-1	2.20E-010	BamE	YP_972865.1	SmpA/OmlA domain-containing protein
Acidovorax citrulli AAC00-1	6.10E-005	BamE	YP_972998.1	putative lipoprotein transmembrane
Acidovorax citrulli AAC00-1	4.70E-179	Omp85	YP_970189.1	surface antigen (D15)
Acidovorax citrulli AAC00-1	3.30E-005	Omp85	YP_972272.1	surface antigen (D15)
Acidovorax ebreus TPSY	1.80E-056	BamB	YP_002552570.1	outer membrane assembly lipoprotein yfgl
Acidovorax ebreus TPSY	8.40E-009	BamC	YP_002552401.1	nlpbdapx family lipoprotein
Acidovorax ebreus TPSY	6.20E-084	BamD	YP_002553356.1	hypothetical protein Dtpsy_1899
Acidovorax ebreus TPSY	1.50E-010	BamE	YP_002554700.1	smpa/omla domain-containing protein
Acidovorax ebreus TPSY	2.20E-174	Omp85	YP_002552698.1	outer membrane protein assembly complex, yaet protein
Acidovorax ebreus TPSY	4.30E-005	Omp85	YP_002554317.1	surface antigen (d15)
Acidovorax sp. JS42	1.70E-057	BamB	YP_985481.1	Pyrrolo-quinoline quinone
Acidovorax sp. JS42	1.10E-008	BamC	YP_985323.1	NlpB/DapX family lipoprotein
Acidovorax sp. JS42	6.20E-084	BamD	YP_986092.1	hypothetical protein Ajs_1829
Acidovorax sp. JS42	1.10E-010	BamE	YP_988105.1	SmpA/OmlA domain-containing protein
Acidovorax sp. JS42	4.30E-173	Omp85	YP_986811.1	surface antigen (D15)
Acidovorax sp. JS42	4.10E-005	Omp85	YP_987748.1	surface antigen (D15)
Alicycliphilus denitrificans BC	7.70E-054	BamB	YP_004127647.1	outer membrane assembly lipoprotein yfgl
Alicycliphilus denitrificans BC	1.10E-007	BamC	YP_004125753.1	nlpb/dapx family lipoprotein
Alicycliphilus denitrificans BC	5.40E-083	BamD	YP_004127282.1	outer membrane assembly lipoprotein yfio

Alicycliphilus denitrificans BC	9.90E-012	BamE	YP_004128711.1	smpa/omla domain-containing protein
Alicycliphilus denitrificans BC	6.20E-005	BamE	YP_004126994.1	trap dicarboxylate transporter, dctp subunit
Alicycliphilus denitrificans BC	6.70E-005	BamE	YP_004128859.1	lipoprotein transmembrane
Alicycliphilus denitrificans BC	1.80E-174	Omp85	YP_004126120.1	outer membrane protein assembly complex, yaet protein
Alicycliphilus denitrificans K601	7.70E-054	BamB	YP_004387330.1	outer membrane assembly lipoprotein YfgL
Alicycliphilus denitrificans K601	1.10E-007	BamC	YP_004389627.1	NlpBDapX family lipoprotein
Alicycliphilus denitrificans K601	5.40E-083	BamD	YP_004388882.1	outer membrane assembly lipoprotein YfiO
Alicycliphilus denitrificans K601	9.90E-012	BamE	YP_004390293.1	SmpA/OmlA domain-containing protein
Alicycliphilus denitrificans K601	1.80E-174	Omp85	YP_004389308.1	outer membrane protein assembly complex, YaeT protein
Alicycliphilus denitrificans K601	7.80E-005	Omp85	YP_004389750.1	surface antigen (D15)
Aromatoleum aromaticum EbN1	6.30E-110	BamB	YP_157696.1	putative quinoprotein
Aromatoleum aromaticum EbN1	3.70E-008	BamC	YP_157423.1	hypothetical protein ebA773
Aromatoleum aromaticum EbN1	6.80E-105	BamD	YP_159693.1	putative competence lipoprotein precursor
Aromatoleum aromaticum EbN1	3.60E-005	BamE	YP_159639.1	hypothetical protein ebB157
Aromatoleum aromaticum EbN1	2.40E-229	Omp85	YP_160448.1	outer membrane protein/surface antigen
Aromatoleum aromaticum EbN1	1.60E-005	Omp85	YP_157653.1	hypothetical protein ebA1189
Azoarcus sp. BH72	6.50E-108	BamB	YP_932434.1	hypothetical protein azo0930
Azoarcus sp. BH72	3.30E-008	BamB	YP_934478.1	putative quinoprotein ethanol dehydrogenase
Azoarcus sp. BH72	2.30E-007	BamB	YP_934347.1	putative quinoprotein ethanol dehydrogenase
Azoarcus sp. BH72	2.30E-007	BamB	YP_935367.1	putative quinoprotein ethanol dehydrogenase
Azoarcus sp. BH72	6.30E-007	BamB	YP_934525.1	putative quinoprotein ethanol dehydrogenase
Azoarcus sp. BH72	6.90E-006	BamB	YP_934475.1	ExaA2 protein
Azoarcus sp. BH72	6.40E-008	BamC	YP_932601.1	putative lipoprotein
Azoarcus sp. BH72	2.90E-104	BamD	YP_932521.1	competence lipoprotein
Azoarcus sp. BH72	1.60E-030	BamE	YP_934081.1	putative outer membrane lipoprotein OmlA
Azoarcus sp. BH72	8.90E-005	BamE	YP_933733.1	hypothetical protein azo2229
Azoarcus sp. BH72	3.30E-230	Omp85	YP_933405.1	outer membrane protein
Azoarcus sp. BH72	1.10E-005	Omp85	YP_932473.1	outer membrane protein
Azoarcus sp. EbN1	1.70E-007	BamB	YP_195704.1	putative PQQ-dependent polyvinyl alcohol dehydrogenase precursor
Bordetella avium 197N	2.80E-098	BamB	YP_786855.1	lipoprotein
Bordetella avium 197N	1.90E-006	BamC	YP_787098.1	lipoprotein
Bordetella avium 197N	2.60E-098	BamD	YP_785740.1	lipoprotein
Bordetella avium 197N	8.10E-018	BamE	YP_787223.1	outer membrane lipoprotein
Bordetella avium 197N	7.10E-006	BamE	YP_784938.1	hypothetical protein BAV0408
Bordetella avium 197N	8.50E-196	Omp85	YP_786260.1	surface antigen
Bordetella bronchiseptica RB50	2.00E-104	BamB	NP_889709.1	quinoprotein
Bordetella bronchiseptica RB50	8.60E-007	BamC	NP_888202.1	lipoprotein

Bordetella bronchiseptica RB50	3.00E-097	BamD	NP_890134.1	competence lipoprotein precursor
Bordetella bronchiseptica RB50	3.50E-014	BamE	NP_890477.1	outer membrane lipoprotein
Bordetella bronchiseptica RB50	7.60E-183	Omp85	NP_889150.1	surface antigen
Bordetella parapertussis 12822	2.00E-104	BamB	NP_885052.1	quinoprotein
Bordetella parapertussis 12822	4.50E-007	BamC	NP_884508.1	lipoprotein
Bordetella parapertussis 12822	3.00E-097	BamD	NP_885369.1	competence lipoprotein precursor
Bordetella parapertussis 12822	3.50E-014	BamE	NP_885655.1	outer membrane lipoprotein
Bordetella parapertussis 12822	3.50E-181	Omp85	NP_883825.1	surface antigen
Bordetella pertussis Tohama I	2.00E-104	BamB	NP_880844.1	putative quinoprotein
Bordetella pertussis Tohama I	8.30E-007	BamC	NP_880303.1	putative lipoprotein
Bordetella pertussis Tohama I	3.00E-097	BamD	NP_879922.1	competence lipoprotein precursor
Bordetella pertussis Tohama I	3.50E-014	BamE	NP_881135.1	outer membrane lipoprotein
Bordetella pertussis Tohama I	3.90E-184	Omp85	NP_880169.1	surface antigen
Bordetella petrii DSM 12804	1.60E-098	BamB	YP_001630631.1	putative quinoprotein
Bordetella petrii DSM 12804	2.20E-005	BamB	YP_001633262.1	hypothetical protein Bpet4644
Bordetella petrii DSM 12804	4.40E-007	BamC	YP_001632084.1	putative lipoprotein
Bordetella petrii DSM 12804	6.00E-081	BamD	YP_001630454.1	competence lipoprotein precursor
Bordetella petrii DSM 12804	3.60E-017	BamE	YP_001630156.1	outer membrane lipoprotein OmlA
Bordetella petrii DSM 12804	7.70E-183	Omp85	YP_001631137.1	outer membrane protein
Burkholderia ambifaria AMMD	4.20E-113	BamB	YP_773637.1	Pyrrolo-quinoline quinone
Burkholderia ambifaria AMMD	7.50E-009	BamB	YP_778040.1	Pyrrolo-quinoline quinone
Burkholderia ambifaria AMMD	2.80E-007	BamC	YP_774021.1	NlpB/DapX family lipoprotein
Burkholderia ambifaria AMMD	4.50E-007	BamC	YP_773261.1	hypothetical protein Bamb_1369
Burkholderia ambifaria AMMD	1.30E-086	BamD	YP_773622.1	competence lipoprotein ComL
Burkholderia ambifaria AMMD	9.00E-024	BamE	YP_772438.1	SmpA/OmlA domain-containing protein
Burkholderia ambifaria AMMD	2.40E-005	BamE	YP_778122.1	OmpA/MotB domain-containing protein
Burkholderia ambifaria AMMD	9.10E-213	Omp85	YP_773934.1	surface antigen (D15)
Burkholderia ambifaria AMMD	3.10E-006	Omp85	YP_774376.1	surface antigen (D15)
Burkholderia ambifaria MC40-6	9.60E-098	BamB	YP_001808421.1	outer membrane assembly lipoprotein YfgL
Burkholderia ambifaria MC40-6	1.50E-008	BamB	YP_001815885.1	methanol/ethanol family PQQ-dependent dehydrogenase
Burkholderia ambifaria MC40-6	2.80E-007	BamC	YP_001808701.1	NlpB/DapX family lipoprotein
Burkholderia ambifaria MC40-6	5.10E-007	BamC	YP_001808113.1	hypothetical protein BamMC406_1409
Burkholderia ambifaria MC40-6	1.30E-086	BamD	YP_001808406.1	competence lipoprotein ComL
Burkholderia ambifaria MC40-6	9.00E-024	BamE	YP_001807284.1	SmpA/OmlA domain-containing protein
Burkholderia ambifaria MC40-6	1.60E-212	Omp85	YP_001808611.1	outer membrane protein assembly complex, YaeT protein
Burkholderia ambifaria MC40-6	3.10E-006	Omp85	YP_001809048.1	surface antigen (D15)
Burkholderia cenocepacia AU 1054	3.60E-008	BamB	YP_623110.1	Pyrrolo-quinoline quinone
Burkholderia cenocepacia AU 1054	3.40E-115	BamB	YP_626107.1	Pyrrolo-quinoline quinone

Burkholderia cenocepacia AU 1054	4.80E-007	BamC	YP_620886.1	uncharacterized lipoprotein-like
Burkholderia cenocepacia AU 1054	6.60E-007	BamC	YP_625821.1	uncharacterized lipoprotein
Burkholderia cenocepacia AU 1054	2.00E-085	BamD	YP_626121.1	DNA uptake lipoprotein-like
Burkholderia cenocepacia AU 1054	4.40E-024	BamE	YP_620054.1	SmpA/OmlA
Burkholderia cenocepacia AU 1054	5.80E-006	Omp85	YP_621704.1	surface antigen (D15)
Burkholderia cenocepacia AU 1054	2.90E-214	Omp85	YP_625904.1	surface antigen (D15)
Burkholderia cenocepacia HI2424	3.40E-115	BamB	YP_835453.1	Pyrrolo-quinoline quinone
Burkholderia cenocepacia HI2424	3.60E-008	BamB	YP_838752.1	Pyrrolo-quinoline quinone
Burkholderia cenocepacia HI2424	4.80E-007	BamC	YP_835131.1	lipoprotein-like protein
Burkholderia cenocepacia HI2424	6.60E-007	BamC	YP_835738.1	lipoprotein
Burkholderia cenocepacia HI2424	2.00E-085	BamD	YP_835438.1	DNA uptake lipoprotein-like protein
Burkholderia cenocepacia HI2424	4.40E-024	BamE	YP_834296.1	SmpA/OmlA domain-containing protein
Burkholderia cenocepacia HI2424	2.00E-007	BamE	YP_840443.1	lipoprotein
Burkholderia cenocepacia HI2424	2.90E-214	Omp85	YP_835655.1	surface antigen (D15)
Burkholderia cenocepacia HI2424	5.80E-006	Omp85	YP_836082.1	surface antigen (D15)
Burkholderia cenocepacia J2315	3.90E-115	BamB	YP_002231008.1	putative lipoprotein
Burkholderia cenocepacia J2315	1.70E-008	BamB	YP_002234969.1	putative quinoprotein ethanol dehydrogenase precursor
Burkholderia cenocepacia J2315	5.70E-007	BamC	YP_002230623.1	hypothetical protein BCAL1493
Burkholderia cenocepacia J2315	8.50E-007	BamC	YP_002231293.1	putative lipoprotein
Burkholderia cenocepacia J2315	7.20E-086	BamD	YP_002230993.1	putative lipoprotein
Burkholderia cenocepacia J2315	1.10E-024	BamE	YP_002232479.1	putative outer membrane protein
Burkholderia cenocepacia J2315	2.80E-006	BamE	YP_002232189.1	putative lipoprotein
Burkholderia cenocepacia J2315	1.00E-006	BamE	YP_002233733.1	SmpA/OmlA family lipoprotein
Burkholderia cenocepacia J2315	5.10E-214	Omp85	YP_002231209.1	outer membrane protein assembly factor YaeT
Burkholderia cenocepacia J2315	6.10E-006	Omp85	YP_002231752.1	putative outer membrane protein
Burkholderia cenocepacia MC0-3	1.40E-115	BamB	YP_001765116.1	outer membrane assembly lipoprotein YfgL
Burkholderia cenocepacia MC0-3	4.30E-008	BamB	YP_001778774.1	methanol/ethanol family PQQ-dependent dehydrogenase
Burkholderia cenocepacia MC0-3	4.80E-007	BamC	YP_001764759.1	lipoprotein-like protein
Burkholderia cenocepacia MC0-3	6.60E-007	BamC	YP_001765395.1	lipoprotein
Burkholderia cenocepacia MC0-3	2.00E-085	BamD	YP_001765101.1	DNA uptake lipoprotein-like protein
Burkholderia cenocepacia MC0-3	4.40E-024	BamE	YP_001763917.1	SmpA/OmlA domain-containing protein
Burkholderia cenocepacia MC0-3	4.00E-213	Omp85	YP_001765314.1	outer membrane protein assembly complex, YaeT protein
Burkholderia cenocepacia MC0-3	5.80E-006	Omp85	YP_001765727.1	surface antigen (D15)
Burkholderia gladioli BSR3	8.10E-116	BamB	YP_004360869.1	hypothetical protein bgla_1g22860
Burkholderia gladioli BSR3	2.50E-006	BamC	YP_004360386.1	Putative lipoprotein
Burkholderia gladioli BSR3	6.30E-006	BamC	YP_004361334.1	Putative lipoprotein
Burkholderia gladioli BSR3	7.90E-086	BamD	YP_004360854.1	Putative competence lipoprotein ComL
Burkholderia gladioli BSR3	2.50E-007	BamE	YP_004350932.1	lipoprotein

Burkholderia gladioli BSR3	5.40E-005	BamE	YP_004351100.1	hypothetical protein bgla_3p0760
Burkholderia gladioli BSR3	2.70E-025	BamE	YP_004359228.1	Outer membrane lipoprotein, SmpA/OmlA
Burkholderia gladioli BSR3	1.90E-201	Omp85	YP_004350514.1	putative outer membrane antigen
Burkholderia gladioli BSR3	8.40E-208	Omp85	YP_004361136.1	Outer membrane protein, OMP85 family protein
Burkholderia gladioli BSR3	2.50E-006	Omp85	YP_004361695.1	outer membrane protein, OMP85 family
Burkholderia glumae BGR1	1.10E-118	BamB	YP_002911305.1	PQQ enzyme repeat-containing protein
Burkholderia glumae BGR1	3.80E-006	BamC	YP_002911829.1	lipoprotein
Burkholderia glumae BGR1	4.00E-005	BamC	YP_002912220.1	NlpBDapX family lipoprotei
Burkholderia glumae BGR1	2.80E-084	BamD	YP_002911320.1	competence lipoprotein ComL
Burkholderia glumae BGR1	2.90E-007	BamE	YP_002907639.1	Hypothetical protein bglu_3p0120
Burkholderia glumae BGR1	9.70E-026	BamE	YP_002910442.1	outer membrane lipoprotein, SmpA/OmlA
Burkholderia glumae BGR1	2.90E-007	BamE	YP_002913216.1	hypothetical protein bglu_4p0150
Burkholderia glumae BGR1	3.70E-205	Omp85	YP_002911142.1	OMP85 family outer membrane protein
Burkholderia glumae BGR1	5.70E-006	Omp85	YP_002912575.1	OMP85 family outer membrane protein
Burkholderia mallei ATCC 23344	1.10E-115	BamB	YP_103002.1	lipoprotein
Burkholderia mallei ATCC 23344	1.40E-006	BamC	YP_102773.1	lipoprotein
Burkholderia mallei ATCC 23344	2.70E-006	BamC	YP_103297.1	lipoprotein
Burkholderia mallei ATCC 23344	6.10E-087	BamD	YP_102985.1	competence lipoprotein ComL
Burkholderia mallei ATCC 23344	2.00E-024	BamE	YP_104004.1	outer membrane lipoprotein, putatitve
Burkholderia mallei ATCC 23344	9.60E-006	BamE	YP_105939.1	OmpA/SmpA/OmlA family outer membrane lipoprotein
Burkholderia mallei ATCC 23344	1.60E-212	Omp85	YP_103187.1	OMP85 family outer membrane protein
Burkholderia mallei ATCC 23344	3.50E-006	Omp85	YP_102475.1	OMP85 family outer membrane protein
Burkholderia mallei NCTC 10229	4.80E-116	BamB	YP_001026074.1	putative lipoprotein
Burkholderia mallei NCTC 10229	1.40E-006	BamC	YP_001026194.1	putative lipoprotein
Burkholderia mallei NCTC 10229	2.70E-006	BamC	YP_001029079.1	putative lipoprotein
Burkholderia mallei NCTC 10229	6.10E-087	BamD	YP_001026091.1	competence lipoprotein ComL
Burkholderia mallei NCTC 10229	5.10E-006	BamE	YP_001024397.1	OmpA/SmpA/OmlA family outer membrane lipoprotein
Burkholderia mallei NCTC 10229	2.00E-024	BamE	YP_001027217.1	SmpA/OmlA family lipoprotein
Burkholderia mallei NCTC 10229	2.90E-211	Omp85	YP_001029200.1	OMP85 family outer membrane protein
Burkholderia mallei NCTC 10229	7.80E-006	Omp85	YP_001028922.1	OMP85 family outer membrane protein
Burkholderia mallei NCTC 10247	4.80E-116	BamB	YP_001080658.1	putative lipoprotein
Burkholderia mallei NCTC 10247	1.40E-006	BamC	YP_001080536.1	putative lipoprotein
Burkholderia mallei NCTC 10247	2.70E-006	BamC	YP_001080999.1	putative lipoprotein
Burkholderia mallei NCTC 10247	6.10E-087	BamD	YP_001080639.1	competence lipoprotein ComL
Burkholderia mallei NCTC 10247	5.10E-006	BamE	YP_001078178.1	OmpA/SmpA/OmlA family outer membrane lipoprotein
Burkholderia mallei NCTC 10247	2.00E-024	BamE	YP_001082169.1	SmpA/OmlA family lipoprotein
Burkholderia mallei NCTC 10247	2.90E-211	Omp85	YP_001080870.1	OMP85 family outer membrane protein
Burkholderia mallei NCTC 10247	7.80E-006	Omp85	YP_001081165.1	OMP85 family outer membrane protein

Burkholderia mallei SAVP1	4.80E-116	BamB	YP_993151.1	putative lipoprotein
Burkholderia mallei SAVP1	1.40E-006	BamC	YP_992852.1	putative lipoprotein
Burkholderia mallei SAVP1	2.70E-006	BamC	YP_993491.1	putative lipoprotein
Burkholderia mallei SAVP1	6.10E-087	BamD	YP_993133.1	competence lipoprotein ComL
Burkholderia mallei SAVP1	5.10E-006	BamE	YP_989916.1	OmpA/SmpA/OmlA family outer membrane lipoprotein
Burkholderia mallei SAVP1	2.00E-024	BamE	YP_991724.1	SmpA/OmlA family lipoprotein
Burkholderia mallei SAVP1	2.90E-211	Omp85	YP_993363.1	OMP85 family outer membrane protein
Burkholderia mallei SAVP1	7.80E-006	Omp85	YP_993617.1	OMP85 family outer membrane protein
Burkholderia multivorans ATCC 17616	6.80E-118	BamB	YP_001579651.1	outer membrane assembly lipoprotein YfgL
Burkholderia multivorans ATCC 17616	1.70E-008	BamB	YP_001585922.1	methanol/ethanol family PQQ-dependent dehydrogenase
Burkholderia multivorans ATCC 17616	1.70E-008	BamB	YP_001941374.1	putative glucose dehydrogenase
Burkholderia multivorans ATCC 17616	6.80E-118	BamB	YP_001946233.1	putative lipoprotein
Burkholderia multivorans ATCC 17616	4.90E-007	BamC	YP_001579367.1	lipoprotein
Burkholderia multivorans ATCC 17616	1.80E-006	BamC	YP_001579955.1	lipoprotein
Burkholderia multivorans ATCC 17616	4.90E-007	BamC	YP_001946519.1	lipoprotein-34
Burkholderia multivorans ATCC 17616	1.80E-006	BamC	YP_001945937.1	lipoprotein-34
Burkholderia multivorans ATCC 17616	6.60E-089	BamD	YP_001579665.1	competence lipoprotein ComL
Burkholderia multivorans ATCC 17616	6.60E-089	BamD	YP_001946218.1	putative lipoprotein
Burkholderia multivorans ATCC 17616	1.90E-025	BamE	YP_001580917.1	SmpA/OmlA domain-containing protein
Burkholderia multivorans ATCC 17616	7.40E-007	BamE	YP_001580650.1	putative lipoprotein
Burkholderia multivorans ATCC 17616	3.40E-005	BamE	YP_001583501.1	OmpA/MotB domain-containing protein
Burkholderia multivorans ATCC 17616	1.90E-025	BamE	YP_001945004.1	small protein A
Burkholderia multivorans ATCC 17616	3.40E-005	BamE	YP_001949374.1	putative OmpA family transmembrane protein
Burkholderia multivorans ATCC 17616	4.30E-216	Omp85	YP_001579450.1	outer membrane protein assembly complex, YaeT protein
Burkholderia multivorans ATCC 17616	3.50E-006	Omp85	YP_001579046.1	surface antigen (D15)
Burkholderia multivorans ATCC 17616	4.30E-216	Omp85	YP_001946435.1	outer membrane protein
Burkholderia multivorans ATCC 17616	3.50E-006	Omp85	YP_001946833.1	outer membrane protein
Burkholderia phymatum STM815	3.30E-121	BamB	YP_001857645.1	outer membrane assembly lipoprotein YfgL
Burkholderia phymatum STM815	5.60E-009	BamB	YP_001863238.1	methanol/ethanol family PQQ-dependent dehydrogenase
Burkholderia phymatum STM815	3.80E-005	BamB	YP_001862554.1	methanol/ethanol family PQQ-dependent dehydrogenase
Burkholderia phymatum STM815	8.00E-007	BamC	YP_001857654.1	putative lipoprotein
Burkholderia phymatum STM815	1.40E-005	BamC	YP_001857751.1	putative exported lipoprotein
Burkholderia phymatum STM815	7.80E-086	BamD	YP_001857201.1	putative competence lipoprotein, ComL
Burkholderia phymatum STM815	6.10E-024	BamE	YP_001858810.1	SmpA/OmlA domain-containing protein
Burkholderia phymatum STM815	9.70E-005	BamE	YP_001856468.1	hypothetical protein Bphy_0229
Burkholderia phymatum STM815	3.40E-211	Omp85	YP_001857558.1	outer membrane protein assembly complex, YaeT protein
Burkholderia phymatum STM815	6.50E-006	Omp85	YP_001858415.1	surface antigen (D15)
Burkholderia phymatum STM815	1.30E-195	Omp85	YP_001859680.1	outer membrane protein assembly complex, YaeT protein

Burkholderia phytofirmans PsJN	1.30E-122	BamB	YP_001896157.1	outer membrane assembly lipoprotein YfgL
Burkholderia phytofirmans PsJN	6.00E-007	BamC	YP_001896166.1	lipoprotein
Burkholderia phytofirmans PsJN	5.50E-006	BamC	YP_001896295.1	exported lipoprotein
Burkholderia phytofirmans PsJN	1.00E-089	BamD	YP_001895472.1	outer membrane assembly lipoprotein YfiO
Burkholderia phytofirmans PsJN	7.40E-005	BamD	YP_001894404.1	tol-pal system protein YbgF
Burkholderia phytofirmans PsJN	2.90E-019	BamE	YP_001896999.1	SmpA/OmlA domain-containing protein
Burkholderia phytofirmans PsJN	9.60E-210	Omp85	YP_001896067.1	outer membrane protein assembly complex, YaeT protein
Burkholderia phytofirmans PsJN	7.00E-205	Omp85	YP_001895743.1	outer membrane protein assembly complex, YaeT protein
Burkholderia phytofirmans PsJN	7.00E-006	Omp85	YP_001894656.1	surface antigen (D15)
Burkholderia pseudomallei 1106a	2.90E-117	BamB	YP_001066489.1	PQQ repeat-containing protein
Burkholderia pseudomallei 1106a	1.50E-006	BamC	YP_001066018.1	putative lipoprotein
Burkholderia pseudomallei 1106a	1.70E-006	BamC	YP_001066871.1	putative lipoprotein
Burkholderia pseudomallei 1106a	4.30E-087	BamD	YP_001066470.1	putative competence lipoprotein ComL
Burkholderia pseudomallei 1106a	2.00E-024	BamE	YP_001067691.1	SmpA/OmlA family lipoprotein
Burkholderia pseudomallei 1106a	5.10E-006	BamE	YP_001075283.1	ompA family protein
Burkholderia pseudomallei 1106a	6.50E-211	Omp85	YP_001066743.1	OMP85 family outer membrane protein
Burkholderia pseudomallei 1106a	2.70E-006	Omp85	YP_001065333.1	OMP85 family outer membrane protein
Burkholderia pseudomallei 1710b	2.90E-117	BamB	YP_333746.1	PQQ repeat-containing protein
Burkholderia pseudomallei 1710b	1.50E-006	BamC	YP_333304.1	hypothetical protein BURPS1710b_1905
Burkholderia pseudomallei 1710b	1.70E-006	BamC	YP_334084.1	putative lipoprotein
Burkholderia pseudomallei 1710b	4.30E-087	BamD	YP_333729.1	competence lipoprotein ComL
Burkholderia pseudomallei 1710b	3.30E-017	BamE	YP_334827.1	outer membrane lipoprotein
Burkholderia pseudomallei 1710b	2.50E-212	Omp85	YP_333967.1	OMP85 family outer membrane protein
Burkholderia pseudomallei 1710b	1.00E-005	Omp85	YP_332617.1	OMP85 family outer membrane protein
Burkholderia pseudomallei 668	3.70E-117	BamB	YP_001059222.1	PQQ repeat-containing protein
Burkholderia pseudomallei 668	1.50E-006	BamC	YP_001058765.1	putative lipoprotein
Burkholderia pseudomallei 668	1.70E-006	BamC	YP_001059590.1	putative lipoprotein
Burkholderia pseudomallei 668	6.30E-087	BamD	YP_001059204.1	putative competence lipoprotein ComL
Burkholderia pseudomallei 668	2.00E-024	BamE	YP_001060430.1	SmpA/OmlA family lipoprotein
Burkholderia pseudomallei 668	5.10E-006	BamE	YP_001062318.1	ompA family protein
Burkholderia pseudomallei 668	2.50E-212	Omp85	YP_001059460.1	OMP85 family outer membrane protein
Burkholderia pseudomallei 668	2.80E-006	Omp85	YP_001058096.2	OMP85 family outer membrane protein
Burkholderia pseudomallei K96243	4.80E-116	BamB	YP_108136.1	hypothetical protein BPSL1516
Burkholderia pseudomallei K96243	1.50E-006	BamC	YP_108532.1	hypothetical protein BPSL1933
Burkholderia pseudomallei K96243	1.70E-006	BamC	YP_108853.1	putative lipoprotein
Burkholderia pseudomallei K96243	2.30E-086	BamD	YP_108151.1	putative lipoprotein
Burkholderia pseudomallei K96243	2.00E-024	BamE	YP_109536.1	putative outer membrane protein
Burkholderia pseudomallei K96243	5.10E-006	BamE	YP_110918.1	OmpA family protein

Burkholderia pseudomallei K96243	3.60E-212	Omp85	YP_108746.1	putative outer membrane protein
Burkholderia pseudomallei K96243	2.80E-006	Omp85	YP_107622.1	hypothetical protein BPSL0994
Burkholderia rhizoxinica HKI 454	4.40E-114	BamB	YP_004029018.1	PQQ enzyme repeat family protein
Burkholderia rhizoxinica HKI 454	1.10E-006	BamC	YP_004029089.1	hypothetical protein RBRH_03658
Burkholderia rhizoxinica HKI 454	8.30E-006	BamC	YP_004029602.1	hypothetical protein RBRH_03861
Burkholderia rhizoxinica HKI 454	2.80E-089	BamD	YP_004029033.1	lipoprotein, ComL family
Burkholderia rhizoxinica HKI 454	2.20E-005	BamD	YP_004029704.1	Tol system periplasmic component YbgF
Burkholderia rhizoxinica HKI 454	1.40E-019	BamE	YP_004028057.1	outer membrane lipoprotein OmlA
Burkholderia rhizoxinica HKI 454	2.00E-216	Omp85	YP_004029174.1	outer membrane protein assembly factor yaeT
Burkholderia rhizoxinica HKI 454	1.60E-005	Omp85	YP_004028280.1	outer membrane protein
Burkholderia sp. 383	4.30E-112	BamB	YP_369348.1	Pyrrolo-quinoline quinone
Burkholderia sp. 383	1.10E-007	BamC	YP_368866.1	uncharacterized lipoprotein-like
Burkholderia sp. 383	2.30E-006	BamC	YP_369638.1	uncharacterized lipoprotein
Burkholderia sp. 383	2.10E-084	BamD	YP_369333.1	DNA uptake lipoprotein-like
Burkholderia sp. 383	1.10E-023	BamE	YP_367981.1	outer membrane lipoprotein, SmpA/OmlA
Burkholderia sp. 383	3.10E-214	Omp85	YP_369559.1	surface antigen (D15)
Burkholderia sp. 383	4.60E-006	Omp85	YP_370008.1	surface antigen (D15)
Burkholderia sp. CCGE1001	1.60E-123	BamB	YP_004228728.1	outer membrane assembly lipoprotein YfgL
Burkholderia sp. CCGE1001	6.90E-009	BamB	YP_004231421.1	PQQ-dependent dehydrogenase
Burkholderia sp. CCGE1001	1.20E-005	BamB	YP_004230414.1	PQQ-dependent dehydrogenase
Burkholderia sp. CCGE1001	8.60E-005	BamB	YP_004231815.1	membrane-bound PQQ-dependent dehydrogenase, glucose/quinate/shikimate family
Burkholderia sp. CCGE1001	1.50E-006	BamC	YP_004228737.1	lipoprotein
Burkholderia sp. CCGE1001	2.20E-005	BamC	YP_004228850.1	exported lipoprotein
Burkholderia sp. CCGE1001	6.00E-091	BamD	YP_004228288.1	outer membrane assembly lipoprotein YfiO
Burkholderia sp. CCGE1001	7.70E-005	BamD	YP_004226989.1	tol-pal system protein YbgF
Burkholderia sp. CCGE1001	5.60E-023	BamE	YP_004229501.1	SmpA/OmlA domain-containing protein
Burkholderia sp. CCGE1001	1.70E-210	Omp85	YP_004228633.1	outer membrane protein assembly complex protein YaeT
Burkholderia sp. CCGE1001	1.90E-006	Omp85	YP_004227287.1	surface antigen (D15)
Burkholderia sp. CCGE1001	9.10E-199	Omp85	YP_004231961.1	outer membrane protein assembly complex, YaeT protein
Burkholderia sp. CCGE1002	3.00E-125	BamB	YP_003605424.1	outer membrane assembly lipoprotein YfgL
Burkholderia sp. CCGE1002	3.40E-008	BamB	YP_003604859.1	PQQ-dependent dehydrogenase, methanol/ethanol family
Burkholderia sp. CCGE1002	9.00E-005	BamB	YP_003604869.1	hypothetical protein
Burkholderia sp. CCGE1002	5.00E-007	BamB	YP_003608990.1	PQQ-dependent enzyme-like protein
Burkholderia sp. CCGE1002	1.30E-005	BamB	YP_003606784.1	PQQ-dependent dehydrogenase, methanol/ethanol family
Burkholderia sp. CCGE1002	3.70E-006	BamB	YP_003609734.1	membrane-bound PQQ-dependent dehydrogenase, glucose/quinate/shikimate family
Burkholderia sp. CCGE1002	2.40E-006	BamC	YP_003605433.1	lipoprotein
Burkholderia sp. CCGE1002	2.50E-006	BamC	YP_003605540.1	exported lipoprotein

Burkholderia sp. CCGE1002	3.70E-088	BamD	YP_003604996.1	outer membrane assembly lipoprotein YfiO
Burkholderia sp. CCGE1002	3.60E-022	BamE	YP_003606205.1	SmpA/OmlA domain protein
Burkholderia sp. CCGE1002	6.90E-211	Omp85	YP_003605341.1	outer membrane protein assembly complex, YaeT protein
Burkholderia sp. CCGE1002	1.90E-006	Omp85	YP_003604338.1	surface antigen (D15)
Burkholderia sp. CCGE1003	5.10E-124	BamB	YP_003906476.1	outer membrane assembly lipoprotein YfgL
Burkholderia sp. CCGE1003	5.90E-009	BamB	YP_003909716.1	PQQ-dependent dehydrogenase, methanol/ethanol family
Burkholderia sp. CCGE1003	2.60E-005	BamB	YP_003910669.1	PQQ-dependent dehydrogenase, methanol/ethanol family
Burkholderia sp. CCGE1003	3.10E-005	BamB	YP_003909274.1	membrane-bound PQQ-dependent dehydrogenase, glucose/quinate/shikimate family
Burkholderia sp. CCGE1003	1.20E-006	BamC	YP_003906467.1	putative lipoprotein
Burkholderia sp. CCGE1003	2.40E-005	BamC	YP_003906355.1	putative exported lipoprotein
Burkholderia sp. CCGE1003	9.20E-091	BamD	YP_003906814.1	outer membrane assembly lipoprotein YfiO
Burkholderia sp. CCGE1003	4.70E-005	BamD	YP_003905773.1	tol-pal system protein YbgF
Burkholderia sp. CCGE1003	3.60E-023	BamE	YP_003908208.1	SmpA/OmlA domain-containing protein
Burkholderia sp. CCGE1003	1.90E-210	Omp85	YP_003906563.1	outer membrane protein assembly complex, YaeT protein
Burkholderia sp. CCGE1003	2.70E-006	Omp85	YP_003906149.1	surface antigen (D15)
Burkholderia sp. CCGE1003	2.70E-201	Omp85	YP_003910252.1	outer membrane protein assembly complex, YaeT protein
Burkholderia sp. JV3	5.80E-116	BamB	YP_004792262.1	outer membrane assembly lipoprotein YfgL
Burkholderia sp. JV3	2.20E-070	BamD	YP_004793738.1	outer membrane assembly lipoprotein YfiO
Burkholderia sp. JV3	1.40E-020	BamE	YP_004792191.1	SmpA/OmlA domain-containing protein
Burkholderia sp. JV3	2.70E-005	BamE	YP_004792918.1	OmpA/MotB domain-containing protein
Burkholderia sp. JV3	6.40E-005	BamE	YP_004790936.1	EF hand repeat-containing protein
Burkholderia sp. JV3	3.60E-182	Omp85	YP_004791805.1	outer membrane protein assembly complex, YaeT protein
Burkholderia sp. JV3	1.10E-006	Omp85	YP_004794564.1	surface antigen (D15)
Burkholderia thailandensis E264	3.00E-117	BamB	YP_442758.1	lipoprotein
Burkholderia thailandensis E264	5.70E-007	BamC	YP_443095.1	lipoprotein
Burkholderia thailandensis E264	8.90E-007	BamC	YP_442437.1	lipoprotein
Burkholderia thailandensis E264	1.10E-086	BamD	YP_442773.1	competence lipoprotein ComL
Burkholderia thailandensis E264	9.40E-006	BamE	YP_439684.1	OmpA/SmpA/OmlA family outer membrane lipoprotein
Burkholderia thailandensis E264	1.40E-025	BamE	YP_441754.1	outer membrane lipoprotein
Burkholderia thailandensis E264	3.30E-211	Omp85	YP_442559.1	OMP85 family outer membrane protein
Burkholderia thailandensis E264	3.80E-007	Omp85	YP_441407.1	OMP85 family outer membrane protein
Burkholderia vietnamiensis G4	2.70E-008	BamB	YP_001115712.1	Pyrrolo-quinoline quinone
Burkholderia vietnamiensis G4	6.30E-115	BamB	YP_001119575.1	Pyrrolo-quinoline quinone
Burkholderia vietnamiensis G4	6.20E-007	BamC	YP_001119296.1	hypothetical protein Bcep1808_1453
Burkholderia vietnamiensis G4	1.30E-006	BamC	YP_001120010.1	lipoprotein
Burkholderia vietnamiensis G4	6.40E-086	BamD	YP_001119560.1	competence lipoprotein ComL
Burkholderia vietnamiensis G4	8.10E-008	BamE	YP_001110306.1	lipoprotein

Burkholderia vietnamiensis G4	8.00E-024	BamE	YP_001118465.1	SmpA/OmlA domain-containing protein
Burkholderia vietnamiensis G4	2.40E-214	Omp85	YP_001119755.1	surface antigen (D15)
Burkholderia vietnamiensis G4	2.30E-006	Omp85	YP_001120356.1	surface antigen (D15)
Burkholderia xenovorans LB400	7.30E-123	BamB	YP_559410.1	transcription accessory protein, TEX
Burkholderia xenovorans LB400	1.50E-008	BamB	YP_554990.1	putative alcohol dehydrogenase
Burkholderia xenovorans LB400	1.50E-007	BamB	YP_552926.1	putative quinoprotein alcohol dehydrogenase
Burkholderia xenovorans LB400	8.50E-006	BamB	YP_552914.1	methanol dehydrogenase-like protein (xoxF)
Burkholderia xenovorans LB400	8.40E-007	BamC	YP_559421.1	putative lipoprotein
Burkholderia xenovorans LB400	5.80E-088	BamD	YP_558675.1	putative competence lipoprotein, ComL
Burkholderia xenovorans LB400	5.10E-005	BamD	YP_557127.1	putative transmembrane protein
Burkholderia xenovorans LB400	9.30E-020	BamE	YP_560413.1	putative outer membrane lipoprotein
Burkholderia xenovorans LB400	3.50E-005	BamE	YP_554483.1	hypothetical protein Bxe_B0821
Burkholderia xenovorans LB400	1.20E-210	Omp85	YP_559317.1	putative outer membrane antigen
Burkholderia xenovorans LB400	7.10E-006	Omp85	YP_557552.1	hypothetical protein Bxe_A3485
Candidatus Accumulibacter phosphatis clade IIA str. UW-1	4.60E-125	BamB	YP_003167339.1	outer membrane assembly lipoprotein YfgL
Candidatus Accumulibacter phosphatis clade IIA str. UW-1	2.60E-009	BamC	YP_003166626.1	NlpBDapX family lipoprotein
Candidatus Accumulibacter phosphatis clade IIA str. UW-1	6.30E-110	BamD	YP_003169102.1	outer membrane assembly lipoprotein YfiO
Candidatus Accumulibacter phosphatis clade IIA str. UW-1	9.10E-022	BamE	YP_003168917.1	SmpA/OmlA domain-containing protein
Candidatus Accumulibacter phosphatis clade IIA str. UW-1	9.00E-216	Omp85	YP_003168077.1	outer membrane protein assembly complex, YaeT protein
Chromobacterium violaceum ATCC 12472	1.80E-065	BamB	NP_903205.1	hypothetical protein CV_3535
Chromobacterium violaceum ATCC 12472	5.10E-009	BamC	NP_903249.1	hypothetical protein CV_3579
Chromobacterium violaceum ATCC 12472	3.70E-102	BamD	NP_901863.1	competence lipoprotein ComL
Chromobacterium violaceum ATCC 12472	7.00E-022	BamE	NP_901466.1	outer membrane lipoprotein OmlA
Chromobacterium violaceum ATCC 12472	2.80E-249	Omp85	NP_901874.1	outer membrane protein
Chromobacterium violaceum ATCC 12472	4.20E-005	Omp85	NP_900203.1	outer membrane protein
Collimonas fungivorans Ter331	1.10E-123	BamB	YP_004752573.1	putative serine/threonine protein kinase with quinoprotein alcohol dehydrogenase domain
Collimonas fungivorans Ter331	6.40E-007	BamC	YP_004752334.1	putative lipoprotein
Collimonas fungivorans Ter331	2.60E-100	BamD	YP_004752426.1	putative lipoprotein with tetratricopeptide repeats (TPR) domain
Collimonas fungivorans Ter331	8.50E-018	BamE	YP_004754502.1	hypothetical protein CFU_3855
Collimonas fungivorans Ter331	2.50E-006	BamE	YP_004750945.1	outer membrane protein A
Collimonas fungivorans Ter331	4.20E-005	BamE	YP_004754128.1	hypothetical protein CFU_3481
Collimonas fungivorans Ter331	8.10E-204	Omp85	YP_004751994.1	outer membrane protein sensing stress
Collimonas fungivorans Ter331	2.10E-005	Omp85	YP_004751831.1	outer membrane protein
Comamonas testosteroni CNB-2	1.00E-043	BamB	YP_003279327.1	pyrrolo-quinoline quinone
Comamonas testosteroni CNB-2	1.20E-007	BamB	YP_003277430.1	pyrrolo-quinoline quinone
Comamonas testosteroni CNB-2	1.40E-007	BamB	YP_003276239.1	pyrrolo-quinoline quinone
Comamonas testosteroni CNB-2	2.40E-006	BamB	YP_003277128.1	pyrrolo-quinoline quinone

Comamonas testosteroni CNB-2	4.00E-005	BamB	YP_003278852.1	pyrrolo-quinoline quinone
Comamonas testosteroni CNB-2	3.80E-007	BamC	YP_003277199.1	NlpBDapX lipoprotein
Comamonas testosteroni CNB-2	2.20E-075	BamD	YP_003278181.1	transmembrane protein
Comamonas testosteroni CNB-2	2.80E-012	BamE	YP_003280686.1	SmpA/OmlA
Comamonas testosteroni CNB-2	4.60E-183	Omp85	YP_003279266.1	surface antigen (D15)
Cupriavidus metallidurans CH34	3.90E-151	BamB	YP_584251.1	outer membrane protein assembly complex subunit YfgL, pyrrolo-quinoline quinone (PQQ) dependent alcohol dehydrogenase subunit
Cupriavidus metallidurans CH34	1.10E-005	BamC	YP_583224.1	putative lipoprotein
Cupriavidus metallidurans CH34	1.20E-097	BamD	YP_583504.1	DNA uptake lipoprotein
Cupriavidus metallidurans CH34	3.70E-018	BamE	YP_583806.1	putative small protein A (tmRNA-binding)-related protein (SmpA/OmlA fragment)
Cupriavidus metallidurans CH34	1.70E-017	BamE	YP_585117.1	Lipoprotein
Cupriavidus metallidurans CH34	5.90E-006	BamE	YP_584912.1	putative lipoprotein
Cupriavidus metallidurans CH34	1.10E-005	BamE	YP_587836.1	putative Small protein A
Cupriavidus metallidurans CH34	1.90E-233	Omp85	YP_583595.1	outer membrane protein assembly factor,outer membrane protein, surface antigen OMA87
Cupriavidus metallidurans CH34	5.80E-006	Omp85	YP_584914.1	outer membrane protein
Cupriavidus necator N-1	1.80E-007	BamB	YP_004681209.1	quinate dehydrogenase (PQQ)
Cupriavidus necator N-1	7.60E-157	BamB	YP_004686085.1	lipoprotein YfgL
Cupriavidus necator N-1	1.80E-007	BamB	YP_004685515.1	quinoprotein ethanol dehydrogenase ExaA
Cupriavidus necator N-1	3.50E-007	BamB	YP_004685687.1	quinohemoprotein ethanol dehydrogenase type-1
Cupriavidus necator N-1	2.70E-006	BamB	YP_004687893.1	alcohol dehydrogenase
Cupriavidus necator N-1	7.20E-007	BamC	YP_004684989.1	hypothetical protein CNE_1c11530
Cupriavidus necator N-1	5.70E-099	BamD	YP_004685288.1	competence lipoprotein ComL
Cupriavidus necator N-1	2.60E-019	BamE	YP_004686891.1	lipoprotein
Cupriavidus necator N-1	1.90E-005	BamE	YP_004686698.1	hypothetical protein CNE_1c29040
Cupriavidus necator N-1	3.50E-223	Omp85	YP_004685806.1	outer membrane protein surface antigen
Cupriavidus necator N-1	6.60E-006	Omp85	YP_004686700.1	hypothetical protein CNE_1c29060
Cupriavidus taiwanensis LMG 19424	6.00E-158	BamB	YP_002005910.1	hypothetical protein RALTA_A1906
Cupriavidus taiwanensis LMG 19424	3.20E-007	BamB	YP_002005589.1	quinohaemoprotein ethanol dehydrogenase
Cupriavidus taiwanensis LMG 19424	2.20E-007	BamB	YP_002007344.1	quinoprotein ethanol dehydrogenase, pqq dehydrogenase family
Cupriavidus taiwanensis LMG 19424	2.70E-007	BamC	YP_002005213.1	lipoprotein
Cupriavidus taiwanensis LMG 19424	4.70E-101	BamD	YP_002005370.1	hypothetical protein RALTA_A1346
Cupriavidus taiwanensis LMG 19424	1.20E-018	BamE	YP_002006609.1	outer membrane lipoprotein, smpa/omla family
Cupriavidus taiwanensis LMG 19424	3.20E-005	BamE	YP_002006423.1	hypothetical protein RALTA_A2428
Cupriavidus taiwanensis LMG 19424	3.50E-223	Omp85	YP_002005691.1	hypothetical protein RALTA_A1683
Cupriavidus taiwanensis LMG 19424	2.90E-005	Omp85	YP_002006425.1	hypothetical protein RALTA_A2430
Dechloromonas aromatica RCB	2.80E-116	BamB	YP_286182.1	Pyrrolo-quinoline quinone
Dechloromonas aromatica RCB	1.20E-007	BamB	YP_284249.1	Pyrrolo-quinoline quinone

Dechloromonas aromatica RCB	2.40E-007	BamC	YP_284073.1	NlpBDapX lipoprotein
Dechloromonas aromatica RCB	1.90E-096	BamD	YP_284822.1	putative competence lipoprotein precursor
Dechloromonas aromatica RCB	2.70E-019	BamE	YP_284161.1	SmpA/OmlA
Dechloromonas aromatica RCB	1.20E-211	Omp85	YP_284967.1	surface antigen (D15):surface antigen variable number
Delftia acidovorans SPH-1	4.00E-044	BamB	YP_001566030.1	outer membrane assembly lipoprotein YfgL
Delftia acidovorans SPH-1	7.30E-007	BamB	YP_001564375.1	methanol/ethanol family PQQ-dependent dehydrogenase
Delftia acidovorans SPH-1	3.40E-005	BamB	YP_001561373.1	PQQ-dependent dehydrogenase glucose/quinate/shikimate family protein
Delftia acidovorans SPH-1	5.40E-008	BamC	YP_001566104.1	NlpB/DapX family lipoprotein
Delftia acidovorans SPH-1	8.30E-074	BamD	YP_001564521.1	putative transmembrane protein
Delftia acidovorans SPH-1	4.90E-012	BamE	YP_001567021.1	SmpA/OmlA domain-containing protein
Delftia acidovorans SPH-1	3.70E-189	Omp85	YP_001565954.1	outer membrane protein assembly complex protein YaeT
Delftia acidovorans SPH-1	6.90E-005	Omp85	YP_001562686.1	surface antigen (D15)
Delftia sp. Cs1-4	9.40E-044	BamB	YP_004486986.1	outer membrane assembly lipoprotein YfgL
Delftia sp. Cs1-4	1.60E-006	BamB	YP_004488764.1	methanol/ethanol family PQQ-dependent dehydrogenase
Delftia sp. Cs1-4	4.40E-005	BamB	YP_004485750.1	membrane-bound PQQ-dependent dehydrogenase
Delftia sp. Cs1-4	9.20E-008	BamC	YP_004486909.1	NlpBDapX family lipoprotein
Delftia sp. Cs1-4	8.30E-074	BamD	YP_004488671.1	outer membrane assembly lipoprotein YfiO
Delftia sp. Cs1-4	4.90E-012	BamE	YP_004491210.1	SmpA/OmlA domain-containing protein
Delftia sp. Cs1-4	1.20E-188	Omp85	YP_004487286.1	outer membrane protein assembly complex, YaeT protein
Delftia sp. Cs1-4	7.60E-005	Omp85	YP_004490245.1	surface antigen (D15)
Gallionella capsiferriformans ES-2	7.30E-104	BamB	YP_003847181.1	outer membrane assembly lipoprotein YfgL
Gallionella capsiferriformans ES-2	3.90E-008	BamC	YP_003847968.1	NlpBDapX lipoprotein
Gallionella capsiferriformans ES-2	1.70E-093	BamD	YP_003847878.1	outer membrane assembly lipoprotein YfiO
Gallionella capsiferriformans ES-2	5.30E-005	BamD	YP_003847368.1	hypothetical protein Galf_1586
Gallionella capsiferriformans ES-2	3.10E-028	BamE	YP_003847922.1	SmpA/OmlA domain-containing protein
Gallionella capsiferriformans ES-2	3.10E-005	BamE	YP_003846457.1	hypothetical protein Galf_0652
Gallionella capsiferriformans ES-2	1.30E-208	Omp85	YP_003847560.1	outer membrane protein assembly complex, YaeT protein
Herbaspirillum seropedicae SmR1	2.60E-114	BamB	YP_003776346.1	lipoprotein transmembrane protein
Herbaspirillum seropedicae SmR1	1.10E-007	BamC	YP_003776664.1	lipoprotein
Herbaspirillum seropedicae SmR1	2.60E-101	BamD	YP_003776400.1	hypothetical protein Hsero_3004
Herbaspirillum seropedicae SmR1	8.80E-006	BamD	YP_003774640.1	Tol-Pal cell envelope complex subunit YbgF protein
Herbaspirillum seropedicae SmR1	2.50E-011	BamE	YP_003774034.1	small protein A
Herbaspirillum seropedicae SmR1	1.50E-005	BamE	YP_003776482.1	lipoprotein
Herbaspirillum seropedicae SmR1	1.00E-213	Omp85	YP_003775593.1	outer membrane /protective OMA87 antigen protein
Herbaspirillum seropedicae SmR1	8.60E-006	Omp85	YP_003774959.1	outer membrane lipoprotein
Herminiimonas arsenicoxydans	9.20E-120	BamB	YP_001099569.1	serine/threonine protein kinase
Herminiimonas arsenicoxydans	2.10E-006	BamC	YP_001100433.1	putative lipoprotein

Herminiimonas arsenicoxydans	2.90E-096	BamD	YP_001100201.1	TPR repeat-containing protein
Herminiimonas arsenicoxydans	4.30E-005	BamD	YP_001100661.1	putative transmembrane protein
Herminiimonas arsenicoxydans	8.80E-005	BamD	YP_001100861.1	hypothetical protein HEAR2618
Herminiimonas arsenicoxydans	2.40E-017	BamE	YP_001100899.1	hypothetical protein HEAR2656
Herminiimonas arsenicoxydans	1.50E-193	Omp85	YP_001099643.1	outer membrane protein sensing stress
Herminiimonas arsenicoxydans	6.70E-006	Omp85	YP_001100761.1	putative signal peptide
Janthinobacterium sp. Marseille	2.30E-108	BamB	YP_001353814.1	pyrrolo-quinoline quinone
Janthinobacterium sp. Marseille	7.40E-007	BamC	YP_001352973.1	hypothetical protein mma_1283
Janthinobacterium sp. Marseille	2.60E-097	BamD	YP_001353082.1	competence lipoprotein ComL
Janthinobacterium sp. Marseille	8.70E-006	BamD	YP_001354159.1	Tol-Pal cell envelope complex subunit YbgF
Janthinobacterium sp. Marseille	1.00E-017	BamE	YP_001354581.1	hypothetical protein mma_2890
Janthinobacterium sp. Marseille	6.50E-005	BamE	YP_001352798.1	hypothetical protein mma_1108
Janthinobacterium sp. Marseille	1.90E-198	Omp85	YP_001353740.1	outer membrane protein
Janthinobacterium sp. Marseille	1.10E-006	Omp85	YP_001354293.1	hypothetical protein mma_2603
Laribacter hongkongensis HLHK9	6.20E-076	BamB	YP_002794690.1	pyrrolo-quinoline quinone
Laribacter hongkongensis HLHK9	2.60E-103	BamD	YP_002796212.1	ComL
Laribacter hongkongensis HLHK9	7.10E-023	BamE	YP_002795429.1	SmpA/OmlA
Laribacter hongkongensis HLHK9	6.00E-005	BamE	YP_002795929.1	hypothetical protein LHK_01936
Laribacter hongkongensis HLHK9	6.50E-232	Omp85	YP_002796276.1	outer membrane protein
Laribacter hongkongensis HLHK9	7.20E-006	Omp85	YP_002794520.1	Surface antigen
Leptothrix cholodnii SP-6	2.60E-029	BamB	YP_001791895.1	outer membrane assembly lipoprotein YfgL
Leptothrix cholodnii SP-6	9.20E-008	BamB	YP_001789888.1	Pyrrolo-quinoline quinone
Leptothrix cholodnii SP-6	1.80E-007	BamB	YP_001790843.1	methanol/ethanol family PQQ-dependent dehydrogenase
Leptothrix cholodnii SP-6	1.10E-006	BamB	YP_001789886.1	Pyrrolo-quinoline quinone
Leptothrix cholodnii SP-6	9.40E-005	BamB	YP_001792131.1	methanol/ethanol family PQQ-dependent dehydrogenase
Leptothrix cholodnii SP-6	4.50E-009	BamC	YP_001791671.1	NlpB/DapX family lipoprotein
Leptothrix cholodnii SP-6	2.10E-069	BamD	YP_001791906.1	putative transmembrane protein
Leptothrix cholodnii SP-6	2.50E-019	BamE	YP_001789515.1	SmpA/OmlA domain-containing protein
Leptothrix cholodnii SP-6	1.90E-173	Omp85	YP_001791872.1	outer membrane protein assembly complex, YaeT protein
Leptothrix cholodnii SP-6	2.00E-005	Omp85	YP_001792186.1	surface antigen (D15)
Methylibium petroleiphilum PM1	1.20E-049	BamB	YP_001021186.1	hypothetical protein Mpe_A1993
Methylibium petroleiphilum PM1	7.50E-009	BamB	YP_001020074.1	alcohol dehydrogenase large subunit
Methylibium petroleiphilum PM1	2.60E-008	BamB	YP_001022848.1	putative alcohol dehydrogenase
Methylibium petroleiphilum PM1	4.70E-008	BamB	YP_001020792.1	putative alcohol dehydrogenase
Methylibium petroleiphilum PM1	7.90E-008	BamB	YP_001020788.1	putative quinoprotein ethanol dehydrogenase
Methylibium petroleiphilum PM1	8.40E-008	BamB	YP_001019560.1	putative alcohol dehydrogenase
Methylibium petroleiphilum PM1	1.80E-007	BamB	YP_001019670.1	quinoprotein ethanol dehydrogenase
Methylibium petroleiphilum PM1	7.50E-007	BamB	YP_001020102.1	alcohol dehydrogenase

Methylibium petroleiphilum PM1	3.30E-006	BamB	YP_001019673.1	quinoprotein alcohol dehydrogenase
Methylibium petroleiphilum PM1	5.70E-006	BamB	YP_001019538.1	putative quinoprotein ethanol dehydrogenase
Methylibium petroleiphilum PM1	1.30E-005	BamB	YP_001020791.1	glucose dehydrogenase
Methylibium petroleiphilum PM1	8.30E-005	BamB	YP_001022581.1	putative methanol dehydrogenase protein, large subunit
Methylibium petroleiphilum PM1	2.00E-005	BamC	YP_001021752.1	putative transmembrane protein
Methylibium petroleiphilum PM1	3.60E-091	BamD	YP_001021197.1	putative transmembrane protein
Methylibium petroleiphilum PM1	9.80E-005	BamD	YP_001021992.1	putative ABC-type Co2+ transport system, periplasmic component
Methylibium petroleiphilum PM1	4.50E-015	BamE	YP_001019419.1	small protein A (tmRNA-binding)-like protein
Methylibium petroleiphilum PM1	1.30E-171	Omp85	YP_001021164.1	putative outer membrane signal peptide protein
Methylibium petroleiphilum PM1	3.50E-006	Omp85	YP_001020286.1	hypothetical protein Mpe_A1090
Methylobacillus flagellatus KT	2.50E-118	BamB	YP_545724.1	Pyrrolo-quinoline quinone
Methylobacillus flagellatus KT	2.50E-006	BamB	YP_546422.1	Pyrrolo-quinoline quinone
Methylobacillus flagellatus KT	1.70E-005	BamB	YP_544456.1	Pyrrolo-quinoline quinone
Methylobacillus flagellatus KT	2.40E-005	BamB	YP_545560.1	Pyrrolo-quinoline quinone
Methylobacillus flagellatus KT	3.00E-005	BamB	YP_545826.1	Pyrrolo-quinoline quinone
Methylobacillus flagellatus KT	7.70E-005	BamB	YP_546152.1	Pyrrolo-quinoline quinone
Methylobacillus flagellatus KT	2.10E-005	BamC	YP_545095.1	uncharacterized lipoprotein
Methylobacillus flagellatus KT	2.10E-005	BamC	YP_544951.1	uncharacterized lipoprotein
Methylobacillus flagellatus KT	7.40E-097	BamD	YP_545722.1	putative competence lipoprotein precursor
Methylobacillus flagellatus KT	9.00E-017	BamE	YP_544885.1	SmpA/OmlA
Methylobacillus flagellatus KT	1.80E-214	Omp85	YP_545631.1	surface antigen (D15)
Methylobacillus flagellatus KT	4.00E-006	Omp85	YP_545928.1	surface antigen (D15)
Methylotenera mobilis JLW8	3.90E-105	BamB	YP_003048308.1	outer membrane assembly lipoprotein YfgL
Methylotenera mobilis JLW8	8.90E-009	BamB	YP_003048647.1	Pyrrolo-quinoline quinone
Methylotenera mobilis JLW8	7.30E-006	BamB	YP_003049477.1	PQQ-dependent dehydrogenase
Methylotenera mobilis JLW8	2.70E-005	BamB	YP_003049201.1	PQQ-dependent dehydrogenase
Methylotenera mobilis JLW8	8.90E-006	BamC	YP_003049382.1	lipoprotein
Methylotenera mobilis JLW8	1.70E-096	BamD	YP_003048313.1	outer membrane assembly lipoprotein YfiO
Methylotenera mobilis JLW8	9.30E-005	BamD	YP_003049506.1	tol-pal system protein YbgF
Methylotenera mobilis JLW8	9.60E-005	BamD	YP_003048513.1	type II and III secretion system protein
Methylotenera mobilis JLW8	4.40E-020	BamE	YP_003049140.1	SmpA/OmlA domain-containing protein
Methylotenera mobilis JLW8	3.80E-214	Omp85	YP_003048593.1	outer membrane protein assembly complex protein YaeT
Methylotenera mobilis JLW8	7.20E-006	Omp85	YP_003048181.1	surface antigen (D15)
Methylotenera versatilis 301	1.30E-105	BamB	YP_003673884.1	outer membrane assembly lipoprotein YfgL
Methylotenera versatilis 301	3.20E-008	BamB	YP_003674218.1	PQQ-dependent enzyme-like protein
Methylotenera versatilis 301	3.80E-006	BamB	YP_003675402.1	PQQ-dependent dehydrogenase
Methylotenera versatilis 301	2.90E-006	BamC	YP_003675311.1	lipoprotein

Methylotenera versatilis 301	8.60E-093	BamD	YP_003673888.1	outer membrane assembly lipoprotein YfiO
Methylotenera versatilis 301	2.50E-005	BamD	YP_003675443.1	tol-pal system protein YbgF
Methylotenera versatilis 301	3.30E-022	BamE	YP_003674953.1	SmpA/OmlA domain-containing protein
Methylotenera versatilis 301	8.80E-229	Omp85	YP_003674280.1	outer membrane protein assembly complex protein YaeT
Methylotenera versatilis 301	4.50E-006	Omp85	YP_003673643.1	surface antigen (D15)
Methylovorus glucosetrophus SIP3-4	3.60E-116	BamB	YP_003051483.1	outer membrane assembly lipoprotein YfgL
Methylovorus glucosetrophus SIP3-4	8.50E-007	BamB	YP_003051084.1	Pyrrolo-quinoline quinone
Methylovorus glucosetrophus SIP3-4	2.70E-006	BamB	YP_003050509.1	PQQ-dependent dehydrogenase
Methylovorus glucosetrophus SIP3-4	4.60E-006	BamB	YP_003052313.1	PQQ-dependent dehydrogenase
Methylovorus glucosetrophus SIP3-4	1.10E-005	BamB	YP_003051583.1	PQQ-dependent dehydrogenase
Methylovorus glucosetrophus SIP3-4	1.10E-005	BamB	YP_003049792.1	PQQ-dependent dehydrogenase
Methylovorus glucosetrophus SIP3-4	1.50E-005	BamB	YP_003052117.1	PQQ-dependent dehydrogenase
Methylovorus glucosetrophus SIP3-4	2.10E-006	BamC	YP_003051572.1	lipoprotein
Methylovorus glucosetrophus SIP3-4	3.10E-098	BamD	YP_003051481.1	outer membrane assembly lipoprotein YfiO
Methylovorus glucosetrophus SIP3-4	1.80E-015	BamE	YP_003051650.1	SmpA/OmlA domain-containing protein
Methylovorus glucosetrophus SIP3-4	4.90E-005	BamE	YP_003050302.1	hypothetical protein Msip34_0527
Methylovorus glucosetrophus SIP3-4	1.80E-208	Omp85	YP_003051175.1	outer membrane protein assembly complex protein YaeT
Methylovorus sp. MP688	5.60E-117	BamB	YP_004040100.1	outer membrane assembly lipoprotein yfgl
Methylovorus sp. MP688	2.70E-006	BamB	YP_004039179.1	pqq-dependent dehydrogenase
Methylovorus sp. MP688	3.00E-006	BamB	YP_004039785.1	pyrrolo-quinoline quinone
Methylovorus sp. MP688	5.50E-006	BamB	YP_004040874.1	pqq-dependent dehydrogenase
Methylovorus sp. MP688	1.10E-005	BamB	YP_004038451.1	pqq-dependent dehydrogenase
Methylovorus sp. MP688	4.20E-006	BamC	YP_004040192.1	lipoprotein
Methylovorus sp. MP688	3.50E-098	BamD	YP_004040098.1	outer membrane assembly lipoprotein yfio
Methylovorus sp. MP688	1.80E-015	BamE	YP_004040280.1	smpa/omla domain-containing protein
Methylovorus sp. MP688	4.90E-005	BamE	YP_004038970.1	hypothetical protein MPQ_0551
Methylovorus sp. MP688	1.80E-208	Omp85	YP_004039871.1	outer membrane protein assembly complex protein YaeT
Neisseria gonorrhoeae FA 1090	2.70E-005	BamC	YP_208051.1	hypothetical protein NGO0948
Neisseria gonorrhoeae FA 1090	3.60E-098	BamD	YP_207439.1	ComL
Neisseria gonorrhoeae FA 1090	2.70E-019	BamE	YP_208811.1	hypothetical protein NGO1780
Neisseria gonorrhoeae FA 1090	0.00E+000	Omp85	YP_208831.1	hypothetical protein NGO1801
Neisseria gonorrhoeae FA 1090	1.90E-005	Omp85	YP_208979.1	hypothetical protein NGO1956
Neisseria gonorrhoeae NCCP11945	2.70E-005	BamC	YP_002001475.1	hypothetical protein NGK_0850
Neisseria gonorrhoeae NCCP11945	3.60E-098	BamD	YP_002001047.1	ComL, competence lipoprotein
Neisseria gonorrhoeae NCCP11945	2.70E-019	BamE	YP_002003119.1	Lipoprotein
Neisseria gonorrhoeae NCCP11945	0.00E+000	Omp85	YP_002003098.1	Outer membrane protein Omp85
Neisseria gonorrhoeae NCCP11945	2.80E-005	Omp85	YP_002002918.1	hypothetical protein NGK_2293
Neisseria lactamica ST-640	4.30E-006	BamC	YP_004048914.1	lipoprotein

Neisseria lactamica ST-640	1.10E-100	BamD	YP_004049101.1	competence lipoprotein
Neisseria lactamica ST-640	7.00E-013	BamE	YP_004047798.1	outer membrane lipoprotein
Neisseria lactamica ST-640	1.10E-005	BamE	YP_004049396.1	lipoprotein
Neisseria lactamica ST-640	0.00E+000	Omp85	YP_004047769.1	outer membrane protein OMP85
Neisseria lactamica ST-640	3.90E-005	Omp85	YP_004049578.1	outer membrane protein
Neisseria meningitidis 053442	1.40E-005	BamC	YP_001599009.1	lipoprotein
Neisseria meningitidis 053442	2.20E-100	BamD	YP_001598811.1	competence lipoprotein
Neisseria meningitidis 053442	6.60E-019	BamE	YP_001600030.1	lipoprotein
Neisseria meningitidis 053442	1.50E-005	BamE	YP_001598484.1	lipoprotein
Neisseria meningitidis 053442	0.00E+000	Omp85	YP_001600055.1	outer membrane protein OMP85
Neisseria meningitidis 053442	2.90E-005	Omp85	YP_001600176.1	putative outer membrane protein
Neisseria meningitidis alpha14	2.00E-005	BamC	YP_003083030.1	putative lipoprotein
Neisseria meningitidis alpha14	2.20E-099	BamD	YP_003082807.1	DNA uptake lipoprotein
Neisseria meningitidis alpha14	3.30E-020	BamE	YP_003083975.1	putative lipoprotein
Neisseria meningitidis alpha14	2.70E-006	BamE	YP_003082505.1	lipoprotein
Neisseria meningitidis alpha14	0.00E+000	Omp85	YP_003083995.1	probable surface antigen
Neisseria meningitidis alpha14	2.20E-005	Omp85	YP_003082289.1	putative outer membrane protein
Neisseria meningitidis FAM18	6.90E-006	BamC	YP_974977.1	putative lipoprotein
Neisseria meningitidis FAM18	2.20E-099	BamD	YP_974745.1	competence lipoprotein
Neisseria meningitidis FAM18	5.10E-020	BamE	YP_974322.1	putative outer membrane lipoprotein
Neisseria meningitidis FAM18	8.30E-006	BamE	YP_974438.1	lipoprotein
Neisseria meningitidis FAM18	0.00E+000	Omp85	YP_974302.1	outer membrane protein OMP85
Neisseria meningitidis FAM18	3.10E-005	Omp85	YP_976025.1	putative outer membrane protein
Neisseria meningitidis MC58	8.60E-006	BamC	NP_273967.1	hypothetical protein NMB0928
Neisseria meningitidis MC58	2.80E-099	BamD	NP_273745.1	competence lipoprotein
Neisseria meningitidis MC58	3.70E-020	BamE	NP_273262.1	putative lipoprotein
Neisseria meningitidis MC58	3.70E-006	BamE	NP_274893.1	lipoprotein
Neisseria meningitidis MC58	0.00E+000	Omp85	NP_273240.1	outer membrane protein OMP85
Neisseria meningitidis MC58	3.30E-005	Omp85	NP_275119.1	hypothetical protein NMB2134
Neisseria meningitidis Z2491	1.30E-005	BamC	YP_002342526.1	putative lipoprotein
Neisseria meningitidis Z2491	2.20E-099	BamD	YP_002342337.1	competence lipoprotein
Neisseria meningitidis Z2491	6.60E-019	BamE	YP_002341618.1	putative lipoprotein
Neisseria meningitidis Z2491	4.50E-006	BamE	YP_002342040.1	lipoprotein
Neisseria meningitidis Z2491	0.00E+000	Omp85	YP_002341637.1	outer membrane protein OMP85
Neisseria meningitidis Z2491	3.00E-005	Omp85	YP_002341819.1	putative outer membrane protein
Nitrosomonas europaea ATCC 19718	3.80E-078	BamB	NP_840248.1	PQQ repeat-containing protein
Nitrosomonas europaea ATCC 19718	1.50E-008	BamC	NP_842399.1	transmembrane protein
Nitrosomonas europaea ATCC 19718	2.10E-098	BamD	NP_840591.1	TPR repeat-containing protein

Nitrosomonas europaea ATCC 19718	1.10E-006	BamD	NP_840314.1	TPR repeat-containing protein
Nitrosomonas europaea ATCC 19718	7.90E-005	BamD	NP_841485.1	TPR repeat-containing protein
Nitrosomonas europaea ATCC 19718	3.20E-025	BamE	NP_840699.1	outer membrane lipoprotein OmlA
Nitrosomonas europaea ATCC 19718	5.90E-213	Omp85	NP_841742.1	surface antigen (D15)
Nitrosomonas europaea ATCC 19718	1.60E-005	Omp85	NP_841004.1	lipoprotein
Nitrosomonas eutropha C91	1.20E-073	BamB	YP_748352.1	Pyrrolo-quinoline quinone
Nitrosomonas eutropha C91	1.20E-008	BamC	YP_746969.1	NlpB/DapX family lipoprotein
Nitrosomonas eutropha C91	5.10E-098	BamD	YP_747448.1	TPR repeat-containing protein
Nitrosomonas eutropha C91	1.90E-006	BamD	YP_746540.1	TPR repeat-containing protein
Nitrosomonas eutropha C91	1.40E-005	BamD	YP_748260.1	TPR repeat-containing protein
Nitrosomonas eutropha C91	1.60E-005	BamD	YP_746758.1	TPR repeat-containing protein
Nitrosomonas eutropha C91	1.90E-021	BamE	YP_748135.1	SmpA/OmlA domain-containing protein
Nitrosomonas eutropha C91	6.80E-215	Omp85	YP_748215.1	surface antigen (D15)
Nitrosomonas eutropha C91	7.10E-005	Omp85	YP_747622.1	surface antigen (D15)
Nitrosomonas sp. AL212	3.90E-083	BamB	YP_004294302.1	outer membrane assembly lipoprotein YfgL
Nitrosomonas sp. AL212	7.00E-007	BamC	YP_004295093.1	putative transmembrane protein
Nitrosomonas sp. AL212	4.60E-108	BamD	YP_004294186.1	outer membrane assembly lipoprotein YfiO
Nitrosomonas sp. AL212	3.80E-005	BamD	YP_004296029.1	hypothetical protein NAL212_3105
Nitrosomonas sp. AL212	4.70E-021	BamE	YP_004295773.1	SmpA/OmlA domain-containing protein
Nitrosomonas sp. AL212	3.00E-005	BamE	YP_004294569.1	hypothetical protein NAL212_1526
Nitrosomonas sp. AL212	5.10E-209	Omp85	YP_004296056.1	outer membrane protein assembly complex, YaeT protein
Nitrosomonas sp. AL212	2.20E-005	Omp85	YP_004294820.1	surface antigen (D15)
Nitrosomonas sp. Is79A3	3.10E-076	BamB	YP_004695345.1	outer membrane assembly lipoprotein YfgL
Nitrosomonas sp. Is79A3	1.70E-007	BamC	YP_004693884.1	NlpBDapX family lipoprotein
Nitrosomonas sp. Is79A3	8.50E-098	BamD	YP_004696217.1	outer membrane assembly lipoprotein YfiO
Nitrosomonas sp. Is79A3	8.80E-031	BamE	YP_004694403.1	SmpA/OmlA domain-containing protein
Nitrosomonas sp. Is79A3	1.70E-005	BamE	YP_004694262.1	hypothetical protein Nit79A3_1000
Nitrosomonas sp. Is79A3	3.30E-214	Omp85	YP_004696598.1	outer membrane protein assembly complex, YaeT protein
Nitrosomonas sp. Is79A3	7.40E-005	Omp85	YP_004695338.1	surface antigen (D15)
Nitrosospira multiformis ATCC 25196	4.80E-090	BamB	YP_413055.1	Pyrrolo-quinoline quinone
Nitrosospira multiformis ATCC 25196	7.10E-008	BamC	YP_412234.1	NlpBDapX lipoprotein
Nitrosospira multiformis ATCC 25196	3.80E-099	BamD	YP_412662.1	TPR repeat-containing protein
Nitrosospira multiformis ATCC 25196	4.90E-005	BamD	YP_413264.1	type II and III secretion system protein
Nitrosospira multiformis ATCC 25196	2.30E-028	BamE	YP_411176.1	SmpA/OmlA
Nitrosospira multiformis ATCC 25196	4.00E-006	BamE	YP_412228.1	hypothetical protein Nmul_A1533
Nitrosospira multiformis ATCC 25196	1.70E-225	Omp85	YP_411364.1	surface antigen (D15)
Nitrosospira multiformis ATCC 25196	1.60E-005	Omp85	YP_412486.1	surface antigen (D15)
Polaromonas naphthalenivorans CJ2	5.60E-062	BamB	YP_982106.1	Pyrrolo-quinoline quinone

Polaromonas naphthalenivorans CJ2	1.60E-007	BamB	YP_980966.1	Pyrrolo-quinoline quinone
Polaromonas naphthalenivorans CJ2	3.60E-007	BamB	YP_983227.1	Pyrrolo-quinoline quinone
Polaromonas naphthalenivorans CJ2	4.00E-007	BamC	YP_981431.1	NlpB/DapX family lipoprotein
Polaromonas naphthalenivorans CJ2	9.50E-070	BamD	YP_982095.1	hypothetical protein Pnap_1864
Polaromonas naphthalenivorans CJ2	4.30E-011	BamE	YP_984007.1	SmpA/OmlA domain-containing protein
Polaromonas naphthalenivorans CJ2	5.30E-006	BamE	YP_980514.1	putative lipoprotein transmembrane
Polaromonas naphthalenivorans CJ2	7.90E-173	Omp85	YP_981997.1	surface antigen (D15)
Polaromonas naphthalenivorans CJ2	2.20E-005	Omp85	YP_981095.1	surface antigen (D15)
Polaromonas sp. JS666	1.70E-063	BamB	YP_549419.1	Pyrrolo-quinoline quinone
Polaromonas sp. JS666	1.80E-007	BamB	YP_552059.1	Pyrrolo-quinoline quinone
Polaromonas sp. JS666	5.70E-008	BamC	YP_549983.1	NlpBDapX lipoprotein
Polaromonas sp. JS666	9.00E-066	BamD	YP_549430.1	hypothetical protein Bpro_2616
Polaromonas sp. JS666	2.00E-010	BamE	YP_551390.1	SmpA/OmlA
Polaromonas sp. JS666	1.00E-006	BamE	YP_547250.1	putative lipoprotein transmembrane
Polaromonas sp. JS666	6.40E-178	Omp85	YP_549501.1	surface antigen (D15)
Polaromonas sp. JS666	9.80E-005	Omp85	YP_548067.1	surface antigen (D15)
Polynucleobacter necessarius subsp. asymbioticus QLW-P1DMWA-1	3.10E-098	BamB	YP_001156066.1	Pyrrolo-quinoline quinone
Polynucleobacter necessarius subsp. asymbioticus QLW-P1DMWA-1	9.30E-008	BamC	YP_001155630.1	NlpB/DapX family lipoprotein
Polynucleobacter necessarius subsp. asymbioticus QLW-P1DMWA-1	9.80E-089	BamD	YP_001155700.1	putative transmembrane protein
Polynucleobacter necessarius subsp. asymbioticus QLW-P1DMWA-1	1.30E-013	BamE	YP_001155015.1	SmpA/OmlA domain-containing protein
Polynucleobacter necessarius subsp. asymbioticus QLW-P1DMWA-1	1.20E-175	Omp85	YP_001156221.1	surface antigen (D15)
Polynucleobacter necessarius subsp. necessarius STIR1	1.30E-105	BamB	YP_001797518.1	outer membrane assembly lipoprotein YfgL
Polynucleobacter necessarius subsp. necessarius STIR1	2.30E-009	BamC	YP_001797788.1	NlpBDapX family lipoprotein
Polynucleobacter necessarius subsp. necessarius STIR1	6.40E-087	BamD	YP_001797734.1	putative transmembrane protein
Polynucleobacter necessarius subsp. necessarius STIR1	2.70E-012	BamE	YP_001797161.1	SmpA/OmlA domain-containing protein
Polynucleobacter necessarius subsp. necessarius STIR1	6.60E-005	BamE	YP_001798099.1	CcmE/CycJ protein
Polynucleobacter necessarius subsp. necessarius STIR1	3.50E-182	Omp85	YP_001797392.1	outer membrane protein assembly complex, YaeT protein
Pusillimonas sp. T7-7	1.50E-097	BamB	YP_004416562.1	putative quinoprotein
Pusillimonas sp. T7-7	3.00E-005	BamB	YP_004418261.1	hypothetical protein PT7_3097
Pusillimonas sp. T7-7	1.80E-007	BamC	YP_004415335.1	lipoprotein
Pusillimonas sp. T7-7	1.70E-082	BamD	YP_004415601.1	competence lipoprotein
Pusillimonas sp. T7-7	7.40E-026	BamE	YP_004417102.1	outer membrane lipoprotein
Pusillimonas sp. T7-7	5.70E-008	BamE	YP_004417684.1	lipoprotein PlpD
Pusillimonas sp. T7-7	4.80E-189	Omp85	YP_004416198.1	surface antigen
Ralstonia eutropha H16	7.90E-158	BamB	YP_726824.1	hypothetical protein H16_A2361
Ralstonia eutropha H16	1.00E-006	BamB	YP_726353.1	dehydrogenase, PQQ dependent
Ralstonia eutropha H16	3.80E-007	BamB	YP_840568.1	quinate dehydrogenase (PQQ)
Ralstonia eutropha H16	3.40E-007	BamC	YP_725714.1	uncharacterized lipoprotein

Ralstonia eutropha H16	3.30E-100	BamD	YP_725936.1	DNA uptake lipoprotein
Ralstonia eutropha H16	3.80E-018	BamE	YP_727585.1	lipoprotein
Ralstonia eutropha H16	3.30E-005	BamE	YP_727398.1	putative lipoprotein
Ralstonia eutropha H16	3.90E-231	Omp85	YP_726515.1	outer membrane protein, surface antigen OMA87
Ralstonia eutropha H16	4.90E-005	Omp85	YP_727400.1	outer membrane protein
Ralstonia eutropha JMP134	1.30E-163	BamB	YP_296291.1	Pyrrolo-quinoline quinone
Ralstonia eutropha JMP134	1.80E-007	BamB	YP_295675.1	Pyrrolo-quinoline quinone
Ralstonia eutropha JMP134	2.00E-007	BamB	YP_295993.1	Pyrrolo-quinoline quinone
Ralstonia eutropha JMP134	2.00E-007	BamB	YP_298358.1	Pyrrolo-quinoline quinone:cytochrome c, class I
Ralstonia eutropha JMP134	1.10E-006	BamB	YP_299608.1	Pyrrolo-quinoline quinone
Ralstonia eutropha JMP134	2.00E-006	BamC	YP_295327.1	transmembrane protein
Ralstonia eutropha JMP134	4.20E-100	BamD	YP_295557.1	transmembrane protein
Ralstonia eutropha JMP134	2.20E-020	BamE	YP_297041.1	SmpA/OmlA
Ralstonia eutropha JMP134	3.10E-005	BamE	YP_294899.1	lipoprotein
Ralstonia eutropha JMP134	2.60E-006	BamE	YP_300034.1	OmpA/MotB:SmpA/OmlA
Ralstonia eutropha JMP134	7.40E-005	BamE	YP_298397.1	transmembrane protein
Ralstonia eutropha JMP134	2.70E-226	Omp85	YP_296082.1	surface antigen (D15):surface antigen variable number
Ralstonia eutropha JMP134	1.70E-005	Omp85	YP_294897.1	surface antigen (D15)
Ralstonia pickettii 12D	2.80E-214	BamB	YP_002981118.1	outer membrane assembly lipoprotein YfgL
Ralstonia pickettii 12D	8.80E-008	BamB	YP_002982049.1	PQQ-dependent dehydrogenase
Ralstonia pickettii 12D	2.50E-006	BamC	YP_002981048.1	NlpBDapX family lipoprotein
Ralstonia pickettii 12D	7.60E-102	BamD	YP_002981548.1	outer membrane assembly lipoprotein YfiO
Ralstonia pickettii 12D	2.90E-023	BamE	YP_002982518.1	SmpA/OmlA domain-containing protein
Ralstonia pickettii 12D	5.60E-005	BamE	YP_002981852.1	hypothetical protein Rpic12D_1899
Ralstonia pickettii 12D	8.20E-005	BamE	YP_002982137.1	lipoprotein
Ralstonia pickettii 12D	1.60E-211	Omp85	YP_002981311.1	outer membrane protein assembly complex, YaeT protein
Ralstonia pickettii 12D	1.80E-005	Omp85	YP_002982139.1	surface antigen (D15)
Ralstonia pickettii 12J	5.50E-213	BamB	YP_001898642.1	outer membrane assembly lipoprotein YfgL
Ralstonia pickettii 12J	5.20E-008	BamB	YP_001900058.1	PQQ-dependent dehydrogenase
Ralstonia pickettii 12J	3.20E-006	BamC	YP_001898568.1	NlpBDapX family lipoprotein
Ralstonia pickettii 12J	4.30E-098	BamD	YP_001899487.1	outer membrane assembly lipoprotein YfiO
Ralstonia pickettii 12J	1.60E-020	BamE	YP_001900540.1	SmpA/OmlA domain-containing protein
Ralstonia pickettii 12J	7.40E-005	BamE	YP_001900154.1	putative lipoprotein
Ralstonia pickettii 12J	8.80E-005	BamE	YP_001899788.1	hypothetical protein Rpic_2222
Ralstonia pickettii 12J	2.10E-217	Omp85	YP_001898862.1	outer membrane protein assembly complex, YaeT protein
Ralstonia pickettii 12J	1.80E-005	Omp85	YP_001900156.1	surface antigen (D15)
Ralstonia solanacearum GMI1000	1.60E-193	BamB	NP_519339.1	lipoprotein transmembrane
Ralstonia solanacearum GMI1000	4.60E-006	BamC	NP_519267.1	hypothetical protein RSc1146

Ralstonia solanacearum GMI1000	7.40E-103	BamD	NP_519748.1	hypothetical protein RSc1627
Ralstonia solanacearum GMI1000	5.00E-024	BamE	NP_520867.1	lipoprotein transmembrane
Ralstonia solanacearum GMI1000	5.40E-005	BamE	NP_520503.1	lipoprotein
Ralstonia solanacearum GMI1000	7.60E-005	BamE	NP_520192.1	hypothetical protein RSc2071
Ralstonia solanacearum GMI1000	1.00E-217	Omp85	NP_519533.1	outer membrane signal peptide protein
Ralstonia solanacearum GMI1000	2.50E-005	Omp85	NP_520505.1	lipoprotein
Ramlibacter tataouinensis TTB310	9.70E-055	BamB	YP_004619007.1	hypothetical protein Rta_18970
Ramlibacter tataouinensis TTB310	1.60E-007	BamC	YP_004619688.1	hypothetical protein Rta_25710
Ramlibacter tataouinensis TTB310	3.20E-017	BamE	YP_004617310.1	Outer membrane lipoprotein omlA
Ramlibacter tataouinensis TTB310	7.10E-174	Omp85	YP_004618860.1	Outer membrane protein insertion machinery protein Omp85
Ramlibacter tataouinensis TTB310	4.40E-005	Omp85	YP_004619657.1	hypothetical protein Rta_25420
Rhodoferax ferrireducens T118	6.20E-040	BamB	YP_523553.1	Pyrrolo-quinoline quinone
Rhodoferax ferrireducens T118	5.30E-008	BamC	YP_523351.1	NlpBDapX lipoprotein
Rhodoferax ferrireducens T118	1.50E-090	BamD	YP_523564.1	hypothetical protein Rfer_2315
Rhodoferax ferrireducens T118	4.90E-013	BamE	YP_522035.1	SmpA/OmlA
Rhodoferax ferrireducens T118	7.20E-182	Omp85	YP_523252.1	surface antigen (D15)
Taylorella equigenitalis MCE9	1.80E-058	BamB	YP_004130585.1	Outer membrane protein YfgL
Taylorella equigenitalis MCE9	1.10E-056	BamD	YP_004129144.1	component of the lipoprotein assembly complex protein
Taylorella equigenitalis MCE9	8.10E-005	BamD	YP_004130052.1	TPR repeat containing exported protein
Taylorella equigenitalis MCE9	1.30E-015	BamE	YP_004129314.1	Outer membrane lipoprotein SmpA
Taylorella equigenitalis MCE9	1.60E-006	BamE	YP_004129769.1	Outer membrane protein A precursor
Taylorella equigenitalis MCE9	1.30E-148	Omp85	YP_004130465.1	Outer membrane protein assembly factor YaeT precursor
Thauera sp. MZ1T	1.10E-098	BamB	YP_002890358.1	outer membrane assembly lipoprotein YfgL
Thauera sp. MZ1T	1.10E-007	BamB	YP_002889793.1	methanol/ethanol family PQQ-dependent dehydrogenase
Thauera sp. MZ1T	3.00E-007	BamC	YP_002889981.1	lipoprotein
Thauera sp. MZ1T	3.30E-099	BamD	YP_002890102.1	outer membrane assembly lipoprotein YfiO
Thauera sp. MZ1T	2.80E-030	BamE	YP_002355377.1	SmpA/OmlA domain-containing protein
Thauera sp. MZ1T	5.10E-223	Omp85	YP_002355806.1	outer membrane protein assembly complex, YaeT protein
Thauera sp. MZ1T	7.60E-006	Omp85	YP_002355065.1	surface antigen (D15)
Thiobacillus denitrificans ATCC 25259	7.70E-089	BamB	YP_314355.1	pyrrolo-quinoline quinone
Thiobacillus denitrificans ATCC 25259	6.10E-007	BamC	YP_314857.1	lipoprotein
Thiobacillus denitrificans ATCC 25259	2.60E-107	BamD	YP_314567.1	putative competence lipoprotein
Thiobacillus denitrificans ATCC 25259	3.00E-005	BamD	YP_315962.1	TPR repeat-containing protein
Thiobacillus denitrificans ATCC 25259	4.40E-025	BamE	YP_314882.1	hypothetical protein Tbd_1124
Thiobacillus denitrificans ATCC 25259	5.10E-235	Omp85	YP_314551.1	surface antigen (D15)
Thiobacillus denitrificans ATCC 25259	3.30E-005	Omp85	YP_314591.1	hypothetical protein Tbd_0833
Thiomonas intermedia K12	1.40E-056	BamB	YP_003643471.1	outer membrane assembly lipoprotein YfgL
Thiomonas intermedia K12	4.20E-008	BamC	YP_003642510.1	NlpBDapX family lipoprotein

Thiomonas intermedia K12	9.40E-089	BamD	YP_003643029.1	outer membrane assembly lipoprotein YfiO
Thiomonas intermedia K12	2.20E-020	BamE	YP_003641783.1	SmpA/OmlA domain protein
Thiomonas intermedia K12	3.70E-183	Omp85	YP_003643813.1	outer membrane protein assembly complex, YaeT protein
Thiomonas intermedia K12	1.40E-005	Omp85	YP_003644636.1	surface antigen (D15)
Variovorax paradoxus EPS	4.10E-051	BamB	YP_004155995.1	outer membrane assembly lipoprotein yfgl
Variovorax paradoxus EPS	3.70E-008	BamB	YP_004157993.1	pqq-dependent dehydrogenase
Variovorax paradoxus EPS	6.50E-005	BamB	YP_004157998.1	membrane-bound pqq-dependent dehydrogenase, glucose/quinate/shikimate family
Variovorax paradoxus EPS	7.40E-008	BamC	YP_004154497.1	nlpbdapx family lipoprotein
Variovorax paradoxus EPS	1.10E-079	BamD	YP_004156006.1	outer membrane assembly lipoprotein yfio
Variovorax paradoxus EPS	5.00E-012	BamE	YP_004152767.1	smpa/omla domain-containing protein
Variovorax paradoxus EPS	5.80E-006	BamE	YP_004156216.1	hypothetical protein Varpa_3932
Variovorax paradoxus EPS	2.00E-164	Omp85	YP_004155615.1	outer membrane protein assembly complex, yaet protein
Variovorax paradoxus EPS	1.80E-156	Omp85	YP_004157140.1	outer membrane protein assembly complex, yaet protein
Variovorax paradoxus S110	7.20E-055	BamB	YP_002944091.1	outer membrane assembly lipoprotein YfgL
Variovorax paradoxus S110	9.00E-008	BamB	YP_002946886.1	PQQ-dependent dehydrogenase
Variovorax paradoxus S110	1.40E-005	BamB	YP_002944925.1	PQQ-dependent dehydrogenase
Variovorax paradoxus S110	1.80E-005	BamB	YP_002942620.1	PQQ-dependent dehydrogenase
Variovorax paradoxus S110	8.70E-005	BamB	YP_002946888.1	membrane-bound PQQ-dependent dehydrogenase, glucose/quinate/shikimate family
Variovorax paradoxus S110	7.30E-008	BamB	YP_002948071.1	PQQ-dependent dehydrogenase, methanol/ethanol family
Variovorax paradoxus S110	5.30E-008	BamC	YP_002945316.1	NlpBDapX family lipoprotein
Variovorax paradoxus S110	4.60E-079	BamD	YP_002944080.1	outer membrane assembly lipoprotein YfiO
Variovorax paradoxus S110	2.20E-013	BamE	YP_002942336.1	SmpA/OmlA domain-containing protein
Variovorax paradoxus S110	5.50E-005	BamE	YP_002947419.1	OmpA/MotB domain protein
Variovorax paradoxus S110	7.60E-166	Omp85	YP_002944498.1	outer membrane protein assembly complex, YaeT protein
Variovorax paradoxus S110	2.60E-162	Omp85	YP_002946133.1	outer membrane protein assembly complex, YaeT protein

Anaeromyxobacter dehalogenans 2CP-1	1.70E-014	BamB	YP_002492694.1	Pyrrolo-quinoline quinone
Anaeromyxobacter dehalogenans 2CP-1	1.80E-006	BamB	YP_002492718.1	Pyrrolo-quinoline quinone
Anaeromyxobacter dehalogenans 2CP-1	2.70E-015	BamD	YP_002493908.1	outer membrane assembly lipoprotein YfiO
Anaeromyxobacter dehalogenans 2CP-1	5.50E-097	Omp85	YP_002491619.1	outer membrane protein assembly complex, YaeT protein
Anaeromyxobacter dehalogenans 2CP-1	2.40E-010	Omp85	YP_002491110.1	surface antigen (D15)
Anaeromyxobacter dehalogenans 2CP-1	1.50E-005	Omp85	YP_002490608.1	surface antigen (D15)
Anaeromyxobacter dehalogenans 2CP-C	6.80E-015	BamB	YP_464866.1	PQQ repeat-containing protein
Anaeromyxobacter dehalogenans 2CP-C	1.50E-006	BamB	YP_464844.1	WD-40 repeat-containing protein
Anaeromyxobacter dehalogenans 2CP-C	6.10E-005	BamB	YP_467411.1	hypothetical protein Adeh_4210
Anaeromyxobacter dehalogenans 2CP-C	3.60E-015	BamD	YP_466570.1	hypothetical protein Adeh_3366
Anaeromyxobacter dehalogenans 2CP-C	2.90E-101	Omp85	YP_464293.1	surface antigen (D15)
Anaeromyxobacter dehalogenans 2CP-C	3.80E-010	Omp85	YP_463867.1	surface antigen (D15)
Anaeromyxobacter dehalogenans 2CP-C	3.00E-005	Omp85	YP_463378.1	surface antigen variable number
Anaeromyxobacter sp. Fw109-5	4.70E-014	BamB	YP_001379339.1	Pyrrolo-quinoline quinone
Anaeromyxobacter sp. Fw109-5	1.00E-005	BamB	YP_001379363.1	Pyrrolo-quinoline quinone
Anaeromyxobacter sp. Fw109-5	4.70E-005	BamB	YP_001381520.1	hypothetical protein Anae109_4358
Anaeromyxobacter sp. Fw109-5	4.80E-014	BamD	YP_001380598.1	tetratricopeptide domain-containing protein
Anaeromyxobacter sp. Fw109-5	2.00E-119	Omp85	YP_001378312.1	surface antigen (D15)
Anaeromyxobacter sp. Fw109-5	2.90E-010	Omp85	YP_001377896.1	surface antigen (D15)
Anaeromyxobacter sp. Fw109-5	1.50E-005	Omp85	YP_001377375.1	surface antigen (D15)
Anaeromyxobacter sp. K	2.30E-014	BamB	YP_002134558.1	pyrrolo-quinoline quinone
Anaeromyxobacter sp. K	1.90E-006	BamB	YP_002134582.1	pyrrolo-quinoline quinone
Anaeromyxobacter sp. K	7.90E-005	BamB	YP_002136674.1	pyrrolo-quinoline quinone
Anaeromyxobacter sp. K	3.10E-015	BamD	YP_002135790.1	outer membrane assembly lipoprotein YfiO
Anaeromyxobacter sp. K	9.20E-097	Omp85	YP_002133502.1	outer membrane protein assembly complex, YaeT protein
Anaeromyxobacter sp. K	1.90E-010	Omp85	YP_002133056.1	surface antigen (D15)
Anaeromyxobacter sp. K	1.30E-005	Omp85	YP_002132544.1	surface antigen (D15)
Bdellovibrio bacteriovorus HD100	2.40E-016	BamB	NP_968885.1	putative lipoprotein
Bdellovibrio bacteriovorus HD100	4.30E-008	BamB	NP_969656.1	putative serine/threonine protein kinase
Bdellovibrio bacteriovorus HD100	4.80E-015	BamD	NP_967143.1	competence protein ComL
Bdellovibrio bacteriovorus HD100	1.20E-005	BamD	NP_969287.1	hypothetical protein Bd2474
Bdellovibrio bacteriovorus HD100	1.80E-005	BamD	NP_967036.1	hypothetical protein Bd0006
Bdellovibrio bacteriovorus HD100	9.70E-005	BamD	NP_968052.1	putative soluble lytic transglycosylase
Bdellovibrio bacteriovorus HD100	4.00E-005	BamE	NP_967278.1	putative lipoprotein
Bdellovibrio bacteriovorus HD100	0.00E+000	Omp85	NP_969304.1	surface antigen
Bdellovibrio bacteriovorus HD100	1.70E-128	Omp85	NP_968381.1	hypothetical protein Bd1493
Desulfarculus baarsii DSM 2075	4.00E-015	BamD	YP_003807100.1	outer membrane assembly lipoprotein YfiO
Desulfarculus baarsii DSM 2075	1.90E-005	BamD	YP_003806871.1	hypothetical protein Deba_0907

Desulfarculus baarsii DSM 2075	2.90E-005	BamD	YP_003807855.1	hypothetical protein Deba_1896
Desulfarculus baarsii DSM 2075	3.10E-176	Omp85	YP_003808068.1	outer membrane protein assembly complex, YaeT protein
Desulfarculus baarsii DSM 2075	9.60E-008	Omp85	YP_003806803.1	surface antigen (D15)
Desulfatibacillum alkenivorans AK-01	1.40E-015	BamD	YP_002431565.1	outer membrane assembly lipoprotein YfiO
Desulfatibacillum alkenivorans AK-01	2.10E-005	BamD	YP_002430976.1	hypothetical protein Dalk_1811
Desulfatibacillum alkenivorans AK-01	5.30E-005	BamD	YP_002432857.1	branched-chain amino acid ABC transporter periplasmic protein
Desulfatibacillum alkenivorans AK-01	5.40E-005	BamD	YP_002429311.1	hypothetical protein Dalk_0133
Desulfatibacillum alkenivorans AK-01	7.00E-005	BamD	YP_002432757.1	hypothetical protein Dalk_3601
Desulfatibacillum alkenivorans AK-01	7.90E-153	Omp85	YP_002430952.1	outer membrane protein assembly complex, YaeT protein
Desulfatibacillum alkenivorans AK-01	1.30E-006	Omp85	YP_002432353.1	surface antigen (D15)
Desulfobacca acetoxidans DSM 11109	6.50E-014	BamD	YP_004369248.1	outer membrane assembly lipoprotein YfiO
Desulfobacca acetoxidans DSM 11109	4.70E-005	BamD	YP_004369366.1	hypothetical protein Desac_0294
Desulfobacca acetoxidans DSM 11109	9.40E-005	BamD	YP_004370349.1	hypothetical protein Desac_1311
Desulfobacca acetoxidans DSM 11109	3.10E-005	BamE	YP_004371168.1	hypothetical protein Desac_2158
Desulfobacca acetoxidans DSM 11109	4.50E-005	BamE	YP_004371862.1	SmpA protein
Desulfobacca acetoxidans DSM 11109	1.80E-169	Omp85	YP_004371714.1	outer membrane protein assembly complex, YaeT protein
Desulfobacca acetoxidans DSM 11109	7.60E-008	Omp85	YP_004371088.1	surface antigen (D15)
Desulfobacterium autotrophicum HRM2	1.00E-012	BamD	YP_002602507.1	putative DNA uptake lipoprotein
Desulfobacterium autotrophicum HRM2	7.30E-005	BamD	YP_002604956.1	TPR repeat family protein
Desulfobacterium autotrophicum HRM2	7.90E-005	BamD	YP_002603523.1	TPR domain protein
Desulfobacterium autotrophicum HRM2	6.00E-143	Omp85	YP_002603503.1	outer membrane protein (putative surface antigen)
Desulfobacterium autotrophicum HRM2	6.80E-009	Omp85	YP_002603845.1	putative outer membrane surface antigen protein
Desulfobulbus propionicus DSM 2032	8.70E-016	BamD	YP_004196379.1	outer membrane assembly lipoprotein YfiO
Desulfobulbus propionicus DSM 2032	9.50E-005	BamD	YP_004193798.1	TPR repeat-containing protein
Desulfobulbus propionicus DSM 2032	3.50E-006	BamE	YP_004196300.1	hypothetical protein Despr_2875
Desulfobulbus propionicus DSM 2032	4.10E-180	Omp85	YP_004194043.1	outer membrane protein assembly complex, YaeT protein
Desulfobulbus propionicus DSM 2032	2.50E-006	Omp85	YP_004196024.1	surface antigen (D15)
Desulfococcus oleovorans Hxd3	2.30E-017	BamD	YP_001529936.1	DNA uptake lipoprotein-like protein
Desulfococcus oleovorans Hxd3	8.60E-006	BamD	YP_001528935.1	response regulator receiver protein
Desulfococcus oleovorans Hxd3	2.70E-005	BamD	YP_001529644.1	branched-chain amino acid ABC transporter periplasmic component-like protein
Desulfococcus oleovorans Hxd3	3.10E-005	BamD	YP_001529463.1	hypothetical protein Dole_1582
Desulfococcus oleovorans Hxd3	3.50E-005	BamE	YP_001530928.1	hypothetical protein Dole_3048
Desulfococcus oleovorans Hxd3	1.90E-166	Omp85	YP_001530724.1	surface antigen (D15)
Desulfohalobium retbaense DSM 5692	1.30E-014	BamD	YP_003198768.1	outer membrane assembly lipoprotein YfiO
Desulfohalobium retbaense DSM 5692	6.40E-007	BamE	YP_003197823.1	hypothetical protein Dret_0957
Desulfohalobium retbaense DSM 5692	3.20E-243	Omp85	YP_003199093.1	outer membrane protein assembly complex, YaeT protein
Desulfohalobium retbaense DSM 5692	1.20E-006	Omp85	YP_003197308.1	surface antigen (D15)

Desulfomicrobium baculatum DSM 4028	9.30E-016	BamD	YP_003157659.1	outer membrane assembly lipoprotein YfiO
Desulfomicrobium baculatum DSM 4028	3.90E-005	BamE	YP_003158026.1	lipoprotein
Desulfomicrobium baculatum DSM 4028	1.20E-246	Omp85	YP_003159869.1	outer membrane protein assembly complex, YaeT protein
Desulfomicrobium baculatum DSM 4028	2.30E-005	Omp85	YP_003156956.1	surface antigen (D15)
Desulfotalea psychrophila LSv54	4.30E-018	BamD	YP_064544.1	hypothetical protein DP0808
Desulfotalea psychrophila LSv54	9.30E-109	Omp85	YP_064017.1	outer membrane protein
Desulfotalea psychrophila LSv54	8.50E-005	Omp85	YP_065180.1	hypothetical protein DP1444
Desulfovibrio aespoeensis Aspo-2	4.90E-014	BamD	YP_004121580.1	outer membrane assembly lipoprotein YfiO
Desulfovibrio aespoeensis Aspo-2	9.20E-005	BamE	YP_004122495.1	CHASE4 domain-containing protein
Desulfovibrio aespoeensis Aspo-2	1.10E-184	Omp85	YP_004121159.1	outer membrane protein assembly complex protein YaeT
Desulfovibrio alaskensis G20	1.10E-014	BamD	YP_388557.1	outer membrane assembly lipoprotein YfiO
Desulfovibrio alaskensis G20	0.00E+000	Omp85	YP_387864.1	outer membrane protein assembly complex, YaeT protein
Desulfovibrio desulfuricans subsp. desulfuricans str. ATC	2.80E-017	BamD	YP_002479783.1	outer membrane assembly lipoprotein YfiO
Desulfovibrio desulfuricans subsp. desulfuricans str. ATC	1.10E-193	Omp85	YP_002479198.1	outer membrane protein assembly complex, YaeT protein
Desulfovibrio desulfuricans subsp. desulfuricans str. ATC	8.00E-007	Omp85	YP_002479055.1	surface antigen (D15)
Desulfovibrio magneticus RS-1	1.10E-015	BamD	YP_002953544.1	hypothetical protein DMR_21670
Desulfovibrio magneticus RS-1	2.40E-188	Omp85	YP_002955231.1	hypothetical protein DMR_38540
Desulfovibrio magneticus RS-1	6.30E-006	Omp85	YP_002954928.1	hypothetical protein DMR_35510
Desulfovibrio salexigens DSM 2638	9.20E-015	BamD	YP_002991420.1	outer membrane assembly lipoprotein YfiO
Desulfovibrio salexigens DSM 2638	3.50E-005	BamD	YP_002991976.1	hypothetical protein Desal_2381
Desulfovibrio salexigens DSM 2638	1.90E-233	Omp85	YP_002992119.1	outer membrane protein assembly complex, YaeT protein
Desulfovibrio vulgaris DP4	1.80E-013	BamD	YP_966770.1	lipoprotein
Desulfovibrio vulgaris DP4	8.50E-259	Omp85	YP_966339.1	surface antigen (D15)
Desulfovibrio vulgaris DP4	3.10E-007	Omp85	YP_966576.1	surface antigen (D15)
Desulfovibrio vulgaris str. 'Miyazaki F'	7.10E-014	BamD	YP_002434825.1	lipoprotein
Desulfovibrio vulgaris str. 'Miyazaki F'	1.80E-259	Omp85	YP_002437401.1	outer membrane protein assembly complex, YaeT protein
Desulfovibrio vulgaris str. 'Miyazaki F'	2.40E-006	Omp85	YP_002437310.1	surface antigen (D15)
Desulfovibrio vulgaris str. Hildenborough	1.80E-013	BamD	YP_011054.1	competence protein
Desulfovibrio vulgaris str. Hildenborough	1.90E-247	Omp85	YP_011586.2	OMP85 family outer membrane protein
Desulfovibrio vulgaris str. Hildenborough	2.90E-007	Omp85	YP_011315.1	OMP85 family outer membrane protein
Desulfurivibrio alkaliphilus AHT2	1.40E-014	BamD	YP_003690951.1	outer membrane assembly lipoprotein YfiO
Desulfurivibrio alkaliphilus AHT2	1.50E-005	BamD	YP_003690510.1	hypothetical protein
Desulfurivibrio alkaliphilus AHT2	5.40E-183	Omp85	YP_003690623.1	outer membrane protein assembly complex, YaeT protein
Desulfurivibrio alkaliphilus AHT2	4.50E-008	Omp85	YP_003691426.1	surface antigen (D15)
Geobacter bemidjiensis Bem	1.10E-012	BamD	YP_002137300.1	outer membrane protein assembly lipoprotein YfiO
Geobacter bemidjiensis Bem	9.80E-006	BamD	YP_002140778.2	peptidoglycan L,D-transpeptidase lipoprotein
Geobacter bemidjiensis Bem	3.00E-005	BamD	YP_002139406.1	lytic transglycosylase domain-containing protein
Geobacter bemidjiensis Bem	6.80E-005	BamD	YP_002140391.1	lipoprotein

Geobacter bemidjiensis Bem	3.40E-131	Omp85	YP_002137658.2	outer membrane protein assembly complex protein YaeT
Geobacter bemidjiensis Bem	8.80E-007	Omp85	YP_002139973.1	outer membrane surface protein
Geobacter lovleyi SZ	2.50E-016	BamD	YP_001953178.1	outer membrane assembly lipoprotein YfiO
Geobacter lovleyi SZ	1.70E-005	BamD	YP_001951255.1	hypothetical protein Glov_1012
Geobacter lovleyi SZ	8.10E-005	BamE	YP_001951007.1	outer membrane chaperone Skp
Geobacter lovleyi SZ	1.30E-127	Omp85	YP_001951006.1	outer membrane protein assembly complex, YaeT protein
Geobacter metallireducens GS-15	3.10E-013	BamD	YP_385954.1	lipoprotein
Geobacter metallireducens GS-15	1.70E-005	BamD	YP_384465.1	tetratricopeptide TPR_4
Geobacter metallireducens GS-15	3.20E-005	BamD	YP_384986.1	TPR repeat-containing protein
Geobacter metallireducens GS-15	7.30E-005	BamD	YP_386165.1	hypothetical protein Gmet_3227
Geobacter metallireducens GS-15	1.50E-147	Omp85	YP_385307.1	surface antigen (D15)
Geobacter metallireducens GS-15	9.60E-007	Omp85	YP_385751.1	surface antigen (D15)
Geobacter sp. FRC-32	4.00E-011	BamD	YP_002536221.1	outer membrane assembly lipoprotein YfiO
Geobacter sp. FRC-32	7.00E-007	BamD	YP_002535606.1	ErfK/YbiS/YcfS/YnhG family protein
Geobacter sp. FRC-32	1.20E-005	BamD	YP_002538427.1	hypothetical protein Geob_2983
Geobacter sp. FRC-32	6.10E-005	BamD	YP_002538836.1	Lytic transglycosylase catalytic
Geobacter sp. FRC-32	7.70E-005	BamD	YP_002536767.1	chromosome segregation ATPase-like protein
Geobacter sp. FRC-32	3.60E-006	BamE	YP_002536626.1	hypothetical protein Geob_1165
Geobacter sp. FRC-32	2.10E-126	Omp85	YP_002537553.1	outer membrane protein assembly complex, YaeT protein
Geobacter sp. FRC-32	5.70E-005	Omp85	YP_002535819.1	surface antigen (D15)
Geobacter sp. M18	5.30E-015	BamD	YP_004200601.1	outer membrane assembly lipoprotein YfiO
Geobacter sp. M18	1.50E-144	Omp85	YP_004197490.1	outer membrane protein assembly complex, YaeT protein
Geobacter sp. M18	4.00E-007	Omp85	YP_004199890.1	surface antigen (D15)
Geobacter sp. M21	8.80E-013	BamD	YP_003020330.1	outer membrane assembly lipoprotein YfiO
Geobacter sp. M21	1.50E-005	BamD	YP_003023856.1	ErfK/YbiS/YcfS/YnhG family protein
Geobacter sp. M21	2.50E-005	BamD	YP_003021437.1	lytic transglycosylase catalytic
Geobacter sp. M21	1.60E-131	Omp85	YP_003023203.1	hypothetical protein GM21_3421
Geobacter sp. M21	1.60E-006	Omp85	YP_003020906.1	surface antigen (D15)
Geobacter sulfurreducens PCA	9.00E-013	BamD	NP_951559.1	lipoprotein
Geobacter sulfurreducens PCA	1.20E-007	BamD	NP_951243.1	lipoprotein
Geobacter sulfurreducens PCA	1.20E-005	BamD	NP_952483.1	hypothetical protein GSU1432
Geobacter sulfurreducens PCA	8.60E-136	Omp85	NP_953317.1	outer membrane protein
Geobacter uraniireducens Rf4	6.40E-014	BamD	YP_001232724.1	DNA uptake lipoprotein-like protein
Geobacter uraniireducens Rf4	3.50E-006	BamD	YP_001230919.1	ErfK/YbiS/YcfS/YnhG family protein
Geobacter uraniireducens Rf4	8.30E-006	BamD	YP_001231119.1	TPR repeat-containing protein
Geobacter uraniireducens Rf4	5.80E-005	BamD	YP_001231334.1	TPR repeat-containing protein
Geobacter uraniireducens Rf4	9.00E-005	BamD	YP_001232418.1	tetratricopeptide domain-containing protein
Geobacter uraniireducens Rf4	7.80E-126	Omp85	YP_001231971.1	surface antigen (D15)

Haliangium ochraceum DSM 14365	4.90E-024	BamB	YP_003268123.1	pyrrolo-quinoline quinone
Haliangium ochraceum DSM 14365	1.70E-007	BamB	YP_003269343.1	pyrrolo-quinoline quinone
Haliangium ochraceum DSM 14365	2.90E-005	BamB	YP_003265792.1	WD40 repeat-containing protein
Haliangium ochraceum DSM 14365	4.10E-015	BamD	YP_003269025.1	outer membrane assembly lipoprotein YfiO
Haliangium ochraceum DSM 14365	1.50E-109	Omp85	YP_003268364.1	outer membrane protein assembly complex, YaeT protein
Haliangium ochraceum DSM 14365	8.80E-009	Omp85	YP_003268088.1	surface antigen (D15)
Haliangium ochraceum DSM 14365	8.00E-007	Omp85	YP_003269643.1	surface antigen (D15)
Hippea maritima DSM 10411	1.50E-012	BamB	YP_004340351.1	hypothetical protein Hipma_1335
Hippea maritima DSM 10411	9.00E-015	BamD	YP_004340303.1	outer membrane assembly lipoprotein YfiO
Hippea maritima DSM 10411	6.30E-094	Omp85	YP_004340308.1	outer membrane protein assembly complex, YaeT protein
Lawsonia intracellularis PHE/MN1-00	1.30E-013	BamD	YP_594742.1	DNA uptake lipoprotein
Lawsonia intracellularis PHE/MN1-00	1.20E-005	BamD	YP_595468.1	Outer membrane protein and related peptidoglycan-associated (lipo)proteins
Lawsonia intracellularis PHE/MN1-00	8.80E-215	Omp85	YP_595399.1	Outer membrane protein/protective antigen OMA87
Lawsonia intracellularis PHE/MN1-00	3.50E-006	Omp85	YP_595063.1	Outer membrane protein
Myxococcus fulvus HW-1	4.10E-015	BamB	YP_004668368.1	PQQ repeat-containing protein
Myxococcus fulvus HW-1	2.80E-010	BamB	YP_004663248.1	putative lipoprotein
Myxococcus fulvus HW-1	2.40E-006	BamB	YP_004668747.1	hypothetical protein LILAB_28940
Myxococcus fulvus HW-1	1.50E-005	BamB	YP_004668334.1	PQQ repeat-containing protein
Myxococcus fulvus HW-1	9.20E-005	BamB	YP_004664905.1	putative lipoprotein
Myxococcus fulvus HW-1	4.50E-017	BamD	YP_004666537.1	putative competence lipoprotein ComL
Myxococcus fulvus HW-1	3.00E-107	Omp85	YP_004669277.1	OMP85 family outer membrane protein
Myxococcus fulvus HW-1	6.50E-009	Omp85	YP_004670214.1	OMP85 family outer membrane protein
Myxococcus fulvus HW-1	3.20E-005	Omp85	YP_004665828.1	OMP85 family outer membrane protein
Myxococcus xanthus DK 1622	7.60E-014	BamB	YP_631950.1	PQQ repeat-containing protein
Myxococcus xanthus DK 1622	6.40E-011	BamB	YP_629749.1	PQQ repeat-containing protein
Myxococcus xanthus DK 1622	7.30E-011	BamB	YP_629770.1	lipoprotein
Myxococcus xanthus DK 1622	2.10E-006	BamB	YP_632331.1	hypothetical protein MXAN_4156
Myxococcus xanthus DK 1622	2.70E-005	BamB	YP_631914.1	PQQ repeat-containing protein
Myxococcus xanthus DK 1622	3.20E-005	BamB	YP_632305.1	WD domain-/G-beta repeat-containing protein
Myxococcus xanthus DK 1622	2.80E-016	BamD	YP_630233.1	competence lipoprotein ComL
Myxococcus xanthus DK 1622	3.10E-005	BamD	YP_631209.1	lipoprotein
Myxococcus xanthus DK 1622	1.20E-090	Omp85	YP_632890.1	OMP85 family outer membrane protein
Myxococcus xanthus DK 1622	1.10E-008	Omp85	YP_633900.1	OMP85 family outer membrane protein
Myxococcus xanthus DK 1622	3.70E-005	Omp85	YP_634587.1	OMP85 family outer membrane protein
Pelobacter carbinolicus DSM 2380	4.30E-013	BamD	YP_357792.1	TPR domain-containing protein
Pelobacter carbinolicus DSM 2380	1.50E-006	BamD	YP_355840.1	hypothetical protein Pcar_0410
Pelobacter carbinolicus DSM 2380	6.40E-006	BamD	YP_356927.1	N-acetylmuramoyl-L-alanine amidase

Pelobacter carbinolicus DSM 2380	4.70E-006	BamE	YP_842177.1	hypothetical protein Pcar_3236
Pelobacter carbinolicus DSM 2380	1.10E-125	Omp85	YP_356670.1	putative outer membrane protein
Pelobacter carbinolicus DSM 2380	1.90E-006	Omp85	YP_355619.1	hypothetical protein Pcar_0188
Pelobacter propionicus DSM 2379	1.60E-015	BamD	YP_902305.1	hypothetical protein Ppro_2643
Pelobacter propionicus DSM 2379	9.40E-007	BamD	YP_901519.1	hypothetical protein Ppro_1849
Pelobacter propionicus DSM 2379	8.90E-006	BamD	YP_900260.1	ErfK/YbiS/YcfS/YnhG family protein
Pelobacter propionicus DSM 2379	2.40E-005	BamE	YP_900637.1	hypothetical protein Ppro_0950
Pelobacter propionicus DSM 2379	7.40E-101	Omp85	YP_902672.1	surface antigen (D15)
Pelobacter propionicus DSM 2379	1.30E-006	Omp85	YP_902916.1	surface antigen (D15)
Sorangium cellulosum 'So ce 56'	1.60E-010	BamB	YP_001614232.1	hypothetical protein sce3592
Sorangium cellulosum 'So ce 56'	3.40E-008	BamB	YP_001614278.1	hypothetical protein sce3638
Sorangium cellulosum 'So ce 56'	4.10E-007	BamB	YP_001616046.1	polyvinylalcohol dehydrogenase
Sorangium cellulosum 'So ce 56'	6.10E-005	BamB	YP_001614270.1	WD repeat-containing protein
Sorangium cellulosum 'So ce 56'	2.30E-011	BamD	YP_001614236.1	hypothetical protein sce3596
Sorangium cellulosum 'So ce 56'	3.30E-112	Omp85	YP_001615093.1	surface antigen
Sorangium cellulosum 'So ce 56'	5.40E-007	Omp85	YP_001611742.1	hypothetical protein sce1105
Sorangium cellulosum 'So ce 56'	4.50E-005	Omp85	YP_001611004.1	surface antigen
Syntrophobacter fumaroxidans MPOB	1.60E-014	BamD	YP_845832.1	ComL family lipoprotein
Syntrophobacter fumaroxidans MPOB	7.80E-005	BamD	YP_845166.1	extracellular ligand-binding receptor
Syntrophobacter fumaroxidans MPOB	1.30E-166	Omp85	YP_847850.1	surface antigen
Syntrophobacter fumaroxidans MPOB	2.60E-008	Omp85	YP_844891.1	surface antigen

Arcobacter butzleri RM4018	4.60E-006	BamD	YP_001489527.1	hypothetical protein Abu_0591
Arcobacter butzleri RM4018	3.90E-031	Omp85	YP_001490140.1	outer membrane surface antigen protein
Arcobacter nitrofigilis DSM 7299	2.90E-009	BamB	YP_003656940.1	PQQ-dependent dehydrogenase
Arcobacter nitrofigilis DSM 7299	2.10E-005	BamD	YP_003654997.1	hypothetical protein Arnit_0826
Arcobacter nitrofigilis DSM 7299	1.70E-043	Omp85	YP_003655889.1	outer membrane protein assembly complex, YaeT protein
Campylobacter concisus 13826	7.20E-006	BamD	YP_001467200.1	tRNA (guanine-N(1)-)-methyltransferase
Campylobacter concisus 13826	1.10E-063	Omp85	YP_001466370.1	outer membrane protein assembly complex, YaeT protein
Campylobacter curvus 525.92	1.80E-006	BamD	YP_001407991.1	hypothetical protein CCV52592_1590
Campylobacter curvus 525.92	2.50E-051	Omp85	YP_001408790.1	outer membrane protein assembly complex, YaeT protein
Campylobacter fetus subsp. fetus 82-40	3.10E-006	BamD	YP_892222.1	hypothetical protein CFF8240_1061
Campylobacter fetus subsp. fetus 82-40	3.50E-055	Omp85	YP_892635.1	OMP85 family outer membrane protein
Campylobacter hominis ATCC BAA-381	3.80E-005	BamB	YP_001407163.1	hypothetical protein CHAB381_1640
Campylobacter hominis ATCC BAA-381	2.40E-006	BamD	YP_001406138.1	competence lipoprotein
Campylobacter hominis ATCC BAA-381	8.50E-005	BamE	YP_001406364.1	Plp4
Campylobacter hominis ATCC BAA-381	2.60E-068	Omp85	YP_001407198.1	outer membrane protein assembly complex, YaeT protein
Campylobacter jejuni RM1221	2.30E-006	BamD	YP_179205.1	lipoprotein
Campylobacter jejuni RM1221	2.80E-047	Omp85	YP_178148.1	OMP85 family outer membrane protein
Campylobacter jejuni subsp. doylei 269.97	6.40E-006	BamD	YP_001397810.1	putative lipoprotein
Campylobacter jejuni subsp. doylei 269.97	4.60E-046	Omp85	YP_001397388.1	OMP85 family outer membrane protein
Campylobacter jejuni subsp. jejuni 81-176	3.80E-006	BamD	YP_001000752.1	putative lipoprotein
Campylobacter jejuni subsp. jejuni 81-176	1.70E-047	Omp85	YP_999853.1	OMP85 family outer membrane protein
Campylobacter jejuni subsp. jejuni 81116	4.00E-006	BamD	YP_001482591.1	putative lipoprotein
Campylobacter jejuni subsp. jejuni 81116	1.70E-047	Omp85	YP_001481698.1	OMP85 family outer membrane protein
Campylobacter jejuni subsp. jejuni ICDCCJ07001	3.80E-006	BamD	YP_004066562.1	putative lipoprotein
Campylobacter jejuni subsp. jejuni ICDCCJ07001	9.90E-049	Omp85	YP_004065735.1	outer membrane protein, OMP85 family
Campylobacter jejuni subsp. jejuni NCTC 11168	3.80E-006	BamD	YP_002344467.1	putative lipoprotein
Campylobacter jejuni subsp. jejuni NCTC 11168	1.00E-045	Omp85	YP_002343589.1	outer membrane protein
Campylobacter lari RM2100	5.50E-006	BamD	YP_002575535.1	hypothetical protein Cla_0960
Campylobacter lari RM2100	5.70E-005	BamD	YP_002575273.1	amino acid ABC transporter substrate-binding protein
Campylobacter lari RM2100	2.60E-046	Omp85	YP_002574829.1	outer membrane protein
Helicobacter acinonychis str. Sheeba	9.70E-008	BamD	YP_663921.1	competence lipoprotein
Helicobacter acinonychis str. Sheeba	1.50E-022	Omp85	YP_664622.1	protective surface antigen
Helicobacter bizzozeronii CIII-1	1.10E-015	Omp85	YP_004608529.1	outer membrane protein assembly factor YaeT
Helicobacter felis ATCC 49179	1.40E-005	BamD	YP_004073790.1	putative lipoprotein
Helicobacter felis ATCC 49179	2.80E-005	BamD	YP_004073509.1	paralysed flagellum protein, PflA
Helicobacter felis ATCC 49179	7.80E-026	Omp85	YP_004073252.1	putative bacterial surface antigen
Helicobacter hepaticus ATCC 51449	7.30E-007	BamD	NP_860375.1	hypothetical protein HH0844
Helicobacter hepaticus ATCC 51449	1.30E-057	Omp85	NP_860523.1	outer membrane protein

Helicobacter mustelae 12198	3.10E-006	BamD	YP_003516301.1	lipoprotein
Helicobacter mustelae 12198	2.50E-012	Omp85	YP_003516293.1	bacterial surface antigen
Helicobacter pylori 26695	9.70E-007	BamD	NP_208169.1	competence lipoprotein (comL)
Helicobacter pylori 26695	1.40E-022	Omp85	NP_207449.1	protective surface antigen D15
Helicobacter pylori B38	9.70E-007	BamD	YP_003058023.1	hypothetical protein HELPY_1365
Helicobacter pylori B38	2.40E-024	Omp85	YP_003057463.1	surface antigen protein, signal peptide
Helicobacter pylori B8	8.60E-007	BamD	YP_003729568.1	hypothetical protein HPB8_1547
Helicobacter pylori B8	1.20E-010	Omp85	YP_003728879.1	putative outer membrane protein
Helicobacter pylori G27	1.10E-006	BamD	YP_002266937.1	competence lipoprotein
Helicobacter pylori G27	1.40E-016	Omp85	YP_002266243.1	protective surface antigen D15
Helicobacter pylori HPAG1	9.70E-007	BamD	YP_628065.1	competence lipoprotein
Helicobacter pylori HPAG1	1.70E-022	Omp85	YP_627381.1	protective surface antigen D15
Helicobacter pylori J99	6.70E-007	BamD	NP_224010.1	hypothetical protein jhp1292
Helicobacter pylori J99	1.40E-019	Omp85	NP_223318.1	protective surface antigen D15
Helicobacter pylori P12	9.70E-007	BamD	YP_002302002.1	competence lipoprotein ComL
Helicobacter pylori P12	1.90E-024	Omp85	YP_002301301.1	protective surface antigen D15
Helicobacter pylori PeCan4	9.70E-007	BamD	YP_003927618.1	hypothetical protein HPPC_06800
Helicobacter pylori PeCan4	2.00E-015	Omp85	YP_003926982.1	protective surface antigen D15
Helicobacter pylori Shi470	9.70E-007	BamD	YP_001910855.1	competence lipoprotein
Helicobacter pylori Shi470	2.60E-013	Omp85	YP_001910183.1	protective surface antigen D15
Helicobacter pylori SJM180	5.60E-007	BamD	YP_003929293.1	hypothetical protein HPSJM_07080
Helicobacter pylori SJM180	3.90E-011	Omp85	YP_003928576.1	protective surface antigen D15
Nautilia profundicola AmH	5.80E-005	BamB	YP_002607035.1	putative lipoprotein
Nautilia profundicola AmH	9.50E-005	BamD	YP_002607234.1	putative lipoprotein
Nautilia profundicola AmH	2.10E-061	Omp85	YP_002607869.1	outer membrane protein, OMP85 family
Nitratifractor salsuginis DSM 16511	2.40E-006	BamB	YP_004168995.1	hypothetical protein Nitsa_2004
Nitratifractor salsuginis DSM 16511	4.10E-053	Omp85	YP_004167301.1	outer membrane protein assembly complex, yaet protein
Nitratifractor salsuginis DSM 16511	1.30E-005	Omp85	YP_004168115.1	surface antigen (d15)
Nitratiruptor sp. SB155-2	7.20E-006	BamD	YP_001356814.1	hypothetical protein NIS_1349
Nitratiruptor sp. SB155-2	7.40E-084	Omp85	YP_001355878.1	hypothetical protein NIS_0407
Sulfuricurvum kujiense DSM 16994	1.20E-005	BamD	YP_004059171.1	outer membrane assembly lipoprotein yfio
Sulfuricurvum kujiense DSM 16994	3.10E-005	BamD	YP_004061375.1	hypothetical protein Sulku_2516
Sulfuricurvum kujiense DSM 16994	2.10E-054	Omp85	YP_004059299.1	outer membrane protein assembly complex, yaet protein
Sulfuricurvum kujiense DSM 16994	1.40E-005	Omp85	YP_004058887.1	surface antigen (d15)
Sulfurimonas autotrophica DSM 16294	5.80E-005	BamD	YP_003892853.1	DNA uptake lipoprotein
Sulfurimonas autotrophica DSM 16294	1.60E-042	Omp85	YP_003891747.1	outer membrane protein assembly complex, YaeT protein
Sulfurimonas denitrificans DSM 1251	4.00E-005	BamE	YP_393909.1	OmpA/MotB
Sulfurimonas denitrificans DSM 1251	9.20E-041	Omp85	YP_393174.1	surface antigen (D15)

Sulfurospirillum deleyianum DSM 6946	8.60E-007	BamD	YP_003303925.1	outer membrane assembly lipoprotein YfiO
Sulfurospirillum deleyianum DSM 6946	2.70E-005	BamE	YP_003305315.1	hypothetical protein Sdel_2268
Sulfurospirillum deleyianum DSM 6946	9.90E-060	Omp85	YP_003303304.1	outer membrane protein assembly complex, YaeT protein
Sulfurovum sp. NBC37-1	3.20E-005	BamB	YP_001357737.1	hypothetical protein SUN_0420
Sulfurovum sp. NBC37-1	2.50E-006	BamD	YP_001359211.1	hypothetical protein SUN_1907
Sulfurovum sp. NBC37-1	2.40E-059	Omp85	YP_001359297.1	hypothetical protein SUN_1997
Sulfurovum sp. NBC37-1	2.40E-005	Omp85	YP_001358883.1	hypothetical protein SUN_1576

Acidithiobacillus caldus SM-1	5.30E-046	BamB	YP_004748721.1	pyrrolo-quinoline quinone
Acidithiobacillus caldus SM-1	2.50E-008	BamB	YP_004748944.1	tetrathionate hydrolase TetH
Acidithiobacillus caldus SM-1	9.30E-007	BamB	YP_004748254.1	hypothetical protein Atc_0905
Acidithiobacillus caldus SM-1	3.00E-060	BamD	YP_004749784.1	component of the lipoprotein assembly complex (forms a complex with YaeT, YfgL, and NlpB)
Acidithiobacillus caldus SM-1	9.70E-035	BamD	YP_004750039.1	component of the lipoprotein assembly complex (forms a complex with YaeT, YfgL, and NlpB)
Acidithiobacillus caldus SM-1	5.60E-017	BamE	YP_004749937.1	outer membrane lipoprotein SmpA
Acidithiobacillus caldus SM-1	6.20E-186	Omp85	YP_004748198.1	outer membrane protein assembly factor YaeT
Acidithiobacillus caldus SM-1	5.20E-006	Omp85	YP_004748650.1	surface antigen (D15)
Acidithiobacillus ferrivorans SS3	3.40E-060	BamB	YP_004783986.1	outer membrane assembly lipoprotein YfgL
Acidithiobacillus ferrivorans SS3	2.00E-008	BamB	YP_004782606.1	pyrrolo-quinoline quinone repeat-containing protein
Acidithiobacillus ferrivorans SS3	4.10E-008	BamB	YP_004785010.1	pyrrolo-quinoline quinone repeat-containing protein
Acidithiobacillus ferrivorans SS3	7.70E-064	BamD	YP_004783161.1	outer membrane assembly lipoprotein YfiO
Acidithiobacillus ferrivorans SS3	1.80E-005	BamD	YP_004782697.1	tol-pal system protein YbgF
Acidithiobacillus ferrivorans SS3	3.80E-015	BamE	YP_004785153.1	SmpA/OmlA domain-containing protein
Acidithiobacillus ferrivorans SS3	2.20E-172	Omp85	YP_004783785.1	outer membrane protein assembly complex, YaeT protein
Acidithiobacillus ferrivorans SS3	1.10E-005	Omp85	YP_004784199.1	surface antigen (D15)
Acidithiobacillus ferrooxidans ATCC 23270	2.70E-052	BamB	YP_002426345.1	PQQ enzyme repeat domain protein
Acidithiobacillus ferrooxidans ATCC 23270	1.30E-009	BamB	YP_002426649.1	PQQ enzyme repeat domain protein
Acidithiobacillus ferrooxidans ATCC 23270	5.00E-007	BamB	YP_002424542.1	tetrathionate hydrolase
Acidithiobacillus ferrooxidans ATCC 23270	9.90E-007	BamB	YP_002426388.1	PQQ enzyme repeat protein
Acidithiobacillus ferrooxidans ATCC 23270	1.90E-055	BamD	YP_002424926.1	competence lipoprotein ComL, putative
Acidithiobacillus ferrooxidans ATCC 23270	9.80E-006	BamD	YP_002424584.1	hypothetical protein AFE_0070
Acidithiobacillus ferrooxidans ATCC 23270	4.20E-019	BamE	YP_002424787.1	lipoprotein, SmpA/OmlA family
Acidithiobacillus ferrooxidans ATCC 23270	5.20E-175	Omp85	YP_002425880.1	outer membrane protein, OMP85 family
Acidithiobacillus ferrooxidans ATCC 23270	2.00E-005	Omp85	YP_002426365.1	outer membrane protein, OMP85 family
Acinetobacter baumannii AB0057	2.00E-056	BamB	YP_002318011.1	outer membrane assembly lipoprotein YfgL
Acinetobacter baumannii AB0057	2.00E-069	BamD	YP_002318281.1	putative competence protein
Acinetobacter baumannii AB0057	1.90E-017	BamE	YP_002318333.1	outer membrane lipoprotein
Acinetobacter baumannii AB0057	3.00E-160	Omp85	YP_002319666.1	outer membrane protein assembly complex
Acinetobacter baumannii AB0057	2.30E-007	Omp85	YP_002319812.1	hypothetical protein AB57_2465
Acinetobacter baumannii AB307-0294	2.00E-056	BamB	YP_002326916.1	outer membrane assembly lipoprotein YfgL
Acinetobacter baumannii AB307-0294	2.00E-069	BamD	YP_002326669.1	Competence lipoprotein comL precursor
Acinetobacter baumannii AB307-0294	1.90E-017	BamE	YP_002326618.1	outer membrane lipoprotein omlA precursor
Acinetobacter baumannii AB307-0294	3.00E-160	Omp85	YP_002325380.1	outer membrane protein assembly complex, YaeT protein
Acinetobacter baumannii AB307-0294	2.30E-007	Omp85	YP_002325237.1	Surface antigen family protein
Acinetobacter baumannii ACICU	2.00E-015	BamB	YP_001845174.1	WD-40 repeat-containing protein
Acinetobacter baumannii ACICU	4.30E-005	BamB	YP_001845347.1	glucose dehydrogenase

Acinetobacter baumannii ACICU	2.00E-069	BamD	YP_001845452.1	DNA uptake lipoprotein
Acinetobacter baumannii ACICU	1.90E-017	BamE	YP_001845504.1	small protein A
Acinetobacter baumannii ACICU	9.80E-005	BamE	YP_001845653.1	small protein A
Acinetobacter baumannii ACICU	3.00E-160	Omp85	YP_001846751.1	outer membrane protein/protective antigen OMA87
Acinetobacter baumannii ACICU	2.20E-007	Omp85	YP_001846991.1	outer membrane protein
Acinetobacter baumannii ATCC 17978	2.00E-030	BamB	YP_001083560.1	hypothetical protein A1S_0505
Acinetobacter baumannii ATCC 17978	6.00E-059	BamD	YP_001083876.1	putative competence protein (ComL)
Acinetobacter baumannii ATCC 17978	5.60E-163	Omp85	YP_001084998.1	putative outer membrane protein
Acinetobacter baumannii ATCC 17978	2.20E-007	Omp85	YP_001085161.1	putative outer membrane protein
Acinetobacter baumannii AYE	2.00E-056	BamB	YP_001715033.1	hypothetical protein ABAYE3260
Acinetobacter baumannii AYE	2.00E-069	BamD	YP_001714768.1	competence protein (ComL)
Acinetobacter baumannii AYE	1.90E-017	BamE	YP_001714718.1	outer membrane lipoprotein
Acinetobacter baumannii AYE	1.70E-164	Omp85	YP_001713480.1	outer membrane protein
Acinetobacter baumannii AYE	2.30E-007	Omp85	YP_001713338.1	hypothetical protein ABAYE1429
Acinetobacter baumannii SDF	1.70E-054	BamB	YP_001708138.1	hypothetical protein ABSDF2997
Acinetobacter baumannii SDF	2.00E-069	BamD	YP_001707834.1	competence protein (ComL)
Acinetobacter baumannii SDF	1.90E-017	BamE	YP_001707766.1	outer membrane lipoprotein
Acinetobacter baumannii SDF	3.00E-165	Omp85	YP_001707071.1	outer membrane protein
Acinetobacter baumannii SDF	2.20E-007	Omp85	YP_001707007.1	hypothetical protein ABSDF1607
Acinetobacter sp. ADP1	1.70E-050	BamB	YP_045310.1	PQQ repeat-containing protein
Acinetobacter sp. ADP1	2.50E-005	BamB	YP_046380.1	quinate/shikimate dehydrogenase, pyrroloquinoline-quinone] (NAD(P)-independent quinate dehydrogenase)
Acinetobacter sp. ADP1	6.80E-005	BamB	YP_047514.1	glucose dehydrogenase
Acinetobacter sp. ADP1	4.60E-067	BamD	YP_047442.1	competence protein (ComL)
Acinetobacter sp. ADP1	5.70E-019	BamE	YP_045627.1	outer membrane lipoprotein
Acinetobacter sp. ADP1	8.40E-006	BamE	YP_046308.1	antigen
Acinetobacter sp. ADP1	3.70E-164	Omp85	YP_046066.1	outer membrane protein
Acinetobacter sp. ADP1	1.10E-007	Omp85	YP_047000.1	outer membrane protein
Acinetobacter sp. DR1	2.00E-054	BamB	YP_003733640.1	outer membrane assembly lipoprotein YfgL
Acinetobacter sp. DR1	4.90E-071	BamD	YP_003733358.1	DNA uptake lipoprotein
Acinetobacter sp. DR1	1.70E-017	BamE	YP_003733308.1	Outer membrane lipoprotein omlA precursor
Acinetobacter sp. DR1	9.40E-166	Omp85	YP_003731732.1	putative outer membrane protein
Acinetobacter sp. DR1	2.60E-007	Omp85	YP_003731564.1	Surface antigen family protein
Actinobacillus pleuropneumoniae serovar 3 str. JL03	6.00E-131	BamD	YP_001652140.1	putative lipoprotein
Actinobacillus pleuropneumoniae serovar 3 str. JL03	8.90E-068	BamE	YP_001651480.1	small protein A
Actinobacillus pleuropneumoniae serovar 3 str. JL03	2.90E-181	Omp85	YP_001651464.1	outer membrane protein D-15
Actinobacillus pleuropneumoniae serovar 3 str. JL03	3.60E-006	Omp85	YP_001651839.1	putative outer membrane protein
Actinobacillus pleuropneumoniae serovar 5b str. L20	6.00E-131	BamD	YP_001053816.1	putative lipoprotein

Actinobacillus pleuropneumoniae serovar 5b str. L20	8.90E-068	BamE	YP_001053137.1	small protein A
Actinobacillus pleuropneumoniae serovar 5b str. L20	1.70E-183	Omp85	YP_001053120.1	protective surface antigen D15 precursor
Actinobacillus pleuropneumoniae serovar 5b str. L20	3.60E-006	Omp85	YP_001053530.1	hypothetical protein APL_0829
Actinobacillus pleuropneumoniae serovar 7 str. AP76	1.50E-130	BamD	YP_001968973.1	lipoprotein
Actinobacillus pleuropneumoniae serovar 7 str. AP76	8.90E-068	BamE	YP_001968246.1	small protein A
Actinobacillus pleuropneumoniae serovar 7 str. AP76	5.30E-184	Omp85	YP_001968229.1	protective surface antigen D15 precursor
Actinobacillus pleuropneumoniae serovar 7 str. AP76	3.60E-006	Omp85	YP_001968680.1	hypothetical protein APP7_0886
Actinobacillus succinogenes 130Z	2.50E-015	BamC	YP_001343335.1	NlpB/DapX family lipoprotein
Actinobacillus succinogenes 130Z	3.00E-137	BamD	YP_001343353.1	TPR repeat-containing protein
Actinobacillus succinogenes 130Z	1.70E-053	BamE	YP_001344085.1	SmpA/OmlA domain-containing protein
Actinobacillus succinogenes 130Z	1.10E-186	Omp85	YP_001345246.1	surface antigen (D15)
Actinobacillus succinogenes 130Z	5.50E-007	Omp85	YP_001343676.1	surface antigen (D15)
Aeromonas hydrophila subsp. hydrophila ATCC 7966	7.40E-165	BamB	YP_856298.1	PQQ repeat-containing protein
Aeromonas hydrophila subsp. hydrophila ATCC 7966	4.90E-041	BamC	YP_855440.1	NlpB/DapX lipoprotein
Aeromonas hydrophila subsp. hydrophila ATCC 7966	1.90E-141	BamD	YP_858499.1	ComL family lipoprotein
Aeromonas hydrophila subsp. hydrophila ATCC 7966	2.40E-040	BamE	YP_855985.1	outer membrane lipoprotein OmlA
Aeromonas hydrophila subsp. hydrophila ATCC 7966	3.70E-005	BamE	YP_858403.1	hypothetical protein AHA_3969
Aeromonas hydrophila subsp. hydrophila ATCC 7966	8.80E-005	BamE	YP_855839.1	hypothetical protein AHA_1297
Aeromonas hydrophila subsp. hydrophila ATCC 7966	1.90E-237	Omp85	YP_855722.1	OMP85 family outer membrane protein
Aeromonas hydrophila subsp. hydrophila ATCC 7966	1.70E-007	Omp85	YP_856091.1	OMP85 family outer membrane protein
Aeromonas salmonicida subsp. salmonicida A449	4.40E-164	BamB	YP_001142369.1	WD40 domain-containing protein
Aeromonas salmonicida subsp. salmonicida A449	1.80E-044	BamC	YP_001143118.1	lipoprotein-34 NlpB
Aeromonas salmonicida subsp. salmonicida A449	1.80E-141	BamD	YP_001140182.1	ComL family lipoprotein
Aeromonas salmonicida subsp. salmonicida A449	2.40E-041	BamE	YP_001142673.1	small protein A
Aeromonas salmonicida subsp. salmonicida A449	7.30E-006	BamE	YP_001143707.1	hypothetical protein ASA_4011
Aeromonas salmonicida subsp. salmonicida A449	2.10E-238	Omp85	YP_001142894.1	surface antigen
Aeromonas salmonicida subsp. salmonicida A449	2.50E-007	Omp85	YP_001142567.1	outer membrane protein
Aeromonas veronii B565	9.50E-164	BamB	YP_004393017.1	WD40 domain-containing protein
Aeromonas veronii B565	1.20E-019	BamC	YP_004393993.1	lipoprotein-34 NlpB
Aeromonas veronii B565	1.20E-141	BamD	YP_004394421.1	ComL family lipoprotein
Aeromonas veronii B565	7.80E-048	BamE	YP_004393390.1	Small protein A
Aeromonas veronii B565	3.40E-005	BamE	YP_004390866.1	hypothetical protein B565_0214
Aeromonas veronii B565	1.00E-235	Omp85	YP_004393699.1	Surface antigen
Aeromonas veronii B565	4.50E-007	Omp85	YP_004393298.1	outer membrane protein, OMP85 family
Aggregatibacter aphrophilus NJ8700	1.00E-018	BamC	YP_003006672.1	NlpB protein
Aggregatibacter aphrophilus NJ8700	1.00E-152	BamD	YP_003008669.1	lipoprotein
Aggregatibacter aphrophilus NJ8700	4.40E-051	BamE	YP_003008210.1	SmpA protein
Aggregatibacter aphrophilus NJ8700	2.30E-005	BamE	YP_003007200.1	Plp4

Aggregatibacter aphrophilus NJ8700	4.70E-175	Omp85	YP_003007977.1	outer membrane protein assembly complex, YaeT protein
Aggregatibacter aphrophilus NJ8700	7.80E-007	Omp85	YP_003008027.1	outer membrane protein
Alcanivorax borkumensis SK2	3.20E-114	BamB	YP_693577.1	hypothetical protein ABO_1857
Alcanivorax borkumensis SK2	2.70E-094	BamD	YP_692195.1	competence lipoprotein ComL
Alcanivorax borkumensis SK2	9.60E-023	BamE	YP_692028.1	outer membrane lipoprotein OmlA
Alcanivorax borkumensis SK2	7.30E-208	Omp85	YP_692871.1	outer membrane protein surface antigen family protein
Alcanivorax borkumensis SK2	2.00E-007	Omp85	YP_692462.1	hypothetical protein ABO_0742
Aliivibrio salmonicida LFI1238	1.00E-157	BamB	YP_002262249.1	outer membrane protein assembly complex subunit YfgL
Aliivibrio salmonicida LFI1238	5.90E-168	BamC	YP_002263733.1	lipoprotein
Aliivibrio salmonicida LFI1238	7.60E-147	BamD	YP_002262181.1	outer membrane protein
Aliivibrio salmonicida LFI1238	3.30E-052	BamE	YP_002263826.1	lipoprotein, small protein A
Aliivibrio salmonicida LFI1238	5.80E-233	Omp85	YP_002263773.1	outer membrane protein assembly factor YaeT
Aliivibrio salmonicida LFI1238	2.20E-006	Omp85	YP_002261962.1	surface antigen protein
Alkalilimnicola ehrlichii MLHE-1	7.90E-101	BamB	YP_742093.1	Pyrrolo-quinoline quinone
Alkalilimnicola ehrlichii MLHE-1	1.40E-007	BamB	YP_743559.1	Pyrrolo-quinoline quinone
Alkalilimnicola ehrlichii MLHE-1	3.40E-007	BamC	YP_743305.1	NlpBDapX lipoprotein
Alkalilimnicola ehrlichii MLHE-1	1.00E-106	BamD	YP_743381.1	putative lipoprotein
Alkalilimnicola ehrlichii MLHE-1	9.90E-015	BamD	YP_743233.1	DNA uptake lipoprotein-like protein
Alkalilimnicola ehrlichii MLHE-1	4.40E-022	BamE	YP_742740.1	SmpA/OmlA domain-containing protein
Alkalilimnicola ehrlichii MLHE-1	4.60E-199	Omp85	YP_742689.1	surface antigen (D15)
Alkalilimnicola ehrlichii MLHE-1	4.00E-008	Omp85	YP_741377.1	surface antigen (D15)
Allochromatium vinosum DSM 180	2.40E-118	BamB	YP_003443245.1	outer membrane assembly lipoprotein YfgL
Allochromatium vinosum DSM 180	2.40E-005	BamC	YP_003444357.1	putative lipoprotein
Allochromatium vinosum DSM 180	2.10E-109	BamD	YP_003442773.1	outer membrane assembly lipoprotein YfiO
Allochromatium vinosum DSM 180	8.20E-005	BamD	YP_003442932.1	tol-pal system protein YbgF
Allochromatium vinosum DSM 180	4.30E-024	BamE	YP_003444194.1	SmpA/OmlA domain-containing protein
Allochromatium vinosum DSM 180	5.70E-207	Omp85	YP_003444006.1	outer membrane protein assembly complex, YaeT protein
Allochromatium vinosum DSM 180	2.40E-006	Omp85	YP_003443296.1	surface antigen
Alteromonas macleodii str. 'Deep ecotype'	1.10E-123	BamB	YP_004427699.1	outer membrane biogenesis protein BamB
Alteromonas macleodii str. 'Deep ecotype'	2.10E-122	BamB	YP_004426190.1	outer membrane biogenesis protein BamB
Alteromonas macleodii str. 'Deep ecotype'	1.20E-083	BamC	YP_004427299.1	lipoprotein-34 NlpB
Alteromonas macleodii str. 'Deep ecotype'	7.10E-131	BamD	YP_004427604.1	Competence lipoprotein ComL
Alteromonas macleodii str. 'Deep ecotype'	6.10E-040	BamE	YP_004426535.1	SmpA/OmlA protein
Alteromonas macleodii str. 'Deep ecotype'	9.30E-005	BamE	YP_004426729.1	hypothetical protein MADE_1007955
Alteromonas macleodii str. 'Deep ecotype'	4.50E-185	Omp85	YP_004426216.1	outer membrane protein, OMP85 family
Alteromonas macleodii str. 'Deep ecotype'	2.60E-007	Omp85	YP_004427398.1	surface antigen D15
Alteromonas macleodii str. 'Deep ecotype'	2.90E-007	Omp85	YP_004429152.1	surface antigen D15
Alteromonas sp. SN2	1.30E-108	BamB	YP_004466149.1	outer membrane biogenesis protein BamB

Alteromonas sp. SN2	1.80E-008	BamB	YP_004465597.1	methanol/ethanol family PQQ-dependent dehydrogenase
Alteromonas sp. SN2	1.50E-074	BamC	YP_004467096.1	lipoprotein-34 NlpB
Alteromonas sp. SN2	2.70E-130	BamD	YP_004466243.1	Competence lipoprotein ComL
Alteromonas sp. SN2	1.60E-039	BamE	YP_004467638.1	SmpA/OmlA protein
Alteromonas sp. SN2	1.50E-185	Omp85	YP_004468053.1	OMP85 family outer membrane protein
Alteromonas sp. SN2	1.20E-006	Omp85	YP_004469654.1	surface antigen D15
Azotobacter vinelandii DJ	1.20E-168	BamB	YP_002801137.1	YfgL-like phosphoquinolipoprotein kinase
Azotobacter vinelandii DJ	2.50E-167	BamD	YP_002798396.1	competence protein ComL
Azotobacter vinelandii DJ	9.60E-005	BamD	YP_002800784.1	tol-pal system YbgF-like protein
Azotobacter vinelandii DJ	4.40E-025	BamE	YP_002801398.1	outer membrane lipoprotein OmlA
Azotobacter vinelandii DJ	3.40E-194	Omp85	YP_002801006.1	surface antigen
Azotobacter vinelandii DJ	5.10E-006	Omp85	YP_002800032.1	outer membrane surface antigen (D15)
Baumannia cicadellinicola str. Hc (Homalodisca coagulata)	2.90E-114	BamB	YP_588489.2	outer membrane protein assembly complex subunit YfgL
Baumannia cicadellinicola str. Hc (Homalodisca coagulata)	1.00E-073	BamD	YP_588649.1	hypothetical protein BCI_0192
Baumannia cicadellinicola str. Hc (Homalodisca coagulata)	8.20E-059	BamE	YP_588995.1	small protein A
Baumannia cicadellinicola str. Hc (Homalodisca coagulata)	5.50E-118	Omp85	YP_588965.1	OMP85 family outer membrane protein
Baumannia cicadellinicola str. Hc (Homalodisca coagulata)	5.90E-005	Omp85	YP_589033.1	OMP85 family outer membrane protein
Buchnera aphidicola (Cinara tujafilina)	1.30E-008	Omp85	YP_004589997.1	outer membrane protein
Buchnera aphidicola str. 5A (Acyrthosiphon pisum)	2.30E-035	BamD	YP_002468144.1	hypothetical 27.8 kDa lipoprotein
Buchnera aphidicola str. 5A (Acyrthosiphon pisum)	4.30E-007	BamE	YP_002467944.1	small protein A
Buchnera aphidicola str. 5A (Acyrthosiphon pisum)	6.50E-089	Omp85	YP_002467993.1	YaeT
Buchnera aphidicola str. APS (Acyrthosiphon pisum)	2.30E-035	BamD	NP_240219.1	hypothetical protein BU402
Buchnera aphidicola str. APS (Acyrthosiphon pisum)	3.90E-007	BamE	NP_240017.1	small protein A
Buchnera aphidicola str. APS (Acyrthosiphon pisum)	2.20E-009	Omp85	NP_240066.1	hypothetical protein BU237
Buchnera aphidicola str. Sg (Schizaphis graminum)	6.60E-028	BamD	NP_660730.1	hypothetical protein BUsg389
Buchnera aphidicola str. Sg (Schizaphis graminum)	1.40E-007	BamE	NP_660534.1	small protein A
Buchnera aphidicola str. Sg (Schizaphis graminum)	7.40E-098	Omp85	NP_660579.1	hypothetical protein BUsg231
Buchnera aphidicola str. Tuc7 (Acyrthosiphon pisum)	2.30E-035	BamD	YP_002468697.1	hypothetical 27.8 kDa lipoprotein
Buchnera aphidicola str. Tuc7 (Acyrthosiphon pisum)	3.90E-007	BamE	YP_002468500.1	small membrane protein A
Buchnera aphidicola str. Tuc7 (Acyrthosiphon pisum)	1.00E-089	Omp85	YP_002468548.1	YaeT
Candidatus Blochmannia floridanus	1.00E-146	BamD	NP_878483.1	putative lipoprotein
Candidatus Blochmannia floridanus	8.10E-011	BamE	NP_878825.1	small membrane protein A
Candidatus Blochmannia floridanus	1.50E-125	Omp85	NP_878576.1	outer membrane protein precursor
Candidatus Blochmannia pennsylvanicus str. BPEN	4.50E-090	BamD	YP_277697.1	putative lipoprotein
Candidatus Blochmannia pennsylvanicus str. BPEN	3.30E-014	BamE	YP_278053.1	small membrane protein A
Candidatus Blochmannia pennsylvanicus str. BPEN	6.20E-136	Omp85	YP_277791.1	outer membrane protein precursor
Candidatus Blochmannia pennsylvanicus str. BPEN	2.50E-005	Omp85	YP_277607.1	putative outer membrane protein
Candidatus Blochmannia vafer str. BVAF	5.70E-092	BamD	YP_004124260.1	lipoprotein

Candidatus Blochmannia vafer str. BVAF	1.00E-010	BamE	YP_004124604.1	Small protein A
Candidatus Blochmannia vafer str. BVAF	1.80E-107	Omp85	YP_004124354.1	outer membrane protein assembly factor YaeT
Candidatus Hamiltonella defensa 5AT (Acyrthosiphon pisum)	1.30E-149	BamB	YP_002923632.1	outer membrane protein assembly complex subunit YfgL
Candidatus Hamiltonella defensa 5AT (Acyrthosiphon pisum)	4.20E-114	BamC	YP_002924581.1	lipoprotein
Candidatus Hamiltonella defensa 5AT (Acyrthosiphon pisum)	3.10E-136	BamD	YP_002923450.1	outer membrane protein assembly complex subunit YfiO
Candidatus Hamiltonella defensa 5AT (Acyrthosiphon pisum)	8.20E-043	BamE	YP_002923698.1	hypothetical protein HDEF_0829
Candidatus Hamiltonella defensa 5AT (Acyrthosiphon pisum)	1.40E-243	Omp85	YP_002923482.1	outer membrane protein assembly factor YaeT
Candidatus Hamiltonella defensa 5AT (Acyrthosiphon pisum)	9.70E-006	Omp85	YP_002923648.1	outer membrane protein
Candidatus Moranella endobia PCIT	1.20E-133	BamB	YP_004706704.1	outer membrane protein assembly complex subunit YfgL
Candidatus Moranella endobia PCIT	3.80E-060	BamD	YP_004706688.1	putative outer membrane protein assembly complex subunit YfiO
Candidatus Moranella endobia PCIT	1.40E-217	Omp85	YP_004706833.1	outer membrane protein assembly factor YaeT
Candidatus Riesia pediculicola USDA	1.40E-047	Omp85	YP_003603211.1	outer membrane protein assembly factor YaeT, putative
Candidatus Ruthia magnifica str. Cm (Calyptogena magnifica)	1.70E-046	BamD	YP_903737.1	putative transmembrane protein
Candidatus Ruthia magnifica str. Cm (Calyptogena magnifica)	6.00E-015	BamE	YP_903933.1	SmpA/OmlA domain-containing protein
Candidatus Ruthia magnifica str. Cm (Calyptogena magnifica)	1.60E-120	Omp85	YP_903776.1	surface antigen (D15)
Candidatus Vesicomyosocius okutanii HA	7.10E-059	BamD	YP_001219313.1	competence lipoprotein ComL
Candidatus Vesicomyosocius okutanii HA	5.60E-016	BamE	YP_001219515.1	hypothetical protein COSY_0679
Candidatus Vesicomyosocius okutanii HA	9.90E-121	Omp85	YP_001219350.1	surface antigen family protein
Cellvibrio japonicus Ueda107	1.20E-121	BamB	YP_001981971.1	PQQ enzyme repeat domain-containing protein
Cellvibrio japonicus Ueda107	4.40E-011	BamB	YP_001982739.1	serine/threonine protein kinase
Cellvibrio japonicus Ueda107	6.50E-108	BamD	YP_001983660.1	competence protein ComL
Cellvibrio japonicus Ueda107	1.40E-015	BamE	YP_001983807.1	outer membrane lipoprotein omlA
Cellvibrio japonicus Ueda107	6.10E-005	BamE	YP_001982829.1	putative lipoprotein
Cellvibrio japonicus Ueda107	2.70E-164	Omp85	YP_001981616.1	outer membrane protein
Cellvibrio japonicus Ueda107	2.30E-006	Omp85	YP_001983825.1	outer membrane protein, OMP85 family
Chromohalobacter salexigens DSM 3043	2.10E-109	BamB	YP_574902.1	Pyrrolo-quinoline quinone
Chromohalobacter salexigens DSM 3043	8.20E-005	BamB	YP_572233.1	quinoprotein glucose dehydrogenase
Chromohalobacter salexigens DSM 3043	1.40E-106	BamD	YP_572557.1	competence lipoprotein ComL
Chromohalobacter salexigens DSM 3043	1.90E-031	BamE	YP_575141.1	SmpA/OmlA
Chromohalobacter salexigens DSM 3043	3.00E-207	Omp85	YP_572632.1	surface antigen (D15)
Chromohalobacter salexigens DSM 3043	4.00E-007	Omp85	YP_573914.1	surface antigen (D15)
Citrobacter koseri ATCC BAA-895	2.30E-217	BamB	YP_001451873.1	outer membrane protein assembly complex subunit YfgL
Citrobacter koseri ATCC BAA-895	2.20E-005	BamB	YP_001454151.1	hypothetical protein CKO_02606
Citrobacter koseri ATCC BAA-895	6.20E-228	BamC	YP_001451909.1	lipoprotein
Citrobacter koseri ATCC BAA-895	1.60E-169	BamD	YP_001455426.1	outer membrane protein assembly complex subunit YfiO
Citrobacter koseri ATCC BAA-895	8.80E-005	BamD	YP_001454159.1	copper/silver efflux system outer membrane protein CusC
Citrobacter koseri ATCC BAA-895	3.80E-070	BamE	YP_001455448.1	hypothetical protein CKO_03939
Citrobacter koseri ATCC BAA-895	4.80E-005	BamE	YP_001453329.1	DNA-binding transcriptional activator OsmE

Citrobacter koseri ATCC BAA-895	6.70E-005	BamE	YP_001454614.1	periplasmic repressor CpxP
Citrobacter koseri ATCC BAA-895	7.30E-005	BamE	YP_001455037.1	hypothetical protein CKO_03520
Citrobacter koseri ATCC BAA-895	0.00E+000	Omp85	YP_001454710.1	outer membrane protein assembly factor YaeT
Citrobacter koseri ATCC BAA-895	4.70E-007	Omp85	YP_001455125.1	hypothetical protein CKO_03610
Citrobacter rodentium ICC168	7.10E-215	BamB	YP_003365993.1	dehydrogenase
Citrobacter rodentium ICC168	4.30E-006	BamB	YP_003363782.1	quinoprotein glucose dehydrogenase
Citrobacter rodentium ICC168	2.50E-222	BamC	YP_003365966.1	lipoprotein
Citrobacter rodentium ICC168	2.40E-173	BamD	YP_003366072.1	lipoprotein
Citrobacter rodentium ICC168	4.20E-069	BamE	YP_003366101.1	outer membrane assembly lipoprotein
Citrobacter rodentium ICC168	2.80E-005	BamE	YP_003364902.1	osmotically inducible lipoprotein E
Citrobacter rodentium ICC168	0.00E+000	Omp85	YP_003363826.1	outer membrane protein assembly factor
Citrobacter rodentium ICC168	3.00E-007	Omp85	YP_003366763.1	outer membrane protein assembly factor
Colwellia psychrerythraea 34H	1.10E-123	BamB	YP_270899.1	outer membrane protein assembly complex subunit YfgL
Colwellia psychrerythraea 34H	1.30E-008	BamB	YP_268617.1	quinoprotein alcohol dehydrogenase
Colwellia psychrerythraea 34H	6.40E-016	BamC	YP_269878.1	putative lipoprotein
Colwellia psychrerythraea 34H	1.20E-164	BamD	YP_270574.1	putative lipoprotein
Colwellia psychrerythraea 34H	6.90E-005	BamD	YP_267348.1	putative lipoprotein
Colwellia psychrerythraea 34H	3.70E-034	BamE	YP_270489.1	SmpA/OmlA family protein
Colwellia psychrerythraea 34H	7.00E-006	BamE	YP_268910.1	putative lipoprotein
Colwellia psychrerythraea 34H	6.70E-204	Omp85	YP_268303.1	OMP85 family outer membrane protein
Colwellia psychrerythraea 34H	3.60E-007	Omp85	YP_269248.1	OMP85 family outer membrane protein
Coxiella burnetii CbuG_Q212	3.00E-091	BamD	YP_002303712.1	lipoprotein, ComL family
Coxiella burnetii CbuG_Q212	6.60E-016	BamE	YP_002303253.1	OmlA
Coxiella burnetii CbuK_Q154	3.00E-094	BamD	YP_002305030.1	lipoprotein, ComL family
Coxiella burnetii CbuK_Q154	6.60E-016	BamE	YP_002305502.1	outer membrane lipoprotein
Coxiella burnetii CbuK_Q154	9.20E-184	Omp85	YP_002305755.1	outer membrane protein assembly factor
Coxiella burnetii Dugway 5J108-111	3.00E-094	BamD	YP_001424166.2	lipoprotein, ComL family
Coxiella burnetii Dugway 5J108-111	6.60E-016	BamE	YP_001424742.1	outer membrane lipoprotein
Coxiella burnetii Dugway 5J108-111	1.30E-185	Omp85	YP_001424030.1	outer membrane protein assembly factor
Coxiella burnetii RSA 331	9.20E-095	BamD	YP_001596931.1	competence lipoprotein ComL
Coxiella burnetii RSA 331	1.30E-185	Omp85	YP_001596536.1	outer membrane protein assembly complex, YaeT protein
Coxiella burnetii RSA 493	5.10E-095	BamD	NP_819783.2	competence lipoprotein ComL
Coxiella burnetii RSA 493	4.90E-015	BamE	NP_820294.1	outer membrane lipoprotein
Coxiella burnetii RSA 493	7.00E-185	Omp85	NP_819641.1	outer membrane protein assembly complex, YaeT protein
Cronobacter sakazakii ATCC BAA-894	1.00E-201	BamB	YP_001436861.1	outer membrane protein assembly complex subunit YfgL
Cronobacter sakazakii ATCC BAA-894	8.30E-005	BamB	YP_001439027.1	hypothetical protein ESA_02962
Cronobacter sakazakii ATCC BAA-894	2.10E-213	BamC	YP_001436885.1	lipoprotein
Cronobacter sakazakii ATCC BAA-894	7.00E-164	BamD	YP_001436778.1	outer membrane protein assembly complex subunit YfiO

Cronobacter sakazakii ATCC BAA-894	4.30E-005	BamD	YP_001437668.1	tetratricopeptide repeat protein
Cronobacter sakazakii ATCC BAA-894	4.90E-068	BamE	YP_001436757.1	hypothetical protein ESA_00638
Cronobacter sakazakii ATCC BAA-894	5.90E-006	BamE	YP_001438292.1	hypothetical protein ESA_02207
Cronobacter sakazakii ATCC BAA-894	9.00E-006	BamE	YP_001438232.1	DNA-binding transcriptional activator OsmE
Cronobacter sakazakii ATCC BAA-894	6.90E-005	BamE	YP_001437352.1	flagellar basal body-associated protein FliL
Cronobacter sakazakii ATCC BAA-894	0.00E+000	Omp85	YP_001439224.1	outer membrane protein assembly factor YaeT
Cronobacter sakazakii ATCC BAA-894	3.80E-007	Omp85	YP_001436364.1	hypothetical protein ESA_00227
Cronobacter turicensis z3032	5.90E-204	BamB	YP_003211469.1	outer membrane biogenesis protein BamB
Cronobacter turicensis z3032	5.60E-203	BamC	YP_003211435.1	lipoprotein
Cronobacter turicensis z3032	8.20E-150	BamD	YP_003211547.1	outer membrane biogenesis protein BamD
Cronobacter turicensis z3032	4.80E-005	BamD	YP_003210704.1	tetratricopeptide repeat protein
Cronobacter turicensis z3032	4.80E-069	BamE	YP_003211566.1	outer membrane biogenesis protein BamE
Cronobacter turicensis z3032	8.40E-006	BamE	YP_003210189.1	DNA-binding transcriptional activator OsmE
Cronobacter turicensis z3032	6.90E-005	BamE	YP_003211024.1	flagellar basal body-associated protein FliL
Cronobacter turicensis z3032	0.00E+000	Omp85	YP_003209168.1	outer membrane protein assembly factor YaeT
Cronobacter turicensis z3032	1.90E-007	Omp85	YP_003212011.1	hypothetical protein CTU_36480
Dichelobacter nodosus VCS1703A	6.80E-042	BamB	YP_001209430.1	quinonprotein alcohol dehydrogenase family protein
Dichelobacter nodosus VCS1703A	7.60E-007	BamC	YP_001209999.1	lipoprotein
Dichelobacter nodosus VCS1703A	3.90E-083	BamD	YP_001210176.1	competence protein ComL
Dichelobacter nodosus VCS1703A	1.40E-008	BamE	YP_001209951.1	SmpA/OmlA family lipoprotein
Dichelobacter nodosus VCS1703A	1.50E-148	Omp85	YP_001209590.1	outer membrane protein
Dichelobacter nodosus VCS1703A	7.90E-007	Omp85	YP_001209758.1	outer membrane protein
Dickeya dadantii 3937	1.20E-189	BamB	YP_003884073.1	protein assembly complex, lipoprotein component
Dickeya dadantii 3937	2.30E-202	BamC	YP_003882036.1	Lipoprotein-34
Dickeya dadantii 3937	3.70E-152	BamD	YP_003884204.1	lipoprotein
Dickeya dadantii 3937	2.30E-005	BamD	YP_003883424.1	hypothetical protein Dda3937_04037
Dickeya dadantii 3937	3.50E-062	BamE	YP_003881667.1	outer membrane lipoprotein OmlA
Dickeya dadantii 3937	2.80E-005	BamE	YP_003884060.1	transmembrane protein
Dickeya dadantii 3937	0.00E+000	Omp85	YP_003881844.1	hypothetical protein Dda3937_01884
Dickeya dadantii 3937	9.70E-007	Omp85	YP_003884415.1	outer membrane protein and surface antigen
Dickeya dadantii Ech586	6.20E-187	BamB	YP_003334594.1	outer membrane assembly lipoprotein YfgL
Dickeya dadantii Ech586	5.30E-197	BamC	YP_003332706.1	NlpBDapX family lipoprotein
Dickeya dadantii Ech586	5.20E-149	BamD	YP_003334706.1	outer membrane assembly lipoprotein YfiO
Dickeya dadantii Ech586	5.40E-006	BamD	YP_003333257.1	tetratricopeptide domain-containing protein
Dickeya dadantii Ech586	9.20E-063	BamE	YP_003332365.1	SmpA/OmlA domain-containing protein
Dickeya dadantii Ech586	0.00E+000	Omp85	YP_003332528.1	outer membrane protein assembly complex, YaeT protein
Dickeya dadantii Ech586	1.20E-006	Omp85	YP_003334898.1	surface antigen (D15)
Dickeya dadantii Ech703	2.50E-191	BamB	YP_002988333.1	outer membrane protein assembly complex subunit YfgL

Escherichia coli B str. REL606	1.70E-174	BamD	YP_003045674.1	outer membrane protein assembly complex subunit YfiO
Escherichia coli B str. REL606	1.30E-007	BamE	YP_003045694.1	small membrane lipoprotein
Escherichia coli B str. REL606	2.30E-005	BamE	YP_003044906.1	DNA-binding transcriptional activator OsmE
Escherichia coli B str. REL606	0.00E+000	Omp85	YP_003043404.1	outer membrane protein assembly factor YaeT
Escherichia coli B str. REL606	1.00E-006	Omp85	YP_003047261.1	putative outer membrane protein and surface antigen
Escherichia coli BW2952	8.70E-225	BamB	YP_002927473.1	protein assembly complex, lipoprotein component
Escherichia coli BW2952	7.50E-006	BamB	YP_002925319.1	glucose dehydrogenase
Escherichia coli BW2952	8.10E-238	BamC	YP_002927438.1	lipoprotein
Escherichia coli BW2952	1.70E-174	BamD	YP_002927552.1	putative lipoprotein
Escherichia coli BW2952	5.80E-068	BamE	YP_002927572.1	small membrane lipoprotein
Escherichia coli BW2952	2.30E-005	BamE	YP_002926749.1	DNA-binding transcriptional activator
Escherichia coli BW2952	0.00E+000	Omp85	YP_002925371.1	hypothetical protein BWG_0169
Escherichia coli BW2952	9.30E-007	Omp85	YP_002929122.1	putative outer membrane protein and surface antigen
Escherichia coli CFT073	1.30E-225	BamB	NP_754916.1	outer membrane protein assembly complex subunit YfgL
Escherichia coli CFT073	8.30E-006	BamB	NP_752103.1	glucose dehydrogenase
Escherichia coli CFT073	2.70E-237	BamC	NP_754887.1	lipoprotein
Escherichia coli CFT073	1.70E-174	BamD	NP_754998.1	outer membrane protein assembly complex subunit YfiO
Escherichia coli CFT073	5.80E-068	BamE	NP_755021.1	hypothetical protein c3139
Escherichia coli CFT073	2.30E-005	BamE	NP_754032.1	DNA-binding transcriptional activator OsmE
Escherichia coli CFT073	0.00E+000	Omp85	NP_752162.1	outer membrane protein assembly factor YaeT
Escherichia coli CFT073	1.10E-006	Omp85	NP_757165.1	hypothetical protein c5318
Escherichia coli E24377A	6.60E-225	BamB	YP_001463834.1	outer membrane protein assembly complex subunit YfgL
Escherichia coli E24377A	1.10E-005	BamB	YP_001461292.1	glucose dehydrogenase
Escherichia coli E24377A	8.10E-238	BamC	YP_001463802.1	lipoprotein
Escherichia coli E24377A	1.70E-174	BamD	YP_001463916.1	outer membrane protein assembly complex subunit YfiO
Escherichia coli E24377A	5.80E-068	BamE	YP_001463936.1	hypothetical protein EcE24377A_2901
Escherichia coli E24377A	2.30E-005	BamE	YP_001463035.1	DNA-binding transcriptional activator OsmE
Escherichia coli E24377A	0.00E+000	Omp85	YP_001461346.1	outer membrane protein assembly factor YaeT
Escherichia coli E24377A	1.10E-006	Omp85	YP_001465728.1	OMP85 family outer membrane protein
Escherichia coli ED1a	1.30E-225	BamB	YP_002398844.1	outer membrane protein assembly complex subunit YfgL
Escherichia coli ED1a	8.30E-006	BamB	YP_002396214.1	glucose dehydrogenase
Escherichia coli ED1a	9.00E-236	BamC	YP_002398816.1	lipoprotein
Escherichia coli ED1a	1.70E-174	BamD	YP_002398926.1	outer membrane protein assembly complex subunit YfiO
Escherichia coli ED1a	5.80E-068	BamE	YP_002398946.1	hypothetical protein ECED1_3055
Escherichia coli ED1a	2.30E-005	BamE	YP_002397892.1	DNA-binding transcriptional activator OsmE
Escherichia coli ED1a	0.00E+000	Omp85	YP_002396264.1	outer membrane protein assembly factor YaeT
Escherichia coli ED1a	9.70E-007	Omp85	YP_002400823.1	hypothetical protein ECED1_5078
Escherichia coli HS	6.60E-225	BamB	YP_001459305.1	outer membrane protein assembly complex subunit YfgL

Escherichia coli HS	7.30E-006	BamB	YP_001456910.1	glucose dehydrogenase
Escherichia coli HS	8.10E-238	BamC	YP_001459257.1	lipoprotein
Escherichia coli HS	1.70E-174	BamD	YP_001459389.1	outer membrane protein assembly complex subunit YfiO
Escherichia coli HS	5.80E-068	BamE	YP_001459410.1	hypothetical protein EcHS_A2775
Escherichia coli HS	2.30E-005	BamE	YP_001458517.1	DNA-binding transcriptional activator OsmE
Escherichia coli HS	0.00E+000	Omp85	YP_001456960.1	outer membrane protein assembly factor YaeT
Escherichia coli HS	9.30E-007	Omp85	YP_001461013.1	OMP85 family outer membrane protein
Escherichia coli IAI1	1.10E-223	BamB	YP_002387950.1	outer membrane protein assembly complex subunit YfgL
Escherichia coli IAI1	7.30E-006	BamB	YP_002385618.1	glucose dehydrogenase
Escherichia coli IAI1	8.10E-238	BamC	YP_002387916.1	lipoprotein
Escherichia coli IAI1	1.70E-174	BamD	YP_002388088.1	outer membrane protein assembly complex subunit YfiO
Escherichia coli IAI1	5.80E-068	BamE	YP_002388109.1	hypothetical protein ECIAI1_2738
Escherichia coli IAI1	2.30E-005	BamE	YP_002387218.1	DNA-binding transcriptional activator OsmE
Escherichia coli IAI1	0.00E+000	Omp85	YP_002385671.1	outer membrane protein assembly factor YaeT
Escherichia coli IAI1	9.30E-007	Omp85	YP_002389686.1	hypothetical protein ECIAI1_4453
Escherichia coli IAI39	2.30E-225	BamB	YP_002408651.1	outer membrane protein assembly complex subunit YfgL
Escherichia coli IAI39	1.10E-005	BamB	YP_002406173.1	glucose dehydrogenase
Escherichia coli IAI39	2.70E-237	BamC	YP_002408564.1	lipoprotein
Escherichia coli IAI39	1.70E-174	BamD	YP_002408736.1	outer membrane protein assembly complex subunit YfiO
Escherichia coli IAI39	5.80E-068	BamE	YP_002408757.1	hypothetical protein ECIAI39_2820
Escherichia coli IAI39	2.30E-005	BamE	YP_002407323.1	DNA-binding transcriptional activator OsmE
Escherichia coli IAI39	0.00E+000	Omp85	YP_002406226.1	outer membrane protein assembly factor YaeT
Escherichia coli IAI39	1.10E-006	Omp85	YP_002410552.1	hypothetical protein ECIAI39_4691
Escherichia coli O103:H2 str. 12009	5.10E-224	BamB	YP_003222920.1	protein assembly complex, lipoprotein component
Escherichia coli O103:H2 str. 12009	7.30E-006	BamB	YP_003220136.1	glucose dehydrogenase
Escherichia coli O103:H2 str. 12009	8.10E-238	BamC	YP_003222889.1	lipoprotein NlpB
Escherichia coli O103:H2 str. 12009	1.70E-174	BamD	YP_003223055.1	putative lipoprotein
Escherichia coli O103:H2 str. 12009	5.80E-068	BamE	YP_003223076.1	small membrane lipoprotein SmpA
Escherichia coli O103:H2 str. 12009	2.30E-005	BamE	YP_003221867.1	DNA-binding transcriptional activator OsmE
Escherichia coli O103:H2 str. 12009	0.00E+000	Omp85	YP_003220187.1	Omp85 family protein
Escherichia coli O103:H2 str. 12009	9.30E-007	Omp85	YP_003224833.1	putative outer membrane protein and surface antigen
Escherichia coli O111:H- str. 11128	1.10E-223	BamB	YP_003235603.1	protein assembly complex, lipoprotein component
Escherichia coli O111:H- str. 11128	1.10E-005	BamB	YP_003232677.1	glucose dehydrogenase
Escherichia coli O111:H- str. 11128	8.10E-238	BamC	YP_003235569.1	lipoprotein NlpB
Escherichia coli O111:H- str. 11128	1.70E-174	BamD	YP_003235679.1	putative lipoprotein
Escherichia coli O111:H- str. 11128	5.80E-068	BamE	YP_003235699.1	small membrane lipoprotein SmpA
Escherichia coli O111:H- str. 11128	2.30E-005	BamE	YP_003234666.1	DNA-binding transcriptional activator OsmE
Escherichia coli O111:H- str. 11128	0.00E+000	Omp85	YP_003232728.1	Omp85 family protein

Escherichia coli O111:H- str. 11128	8.00E-007	Omp85	YP_003237380.1	putative outer membrane protein and surface antigen
Escherichia coli O127:H6 str. E2348/69	3.20E-225	BamB	YP_002330293.1	outer membrane protein assembly complex subunit YfgL
Escherichia coli O127:H6 str. E2348/69	9.90E-006	BamB	YP_002327719.1	glucose dehydrogenase
Escherichia coli O127:H6 str. E2348/69	2.70E-237	BamC	YP_002330212.1	lipoprotein
Escherichia coli O127:H6 str. E2348/69	1.70E-174	BamD	YP_002330365.1	outer membrane protein assembly complex subunit YfiO
Escherichia coli O127:H6 str. E2348/69	5.80E-068	BamE	YP_002330397.1	hypothetical protein E2348C_2905
Escherichia coli O127:H6 str. E2348/69	2.30E-005	BamE	YP_002329387.1	DNA-binding transcriptional activator OsmE
Escherichia coli O127:H6 str. E2348/69	0.00E+000	Omp85	YP_002327769.1	outer membrane protein assembly factor YaeT
Escherichia coli O127:H6 str. E2348/69	9.50E-007	Omp85	YP_002332000.1	predicted outer membrane protein and surface antigen
Escherichia coli O157:H7 str. EC4115	1.30E-223	BamB	YP_002271982.1	outer membrane assembly lipoprotein YfgL
Escherichia coli O157:H7 str. EC4115	1.10E-005	BamB	YP_002268732.1	quinoprotein glucose dehydrogenase
Escherichia coli O157:H7 str. EC4115	6.90E-237	BamC	YP_002271946.1	lipoprotein, NlpB/DapX family
Escherichia coli O157:H7 str. EC4115	1.70E-174	BamD	YP_002272068.1	outer membrane assembly lipoprotein YfiO
Escherichia coli O157:H7 str. EC4115	5.80E-068	BamE	YP_002272086.1	lipoprotein, SmpA/OmlA family
Escherichia coli O157:H7 str. EC4115	2.30E-005	BamE	YP_002270808.1	osmotically-inducible lipoprotein E
Escherichia coli O157:H7 str. EC4115	0.00E+000	Omp85	YP_002268785.1	outer membrane protein assembly complex, YaeT protein
Escherichia coli O157:H7 str. EC4115	9.30E-007	Omp85	YP_002273764.1	outer membrane protein, OMP85 family
Escherichia coli O157:H7 str. EDL933	1.30E-223	BamB	NP_289065.1	outer membrane protein assembly complex subunit YfgL
Escherichia coli O157:H7 str. EDL933	1.10E-005	BamB	NP_285820.1	glucose dehydrogenase
Escherichia coli O157:H7 str. EDL933	1.70E-235	BamC	NP_289030.2	lipoprotein
Escherichia coli O157:H7 str. EDL933	1.70E-174	BamD	NP_289150.1	outer membrane protein assembly complex subunit YfiO
Escherichia coli O157:H7 str. EDL933	1.30E-007	BamE	NP_289169.1	small membrane protein A
Escherichia coli O157:H7 str. EDL933	2.30E-005	BamE	NP_288172.1	DNA-binding transcriptional activator OsmE
Escherichia coli O157:H7 str. EDL933	0.00E+000	Omp85	NP_285871.1	outer membrane protein assembly factor YaeT
Escherichia coli O157:H7 str. EDL933	8.70E-007	Omp85	NP_290852.1	hypothetical protein Z5831
Escherichia coli O157:H7 str. Sakai	1.30E-223	BamB	NP_311401.1	outer membrane protein assembly complex subunit YfgL
Escherichia coli O157:H7 str. Sakai	1.10E-005	BamB	NP_308155.1	glucose dehydrogenase
Escherichia coli O157:H7 str. Sakai	6.90E-237	BamC	NP_311366.2	lipoprotein
Escherichia coli O157:H7 str. Sakai	1.70E-174	BamD	NP_311485.1	outer membrane protein assembly complex subunit YfiO
Escherichia coli O157:H7 str. Sakai	1.30E-007	BamE	NP_311506.1	small membrane protein A
Escherichia coli O157:H7 str. Sakai	2.30E-005	BamE	NP_310472.1	DNA-binding transcriptional activator OsmE
Escherichia coli O157:H7 str. Sakai	0.00E+000	Omp85	NP_308206.1	outer membrane protein assembly factor YaeT
Escherichia coli O157:H7 str. Sakai	9.30E-007	Omp85	NP_313225.1	hypothetical protein ECs5198
Escherichia coli O157:H7 str. TW14359	1.30E-223	BamB	YP_003079295.1	outer membrane protein assembly complex subunit YfgL
Escherichia coli O157:H7 str. TW14359	1.10E-005	BamB	YP_003076096.1	glucose dehydrogenase
Escherichia coli O157:H7 str. TW14359	6.90E-237	BamC	YP_003079259.1	lipoprotein
Escherichia coli O157:H7 str. TW14359	1.70E-174	BamD	YP_003079380.1	outer membrane protein assembly complex subunit YfiO
Escherichia coli O157:H7 str. TW14359	5.80E-068	BamE	YP_003079399.1	hypothetical protein ECSP_3561

Escherichia coli O157:H7 str. TW14359	2.30E-005	BamE	YP_003078194.1	DNA-binding transcriptional activator OsmE
Escherichia coli O157:H7 str. TW14359	0.00E+000	Omp85	YP_003076147.1	outer membrane protein assembly factor YaeT
Escherichia coli O157:H7 str. TW14359	9.30E-007	Omp85	YP_003081078.1	putative outer membrane protein and surface antigen
Escherichia coli O26:H11 str. 11368	1.10E-223	BamB	YP_003230497.1	outer membrane protein assembly complex subunit YfgL
Escherichia coli O26:H11 str. 11368	1.10E-005	BamB	YP_003227227.1	glucose dehydrogenase
Escherichia coli O26:H11 str. 11368	8.10E-238	BamC	YP_003230463.1	lipoprotein
Escherichia coli O26:H11 str. 11368	1.70E-174	BamD	YP_003230576.1	outer membrane protein assembly complex subunit YfiO
Escherichia coli O26:H11 str. 11368	5.80E-068	BamE	YP_003230596.1	hypothetical protein ECO26_3658
Escherichia coli O26:H11 str. 11368	2.30E-005	BamE	YP_003229495.1	DNA-binding transcriptional activator OsmE
Escherichia coli O26:H11 str. 11368	0.00E+000	Omp85	YP_003227278.1	outer membrane protein assembly factor YaeT
Escherichia coli O26:H11 str. 11368	9.30E-007	Omp85	YP_003232265.1	outer membrane protein and surface antigen
Escherichia coli O55:H7 str. CB9615	6.60E-225	BamB	YP_003500551.1	outer membrane assembly lipoprotein YfgL
Escherichia coli O55:H7 str. CB9615	1.10E-005	BamB	YP_003497766.1	Quinoprotein glucose dehydrogenase
Escherichia coli O55:H7 str. CB9615	3.00E-236	BamC	YP_003500515.1	lipoprotein-34
Escherichia coli O55:H7 str. CB9615	1.70E-174	BamD	YP_003500686.1	lipoprotein
Escherichia coli O55:H7 str. CB9615	5.80E-068	BamE	YP_003500706.1	lipoprotein, SmpA/OmlA family
Escherichia coli O55:H7 str. CB9615	2.30E-005	BamE	YP_003499736.1	Osmotically-inducible lipoprotein E precursor
Escherichia coli O55:H7 str. CB9615	7.30E-005	BamE	YP_003500795.1	hypothetical protein G2583_3290
Escherichia coli O55:H7 str. CB9615	0.00E+000	Omp85	YP_003497817.1	outer membrane protein assembly factor yaeT precursor
Escherichia coli O55:H7 str. CB9615	9.30E-007	Omp85	YP_003502452.1	hypothetical protein G2583_5050
Escherichia coli S88	1.30E-225	BamB	YP_002392351.1	outer membrane protein assembly complex subunit YfgL
Escherichia coli S88	9.30E-006	BamB	YP_002389980.1	glucose dehydrogenase
Escherichia coli S88	2.70E-237	BamC	YP_002392326.1	lipoprotein
Escherichia coli S88	1.70E-174	BamD	YP_002392429.1	outer membrane protein assembly complex subunit YfiO
Escherichia coli S88	5.80E-068	BamE	YP_002392449.1	hypothetical protein ECS88_2803
Escherichia coli S88	2.30E-005	BamE	YP_002391520.1	DNA-binding transcriptional activator OsmE
Escherichia coli S88	0.00E+000	Omp85	YP_002390031.1	outer membrane protein assembly factor YaeT
Escherichia coli S88	1.10E-006	Omp85	YP_002394297.1	hypothetical protein ECS88_4813
Escherichia coli SE11	5.10E-224	BamB	YP_002294073.1	outer membrane protein assembly complex subunit YfgL
Escherichia coli SE11	7.30E-006	BamB	YP_002291399.1	glucose dehydrogenase
Escherichia coli SE11	8.10E-238	BamC	YP_002294036.1	lipoprotein
Escherichia coli SE11	1.70E-174	BamD	YP_002294156.1	outer membrane protein assembly complex subunit YfiO
Escherichia coli SE11	5.80E-068	BamE	YP_002294175.1	hypothetical protein ECSE_2900
Escherichia coli SE11	2.30E-005	BamE	YP_002293184.1	DNA-binding transcriptional activator OsmE
Escherichia coli SE11	0.00E+000	Omp85	YP_002291451.1	outer membrane protein assembly factor YaeT
Escherichia coli SE11	9.30E-007	Omp85	YP_002295801.1	hypothetical protein ECSE_4526
Escherichia coli SMS-3-5	4.70E-225	BamB	YP_001744700.1	outer membrane protein assembly complex subunit YfgL
Escherichia coli SMS-3-5	7.50E-006	BamB	YP_001742251.1	quinoprotein glucose dehydrogenase

Escherichia coli SMS-3-5	8.10E-238	BamC	YP_001744660.1	lipoprotein
Escherichia coli SMS-3-5	1.70E-174	BamD	YP_001744780.1	outer membrane protein assembly complex subunit YfiO
Escherichia coli SMS-3-5	2.10E-067	BamE	YP_001744800.1	hypothetical protein EcSMS35_2769
Escherichia coli SMS-3-5	2.30E-005	BamE	YP_001743510.1	DNA-binding transcriptional activator OsmE
Escherichia coli SMS-3-5	0.00E+000	Omp85	YP_001742305.1	outer membrane protein assembly factor YaeT
Escherichia coli SMS-3-5	1.10E-006	Omp85	YP_001746623.1	OMP85 family outer membrane protein
Escherichia coli str. K-12 substr. DH10B	8.70E-225	BamB	YP_001731442.1	outer membrane protein assembly complex subunit YfgL
Escherichia coli str. K-12 substr. DH10B	7.50E-006	BamB	YP_001729081.1	glucose dehydrogenase
Escherichia coli str. K-12 substr. DH10B	8.10E-238	BamC	YP_001731407.1	lipoprotein
Escherichia coli str. K-12 substr. DH10B	1.70E-174	BamD	YP_001731520.1	outer membrane protein assembly complex subunit YfiO
Escherichia coli str. K-12 substr. DH10B	5.80E-068	BamE	YP_001731539.1	hypothetical protein ECDH10B_2783
Escherichia coli str. K-12 substr. DH10B	2.30E-005	BamE	YP_001730716.1	DNA-binding transcriptional activator OsmE
Escherichia coli str. K-12 substr. DH10B	0.00E+000	Omp85	YP_001729133.1	outer membrane protein assembly factor YaeT
Escherichia coli str. K-12 substr. DH10B	9.30E-007	Omp85	YP_001732984.1	outer membrane protein and surface antigen
Escherichia coli str. K-12 substr. MG1655	8.70E-225	BamB	NP_417007.1	lipoprotein required for OM biogenesis, in BamABCDE complex
Escherichia coli str. K-12 substr. MG1655	7.50E-006	BamB	NP_414666.1	glucose dehydrogenase
Escherichia coli str. K-12 substr. MG1655	8.10E-238	BamC	NP_416972.4	lipoprotein required for OM biogenesis, in BamABCDE complex
Escherichia coli str. K-12 substr. MG1655	1.70E-174	BamD	NP_417086.1	lipoprotein required for OM biogenesis, in BamABCDE complex
Escherichia coli str. K-12 substr. MG1655	5.80E-068	BamE	NP_417107.2	lipoprotein component of BamABCDE OM biogenesis complex
Escherichia coli str. K-12 substr. MG1655	2.30E-005	BamE	NP_416253.1	DNA-binding transcriptional activator
Escherichia coli str. K-12 substr. MG1655	0.00E+000	Omp85	NP_414719.1	outer membrane protein assembly factor, forms pores, required for OM biogenesis in BamABCDE OM protein complex
Escherichia coli str. K-12 substr. MG1655	9.30E-007	Omp85	NP_418641.1	predicted outer membrane protein and surface antigen
Escherichia coli UMN026	6.60E-225	BamB	YP_002413534.1	outer membrane protein assembly complex subunit YfgL
Escherichia coli UMN026	7.50E-006	BamB	YP_002410900.1	glucose dehydrogenase
Escherichia coli UMN026	8.10E-238	BamC	YP_002413495.1	lipoprotein
Escherichia coli UMN026	1.70E-174	BamD	YP_002413616.1	outer membrane protein assembly complex subunit YfiO
Escherichia coli UMN026	5.80E-068	BamE	YP_002413636.1	hypothetical protein ECUMN_2941
Escherichia coli UMN026	2.30E-005	BamE	YP_002412756.1	DNA-binding transcriptional activator OsmE
Escherichia coli UMN026	0.00E+000	Omp85	YP_002410950.1	outer membrane protein assembly factor YaeT
Escherichia coli UMN026	1.10E-006	Omp85	YP_002415352.1	hypothetical protein ECUMN_4756
Escherichia coli UTI89	1.30E-225	BamB	YP_541824.1	outer membrane protein assembly complex subunit YfgL
Escherichia coli UTI89	9.30E-006	BamB	YP_539178.1	glucose dehydrogenase
Escherichia coli UTI89	2.70E-237	BamC	YP_541795.1	lipoprotein
Escherichia coli UTI89	1.70E-174	BamD	YP_541915.1	outer membrane protein assembly complex subunit YfiO
Escherichia coli UTI89	5.80E-068	BamE	YP_541938.1	hypothetical protein UTI89_C2951
Escherichia coli UTI89	2.30E-005	BamE	YP_540940.1	DNA-binding transcriptional activator OsmE
Escherichia coli UTI89	0.00E+000	Omp85	YP_539233.1	outer membrane protein assembly factor YaeT

Escherichia coli UTI89	1.00E-006	Omp85	YP_543763.1	hypothetical protein UTI89_C4828
Escherichia fergusonii ATCC 35469	2.70E-213	BamB	YP_002381842.1	outer membrane protein assembly complex subunit YfgL
Escherichia fergusonii ATCC 35469	7.50E-006	BamB	YP_002381371.1	glucose dehydrogenase
Escherichia fergusonii ATCC 35469	4.00E-231	BamC	YP_002381880.1	lipoprotein
Escherichia fergusonii ATCC 35469	2.00E-172	BamD	YP_002381673.1	outer membrane protein assembly complex subunit YfiO
Escherichia fergusonii ATCC 35469	5.80E-068	BamE	YP_002381656.1	hypothetical protein EFER_0456
Escherichia fergusonii ATCC 35469	4.60E-005	BamE	YP_002382483.1	DNA-binding transcriptional activator OsmE
Escherichia fergusonii ATCC 35469	8.80E-005	BamE	YP_002385231.1	N-acetylmuramoyl-l-alanine amidase II
Escherichia fergusonii ATCC 35469	0.00E+000	Omp85	YP_002381423.1	outer membrane protein assembly factor YaeT
Escherichia fergusonii ATCC 35469	1.10E-006	Omp85	YP_002385308.1	hypothetical protein EFER_4300
Ferrimonas balearica DSM 9799	1.80E-140	BamB	YP_003914020.1	outer membrane assembly lipoprotein YfgL
Ferrimonas balearica DSM 9799	2.60E-042	BamC	YP_003912570.1	NlpBDapX family lipoprotein
Ferrimonas balearica DSM 9799	2.70E-138	BamD	YP_003912082.1	outer membrane assembly lipoprotein YfiO
Ferrimonas balearica DSM 9799	1.20E-005	BamD	YP_003913913.1	hypothetical protein Fbal_2637
Ferrimonas balearica DSM 9799	3.00E-043	BamE	YP_003914182.1	SmpA/OmlA domain protein
Ferrimonas balearica DSM 9799	9.80E-231	Omp85	YP_003912243.1	outer membrane protein assembly complex, YaeT protein
Ferrimonas balearica DSM 9799	4.30E-009	Omp85	YP_003912718.1	surface antigen (D15)
Francisella philomiragia subsp. philomiragia ATCC 25017	1.20E-046	BamB	YP_001677793.1	hypothetical protein Fphi_1069
Francisella philomiragia subsp. philomiragia ATCC 25017	4.50E-049	BamD	YP_001678147.1	competence lipoprotein ComL
Francisella philomiragia subsp. philomiragia ATCC 25017	1.70E-010	BamE	YP_001677446.1	outer membrane lipoprotein
Francisella philomiragia subsp. philomiragia ATCC 25017	0.00E+000	Omp85	YP_001677922.1	outer hypothetical protein
Francisella sp. TX077308	1.90E-038	BamB	YP_004647193.1	outer membrane protein YfgL, lipoprotein component of the protein assembly complex (forms a complex with YaeT, Yfio, and NlpB)
Francisella sp. TX077308	7.40E-048	BamD	YP_004647589.1	lipoprotein assembly complex protein
Francisella sp. TX077308	2.30E-010	BamE	YP_004646622.1	outer membrane lipoprotein SmpA
Francisella sp. TX077308	0.00E+000	Omp85	YP_004647319.1	outer membrane protein assembly factor YaeT
Francisella tularensis subsp. holarctica FTNF002-00	3.70E-044	BamB	YP_001429258.1	lipoprotein
Francisella tularensis subsp. holarctica FTNF002-00	1.40E-051	BamD	YP_001428171.2	lipoprotein
Francisella tularensis subsp. holarctica FTNF002-00	5.00E-009	BamE	YP_001427511.1	Smp A/Oml family membrane lipoprotein
Francisella tularensis subsp. holarctica FTNF002-00	0.00E+000	Omp85	YP_001428000.2	outer membrane protein
Francisella tularensis subsp. holarctica LVS	3.70E-044	BamB	YP_514355.1	lipoprotein
Francisella tularensis subsp. holarctica LVS	1.40E-051	BamD	YP_513451.1	lipoprotein
Francisella tularensis subsp. holarctica LVS	5.00E-009	BamE	YP_512873.1	outer membrane lipoprotein
Francisella tularensis subsp. holarctica LVS	0.00E+000	Omp85	YP_513304.1	outer membrane protein
Francisella tularensis subsp. holarctica OSU18	3.70E-044	BamB	YP_764076.1	hypothetical protein FTH_1663
Francisella tularensis subsp. holarctica OSU18	8.60E-051	BamD	YP_763288.1	hypothetical protein FTH_0702
Francisella tularensis subsp. holarctica OSU18	5.00E-009	BamE	YP_762754.1	outer membrane lipoprotein
Francisella tularensis subsp. holarctica OSU18	0.00E+000	Omp85	YP_763147.1	outer membrane protein/protective antigen

Francisella tularensis subsp. mediasiatica FSC147	1.10E-044	BamB	YP_001891084.1	outer membrane assembly lipoprotein YfgL
Francisella tularensis subsp. mediasiatica FSC147	1.40E-051	BamD	YP_001891796.1	competence lipoprotein ComL
Francisella tularensis subsp. mediasiatica FSC147	1.60E-009	BamE	YP_001890931.1	outer membrane lipoprotein
Francisella tularensis subsp. mediasiatica FSC147	0.00E+000	Omp85	YP_001891156.1	conserved outer membrane protein of unknown function
Francisella tularensis subsp. novicida U112	5.90E-044	BamB	YP_899166.1	hypothetical protein FTN_1548
Francisella tularensis subsp. novicida U112	1.40E-051	BamD	YP_898898.1	competence lipoprotein ComL
Francisella tularensis subsp. novicida U112	1.30E-009	BamE	YP_897770.1	outer membrane lipoprotein
Francisella tularensis subsp. novicida U112	0.00E+000	Omp85	YP_899103.1	outer membrane protein of unknown function
Francisella tularensis subsp. tularensis FSC198	1.10E-044	BamB	YP_666362.1	lipoprotein
Francisella tularensis subsp. tularensis FSC198	1.40E-051	BamD	YP_667334.1	lipoprotein
Francisella tularensis subsp. tularensis FSC198	1.60E-009	BamE	YP_667719.1	outer membrane lipoprotein
Francisella tularensis subsp. tularensis FSC198	0.00E+000	Omp85	YP_667627.1	outer membrane protein
Francisella tularensis subsp. tularensis SCHU S4	1.10E-044	BamB	YP_169231.1	lipoprotein
Francisella tularensis subsp. tularensis SCHU S4	1.40E-051	BamD	YP_170202.1	lipoprotein
Francisella tularensis subsp. tularensis SCHU S4	1.60E-009	BamE	YP_170586.1	outer membrane lipoprotein
Francisella tularensis subsp. tularensis SCHU S4	0.00E+000	Omp85	YP_170495.1	outer membrane protein
Francisella tularensis subsp. tularensis WY96-3418	7.60E-045	BamB	YP_001121340.1	lipoprotein
Francisella tularensis subsp. tularensis WY96-3418	6.10E-050	BamD	YP_001121708.1	lipoprotein
Francisella tularensis subsp. tularensis WY96-3418	1.60E-009	BamE	YP_001122700.1	outer membrane lipoprotein
Francisella tularensis subsp. tularensis WY96-3418	0.00E+000	Omp85	YP_001121414.1	outer membrane protein
Gallibacterium anatis UMN179	3.70E-119	BamD	YP_004420935.1	outer membrane protein assembly complex subunit YfiO
Gallibacterium anatis UMN179	3.10E-036	BamE	YP_004420851.1	SmpA/OmlA family
Gallibacterium anatis UMN179	4.50E-006	BamE	YP_004419241.1	outer membrane protein A
Gallibacterium anatis UMN179	1.20E-178	Omp85	YP_004420787.1	outer membrane protein assembly factor YaeT
Gallibacterium anatis UMN179	3.50E-006	Omp85	YP_004419926.1	outer membrane protein assembly factor YaeT
Glaciecola sp. 4H-3-7+YE-5	1.50E-130	BamB	YP_004433438.1	outer membrane assembly lipoprotein YfgL
Glaciecola sp. 4H-3-7+YE-5	1.10E-007	BamB	YP_004434056.1	PQQ-dependent dehydrogenase
Glaciecola sp. 4H-3-7+YE-5	8.00E-005	BamB	YP_004434368.1	membrane-bound PQQ-dependent dehydrogenase
Glaciecola sp. 4H-3-7+YE-5	5.80E-056	BamC	YP_004434739.1	NlpBDapX lipoprotein
Glaciecola sp. 4H-3-7+YE-5	5.10E-137	BamD	YP_004435268.1	outer membrane assembly lipoprotein YfiO
Glaciecola sp. 4H-3-7+YE-5	9.10E-005	BamD	YP_004433617.1	tol-pal system protein YbgF
Glaciecola sp. 4H-3-7+YE-5	1.30E-037	BamE	YP_004434825.1	SmpA/OmlA domain-containing protein
Glaciecola sp. 4H-3-7+YE-5	7.10E-178	Omp85	YP_004435348.1	outer membrane protein assembly complex, YaeT protein
Glaciecola sp. 4H-3-7+YE-5	9.50E-006	Omp85	YP_004432409.1	surface antigen (D15)
Haemophilus ducreyi 35000HP	7.20E-111	BamD	NP_873040.1	putative lipoprotein
Haemophilus ducreyi 35000HP	1.20E-057	BamE	NP_873668.1	small protein A
Haemophilus ducreyi 35000HP	3.20E-172	Omp85	NP_873652.1	outer membrane protein D-15
Haemophilus ducreyi 35000HP	1.20E-005	Omp85	NP_874040.1	hypothetical protein HD1655

Haemophilus influenzae 86-028NP	5.90E-005	BamC	YP_247975.1	lipoprotein
Haemophilus influenzae 86-028NP	9.00E-165	BamD	YP_247894.1	hypothetical protein NTHI0266
Haemophilus influenzae 86-028NP	4.20E-048	BamE	YP_248545.1	small protein A
Haemophilus influenzae 86-028NP	2.00E-166	Omp85	YP_248615.1	protective surface antigen D15
Haemophilus influenzae 86-028NP	2.60E-006	Omp85	YP_248385.1	hypothetical protein NTHI0821
Haemophilus influenzae F3031	2.70E-005	BamC	YP_004134784.1	lipoprotein
Haemophilus influenzae F3031	1.20E-163	BamD	YP_004136339.1	lipoprotein
Haemophilus influenzae F3031	5.70E-049	BamE	YP_004135864.1	small protein a
Haemophilus influenzae F3031	8.70E-089	Omp85	YP_004135793.1	protective surface antigen, partial
Haemophilus influenzae F3031	3.40E-006	Omp85	YP_004136061.1	outer membrane protein and surface antigen
Haemophilus influenzae F3047	2.00E-005	BamC	YP_004138361.1	lipoprotein
Haemophilus influenzae F3047	1.20E-163	BamD	YP_004138441.1	lipoprotein
Haemophilus influenzae F3047	5.70E-049	BamE	YP_004137717.1	small protein A
Haemophilus influenzae F3047	2.30E-088	Omp85	YP_004137639.1	protective surface antigen
Haemophilus influenzae F3047	4.80E-006	Omp85	YP_004137945.1	outer membrane protein and surface antigen
Haemophilus influenzae PittEE	7.10E-005	BamC	YP_001290218.1	putative deoxyribonucleotide triphosphate pyrophosphatase
Haemophilus influenzae PittEE	4.10E-164	BamD	YP_001290315.1	hypothetical protein CGSHiEE_02415
Haemophilus influenzae PittEE	5.50E-052	BamE	YP_001291268.1	small protein A
Haemophilus influenzae PittEE	1.10E-172	Omp85	YP_001291192.1	protective surface antigen D15
Haemophilus influenzae PittEE	3.00E-006	Omp85	YP_001291412.1	hypothetical protein CGSHiEE_08650
Haemophilus influenzae PittGG	2.90E-005	BamC	YP_001292152.1	lipoprotein
Haemophilus influenzae PittGG	3.50E-166	BamD	YP_001292068.1	hypothetical protein CGSHiGG_03480
Haemophilus influenzae PittGG	5.50E-052	BamE	YP_001292776.1	small protein A
Haemophilus influenzae PittGG	1.00E-169	Omp85	YP_001292848.1	protective surface antigen D15
Haemophilus influenzae PittGG	2.40E-006	Omp85	YP_001292623.1	hypothetical protein CGSHiGG_06780
Haemophilus influenzae Rd KW20	1.70E-005	BamC	NP_438425.1	lipoprotein
Haemophilus influenzae Rd KW20	5.50E-161	BamD	NP_438345.1	hypothetical protein HI0177
Haemophilus influenzae Rd KW20	5.20E-050	BamE	NP_438998.1	small protein A
Haemophilus influenzae Rd KW20	3.00E-169	Omp85	NP_439077.1	protective surface antigen D15
Haemophilus influenzae Rd KW20	2.80E-006	Omp85	NP_438857.1	hypothetical protein HI0698
Haemophilus parasuis SH0165	8.60E-126	BamD	YP_002475502.1	DNA uptake lipoprotein, TPR repeat-containing protein
Haemophilus parasuis SH0165	7.50E-057	BamE	YP_002475299.1	SmpA/OmlA domain-containing protein
Haemophilus parasuis SH0165	3.50E-166	Omp85	YP_002475807.1	protective surface antigen D15
Haemophilus parasuis SH0165	1.30E-005	Omp85	YP_002474860.1	surface antigen (D15), outer membrane protein
Haemophilus somnus 129PT	2.20E-139	BamD	YP_718576.1	DNA uptake lipoprotein
Haemophilus somnus 129PT	7.50E-042	BamE	YP_718435.1	small protein A
Haemophilus somnus 129PT	3.00E-164	Omp85	YP_719191.1	surface antigen
Haemophilus somnus 129PT	2.90E-006	Omp85	YP_718547.1	outer membrane protein

Haemophilus somnus 2336	1.00E-138	BamD	YP_001783974.1	hypothetical protein HSM_0636
Haemophilus somnus 2336	5.20E-048	BamE	YP_001783450.1	SmpA/OmlA domain-containing protein
Haemophilus somnus 2336	1.30E-164	Omp85	YP_001784778.1	surface antigen (D15)
Haemophilus somnus 2336	3.30E-006	Omp85	YP_001783561.1	surface antigen (D15)
Hahella chejuensis KCTC 2396	5.90E-223	BamB	YP_435582.1	WD-40 repeat-containing protein
Hahella chejuensis KCTC 2396	5.40E-005	BamB	YP_436596.1	WD-40 repeat-containing protein
Hahella chejuensis KCTC 2396	8.80E-102	BamD	YP_436997.1	DNA uptake lipoprotein
Hahella chejuensis KCTC 2396	2.40E-018	BamE	YP_432517.1	small protein A (tmRNA-binding)
Hahella chejuensis KCTC 2396	3.70E-193	Omp85	YP_436344.1	outer membrane protein/protective antigen OMA87
Hahella chejuensis KCTC 2396	9.80E-006	Omp85	YP_433471.1	outer membrane protein
Halomonas elongata DSM 2581	2.90E-109	BamB	YP_003896452.1	pyrrolo-quinoline quinone
Halomonas elongata DSM 2581	8.00E-005	BamB	YP_003899075.1	quinoprotein glucose dehydrogenase
Halomonas elongata DSM 2581	7.50E-108	BamD	YP_003896397.1	lipoprotein
Halomonas elongata DSM 2581	9.40E-027	BamE	YP_003899232.1	outer membrane lipoprotein OmlA
Halomonas elongata DSM 2581	7.20E-006	BamE	YP_003896726.1	outer membrane lipoprotein, OmpA/SmpA/OmlA family
Halomonas elongata DSM 2581	1.10E-194	Omp85	YP_003898865.1	hypothetical protein HELO_3796
Halomonas elongata DSM 2581	5.40E-007	Omp85	YP_003897645.1	hypothetical protein HELO_2576
Halorhodospira halophila SL1	2.00E-074	BamB	YP_001003352.1	Pyrrolo-quinoline quinone
Halorhodospira halophila SL1	9.50E-006	BamB	YP_001002863.1	Pyrrolo-quinoline quinone
Halorhodospira halophila SL1	2.30E-077	BamD	YP_001003802.1	putative lipoprotein
Halorhodospira halophila SL1	1.50E-015	BamD	YP_001003934.1	DNA uptake lipoprotein-like protein
Halorhodospira halophila SL1	9.70E-020	BamE	YP_001003051.1	SmpA/OmlA domain-containing protein
Halorhodospira halophila SL1	8.60E-189	Omp85	YP_001003027.1	surface antigen (D15)
Halorhodospira halophila SL1	4.60E-008	Omp85	YP_001003535.1	surface antigen (D15)
Halothiobacillus neapolitanus c2	1.60E-099	BamB	YP_003264060.1	outer membrane assembly lipoprotein YfgL
Halothiobacillus neapolitanus c2	2.10E-007	BamC	YP_003262577.1	lipoprotein
Halothiobacillus neapolitanus c2	7.90E-091	BamD	YP_003263903.1	outer membrane assembly lipoprotein YfiO
Halothiobacillus neapolitanus c2	4.00E-012	BamE	YP_003262869.1	SmpA/OmlA domain-containing protein
Halothiobacillus neapolitanus c2	2.70E-196	Omp85	YP_003263325.1	outer membrane protein assembly complex, YaeT protein
Halothiobacillus neapolitanus c2	2.50E-006	Omp85	YP_003263622.1	surface antigen (D15)
Idiomarina loihiensis L2TR	9.70E-139	BamB	YP_156412.1	outer membrane protein assembly complex subunit YfgL
Idiomarina loihiensis L2TR	1.70E-005	BamB	YP_155179.1	glucose dehydrogenase
Idiomarina loihiensis L2TR	6.50E-018	BamC	YP_155848.1	hypothetical protein IL1459
Idiomarina loihiensis L2TR	4.20E-132	BamD	YP_155573.1	competence lipoprotein ComL
Idiomarina loihiensis L2TR	2.20E-037	BamE	YP_155379.1	Outer membrane lipoprotein OmlA (small protein A)
Idiomarina loihiensis L2TR	2.00E-188	Omp85	YP_155226.1	surface antigen
Idiomarina loihiensis L2TR	1.00E-006	Omp85	YP_156268.1	outer membrane protein
Kangiella koreensis DSM 16069	1.40E-113	BamB	YP_003147015.1	outer membrane assembly lipoprotein YfgL

Kangiella koreensis DSM 16069	1.30E-013	BamC	YP_003146806.1	NlpBDapX family lipoprotein
Kangiella koreensis DSM 16069	2.00E-105	BamD	YP_003146268.1	outer membrane assembly lipoprotein YfiO
Kangiella koreensis DSM 16069	2.20E-021	BamE	YP_003147623.1	SmpA/OmlA domain-containing protein
Kangiella koreensis DSM 16069	3.50E-156	Omp85	YP_003147082.1	outer membrane protein assembly complex, YaeT protein
Klebsiella pneumoniae 342	6.70E-214	BamB	YP_002237138.1	outer membrane protein assembly complex subunit YfgL
Klebsiella pneumoniae 342	4.60E-006	BamB	YP_002238229.1	quinate dehydrogenase (pyrroloquinoline-quinone)
Klebsiella pneumoniae 342	4.80E-005	BamB	YP_002240396.1	quinoprotein glucose dehydrogenase
Klebsiella pneumoniae 342	8.40E-005	BamB	YP_002237959.1	quinate/shikimate dehydrogenase (pyrroloquinoline-quinone)
Klebsiella pneumoniae 342	1.80E-213	BamC	YP_002237185.1	lipoprotein
Klebsiella pneumoniae 342	1.10E-169	BamD	YP_002237067.1	outer membrane protein assembly complex subunit YfiO
Klebsiella pneumoniae 342	1.40E-066	BamE	YP_002237045.1	hypothetical protein KPK_1181
Klebsiella pneumoniae 342	2.60E-006	BamE	YP_002239039.1	DNA-binding transcriptional activator OsmE
Klebsiella pneumoniae 342	6.20E-005	BamE	YP_002240542.1	lipoprotein
Klebsiella pneumoniae 342	0.00E+000	Omp85	YP_002240335.1	outer membrane protein assembly factor YaeT
Klebsiella pneumoniae 342	2.00E-249	Omp85	YP_002240991.1	outer membrane protein assembly factor YaeT
Klebsiella pneumoniae 342	2.80E-007	Omp85	YP_002240823.1	outer membrane protein, OMP85 family
Klebsiella pneumoniae subsp. pneumoniae MGH 78578	6.20E-214	BamB	YP_001336482.1	outer membrane protein assembly complex subunit YfgL
Klebsiella pneumoniae subsp. pneumoniae MGH 78578	4.90E-006	BamB	YP_001335612.1	glucose dehydrogenase
Klebsiella pneumoniae subsp. pneumoniae MGH 78578	4.90E-005	BamB	YP_001333822.1	glucose dehydrogenase
Klebsiella pneumoniae subsp. pneumoniae MGH 78578	5.00E-212	BamC	YP_001336451.1	lipoprotein
Klebsiella pneumoniae subsp. pneumoniae MGH 78578	1.10E-169	BamD	YP_001336554.1	outer membrane protein assembly complex subunit YfiO
Klebsiella pneumoniae subsp. pneumoniae MGH 78578	7.90E-067	BamE	YP_001336575.1	hypothetical protein KPN_02939
Klebsiella pneumoniae subsp. pneumoniae MGH 78578	5.30E-006	BamE	YP_001334892.1	DNA-binding transcriptional activator OsmE
Klebsiella pneumoniae subsp. pneumoniae MGH 78578	0.00E+000	Omp85	YP_001333880.1	outer membrane protein assembly factor YaeT
Klebsiella pneumoniae subsp. pneumoniae MGH 78578	1.40E-253	Omp85	YP_001338074.1	outer membrane protein assembly factor YaeT
Klebsiella pneumoniae subsp. pneumoniae MGH 78578	2.30E-007	Omp85	YP_001338234.1	putative outer membrane protein
Klebsiella pneumoniae subsp. pneumoniae NTUH-K2044	2.00E-214	BamB	YP_002920703.1	outer membrane protein assembly complex subunit YfgL
Klebsiella pneumoniae subsp. pneumoniae NTUH-K2044	8.40E-006	BamB	YP_002919726.1	glucose dehydrogenase
Klebsiella pneumoniae subsp. pneumoniae NTUH-K2044	4.90E-005	BamB	YP_002917845.1	glucose dehydrogenase
Klebsiella pneumoniae subsp. pneumoniae NTUH-K2044	8.00E-005	BamB	YP_002918293.1	putative periplasmic protein
Klebsiella pneumoniae subsp. pneumoniae NTUH-K2044	2.50E-211	BamC	YP_002920673.1	lipoprotein
Klebsiella pneumoniae subsp. pneumoniae NTUH-K2044	1.10E-169	BamD	YP_002920777.1	outer membrane protein assembly complex subunit YfiO
Klebsiella pneumoniae subsp. pneumoniae NTUH-K2044	7.90E-067	BamE	YP_002920798.1	hypothetical protein KP1_4195
Klebsiella pneumoniae subsp. pneumoniae NTUH-K2044	5.30E-006	BamE	YP_002919018.1	DNA-binding transcriptional activator OsmE
Klebsiella pneumoniae subsp. pneumoniae NTUH-K2044	0.00E+000	Omp85	YP_002917907.1	outer membrane protein assembly factor YaeT
Klebsiella pneumoniae subsp. pneumoniae NTUH-K2044	2.30E-253	Omp85	YP_002917274.1	outer membrane protein assembly factor YaeT
Klebsiella pneumoniae subsp. pneumoniae NTUH-K2044	2.50E-007	Omp85	YP_002917433.1	putative outer membrane protein
Klebsiella variicola At-22	6.70E-214	BamB	YP_003438156.1	outer membrane assembly lipoprotein YfgL

Klebsiella variicola At-22	4.10E-006	BamB	YP_003439276.1	membrane-bound PQQ-dependent dehydrogenase, glucose/quinate/shikimate family
Klebsiella variicola At-22	6.40E-005	BamB	YP_003441159.1	membrane-bound PQQ-dependent dehydrogenase, glucose/quinate/shikimate family
Klebsiella variicola At-22	7.00E-005	BamB	YP_003438927.1	membrane-bound PQQ-dependent dehydrogenase, glucose/quinate/shikimate family
Klebsiella variicola At-22	1.80E-213	BamC	YP_003438183.1	NlpBDapX family lipoprotein
Klebsiella variicola At-22	1.00E-168	BamD	YP_003438088.1	outer membrane assembly lipoprotein YfiO
Klebsiella variicola At-22	1.40E-066	BamE	YP_003438067.1	SmpA/OmlA domain-containing protein
Klebsiella variicola At-22	2.60E-006	BamE	YP_003440002.1	SmpA/OmlA domain-containing protein
Klebsiella variicola At-22	0.00E+000	Omp85	YP_003441100.1	outer membrane protein assembly complex, YaeT protein
Klebsiella variicola At-22	1.00E-251	Omp85	YP_003441700.1	outer membrane protein assembly complex, YaeT protein
Klebsiella variicola At-22	2.60E-007	Omp85	YP_003441537.1	surface antigen (D15)
Legionella longbeachae NSW150	4.40E-090	BamB	YP_003455006.1	PQQ (pyrrolo-quinoline quinone) enzyme repeat protein
Legionella longbeachae NSW150	9.70E-077	BamD	YP_003454771.1	competence lipoprotein comL precursor
Legionella longbeachae NSW150	2.00E-015	BamE	YP_003454012.1	small protein A, tmRNA-binding
Legionella longbeachae NSW150	3.10E-301	Omp85	YP_003456150.1	outer membrane protein assembly factor
Legionella pneumophila 2300/99 Alcoy	8.90E-073	BamB	YP_003618824.1	PQQ (pyrrolo quinoline) WD40-like repeat, enzyme repeat domain protein
Legionella pneumophila 2300/99 Alcoy	9.10E-080	BamD	YP_003618517.1	DNA uptake lipoprotein
Legionella pneumophila 2300/99 Alcoy	6.90E-016	BamE	YP_003617620.1	small protein A
Legionella pneumophila 2300/99 Alcoy	0.00E+000	Omp85	YP_003617755.1	Outer membrane protein/protective antigen OMA87
Legionella pneumophila str. Corby	8.90E-073	BamB	YP_001250283.1	PQQ (pyrrolo quinoline) WD40-like protein
Legionella pneumophila str. Corby	9.10E-080	BamD	YP_001249979.1	competence lipoprotein ComL
Legionella pneumophila str. Corby	6.90E-016	BamE	YP_001252220.1	tmRNA-binding small protein A
Legionella pneumophila str. Corby	0.00E+000	Omp85	YP_001252086.1	outer membrane protein
Legionella pneumophila str. Lens	1.70E-079	BamB	YP_126830.1	hypothetical protein lpl1484
Legionella pneumophila str. Lens	3.10E-080	BamD	YP_126545.1	hypothetical protein lpl1194
Legionella pneumophila str. Lens	5.10E-014	BamE	YP_125779.1	hypothetical protein lpl0413
Legionella pneumophila str. Lens	0.00E+000	Omp85	YP_125911.1	hypothetical protein lpl0545
Legionella pneumophila str. Paris	5.20E-081	BamB	YP_123823.1	hypothetical protein lpp1499
Legionella pneumophila str. Paris	3.10E-080	BamD	YP_123512.1	hypothetical protein lpp1188
Legionella pneumophila str. Paris	5.10E-014	BamE	YP_122777.1	hypothetical protein lpp0437
Legionella pneumophila str. Paris	0.00E+000	Omp85	YP_122907.1	hypothetical protein lpp0569
Legionella pneumophila subsp. pneumophila str. Philadelphia 1	2.00E-078	BamB	YP_095571.1	PQQ WD-40-like repeat-containing protein
Legionella pneumophila subsp. pneumophila str. Philadelphia 1	9.10E-080	BamD	YP_095218.1	competence lipoprotein ComL
Legionella pneumophila subsp. pneumophila str. Philadelphia 1	2.30E-015	BamE	YP_094416.1	small protein A, tmRNA-binding
Legionella pneumophila subsp. pneumophila str. Philadelphia 1	0.00E+000	Omp85	YP_094550.1	outer membrane protein
Mannheimia succiniciproducens MBEL55E	1.40E-015	BamC	YP_087459.1	NlpB protein
Mannheimia succiniciproducens MBEL55E	1.30E-144	BamD	YP_089012.1	NrfG protein

Mannheimia succiniciproducens MBEL55E	6.50E-005	BamD	YP_088323.1	TolC protein
Mannheimia succiniciproducens MBEL55E	9.30E-005	BamD	YP_088665.1	tetratricopeptide repeat protein
Mannheimia succiniciproducens MBEL55E	4.30E-055	BamE	YP_089104.1	SmpA protein
Mannheimia succiniciproducens MBEL55E	4.70E-178	Omp85	YP_089116.1	hypothetical protein MS1924
Mannheimia succiniciproducens MBEL55E	1.60E-006	Omp85	YP_087477.1	hypothetical protein MS0285
Marinobacter aquaeolei VT8	4.30E-119	BamB	YP_958409.1	Pyrrolo-quinoline quinone
Marinobacter aquaeolei VT8	1.00E-005	BamC	YP_958961.1	hypothetical protein Maqu_1690
Marinobacter aquaeolei VT8	4.00E-095	BamD	YP_958160.1	DNA uptake lipoprotein
Marinobacter aquaeolei VT8	4.20E-011	BamE	YP_960624.1	SmpA/OmlA domain-containing protein
Marinobacter aquaeolei VT8	6.60E-007	BamE	YP_961077.1	hypothetical protein Maqu_3821
Marinobacter aquaeolei VT8	1.90E-200	Omp85	YP_959802.1	surface antigen (D15)
Marinobacter aquaeolei VT8	7.50E-007	Omp85	YP_959280.1	surface antigen (D15)
Marinomonas mediterranea MMB-1	4.90E-049	BamB	YP_004312337.1	outer membrane assembly lipoprotein YfgL
Marinomonas mediterranea MMB-1	2.00E-071	BamD	YP_004314034.1	outer membrane assembly lipoprotein YfiO
Marinomonas mediterranea MMB-1	1.80E-014	BamE	YP_004311694.1	SmpA/OmlA domain-containing protein
Marinomonas mediterranea MMB-1	1.00E-177	Omp85	YP_004312263.1	outer membrane protein assembly complex, YaeT protein
Marinomonas posidonica IVIA-Po-181	4.10E-067	BamB	YP_004482394.1	outer membrane assembly lipoprotein YfgL
Marinomonas posidonica IVIA-Po-181	2.70E-073	BamD	YP_004480666.1	outer membrane assembly lipoprotein YfiO
Marinomonas posidonica IVIA-Po-181	1.10E-020	BamE	YP_004480342.1	SmpA/OmlA domain-containing protein
Marinomonas posidonica IVIA-Po-181	9.50E-176	Omp85	YP_004482572.1	outer membrane protein assembly complex, YaeT protein
Marinomonas sp. MWYL1	2.00E-057	BamB	YP_001340223.1	Pyrrolo-quinoline quinone
Marinomonas sp. MWYL1	1.40E-005	BamB	YP_001339858.1	Pyrrolo-quinoline quinone
Marinomonas sp. MWYL1	5.70E-070	BamD	YP_001342449.1	competence lipoprotein ComL
Marinomonas sp. MWYL1	8.40E-016	BamE	YP_001342800.1	SmpA/OmlA domain-containing protein
Marinomonas sp. MWYL1	6.50E-177	Omp85	YP_001340144.1	surface antigen (D15)
Methylococcus capsulatus str. Bath	9.50E-112	BamB	YP_115284.1	putative lipoprotein
Methylococcus capsulatus str. Bath	4.80E-006	BamB	YP_113281.1	methanol dehydrogenase protein, large subunit
Methylococcus capsulatus str. Bath	9.20E-006	BamB	YP_112833.1	methanol dehydrogenase protein, large subunit
Methylococcus capsulatus str. Bath	1.60E-166	BamD	YP_113450.1	competence lipoprotein ComL
Methylococcus capsulatus str. Bath	4.10E-005	BamD	YP_113695.1	hypothetical protein MCA1231
Methylococcus capsulatus str. Bath	6.00E-023	BamE	YP_115026.1	small protein A
Methylococcus capsulatus str. Bath	1.80E-208	Omp85	YP_114861.1	OMP85 family outer membrane protein
Methylomonas methanica MC09	1.60E-115	BamB	YP_004514798.1	outer membrane assembly lipoprotein YfgL
Methylomonas methanica MC09	3.50E-006	BamB	YP_004512440.1	PQQ-dependent dehydrogenase
Methylomonas methanica MC09	1.30E-005	BamB	YP_004515165.1	PQQ-dependent dehydrogenase
Methylomonas methanica MC09	5.20E-121	BamD	YP_004515268.1	outer membrane assembly lipoprotein YfiO
Methylomonas methanica MC09	5.70E-005	BamD	YP_004512418.1	tol-pal system protein YbgF
Methylomonas methanica MC09	5.20E-024	BamE	YP_004513140.1	SmpA/OmlA domain-containing protein

Methylomonas methanica MC09	3.40E-006	BamE	YP_004515290.1	hypothetical protein Metme_4448
Methylomonas methanica MC09	5.20E-006	BamE	YP_004511281.1	hypothetical protein Metme_0334
Methylomonas methanica MC09	5.20E-200	Omp85	YP_004513527.1	outer membrane protein assembly complex, YaeT protein
Methylomonas methanica MC09	1.00E-007	Omp85	YP_004512822.1	surface antigen (D15)
Moraxella catarrhalis RH4	1.20E-047	BamB	YP_003627325.1	outer membrane assembly lipoprotein YfgL
Moraxella catarrhalis RH4	2.30E-052	BamD	YP_003627211.1	DNA uptake lipoprotein-like protein
Moraxella catarrhalis RH4	2.30E-022	BamE	YP_003626497.1	SmpA/OmlA family protein
Moraxella catarrhalis RH4	1.80E-135	Omp85	YP_003626711.1	outer membrane protein assembly complex protein YaeT
Moraxella catarrhalis RH4	1.70E-006	Omp85	YP_003627384.1	D15 surface antigen family protein
Nitrosococcus halophilus Nc4	1.70E-126	BamB	YP_003527940.1	outer membrane assembly lipoprotein YfgL
Nitrosococcus halophilus Nc4	5.20E-005	BamC	YP_003526219.1	NlpB/DapX lipoprotein
Nitrosococcus halophilus Nc4	5.60E-115	BamD	YP_003526162.1	outer membrane assembly lipoprotein YfiO
Nitrosococcus halophilus Nc4	5.40E-006	BamD	YP_003525782.1	tol-pal system protein YbgF
Nitrosococcus halophilus Nc4	2.60E-026	BamE	YP_003526446.1	SmpA/OmlA domain-containing protein
Nitrosococcus halophilus Nc4	3.00E-227	Omp85	YP_003527935.1	outer membrane protein assembly complex, YaeT protein
Nitrosococcus halophilus Nc4	7.30E-007	Omp85	YP_003525807.1	surface antigen (D15)
Nitrosococcus oceani ATCC 19707	8.20E-124	BamB	YP_342864.1	Pyrrolo-quinoline quinone
Nitrosococcus oceani ATCC 19707	3.00E-005	BamC	YP_344506.1	NlpB/DapX lipoprotein
Nitrosococcus oceani ATCC 19707	2.20E-107	BamD	YP_342760.1	transmembrane protein
Nitrosococcus oceani ATCC 19707	1.20E-006	BamD	YP_342210.1	TPR repeat-containing protein
Nitrosococcus oceani ATCC 19707	1.70E-026	BamE	YP_343227.1	SmpA/OmlA
Nitrosococcus oceani ATCC 19707	2.80E-221	Omp85	YP_342859.1	Outer membrane protein
Nitrosococcus oceani ATCC 19707	2.30E-006	Omp85	YP_342426.1	Outer membrane protein
Nitrosococcus watsonii C-113	2.10E-137	BamB	YP_003761438.1	outer membrane assembly lipoprotein Yfgl
Nitrosococcus watsonii C-113	5.40E-005	BamB	YP_003760519.1	membrane-bound PQQ-dependent dehydrogenase, glucose/quinate/shikimate family
Nitrosococcus watsonii C-113	6.60E-109	BamD	YP_003761539.1	outer membrane assembly lipoprotein YfiO
Nitrosococcus watsonii C-113	4.50E-007	BamD	YP_003759435.1	tol-pal system protein YbgF
Nitrosococcus watsonii C-113	8.70E-025	BamE	YP_003761000.1	SmpA/OmlA domain-containing protein
Nitrosococcus watsonii C-113	5.10E-222	Omp85	YP_003761443.1	outer membrane protein assembly complex protein YaeT
Nitrosococcus watsonii C-113	5.00E-006	Omp85	YP_003761820.1	surface antigen
Pantoea ananatis LMG 20103	1.70E-198	BamB	YP_003521150.1	YfgL
Pantoea ananatis LMG 20103	5.80E-005	BamB	YP_003518607.1	Gcd
Pantoea ananatis LMG 20103	3.90E-138	BamC	YP_003521101.1	NlpB
Pantoea ananatis LMG 20103	7.20E-152	BamD	YP_003521308.1	YfiO
Pantoea ananatis LMG 20103	9.80E-052	BamE	YP_003521203.1	SmpA
Pantoea ananatis LMG 20103	7.60E-006	BamE	YP_003519980.1	OsmE
Pantoea ananatis LMG 20103	2.40E-005	BamE	YP_003520034.1	SodC

Pantoea ananatis LMG 20103	6.40E-005	BamE	YP_003522528.1	hypothetical Protein PANA_4233
Pantoea ananatis LMG 20103	7.70E-005	BamE	YP_003521835.1	AmiB
Pantoea ananatis LMG 20103	0.00E+000	Omp85	YP_003519095.1	YaeT
Pantoea ananatis LMG 20103	9.00E-007	Omp85	YP_003521807.1	YtfM
Pantoea sp. At-9b	1.50E-198	BamB	YP_004116781.1	outer membrane assembly lipoprotein YfgL
Pantoea sp. At-9b	1.20E-005	BamB	YP_004115705.1	glucose/quinate/shikimate family membrane-bound PQQ-dependent dehydrogenase
Pantoea sp. At-9b	2.20E-005	BamB	YP_004118314.1	membrane-bound PQQ-dependent dehydrogenase, glucose/quinate/shikimate family
Pantoea sp. At-9b	3.50E-005	BamB	YP_004119134.1	membrane-bound PQQ-dependent dehydrogenase, glucose/quinate/shikimate family
Pantoea sp. At-9b	7.50E-005	BamB	YP_004118995.1	membrane-bound PQQ-dependent dehydrogenase, glucose/quinate/shikimate family
Pantoea sp. At-9b	5.50E-187	BamC	YP_004116735.1	NlpBDapX family lipoprotein
Pantoea sp. At-9b	4.00E-155	BamD	YP_004116898.1	outer membrane assembly lipoprotein YfiO
Pantoea sp. At-9b	3.50E-005	BamD	YP_004115939.1	tetratricopeptide repeat-containing protein
Pantoea sp. At-9b	4.10E-062	BamE	YP_004116843.1	SmpA/OmlA domain-containing protein
Pantoea sp. At-9b	4.40E-007	BamE	YP_004115426.1	hypothetical protein Pat9b_1553
Pantoea sp. At-9b	9.90E-006	BamE	YP_004115504.1	SmpA/OmlA domain-containing protein
Pantoea sp. At-9b	0.00E+000	Omp85	YP_004114638.1	outer membrane protein assembly complex, YaeT protein
Pantoea sp. At-9b	1.30E-006	Omp85	YP_004117378.1	surface antigen (D15)
Pantoea sp. At-9b	4.10E-287	Omp85	YP_004117998.1	outer membrane protein assembly complex, YaeT protein
Pantoea sp. At-9b	1.90E-271	Omp85	YP_004119546.1	outer membrane protein assembly complex, YaeT protein
Pantoea vagans C9-1	6.40E-195	BamB	YP_003931898.1	outer membrane protein yfgL
Pantoea vagans C9-1	3.70E-005	BamB	YP_003930967.1	glucose dehydrogenase
Pantoea vagans C9-1	8.40E-005	BamB	YP_003933125.1	hypothetical protein Pvag_3557
Pantoea vagans C9-1	2.80E-188	BamC	YP_003931857.1	lipoprotein 34 precursor nlpB
Pantoea vagans C9-1	4.50E-155	BamD	YP_003931997.1	UPF0169 lipoprotein yfiO precursor
Pantoea vagans C9-1	7.80E-064	BamE	YP_003931953.1	Small protein A precursor
Pantoea vagans C9-1	4.00E-007	BamE	YP_003930766.1	Osmotically-inducible lipoprotein E precursor
Pantoea vagans C9-1	0.00E+000	Omp85	YP_003929879.1	outer membrane protein assembly factor yaeT precursor
Pantoea vagans C9-1	1.00E-006	Omp85	YP_003932375.1	hypothetical protein Pvag_2767
Pasteurella multocida subsp. multocida str. Pm70	6.50E-018	BamC	NP_245987.1	hypothetical protein PM1050
Pasteurella multocida subsp. multocida str. Pm70	3.80E-167	BamD	NP_246659.1	hypothetical protein PM1720
Pasteurella multocida subsp. multocida str. Pm70	1.30E-052	BamE	NP_246825.1	hypothetical protein PM1886
Pasteurella multocida subsp. multocida str. Pm70	9.20E-006	BamE	NP_245523.1	Plp4
Pasteurella multocida subsp. multocida str. Pm70	1.30E-179	Omp85	NP_246931.1	hypothetical protein PM1992
Pasteurella multocida subsp. multocida str. Pm70	5.60E-006	Omp85	NP_246748.1	hypothetical protein PM1809
Pectobacterium atrosepticum SCRI1043	6.20E-198	BamB	YP_051306.1	outer membrane protein assembly complex subunit YfgL
Pectobacterium atrosepticum SCRI1043	5.30E-005	BamB	YP_048911.1	putative outer membrane protein

Pectobacterium atrosepticum SCRI1043	8.60E-208	BamC	YP_049369.1	lipoprotein
Pectobacterium atrosepticum SCRI1043	2.00E-152	BamD	YP_051437.1	outer membrane protein assembly complex subunit YfiO
Pectobacterium atrosepticum SCRI1043	4.30E-067	BamE	YP_048949.1	hypothetical protein ECA0839
Pectobacterium atrosepticum SCRI1043	5.60E-006	BamE	YP_050506.1	DNA-binding transcriptional activator OsmE
Pectobacterium atrosepticum SCRI1043	2.90E-306	Omp85	YP_049146.1	outer membrane protein assembly factor YaeT
Pectobacterium atrosepticum SCRI1043	3.90E-007	Omp85	YP_051684.1	hypothetical protein ECA3596
Pectobacterium carotovorum subsp. carotovorum PC1	2.10E-195	BamB	YP_003018570.1	outer membrane assembly lipoprotein YfgL
Pectobacterium carotovorum subsp. carotovorum PC1	2.80E-005	BamB	YP_003016268.1	Ig family protein
Pectobacterium carotovorum subsp. carotovorum PC1	1.50E-208	BamC	YP_003016724.1	NlpBDapX family lipoprotein
Pectobacterium carotovorum subsp. carotovorum PC1	8.00E-153	BamD	YP_003018703.1	outer membrane assembly lipoprotein YfiO
Pectobacterium carotovorum subsp. carotovorum PC1	4.30E-067	BamE	YP_003016317.1	SmpA/OmlA domain-containing protein
Pectobacterium carotovorum subsp. carotovorum PC1	7.80E-006	BamE	YP_003017185.1	hypothetical protein PC1_1608
Pectobacterium carotovorum subsp. carotovorum PC1	1.70E-005	BamE	YP_003017476.1	SmpA/OmlA domain-containing protein
Pectobacterium carotovorum subsp. carotovorum PC1	0.00E+000	Omp85	YP_003016535.1	outer membrane protein assembly complex, YaeT protein
Pectobacterium carotovorum subsp. carotovorum PC1	1.80E-006	Omp85	YP_003018968.1	surface antigen (D15)
Pectobacterium wasabiae WPP163	1.20E-198	BamB	YP_003258682.1	outer membrane protein assembly complex subunit YfgL
Pectobacterium wasabiae WPP163	5.00E-205	BamC	YP_003260527.1	lipoprotein
Pectobacterium wasabiae WPP163	3.00E-152	BamD	YP_003258556.1	outer membrane protein assembly complex subunit YfiO
Pectobacterium wasabiae WPP163	1.00E-066	BamE	YP_003258386.1	hypothetical protein Pecwa_0962
Pectobacterium wasabiae WPP163	7.20E-006	BamE	YP_003259101.1	hypothetical protein Pecwa_1707
Pectobacterium wasabiae WPP163	6.80E-005	BamE	YP_003259569.1	DNA-binding transcriptional activator OsmE
Pectobacterium wasabiae WPP163	1.30E-306	Omp85	YP_003260697.1	outer membrane protein assembly factor YaeT
Pectobacterium wasabiae WPP163	8.70E-007	Omp85	YP_003260918.1	surface antigen (D15)
Photobacterium profundum SS9	2.30E-154	BamB	YP_128981.1	outer membrane protein assembly complex subunit YfgL
Photobacterium profundum SS9	3.10E-219	BamC	YP_131042.1	lipoprotein
Photobacterium profundum SS9	9.50E-164	BamD	YP_131152.1	hypothetical protein PBPRA3022
Photobacterium profundum SS9	4.10E-005	BamD	YP_132273.1	hypothetical protein PBPRB0600
Photobacterium profundum SS9	4.20E-072	BamE	YP_128916.1	hypothetical protein PBPRA0693
Photobacterium profundum SS9	1.00E-222	Omp85	YP_131096.1	outer membrane protein assembly factor YaeT
Photobacterium profundum SS9	6.50E-007	Omp85	YP_128613.1	hypothetical protein PBPRA0379
Photorhabdus asymbiotica subsp. asymbiotica ATCC 43949	2.30E-171	BamB	YP_003040226.1	outer membrane protein assembly complex subunit YfgL
Photorhabdus asymbiotica subsp. asymbiotica ATCC 43949	1.10E-219	BamC	YP_003040633.1	lipoprotein
Photorhabdus asymbiotica subsp. asymbiotica ATCC 43949	3.90E-144	BamD	YP_003042023.1	outer membrane protein assembly complex subunit YfiO
Photorhabdus asymbiotica subsp. asymbiotica ATCC 43949	4.80E-005	BamD	YP_003040917.1	tetratricopeptide repeat-containing protein
Photorhabdus asymbiotica subsp. asymbiotica ATCC 43949	9.30E-062	BamE	YP_003040109.1	hypothetical protein PAU_01272
Photorhabdus asymbiotica subsp. asymbiotica ATCC 43949	5.30E-305	Omp85	YP_003039489.1	outer membrane protein assembly factor YaeT
Photorhabdus asymbiotica subsp. asymbiotica ATCC 43949	1.40E-006	Omp85	YP_003042884.1	hypothetical protein PAU_04055
Photorhabdus luminescens subsp. laumondii TTO1	4.80E-172	BamB	NP_928679.1	outer membrane protein assembly complex subunit YfgL

Photorhabdus luminescens subsp. laumondii TTO1	7.50E-243	BamC	NP_929979.1	lipoprotein
Photorhabdus luminescens subsp. laumondii TTO1	2.20E-136	BamD	NP_928578.1	outer membrane protein assembly complex subunit YfiO
Photorhabdus luminescens subsp. laumondii TTO1	3.50E-062	BamE	NP_930593.1	hypothetical protein plu3375
Photorhabdus luminescens subsp. laumondii TTO1	8.80E-005	BamE	NP_931467.1	hypothetical protein plu4291
Photorhabdus luminescens subsp. laumondii TTO1	3.00E-303	Omp85	NP_928025.1	outer membrane protein assembly factor YaeT
Photorhabdus luminescens subsp. laumondii TTO1	8.30E-007	Omp85	NP_931718.1	hypothetical protein plu4554
Proteus mirabilis HI4320	8.30E-167	BamB	YP_002151569.1	outer membrane protein assembly complex subunit YfgL
Proteus mirabilis HI4320	1.10E-170	BamC	YP_002151294.1	lipoprotein
Proteus mirabilis HI4320	4.40E-147	BamD	YP_002150164.1	outer membrane protein assembly complex subunit YfiO
Proteus mirabilis HI4320	1.00E-053	BamE	YP_002151630.1	hypothetical protein PMI1903
Proteus mirabilis HI4320	2.80E-294	Omp85	YP_002151995.1	outer membrane protein assembly factor YaeT
Proteus mirabilis HI4320	1.60E-006	Omp85	YP_002153074.1	cell surface protein
Pseudoalteromonas atlantica T6c	3.40E-131	BamB	YP_662683.1	outer membrane protein assembly complex subunit YfgL
Pseudoalteromonas atlantica T6c	7.20E-008	BamB	YP_661429.1	Pyrrolo-quinoline quinone
Pseudoalteromonas atlantica T6c	2.50E-057	BamC	YP_662047.1	NlpBDapX lipoprotein
Pseudoalteromonas atlantica T6c	7.40E-138	BamD	YP_660914.1	putative lipoprotein
Pseudoalteromonas atlantica T6c	8.70E-005	BamD	YP_662499.1	hypothetical protein Patl_2937
Pseudoalteromonas atlantica T6c	4.40E-040	BamE	YP_661288.1	SmpA/OmlA
Pseudoalteromonas atlantica T6c	7.50E-179	Omp85	YP_660837.1	surface antigen (D15)
Pseudoalteromonas atlantica T6c	7.70E-006	Omp85	YP_659846.1	surface antigen (D15)
Pseudoalteromonas haloplanktis TAC125	8.90E-136	BamB	YP_341628.2	outer membrane protein assembly complex subunit YfgL
Pseudoalteromonas haloplanktis TAC125	1.80E-019	BamC	YP_339643.1	lipoprotein-34 NlpB
Pseudoalteromonas haloplanktis TAC125	3.90E-101	BamD	YP_339454.1	hypothetical protein PSHAa0932
Pseudoalteromonas haloplanktis TAC125	5.80E-005	BamD	YP_341439.1	hypothetical protein PSHAa2962
Pseudoalteromonas haloplanktis TAC125	2.30E-044	BamE	YP_339368.1	small protein A , precursor] (smpA)
Pseudoalteromonas haloplanktis TAC125	7.40E-167	Omp85	YP_340527.1	outer membrane protein assembly factor
Pseudoalteromonas haloplanktis TAC125	6.30E-006	Omp85	YP_340482.1	hypothetical protein PSHAa1983
Pseudoalteromonas sp. SM9913	6.20E-136	BamB	YP_004065119.1	outer membrane protein assembly complex subunit YfgL
Pseudoalteromonas sp. SM9913	4.90E-023	BamC	YP_004068977.1	lipoprotein-34 NlpB
Pseudoalteromonas sp. SM9913	1.70E-102	BamD	YP_004068101.1	hypothetical protein PSM_A1005
Pseudoalteromonas sp. SM9913	2.70E-042	BamE	YP_004069269.1	small protein A
Pseudoalteromonas sp. SM9913	4.50E-172	Omp85	YP_004069159.1	outer membrane protein assembly factor
Pseudoalteromonas sp. SM9913	7.50E-006	Omp85	YP_004069099.1	outer membrane protein
Pseudomonas aeruginosa LESB58	4.00E-177	BamB	YP_002438780.1	hypothetical protein PLES_11741
Pseudomonas aeruginosa LESB58	3.40E-008	BamB	YP_002440926.1	quinoprotein alcohol dehydrogenase
Pseudomonas aeruginosa LESB58	2.50E-005	BamB	YP_002440603.1	glucose dehydrogenase
Pseudomonas aeruginosa LESB58	1.90E-147	BamD	YP_002442509.1	competence protein ComL
Pseudomonas aeruginosa LESB58	5.50E-005	BamD	YP_002441924.1	hypothetical protein PLES_43401

Pseudomonas aeruginosa LESB58	9.30E-005	BamD	YP_002443475.1	putative lipoprotein
Pseudomonas aeruginosa LESB58	1.10E-025	BamE	YP_002442729.1	Outer membrane lipoprotein OmlA precursor
Pseudomonas aeruginosa LESB58	2.30E-006	BamE	YP_002442840.1	DNA-binding transcriptional activator OsmE
Pseudomonas aeruginosa LESB58	5.40E-005	BamE	YP_002443076.1	putative lipoprotein
Pseudomonas aeruginosa LESB58	4.40E-205	Omp85	YP_002438991.1	putative outer membrane protein precursor
Pseudomonas aeruginosa LESB58	1.20E-006	Omp85	YP_002440345.1	putative outer membrane protein
Pseudomonas aeruginosa PA7	5.30E-177	BamB	YP_001346698.1	hypothetical protein PSPA7_1314
Pseudomonas aeruginosa PA7	2.70E-008	BamB	YP_001348672.1	quinoprotein alcohol dehydrogenase
Pseudomonas aeruginosa PA7	1.10E-005	BamB	YP_001348471.1	quinoprotein glucose dehydrogenase
Pseudomonas aeruginosa PA7	8.10E-005	BamB	YP_001348311.1	glucose dehydrogenase
Pseudomonas aeruginosa PA7	1.60E-146	BamD	YP_001350516.1	competence protein ComL
Pseudomonas aeruginosa PA7	6.40E-005	BamD	YP_001349883.1	TPR repeat-containing protein
Pseudomonas aeruginosa PA7	1.20E-025	BamE	YP_001350807.1	outer membrane lipoprotein OmlA
Pseudomonas aeruginosa PA7	3.30E-006	BamE	YP_001350919.1	DNA-binding transcriptional activator OsmE
Pseudomonas aeruginosa PA7	3.00E-005	BamE	YP_001351160.1	putative lipoprotein
Pseudomonas aeruginosa PA7	7.10E-205	Omp85	YP_001346875.1	outer membrane protein assembly complex, YaeT protein
Pseudomonas aeruginosa PA7	1.50E-006	Omp85	YP_001348054.1	hypothetical protein PSPA7_2694
Pseudomonas aeruginosa PAO1	4.00E-177	BamB	NP_252489.1	hypothetical protein PA3800
Pseudomonas aeruginosa PAO1	2.20E-008	BamB	NP_250672.1	quinoprotein ethanol dehydrogenase
Pseudomonas aeruginosa PAO1	3.30E-005	BamB	NP_250980.1	glucose dehydrogenase
Pseudomonas aeruginosa PAO1	1.90E-147	BamD	NP_253235.1	competence protein ComL
Pseudomonas aeruginosa PAO1	3.50E-005	BamD	NP_249665.1	hypothetical protein PA0974
Pseudomonas aeruginosa PAO1	8.50E-005	BamD	NP_254189.1	hypothetical protein PA5502
Pseudomonas aeruginosa PAO1	1.10E-025	BamE	NP_253453.1	Outer membrane lipoprotein OmlA precursor
Pseudomonas aeruginosa PAO1	2.30E-006	BamE	NP_253563.1	OsmE family transcriptional regulator
Pseudomonas aeruginosa PAO1	5.40E-005	BamE	NP_253795.1	hypothetical protein PA5108
Pseudomonas aeruginosa PAO1	2.40E-205	Omp85	NP_252338.1	outer membrane protein Opr86
Pseudomonas aeruginosa PAO1	1.20E-006	Omp85	NP_251233.1	hypothetical protein PA2543
Pseudomonas aeruginosa UCBPP-PA14	4.80E-177	BamB	YP_789332.1	hypothetical protein PA14_14910
Pseudomonas aeruginosa UCBPP-PA14	2.20E-008	BamB	YP_791257.1	quinoprotein alcohol dehydrogenase
Pseudomonas aeruginosa UCBPP-PA14	3.10E-005	BamB	YP_790946.1	glucose dehydrogenase
Pseudomonas aeruginosa UCBPP-PA14	1.90E-147	BamD	YP_793006.1	competence protein ComL
Pseudomonas aeruginosa UCBPP-PA14	3.50E-005	BamD	YP_792300.1	hypothetical protein PA14_51690
Pseudomonas aeruginosa UCBPP-PA14	8.50E-005	BamD	YP_793979.1	putative lipoprotein
Pseudomonas aeruginosa UCBPP-PA14	1.10E-025	BamE	YP_793229.1	outer membrane lipoprotein OmlA precursor
Pseudomonas aeruginosa UCBPP-PA14	2.30E-006	BamE	YP_793344.1	DNA-binding transcriptional activator OsmE
Pseudomonas aeruginosa UCBPP-PA14	5.40E-005	BamE	YP_793578.1	putative lipoprotein
Pseudomonas aeruginosa UCBPP-PA14	6.60E-207	Omp85	YP_789516.1	putative outer membrane antigen

Pseudomonas aeruginosa UCBPP-PA14	1.20E-006	Omp85	YP_790694.1	hypothetical protein PA14_31680
Pseudomonas brassicacearum subsp. brassicacearum NFM421	1.50E-198	BamB	YP_004352112.1	hypothetical protein PSEBR_a939
Pseudomonas brassicacearum subsp. brassicacearum NFM421	3.10E-009	BamB	YP_004353962.1	alcohol dehydrogenase
Pseudomonas brassicacearum subsp. brassicacearum NFM421	3.40E-008	BamB	YP_004353957.1	quinoprotein ethanol dehydrogenase
Pseudomonas brassicacearum subsp. brassicacearum NFM421	4.40E-005	BamB	YP_004355949.1	Quinoprotein glucose dehydrogenase
Pseudomonas brassicacearum subsp. brassicacearum NFM421	3.50E-005	BamC	YP_004352541.1	lipoprotein
Pseudomonas brassicacearum subsp. brassicacearum NFM421	1.90E-146	BamD	YP_004356266.1	DNA uptake lipoprotein ComL
Pseudomonas brassicacearum subsp. brassicacearum NFM421	4.50E-026	BamE	YP_004351926.1	outer membrane lipoprotein
Pseudomonas brassicacearum subsp. brassicacearum NFM421	9.70E-005	BamE	YP_004354101.1	hypothetical protein PSEBR_a2798
Pseudomonas brassicacearum subsp. brassicacearum NFM421	6.40E-196	Omp85	YP_004355007.1	Surface antigen protein
Pseudomonas brassicacearum subsp. brassicacearum NFM421	2.90E-194	Omp85	YP_004352251.1	hypothetical protein PSEBR_a1073
Pseudomonas brassicacearum subsp. brassicacearum NFM421	1.50E-006	Omp85	YP_004355082.1	hypothetical protein PSEBR_a3727
Pseudomonas entomophila L48	9.20E-213	BamB	YP_606741.1	hypothetical protein PSEEN1024
Pseudomonas entomophila L48	6.10E-006	BamB	YP_606877.1	glucose dehydrogenase Gcd
Pseudomonas entomophila L48	4.10E-005	BamB	YP_608486.1	quinate dehydrogenase (pyrroloquinoline-quinone)
Pseudomonas entomophila L48	4.50E-005	BamC	YP_609543.1	lipoprotein
Pseudomonas entomophila L48	6.10E-144	BamD	YP_610133.1	competence lipoprotein ComL
Pseudomonas entomophila L48	8.60E-025	BamE	YP_606506.1	outer membrane lipoprotein OmlA
Pseudomonas entomophila L48	7.20E-006	BamE	YP_610336.1	DNA-binding transcriptional activator OsmE
Pseudomonas entomophila L48	7.90E-005	BamE	YP_610516.1	lipoprotein
Pseudomonas entomophila L48	5.70E-201	Omp85	YP_609685.1	surface antigen family outer membrane protein
Pseudomonas entomophila L48	2.90E-006	Omp85	YP_607711.1	surface antigen
Pseudomonas fluorescens Pf-5	6.80E-192	BamB	YP_262031.1	outer membrane assembly lipoprotein YfgL
Pseudomonas fluorescens Pf-5	3.40E-009	BamB	YP_259328.1	quinoprotein ethanol dehydrogenase PedH
Pseudomonas fluorescens Pf-5	1.20E-008	BamB	YP_259323.1	quinoprotein ethanol dehydrogenase PedE
Pseudomonas fluorescens Pf-5	8.90E-006	BamB	YP_262726.1	quinate/shikimate dehydrogenase
Pseudomonas fluorescens Pf-5	1.40E-005	BamB	YP_261996.1	quinoprotein glucose dehydrogenase
Pseudomonas fluorescens Pf-5	9.90E-005	BamC	YP_258586.1	lipoprotein
Pseudomonas fluorescens Pf-5	9.60E-148	BamD	YP_262375.1	competence lipoprotein ComL
Pseudomonas fluorescens Pf-5	1.80E-026	BamE	YP_257958.1	outer membrane lipoprotein OmlA
Pseudomonas fluorescens Pf-5	7.90E-192	Omp85	YP_258315.1	outer membrane protein assembly complex, YaeT protein
Pseudomonas fluorescens Pf-5	4.10E-006	Omp85	YP_261026.2	OMP85 family outer membrane protein
Pseudomonas fluorescens Pf0-1	5.80E-195	BamB	YP_350326.1	Pyrrolo-quinoline quinone
Pseudomonas fluorescens Pf0-1	3.20E-005	BamB	YP_350305.1	quinoprotein glucose dehydrogenase
Pseudomonas fluorescens Pf0-1	5.90E-005	BamB	YP_348443.1	Pyrrolo-quinoline quinone
Pseudomonas fluorescens Pf0-1	1.80E-005	BamC	YP_347104.1	putative lipoprotein
Pseudomonas fluorescens Pf0-1	1.70E-149	BamD	YP_350565.1	competence lipoprotein ComL
Pseudomonas fluorescens Pf0-1	1.00E-005	BamD	YP_347719.1	type II and III secretion system protein

Pseudomonas fluorescens Pf0-1	2.20E-025	BamE	YP_346491.1	SmpA/OmlA
Pseudomonas fluorescens Pf0-1	1.80E-191	Omp85	YP_346841.1	surface antigen (D15)
Pseudomonas fluorescens Pf0-1	5.60E-007	Omp85	YP_349376.1	surface antigen (D15)
Pseudomonas fluorescens SBW25	3.00E-191	BamB	YP_002874557.1	hypothetical protein PFLU5054
Pseudomonas fluorescens SBW25	8.50E-006	BamB	YP_002870745.1	quinoprotein glucose dehydrogenase
Pseudomonas fluorescens SBW25	4.30E-005	BamC	YP_002871123.1	putative lipoprotein
Pseudomonas fluorescens SBW25	3.90E-005	BamD	YP_001202223.1	hypothetical protein pQBR0478
Pseudomonas fluorescens SBW25	3.10E-148	BamD	YP_002870444.1	putative lipoprotein
Pseudomonas fluorescens SBW25	1.30E-006	BamE	YP_001202124.1	hypothetical protein pQBR0378
Pseudomonas fluorescens SBW25	4.80E-027	BamE	YP_002874775.1	outer membrane lipoprotein
Pseudomonas fluorescens SBW25	6.30E-201	Omp85	YP_002873654.1	putative surface antigen
Pseudomonas fluorescens SBW25	2.30E-195	Omp85	YP_002870930.1	putative surface exported protein
Pseudomonas fluorescens SBW25	2.10E-006	Omp85	YP_002873432.1	hypothetical protein PFLU3880
Pseudomonas fulva 12-X	3.10E-176	BamB	YP_004475345.1	outer membrane assembly lipoprotein YfgL
Pseudomonas fulva 12-X	4.10E-005	BamB	YP_004475831.1	membrane-bound PQQ-dependent dehydrogenase
Pseudomonas fulva 12-X	7.90E-005	BamB	YP_004474276.1	membrane-bound PQQ-dependent dehydrogenase
Pseudomonas fulva 12-X	4.80E-150	BamD	YP_004475866.1	outer membrane assembly lipoprotein YfiO
Pseudomonas fulva 12-X	1.10E-026	BamE	YP_004475660.1	SmpA/OmlA domain-containing protein
Pseudomonas fulva 12-X	1.30E-005	BamE	YP_004475709.1	SmpA/OmlA domain-containing protein
Pseudomonas fulva 12-X	6.30E-005	BamE	YP_004475180.1	hypothetical protein Psefu_3123
Pseudomonas fulva 12-X	1.20E-204	Omp85	YP_004475480.1	outer membrane protein assembly complex, YaeT protein
Pseudomonas fulva 12-X	1.50E-006	Omp85	YP_004474578.1	surface antigen (D15)
Pseudomonas mendocina NK-01	9.80E-197	BamB	YP_004381545.1	pyrrolo-quinoline quinone
Pseudomonas mendocina NK-01	6.90E-009	BamB	YP_004380720.1	pyrrolo-quinoline quinone
Pseudomonas mendocina NK-01	1.80E-007	BamB	YP_004380727.1	pyrrolo-quinoline quinone
Pseudomonas mendocina NK-01	4.80E-007	BamB	YP_004380463.1	methanol/ethanol family PQQ-dependent dehydrogenase
Pseudomonas mendocina NK-01	8.30E-141	BamD	YP_004378839.1	DNA uptake lipoprotein-like protein
Pseudomonas mendocina NK-01	1.20E-025	BamE	YP_004381716.1	SmpA/OmlA domain-containing protein
Pseudomonas mendocina NK-01	1.80E-005	BamE	YP_004380344.1	DNA-binding transcriptional activator OsmE
Pseudomonas mendocina NK-01	7.00E-197	Omp85	YP_004381105.1	surface antigen
Pseudomonas mendocina NK-01	1.10E-006	Omp85	YP_004380119.1	surface antigen
Pseudomonas mendocina ymp	1.70E-195	BamB	YP_001188977.1	Pyrrolo-quinoline quinone
Pseudomonas mendocina ymp	1.10E-008	BamB	YP_001187456.1	Pyrrolo-quinoline quinone
Pseudomonas mendocina ymp	3.20E-007	BamB	YP_001187450.1	Pyrrolo-quinoline quinone
Pseudomonas mendocina ymp	5.20E-143	BamD	YP_001186468.1	DNA uptake lipoprotein-like protein
Pseudomonas mendocina ymp	1.40E-025	BamE	YP_001189108.1	SmpA/OmlA domain-containing protein
Pseudomonas mendocina ymp	3.00E-006	BamE	YP_001187650.1	DNA-binding transcriptional activator OsmE
Pseudomonas mendocina ymp	7.80E-005	BamE	YP_001186012.1	hypothetical protein Pmen_0510

Pseudomonas mendocina ymp	3.20E-197	Omp85	YP_001188531.1	surface antigen (D15)
Pseudomonas mendocina ymp	1.30E-006	Omp85	YP_001187922.1	surface antigen (D15)
Pseudomonas putida F1	5.90E-215	BamB	YP_001266232.1	Pyrrolo-quinoline quinone
Pseudomonas putida F1	3.90E-009	BamB	YP_001268396.1	Pyrrolo-quinoline quinone
Pseudomonas putida F1	2.90E-008	BamB	YP_001268401.1	Pyrrolo-quinoline quinone
Pseudomonas putida F1	2.90E-006	BamB	YP_001269585.1	Pyrrolo-quinoline quinone
Pseudomonas putida F1	1.30E-005	BamC	YP_001266613.1	hypothetical protein Pput_1268
Pseudomonas putida F1	7.60E-147	BamD	YP_001266014.1	DNA uptake lipoprotein-like protein
Pseudomonas putida F1	2.20E-027	BamE	YP_001269901.1	SmpA/OmlA domain-containing protein
Pseudomonas putida F1	6.60E-006	BamE	YP_001270037.1	DNA-binding transcriptional activator OsmE
Pseudomonas putida F1	3.40E-005	BamE	YP_001270216.1	hypothetical protein Pput_4912
Pseudomonas putida F1	6.20E-203	Omp85	YP_001269486.1	surface antigen (D15)
Pseudomonas putida F1	1.60E-197	Omp85	YP_001267708.1	surface antigen (D15)
Pseudomonas putida F1	9.70E-007	Omp85	YP_001268114.1	surface antigen (D15)
Pseudomonas putida GB-1	4.30E-211	BamB	YP_001667145.1	outer membrane assembly lipoprotein YfgL
Pseudomonas putida GB-1	8.10E-009	BamB	YP_001669352.1	methanol/ethanol family PQQ-dependent dehydrogenase
Pseudomonas putida GB-1	1.50E-008	BamB	YP_001669357.1	methanol/ethanol family PQQ-dependent dehydrogenase
Pseudomonas putida GB-1	2.80E-006	BamB	YP_001670585.1	PQQ-dependent dehydrogenase glucose/quinate/shikimate family protein
Pseudomonas putida GB-1	9.60E-005	BamB	YP_001668588.1	PQQ-dependent dehydrogenase glucose/quinate/shikimate family protein
Pseudomonas putida GB-1	1.00E-005	BamC	YP_001670404.1	lipoprotein
Pseudomonas putida GB-1	1.60E-145	BamD	YP_001666915.1	competence lipoprotein ComL
Pseudomonas putida GB-1	2.30E-027	BamE	YP_001670954.1	SmpA/OmlA domain-containing protein
Pseudomonas putida GB-1	6.60E-006	BamE	YP_001671132.1	DNA-binding transcriptional activator OsmE
Pseudomonas putida GB-1	8.30E-006	BamE	YP_001669053.1	SmpA/OmlA domain-containing protein
Pseudomonas putida GB-1	3.40E-005	BamE	YP_001671308.1	hypothetical protein PputGB1_5088
Pseudomonas putida GB-1	4.80E-205	Omp85	YP_001668806.1	outer membrane protein assembly complex, YaeT protein
Pseudomonas putida GB-1	1.10E-204	Omp85	YP_001667398.1	outer membrane protein assembly complex, YaeT protein
Pseudomonas putida GB-1	1.30E-006	Omp85	YP_001669120.1	surface antigen (D15)
Pseudomonas putida KT2440	8.60E-215	BamB	NP_743017.1	hypothetical protein PP_0856
Pseudomonas putida KT2440	4.50E-009	BamB	NP_744823.1	quinoprotein ethanol dehydrogenase
Pseudomonas putida KT2440	1.50E-008	BamB	NP_744818.1	quinoprotein ethanol dehydrogenase
Pseudomonas putida KT2440	3.10E-006	BamB	NP_743602.1	glucose dehydrogenase (pyrroloquinoline-quinone)
Pseudomonas putida KT2440	1.80E-005	BamC	NP_743398.1	lipoprotein
Pseudomonas putida KT2440	2.00E-146	BamD	NP_742784.1	competence lipoprotein ComL
Pseudomonas putida KT2440	2.20E-027	BamE	NP_746839.1	SmpA/OmlA domain-containing protein
Pseudomonas putida KT2440	3.40E-006	BamE	NP_746960.1	DNA-binding transcriptional activator OsmE
Pseudomonas putida KT2440	3.40E-005	BamE	NP_747139.1	hypothetical protein PP_5038

Pseudomonas putida KT2440	2.10E-202	Omp85	NP_743756.1	surface antigen family outer membrane protein
Pseudomonas putida KT2440	9.10E-198	Omp85	NP_745513.1	surface antigen family protein
Pseudomonas putida KT2440	8.20E-007	Omp85	NP_745036.1	surface antigen (D15)
Pseudomonas putida S16	2.10E-214	BamB	YP_004700381.1	outer membrane assembly lipoprotein YfgL
Pseudomonas putida S16	1.50E-008	BamB	YP_004701662.1	quinoprotein ethanol dehydrogenase
Pseudomonas putida S16	2.00E-008	BamB	YP_004701667.1	putative quinoprotein ethanol dehydrogenase
Pseudomonas putida S16	5.30E-006	BamB	YP_004703623.1	glucose dehydrogenase (pyrroloquinoline-quinone)
Pseudomonas putida S16	1.90E-005	BamC	YP_004703400.1	lipoprotein
Pseudomonas putida S16	1.50E-146	BamD	YP_004700079.1	putative competence lipoprotein ComL
Pseudomonas putida S16	1.60E-005	BamE	YP_004702035.1	DNA-binding transcriptional activator OsmE
Pseudomonas putida S16	1.90E-005	BamE	YP_004704290.1	putative lipoprotein
Pseudomonas putida S16	2.90E-005	BamE	YP_004704111.1	DNA-binding transcriptional activator OsmE
Pseudomonas putida S16	1.00E-205	Omp85	YP_004700649.1	surface antigen family outer membrane protein
Pseudomonas putida S16	1.10E-006	Omp85	YP_004702447.1	surface antigen (D15)
Pseudomonas putida W619	8.80E-228	BamB	YP_001751171.1	outer membrane assembly lipoprotein YfgL
Pseudomonas putida W619	7.70E-007	BamB	YP_001749557.1	methanol/ethanol family PQQ-dependent dehydrogenase
Pseudomonas putida W619	5.00E-006	BamB	YP_001747955.1	PQQ-dependent dehydrogenase glucose/quinate/shikimate family protein
Pseudomonas putida W619	4.40E-005	BamB	YP_001749817.1	PQQ-dependent dehydrogenase glucose/quinate/shikimate family protein
Pseudomonas putida W619	9.30E-006	BamC	YP_001750823.1	lipoprotein
Pseudomonas putida W619	2.70E-146	BamD	YP_001751397.1	competence lipoprotein ComL
Pseudomonas putida W619	1.10E-027	BamE	YP_001747575.1	SmpA/OmlA domain-containing protein
Pseudomonas putida W619	5.50E-006	BamE	YP_001751496.1	DNA-binding transcriptional activator OsmE
Pseudomonas putida W619	5.50E-005	BamE	YP_001747300.1	lipoprotein
Pseudomonas putida W619	1.20E-203	Omp85	YP_001750923.1	outer membrane protein assembly complex, YaeT protein
Pseudomonas putida W619	9.20E-007	Omp85	YP_001749264.1	surface antigen (D15)
Pseudomonas stutzeri A1501	2.80E-177	BamB	YP_001173511.1	PQQ repeat-containing protein
Pseudomonas stutzeri A1501	4.40E-008	BamB	YP_001172772.1	quinoprotein alcohol dehydrogenase
Pseudomonas stutzeri A1501	5.90E-008	BamB	YP_001172766.1	quinoprotein alcohol dehydrogenase
Pseudomonas stutzeri A1501	2.10E-005	BamB	YP_001171530.1	glucose dehydrogenase
Pseudomonas stutzeri A1501	2.40E-005	BamB	YP_001171988.1	glucose dehydrogenase
Pseudomonas stutzeri A1501	6.10E-142	BamD	YP_001174110.1	competence protein ComL
Pseudomonas stutzeri A1501	9.80E-005	BamD	YP_001170827.1	cellulose synthase subunit BcsC
Pseudomonas stutzeri A1501	2.00E-026	BamE	YP_001173802.1	outer membrane lipoprotein OmlA
Pseudomonas stutzeri A1501	2.00E-192	Omp85	YP_001172072.1	surface antigen family outer membrane protein
Pseudomonas stutzeri A1501	2.40E-007	Omp85	YP_001172689.1	Outer membrane protein
Pseudomonas stutzeri ATCC 17588 = LMG 11199	7.00E-179	BamB	YP_004715444.1	PQQ repeat-containing protein
Pseudomonas stutzeri ATCC 17588 = LMG 11199	5.90E-008	BamB	YP_004714520.1	quinoprotein alcohol dehydrogenase

Pseudomonas stutzeri ATCC 17588 = LMG 11199	6.00E-008	BamB	YP_004714526.1	quinoprotein alcohol dehydrogenase
Pseudomonas stutzeri ATCC 17588 = LMG 11199	2.90E-006	BamB	YP_004715275.1	alcohol dehydrogenase
Pseudomonas stutzeri ATCC 17588 = LMG 11199	1.20E-005	BamB	YP_004713262.1	glucose dehydrogenase
Pseudomonas stutzeri ATCC 17588 = LMG 11199	1.90E-005	BamB	YP_004713757.1	glucose dehydrogenase
Pseudomonas stutzeri ATCC 17588 = LMG 11199	6.10E-142	BamD	YP_004715979.1	competence protein ComL
Pseudomonas stutzeri ATCC 17588 = LMG 11199	2.00E-026	BamE	YP_004715746.1	outer membrane lipoprotein OmlA
Pseudomonas stutzeri ATCC 17588 = LMG 11199	1.40E-201	Omp85	YP_004713819.1	surface antigen family outer membrane protein
Pseudomonas stutzeri ATCC 17588 = LMG 11199	8.10E-007	Omp85	YP_004714440.1	Outer membrane protein
Pseudomonas syringae pv. phaseolicola 1448A	2.00E-198	BamB	YP_273581.1	PQQ repeat-containing protein
Pseudomonas syringae pv. phaseolicola 1448A	8.70E-007	BamB	YP_275108.2	quinate/shikimate dehydrogenase
Pseudomonas syringae pv. phaseolicola 1448A	2.40E-005	BamB	YP_276063.1	quinoprotein glucose dehydrogenase
Pseudomonas syringae pv. phaseolicola 1448A	3.00E-142	BamD	YP_273031.1	competence lipoprotein ComL
Pseudomonas syringae pv. phaseolicola 1448A	8.90E-005	BamD	YP_273266.1	TPR domain-containing protein
Pseudomonas syringae pv. phaseolicola 1448A	2.60E-027	BamE	YP_276330.1	outer membrane lipoprotein OmlA
Pseudomonas syringae pv. phaseolicola 1448A	1.70E-006	BamE	YP_276605.1	DNA-binding transcriptional activator OsmE
Pseudomonas syringae pv. phaseolicola 1448A	9.60E-207	Omp85	YP_275970.1	outer membrane protein
Pseudomonas syringae pv. phaseolicola 1448A	5.50E-199	Omp85	YP_273714.1	OMP85 family outer membrane protein
Pseudomonas syringae pv. phaseolicola 1448A	2.30E-006	Omp85	YP_274827.1	OMP85 family outer membrane protein
Pseudomonas syringae pv. syringae B728a	8.90E-197	BamB	YP_234340.1	quinoprotein
Pseudomonas syringae pv. syringae B728a	1.80E-005	BamB	YP_236998.1	quinoprotein
Pseudomonas syringae pv. syringae B728a	8.20E-005	BamB	YP_235651.1	quinoprotein
Pseudomonas syringae pv. syringae B728a	6.90E-143	BamD	YP_233831.1	competence lipoprotein ComL, putative
Pseudomonas syringae pv. syringae B728a	2.60E-027	BamE	YP_237267.1	SmpA/OmlA
Pseudomonas syringae pv. syringae B728a	7.50E-007	BamE	YP_237514.1	DNA-binding transcriptional activator OsmE
Pseudomonas syringae pv. syringae B728a	5.70E-207	Omp85	YP_234440.1	surface antigen (D15):surface antigen variable number
Pseudomonas syringae pv. syringae B728a	1.10E-198	Omp85	YP_236863.1	surface antigen (D15):surface antigen variable number
Pseudomonas syringae pv. syringae B728a	3.40E-006	Omp85	YP_235555.1	surface antigen (D15):surface antigen variable number
Pseudomonas syringae pv. tomato str. DC3000	2.00E-198	BamB	NP_791263.1	PQQ enzyme repeat domain-containing protein
Pseudomonas syringae pv. tomato str. DC3000	2.00E-006	BamB	NP_792378.1	glucose dehydrogenase
Pseudomonas syringae pv. tomato str. DC3000	2.00E-005	BamB	NP_793957.1	glucose dehydrogenase
Pseudomonas syringae pv. tomato str. DC3000	9.20E-141	BamD	NP_790673.1	competence lipoprotein ComL
Pseudomonas syringae pv. tomato str. DC3000	6.60E-027	BamE	NP_794262.1	outer membrane lipoprotein OmlA
Pseudomonas syringae pv. tomato str. DC3000	5.20E-007	BamE	NP_794637.1	osmotically-inducible lipoprotein OsmE
Pseudomonas syringae pv. tomato str. DC3000	2.10E-208	Omp85	NP_791367.1	outer membrane protein
Pseudomonas syringae pv. tomato str. DC3000	3.60E-006	Omp85	NP_792555.1	hypothetical protein PSPTO_2749
Pseudoxanthomonas suwonensis 11-1	3.30E-108	BamB	YP_004146477.1	outer membrane assembly lipoprotein YfgL
Pseudoxanthomonas suwonensis 11-1	1.70E-081	BamD	YP_004145647.1	outer membrane assembly lipoprotein YfiO
Pseudoxanthomonas suwonensis 11-1	8.00E-022	BamE	YP_004146733.1	SmpA/OmlA domain-containing protein

Pseudoxanthomonas suwonensis 11-1	6.70E-178	Omp85	YP_004145981.1	outer membrane protein assembly complex, YaeT protein
Pseudoxanthomonas suwonensis 11-1	3.00E-006	Omp85	YP_004148019.1	surface antigen (D15)
Psychrobacter arcticus 273-4	1.50E-046	BamB	YP_263978.1	hypothetical protein Psyc_0685
Psychrobacter arcticus 273-4	4.10E-006	BamB	YP_264521.1	quinoprotein glucose dehydrogenase
Psychrobacter arcticus 273-4	3.30E-065	BamD	YP_264542.1	lipoprotein
Psychrobacter arcticus 273-4	5.30E-020	BamE	YP_263568.1	outer membrane lipoprotein OmlA
Psychrobacter arcticus 273-4	8.40E-146	Omp85	YP_264811.1	putative surface antigen (D15)
Psychrobacter arcticus 273-4	3.30E-006	Omp85	YP_264792.1	hypothetical protein Psyc_1510
Psychrobacter cryohalolentis K5	4.10E-043	BamB	YP_579922.1	Pyrrolo-quinoline quinone
Psychrobacter cryohalolentis K5	1.20E-065	BamD	YP_580391.1	hypothetical protein Pcryo_1126
Psychrobacter cryohalolentis K5	5.20E-020	BamE	YP_579558.1	SmpA/OmlA
Psychrobacter cryohalolentis K5	4.10E-142	Omp85	YP_580969.1	surface antigen (D15)
Psychrobacter cryohalolentis K5	3.20E-006	Omp85	YP_580950.1	surface antigen (D15)
Psychrobacter sp. PRwf-1	1.40E-060	BamB	YP_001280789.1	pyrrolo-quinoline quinone
Psychrobacter sp. PRwf-1	2.80E-006	BamB	YP_001280665.1	pyrrolo-quinoline quinone
Psychrobacter sp. PRwf-1	1.30E-058	BamD	YP_001280464.1	DNA uptake lipoprotein-like protein
Psychrobacter sp. PRwf-1	1.20E-015	BamE	YP_001280776.1	SmpA/OmlA domain-containing protein
Psychrobacter sp. PRwf-1	2.50E-145	Omp85	YP_001280686.1	surface antigen (D15)
Psychrobacter sp. PRwf-1	9.20E-006	Omp85	YP_001280498.1	surface antigen (D15)
Psychromonas ingrahamii 37	6.10E-193	BamB	YP_942606.1	Pyrrolo-quinoline quinone
Psychromonas ingrahamii 37	2.40E-005	BamB	YP_944384.1	glucose dehydrogenase
Psychromonas ingrahamii 37	5.80E-016	BamC	YP_943237.1	NlpB/DapX family lipoprotein
Psychromonas ingrahamii 37	2.00E-115	BamD	YP_944552.1	putative lipoprotein
Psychromonas ingrahamii 37	2.00E-005	BamD	YP_941758.1	lytic transglycosylase
Psychromonas ingrahamii 37	4.40E-005	BamD	YP_942612.1	LppC family lipoprotein
Psychromonas ingrahamii 37	8.20E-029	BamE	YP_943001.1	Outer membrane lipoprotein OmlA
Psychromonas ingrahamii 37	3.30E-192	Omp85	YP_944269.1	surface antigen (D15)
Psychromonas ingrahamii 37	9.30E-006	Omp85	YP_943286.1	surface antigen (D15)
Rahnella sp. Y9602	1.30E-187	BamB	YP_004211796.1	outer membrane assembly lipoprotein YfgL
Rahnella sp. Y9602	1.10E-005	BamB	YP_004215425.1	membrane-bound PQQ-dependent dehydrogenase, glucose/quinate/shikimate family
Rahnella sp. Y9602	1.60E-005	BamB	YP_004215484.1	membrane-bound PQQ-dependent dehydrogenase, glucose/quinate/shikimate family
Rahnella sp. Y9602	2.60E-198	BamC	YP_004213984.1	lipoprotein
Rahnella sp. Y9602	8.90E-155	BamD	YP_004211488.1	outer membrane assembly lipoprotein YfiO
Rahnella sp. Y9602	1.60E-064	BamE	YP_004214170.1	SmpA/OmlA domain-containing protein
Rahnella sp. Y9602	2.20E-008	BamE	YP_004212321.1	SmpA/OmlA domain-containing protein
Rahnella sp. Y9602	0.00E+000	Omp85	YP_004211623.1	outer membrane protein assembly complex, YaeT protein
Rahnella sp. Y9602	1.70E-006	Omp85	YP_004211209.1	surface antigen (D15)

Rahnella sp. Y9602	1.40E-285	Omp85	YP_004215526.1	outer membrane protein assembly complex, YaeT protein
Saccharophagus degradans 2-40	6.40E-122	BamB	YP_526911.1	PQQ repeat-containing protein
Saccharophagus degradans 2-40	1.00E-006	BamC	YP_528093.1	hypothetical protein Sde_2621
Saccharophagus degradans 2-40	8.90E-105	BamD	YP_528024.1	Acyl-(acyl-carrier-protein)--UDP-N-acetylglucosamine O-acyltransferase
Saccharophagus degradans 2-40	1.80E-005	BamD	YP_529032.1	hypothetical protein Sde_3565
Saccharophagus degradans 2-40	4.20E-025	BamE	YP_528209.1	small protein A (tmRNA-binding)-like protein
Saccharophagus degradans 2-40	4.90E-005	BamE	YP_526996.1	hypothetical protein Sde_1522
Saccharophagus degradans 2-40	3.40E-145	Omp85	YP_528061.1	dihydrodipicolinate synthase subfamily protein
Saccharophagus degradans 2-40	2.90E-005	Omp85	YP_525547.1	Outer membrane protein/protective antigen OMA87-like protein
Salmonella bongori NCTC 12419	9.40E-213	BamB	YP_004731122.1	putative lipoprotein
Salmonella bongori NCTC 12419	9.70E-005	BamB	YP_004729070.1	glucose dehydrogenase
Salmonella bongori NCTC 12419	2.20E-228	BamC	YP_004731094.1	putative lipoprotein
Salmonella bongori NCTC 12419	1.00E-171	BamD	YP_004731203.1	putative lipoprotein
Salmonella bongori NCTC 12419	1.10E-067	BamE	YP_004731225.1	small protein A
Salmonella bongori NCTC 12419	4.00E-005	BamE	YP_004730037.1	osmotically inducible lipoprotein E
Salmonella bongori NCTC 12419	0.00E+000	Omp85	YP_004729124.1	outer membrane protein
Salmonella bongori NCTC 12419	8.90E-007	Omp85	YP_004732638.1	hypothetical protein SBG_3853
Salmonella enterica subsp. arizonae serovar 62:z4,z23:-- str. RSK2980	5.70E-212	BamB	YP_001569438.1	outer membrane protein assembly complex subunit YfgL
Salmonella enterica subsp. arizonae serovar 62:z4,z23:-- str. RSK2980	8.00E-005	BamB	YP_001571815.1	glucose dehydrogenase
Salmonella enterica subsp. arizonae serovar 62:z4,z23:-- str. RSK2980	2.90E-226	BamC	YP_001569473.1	lipoprotein
Salmonella enterica subsp. arizonae serovar 62:z4,z23:-- str. RSK2980	4.10E-171	BamD	YP_001569350.1	outer membrane protein assembly complex subunit YfiO
Salmonella enterica subsp. arizonae serovar 62:z4,z23:-- str. RSK2980	3.80E-068	BamE	YP_001569331.1	hypothetical protein SARI_00243
Salmonella enterica subsp. arizonae serovar 62:z4,z23:-- str. RSK2980	2.50E-005	BamE	YP_001572580.1	hypothetical protein SARI_03626
Salmonella enterica subsp. arizonae serovar 62:z4,z23:-- str. RSK2980	4.00E-005	BamE	YP_001570700.1	DNA-binding transcriptional activator OsmE
Salmonella enterica subsp. arizonae serovar 62:z4,z23:-- str. RSK2980	0.00E+000	Omp85	YP_001571769.1	outer membrane protein assembly factor YaeT
Salmonella enterica subsp. arizonae serovar 62:z4,z23:-- str. RSK2980	1.10E-006	Omp85	YP_001572199.1	hypothetical protein SARI_03221
Salmonella enterica subsp. enterica serovar Agona str. SL483	5.70E-214	BamB	YP_002147473.1	outer membrane protein assembly complex subunit YfgL
Salmonella enterica subsp. enterica serovar Agona str. SL483	5.40E-005	BamB	YP_002145168.1	quinoprotein glucose dehydrogenase
Salmonella enterica subsp. enterica serovar Agona str. SL483	1.90E-227	BamC	YP_002147439.1	lipoprotein
Salmonella enterica subsp. enterica serovar Agona str. SL483	4.10E-171	BamD	YP_002147566.1	outer membrane protein assembly complex subunit YfiO
Salmonella enterica subsp. enterica serovar Agona str. SL483	5.10E-069	BamE	YP_002147620.1	hypothetical protein SeAg_B2830
Salmonella enterica subsp. enterica serovar Agona str. SL483	4.00E-005	BamE	YP_002146724.1	DNA-binding transcriptional activator OsmE
Salmonella enterica subsp. enterica serovar Agona str. SL483	0.00E+000	Omp85	YP_002145229.1	outer membrane protein assembly factor YaeT
Salmonella enterica subsp. enterica serovar Agona str. SL483	7.70E-007	Omp85	YP_002149324.1	outer membrane protein, OMP85 family
Salmonella enterica subsp. enterica serovar Choleraesuis str. SC-B67	1.20E-212	BamB	YP_217504.1	outer membrane protein assembly complex subunit YfgL
Salmonella enterica subsp. enterica serovar Choleraesuis str. SC-B67	8.20E-005	BamB	YP_215156.1	glucose dehydrogenase
Salmonella enterica subsp. enterica serovar Choleraesuis str. SC-B67	4.20E-226	BamC	YP_217470.1	lipoprotein

Salmonella enterica subsp. enterica serovar Choleraesuis str. SC-B67	3.30E-170	BamD	YP_217653.1	outer membrane protein assembly complex subunit YfiO
Salmonella enterica subsp. enterica serovar Choleraesuis str. SC-B67	5.10E-069	BamE	YP_217672.1	hypothetical protein SC2685
Salmonella enterica subsp. enterica serovar Choleraesuis str. SC-B67	4.00E-005	BamE	YP_216319.1	DNA-binding transcriptional activator OsmE
Salmonella enterica subsp. enterica serovar Choleraesuis str. SC-B67	0.00E+000	Omp85	YP_215211.1	outer membrane protein assembly factor YaeT
Salmonella enterica subsp. enterica serovar Choleraesuis str. SC-B67	8.70E-007	Omp85	YP_219271.1	hypothetical protein SC4284
Salmonella enterica subsp. enterica serovar Dublin str. CT_02021853	2.70E-212	BamB	YP_002216583.1	outer membrane protein assembly complex subunit YfgL
Salmonella enterica subsp. enterica serovar Dublin str. CT_02021853	7.80E-005	BamB	YP_002214125.1	quinoprotein glucose dehydrogenase
Salmonella enterica subsp. enterica serovar Dublin str. CT_02021853	9.20E-228	BamC	YP_002216555.1	lipoprotein
Salmonella enterica subsp. enterica serovar Dublin str. CT_02021853	4.10E-171	BamD	YP_002216674.1	outer membrane protein assembly complex subunit YfiO
Salmonella enterica subsp. enterica serovar Dublin str. CT_02021853	5.10E-069	BamE	YP_002216693.1	hypothetical protein SeD_A3012
Salmonella enterica subsp. enterica serovar Dublin str. CT_02021853	4.00E-005	BamE	YP_002215813.1	DNA-binding transcriptional activator OsmE
Salmonella enterica subsp. enterica serovar Dublin str. CT_02021853	0.00E+000	Omp85	YP_002214185.1	outer membrane protein assembly factor YaeT
Salmonella enterica subsp. enterica serovar Dublin str. CT_02021853	8.60E-007	Omp85	YP_002218292.1	OMP85 family outer membrane protein
Salmonella enterica subsp. enterica serovar Enteritidis str. P125109	5.70E-214	BamB	YP_002244579.1	outer membrane protein assembly complex subunit YfgL
Salmonella enterica subsp. enterica serovar Enteritidis str. P125109	7.80E-005	BamB	YP_002242336.1	glucose dehydrogenase
Salmonella enterica subsp. enterica serovar Enteritidis str. P125109	3.00E-226	BamC	YP_002244550.1	lipoprotein
Salmonella enterica subsp. enterica serovar Enteritidis str. P125109	4.10E-171	BamD	YP_002244664.1	outer membrane protein assembly complex subunit YfiO
Salmonella enterica subsp. enterica serovar Enteritidis str. P125109	5.10E-069	BamE	YP_002244683.1	hypothetical protein SEN2605
Salmonella enterica subsp. enterica serovar Enteritidis str. P125109	4.00E-005	BamE	YP_002243831.1	DNA-binding transcriptional activator OsmE
Salmonella enterica subsp. enterica serovar Enteritidis str. P125109	0.00E+000	Omp85	YP_002242393.1	outer membrane protein assembly factor YaeT
Salmonella enterica subsp. enterica serovar Enteritidis str. P125109	9.40E-007	Omp85	YP_002246207.1	hypothetical protein SEN4178
Salmonella enterica subsp. enterica serovar Gallinarum str. 287/91	5.70E-214	BamB	YP_002227420.1	outer membrane protein assembly complex subunit YfgL
Salmonella enterica subsp. enterica serovar Gallinarum str. 287/91	3.00E-226	BamC	YP_002227392.1	lipoprotein
Salmonella enterica subsp. enterica serovar Gallinarum str. 287/91	4.10E-171	BamD	YP_002227503.1	outer membrane protein assembly complex subunit YfiO
Salmonella enterica subsp. enterica serovar Gallinarum str. 287/91	5.10E-069	BamE	YP_002227522.1	hypothetical protein SG2662
Salmonella enterica subsp. enterica serovar Gallinarum str. 287/91	4.00E-005	BamE	YP_002226765.1	DNA-binding transcriptional activator OsmE
Salmonella enterica subsp. enterica serovar Gallinarum str. 287/91	0.00E+000	Omp85	YP_002225362.1	outer membrane protein assembly factor YaeT
Salmonella enterica subsp. enterica serovar Gallinarum str. 287/91	7.90E-007	Omp85	YP_002228967.1	hypothetical protein SG4254
Salmonella enterica subsp. enterica serovar Heidelberg str. SL476	5.70E-214	BamB	YP_002046578.1	outer membrane protein assembly complex subunit YfgL
Salmonella enterica subsp. enterica serovar Heidelberg str. SL476	8.90E-005	BamB	YP_002044146.1	quinoprotein glucose dehydrogenase
Salmonella enterica subsp. enterica serovar Heidelberg str. SL476	3.60E-227	BamC	YP_002046551.1	lipoprotein
Salmonella enterica subsp. enterica serovar Heidelberg str. SL476	4.10E-171	BamD	YP_002046669.1	outer membrane protein assembly complex subunit YfiO
Salmonella enterica subsp. enterica serovar Heidelberg str. SL476	5.10E-069	BamE	YP_002046688.1	hypothetical protein SeHA_C2900
Salmonella enterica subsp. enterica serovar Heidelberg str. SL476	4.00E-005	BamE	YP_002045319.1	DNA-binding transcriptional activator OsmE
Salmonella enterica subsp. enterica serovar Heidelberg str. SL476	0.00E+000	Omp85	YP_002044214.1	outer membrane protein assembly factor YaeT
Salmonella enterica subsp. enterica serovar Heidelberg str. SL476	7.90E-007	Omp85	YP_002048452.1	OMP85 family outer membrane protein
Salmonella enterica subsp. enterica serovar Newport str. SL254	5.70E-214	BamB	YP_002041778.1	outer membrane protein assembly complex subunit YfgL
Salmonella enterica subsp. enterica serovar Newport str. SL254	8.90E-005	BamB	YP_002039404.1	quinoprotein glucose dehydrogenase

Salmonella enterica subsp. enterica serovar Newport str. SL254	3.60E-227	BamC	YP_002041747.1	lipoprotein
Salmonella enterica subsp. enterica serovar Newport str. SL254	4.10E-171	BamD	YP_002041927.1	outer membrane protein assembly complex subunit YfiO
Salmonella enterica subsp. enterica serovar Newport str. SL254	5.10E-069	BamE	YP_002041946.1	hypothetical protein SNSL254_A2898
Salmonella enterica subsp. enterica serovar Newport str. SL254	4.00E-005	BamE	YP_002040571.1	DNA-binding transcriptional activator OsmE
Salmonella enterica subsp. enterica serovar Newport str. SL254	0.00E+000	Omp85	YP_002039464.1	outer membrane protein assembly factor YaeT
Salmonella enterica subsp. enterica serovar Newport str. SL254	8.60E-007	Omp85	YP_002043666.1	outer membrane protein OMP85 family
Salmonella enterica subsp. enterica serovar Paratyphi A str. AKU_12601	5.70E-214	BamB	YP_002141172.1	outer membrane protein assembly complex subunit YfgL
Salmonella enterica subsp. enterica serovar Paratyphi A str. AKU_12601	5.00E-005	BamB	YP_002141013.1	glucose dehydrogenase
Salmonella enterica subsp. enterica serovar Paratyphi A str. AKU_12601	4.20E-226	BamC	YP_002141201.1	lipoprotein
Salmonella enterica subsp. enterica serovar Paratyphi A str. AKU_12601	4.10E-171	BamD	YP_002143194.1	outer membrane protein assembly complex subunit YfiO
Salmonella enterica subsp. enterica serovar Paratyphi A str. AKU_12601	1.80E-041	BamE	YP_002143214.1	hypothetical protein SSPA2369
Salmonella enterica subsp. enterica serovar Paratyphi A str. AKU_12601	4.00E-005	BamE	YP_002142263.1	DNA-binding transcriptional activator OsmE
Salmonella enterica subsp. enterica serovar Paratyphi A str. AKU_12601	0.00E+000	Omp85	YP_002141068.1	outer membrane protein assembly factor YaeT
Salmonella enterica subsp. enterica serovar Paratyphi A str. AKU_12601	1.00E-006	Omp85	YP_002144762.1	hypothetical protein SSPA3928
Salmonella enterica subsp. enterica serovar Paratyphi A str. ATCC 9150	5.70E-214	BamB	YP_149676.1	outer membrane protein assembly complex subunit YfgL
Salmonella enterica subsp. enterica serovar Paratyphi A str. ATCC 9150	5.00E-005	BamB	YP_149517.1	glucose dehydrogenase
Salmonella enterica subsp. enterica serovar Paratyphi A str. ATCC 9150	4.20E-226	BamC	YP_149705.1	lipoprotein
Salmonella enterica subsp. enterica serovar Paratyphi A str. ATCC 9150	4.10E-171	BamD	YP_151705.1	outer membrane protein assembly complex subunit YfiO
Salmonella enterica subsp. enterica serovar Paratyphi A str. ATCC 9150	9.40E-068	BamE	YP_151725.2	hypothetical protein SPA2544
Salmonella enterica subsp. enterica serovar Paratyphi A str. ATCC 9150	4.00E-005	BamE	YP_150778.1	DNA-binding transcriptional activator OsmE
Salmonella enterica subsp. enterica serovar Paratyphi A str. ATCC 9150	0.00E+000	Omp85	YP_149572.1	outer membrane protein assembly factor YaeT
Salmonella enterica subsp. enterica serovar Paratyphi A str. ATCC 9150	8.30E-007	Omp85	YP_153277.1	hypothetical protein SPA4229
Salmonella enterica subsp. enterica serovar Paratyphi C strain RKS4594	4.50E-211	BamB	YP_002636741.1	outer membrane protein assembly complex subunit YfgL
Salmonella enterica subsp. enterica serovar Paratyphi C strain RKS4594	8.20E-005	BamB	YP_002635813.1	glucose dehydrogenase
Salmonella enterica subsp. enterica serovar Paratyphi C strain RKS4594	1.70E-224	BamC	YP_002636778.1	lipoprotein
Salmonella enterica subsp. enterica serovar Paratyphi C strain RKS4594	4.10E-171	BamD	YP_002638314.1	outer membrane protein assembly complex subunit YfiO
Salmonella enterica subsp. enterica serovar Paratyphi C strain RKS4594	5.10E-069	BamE	YP_002638334.1	hypothetical protein SPC_2795
Salmonella enterica subsp. enterica serovar Paratyphi C strain RKS4594	4.00E-005	BamE	YP_002637972.1	DNA-binding transcriptional activator OsmE
Salmonella enterica subsp. enterica serovar Paratyphi C strain RKS4594	0.00E+000	Omp85	YP_002635870.1	outer membrane protein assembly factor YaeT
Salmonella enterica subsp. enterica serovar Paratyphi C strain RKS4594	7.70E-007	Omp85	YP_002640051.1	hypothetical protein SPC_4560
Salmonella enterica subsp. enterica serovar Schwarzengrund str. CVM19633	8.90E-214	BamB	YP_002115582.1	outer membrane protein assembly complex subunit YfgL
Salmonella enterica subsp. enterica serovar Schwarzengrund str. CVM19633	9.60E-005	BamB	YP_002113188.1	quinoprotein glucose dehydrogenase
Salmonella enterica subsp. enterica serovar Schwarzengrund str. CVM19633	3.60E-227	BamC	YP_002115551.1	lipoprotein
Salmonella enterica subsp. enterica serovar Schwarzengrund str. CVM19633	1.80E-169	BamD	YP_002115674.1	outer membrane protein assembly complex subunit YfiO
Salmonella enterica subsp. enterica serovar Schwarzengrund str. CVM19633	5.10E-069	BamE	YP_002115694.1	hypothetical protein SeSA_A2880
Salmonella enterica subsp. enterica serovar Schwarzengrund str. CVM19633	4.00E-005	BamE	YP_002114331.1	DNA-binding transcriptional activator OsmE
Salmonella enterica subsp. enterica serovar Schwarzengrund str. CVM19633	0.00E+000	Omp85	YP_002113247.1	outer membrane protein assembly factor YaeT
Salmonella enterica subsp. enterica serovar Schwarzengrund str. CVM19633	9.00E-007	Omp85	YP_002117349.1	outer membrane protein, OMP85 family protein

Salmonella enterica subsp. enterica serovar Typhi str. CT18	5.70E-214	BamB	NP_457052.1	outer membrane protein assembly complex subunit YfgL
Salmonella enterica subsp. enterica serovar Typhi str. CT18	9.10E-005	BamB	NP_454782.1	glucose dehydrogenase
Salmonella enterica subsp. enterica serovar Typhi str. CT18	3.00E-226	BamC	NP_457022.1	lipoprotein
Salmonella enterica subsp. enterica serovar Typhi str. CT18	3.00E-171	BamD	NP_457134.1	outer membrane protein assembly complex subunit YfiO
Salmonella enterica subsp. enterica serovar Typhi str. CT18	5.10E-069	BamE	NP_457154.1	hypothetical protein STY2871
Salmonella enterica subsp. enterica serovar Typhi str. CT18	4.00E-005	BamE	NP_456201.1	DNA-binding transcriptional activator OsmE
Salmonella enterica subsp. enterica serovar Typhi str. CT18	0.00E+000	Omp85	NP_454831.1	outer membrane protein assembly factor YaeT
Salmonella enterica subsp. enterica serovar Typhi str. CT18	9.60E-007	Omp85	NP_458846.1	hypothetical protein STY4768
Salmonella enterica subsp. enterica serovar Typhi str. Ty2	5.70E-214	BamB	NP_804210.1	outer membrane protein assembly complex subunit YfgL
Salmonella enterica subsp. enterica serovar Typhi str. Ty2	9.10E-005	BamB	NP_804057.1	glucose dehydrogenase
Salmonella enterica subsp. enterica serovar Typhi str. Ty2	1.90E-227	BamC	NP_804240.1	lipoprotein
Salmonella enterica subsp. enterica serovar Typhi str. Ty2	3.00E-171	BamD	NP_806330.1	outer membrane protein assembly complex subunit YfiO
Salmonella enterica subsp. enterica serovar Typhi str. Ty2	5.10E-069	BamE	NP_806350.1	hypothetical protein t2639
Salmonella enterica subsp. enterica serovar Typhi str. Ty2	4.00E-005	BamE	NP_804997.1	DNA-binding transcriptional activator OsmE
Salmonella enterica subsp. enterica serovar Typhi str. Ty2	0.00E+000	Omp85	NP_804106.1	outer membrane protein assembly factor YaeT
Salmonella enterica subsp. enterica serovar Typhi str. Ty2	9.60E-007	Omp85	NP_808050.1	hypothetical protein t4463
Salmonella enterica subsp. enterica serovar Typhimurium str. LT2	1.20E-213	BamB	NP_461455.1	outer membrane protein assembly complex subunit YfgL
Salmonella enterica subsp. enterica serovar Typhimurium str. LT2	8.90E-005	BamB	NP_459174.1	glucose dehydrogenase
Salmonella enterica subsp. enterica serovar Typhimurium str. LT2	3.60E-227	BamC	NP_461423.1	lipoprotein
Salmonella enterica subsp. enterica serovar Typhimurium str. LT2	4.10E-171	BamD	NP_461594.1	outer membrane protein assembly complex subunit YfiO
Salmonella enterica subsp. enterica serovar Typhimurium str. LT2	5.10E-069	BamE	NP_461615.1	hypothetical protein STM2685
Salmonella enterica subsp. enterica serovar Typhimurium str. LT2	4.00E-005	BamE	NP_460277.1	DNA-binding transcriptional activator OsmE
Salmonella enterica subsp. enterica serovar Typhimurium str. LT2	0.00E+000	Omp85	NP_459229.1	outer membrane protein assembly factor YaeT
Salmonella enterica subsp. enterica serovar Typhimurium str. LT2	9.60E-007	Omp85	NP_463270.1	outer membrane protein
Serratia proteamaculans 568	3.70E-193	BamB	YP_001479830.1	outer membrane protein assembly complex subunit YfgL
Serratia proteamaculans 568	8.10E-005	BamB	YP_001481070.1	Pyrrolo-quinoline quinone
Serratia proteamaculans 568	7.50E-213	BamC	YP_001479734.1	lipoprotein
Serratia proteamaculans 568	6.10E-155	BamD	YP_001477117.1	outer membrane protein assembly complex subunit YfiO
Serratia proteamaculans 568	4.90E-063	BamE	YP_001479911.1	hypothetical protein Spro_3687
Serratia proteamaculans 568	1.20E-006	BamE	YP_001479484.1	DNA-binding transcriptional activator OsmE
Serratia proteamaculans 568	1.80E-005	BamE	YP_001480311.1	hypothetical protein Spro_4088
Serratia proteamaculans 568	2.40E-005	BamE	YP_001476573.1	hypothetical protein Spro_0335
Serratia proteamaculans 568	2.60E-005	BamE	YP_001480393.1	hypothetical protein Spro_4171
Serratia proteamaculans 568	6.40E-005	BamE	YP_001481034.1	hypothetical protein Spro_4813
Serratia proteamaculans 568	0.00E+000	Omp85	YP_001480006.1	outer membrane protein assembly factor YaeT
Serratia proteamaculans 568	3.30E-007	Omp85	YP_001476695.1	surface antigen (D15)
Serratia sp. AS12	1.50E-195	BamB	YP_004502221.1	outer membrane assembly lipoprotein YfgL

Serratia sp. AS12	9.00E-005	BamB	YP_004500827.1	membrane-bound PQQ-dependent dehydrogenase, glucose/quinate/shikimate family
Serratia sp. AS12	1.60E-214	BamC	YP_004502119.1	NlpBDapX family lipoprotein
Serratia sp. AS12	2.40E-152	BamD	YP_004499269.1	outer membrane assembly lipoprotein YfiO
Serratia sp. AS12	2.80E-063	BamE	YP_004502288.1	SmpA/OmlA domain-containing protein
Serratia sp. AS12	1.40E-006	BamE	YP_004501872.1	SmpA/OmlA domain-containing protein
Serratia sp. AS12	6.80E-005	BamE	YP_004498763.1	integrating conjugative element protein
Serratia sp. AS12	0.00E+000	Omp85	YP_004502380.1	Outer membrane protein assembly factor yaeT
Serratia sp. AS12	7.10E-282	Omp85	YP_004500840.1	outer membrane protein assembly complex, YaeT protein
Serratia sp. AS12	3.90E-007	Omp85	YP_004498864.1	surface antigen (D15)
Serratia sp. AS9	1.50E-195	BamB	YP_004507173.1	outer membrane assembly lipoprotein YfgL
Serratia sp. AS9	9.00E-005	BamB	YP_004505780.1	membrane-bound PQQ-dependent dehydrogenase
Serratia sp. AS9	1.60E-214	BamC	YP_004507071.1	NlpBDapX family lipoprotein
Serratia sp. AS9	2.40E-152	BamD	YP_004504221.1	outer membrane assembly lipoprotein YfiO
Serratia sp. AS9	2.80E-063	BamE	YP_004507240.1	SmpA/OmlA domain-containing protein
Serratia sp. AS9	1.40E-006	BamE	YP_004506824.1	SmpA/OmlA domain-containing protein
Serratia sp. AS9	6.80E-005	BamE	YP_004503715.1	integrating conjugative element protein
Serratia sp. AS9	0.00E+000	Omp85	YP_004507332.1	Outer membrane protein assembly factor yaeT
Serratia sp. AS9	7.10E-282	Omp85	YP_004505793.1	outer membrane protein assembly complex, YaeT protein
Serratia sp. AS9	3.90E-007	Omp85	YP_004503816.1	surface antigen (D15)
Shewanella amazonensis SB2B	4.80E-202	BamB	YP_928236.1	outer membrane protein assembly complex subunit YfgL
Shewanella amazonensis SB2B	2.00E-039	BamC	YP_927782.1	lipoprotein-34 NlpB
Shewanella amazonensis SB2B	9.90E-145	BamD	YP_928478.1	putative lipoprotein
Shewanella amazonensis SB2B	1.10E-036	BamE	YP_928259.1	small protein A
Shewanella amazonensis SB2B	4.40E-229	Omp85	YP_927024.1	surface antigen
Shewanella amazonensis SB2B	1.90E-034	Omp85	YP_927493.1	hypothetical protein Sama_1616
Shewanella baltica OS155	1.80E-235	BamB	YP_001051338.1	outer membrane protein assembly complex subunit YfgL
Shewanella baltica OS155	2.30E-029	BamC	YP_001050913.1	NlpB/DapX family lipoprotein
Shewanella baltica OS155	1.40E-163	BamD	YP_001049409.1	putative lipoprotein
Shewanella baltica OS155	9.00E-005	BamD	YP_001049159.1	peptidase S45, penicillin amidase
Shewanella baltica OS155	1.00E-036	BamE	YP_001049697.1	SmpA/OmlA domain-containing protein
Shewanella baltica OS155	5.40E-215	Omp85	YP_001049845.1	surface antigen (D15)
Shewanella baltica OS155	5.30E-069	Omp85	YP_001050278.1	surface antigen (D15)
Shewanella baltica OS185	3.50E-235	BamB	YP_001367196.1	outer membrane protein assembly complex subunit YfgL
Shewanella baltica OS185	1.00E-029	BamC	YP_001366787.1	NlpB/DapX family lipoprotein
Shewanella baltica OS185	1.40E-163	BamD	YP_001365300.1	putative lipoprotein
Shewanella baltica OS185	9.00E-005	BamD	YP_001367743.1	peptidase S45 penicillin amidase
Shewanella baltica OS185	1.00E-036	BamE	YP_001365514.1	SmpA/OmlA domain-containing protein

Shewanella baltica OS185	1.10E-220	Omp85	YP_001365666.1	surface antigen (D15)
Shewanella baltica OS185	2.10E-069	Omp85	YP_001366135.1	surface antigen (D15)
Shewanella baltica OS195	3.90E-235	BamB	YP_001555567.1	outer membrane protein assembly complex subunit YfgL
Shewanella baltica OS195	2.40E-032	BamC	YP_001555094.1	NlpB/DapX family lipoprotein
Shewanella baltica OS195	1.40E-163	BamD	YP_001553554.1	putative lipoprotein
Shewanella baltica OS195	1.00E-036	BamE	YP_001553772.1	SmpA/OmlA domain-containing protein
Shewanella baltica OS195	1.10E-220	Omp85	YP_001553922.1	surface antigen (D15)
Shewanella baltica OS195	3.80E-068	Omp85	YP_001554367.1	surface antigen (D15)
Shewanella baltica OS223	3.50E-235	BamB	YP_002357307.1	outer membrane protein assembly complex subunit YfgL
Shewanella baltica OS223	1.00E-029	BamC	YP_002357721.1	NlpBDapX family lipoprotein
Shewanella baltica OS223	1.40E-163	BamD	YP_002359180.1	outer membrane assembly lipoprotein YfiO
Shewanella baltica OS223	1.00E-036	BamE	YP_002358957.1	SmpA/OmlA domain-containing protein
Shewanella baltica OS223	2.50E-221	Omp85	YP_002358803.1	outer membrane protein assembly complex, YaeT protein
Shewanella baltica OS223	2.10E-069	Omp85	YP_002358308.1	surface antigen (D15)
Shewanella denitrificans OS217	7.10E-188	BamB	YP_562268.1	outer membrane protein assembly complex subunit YfgL
Shewanella denitrificans OS217	3.30E-019	BamC	YP_562743.1	NlpBDapX lipoprotein
Shewanella denitrificans OS217	2.80E-154	BamD	YP_563866.1	putative lipoprotein
Shewanella denitrificans OS217	7.30E-030	BamE	YP_562251.1	SmpA/OmlA
Shewanella denitrificans OS217	5.30E-006	BamE	YP_562525.1	hypothetical protein Sden_1517
Shewanella denitrificans OS217	1.20E-202	Omp85	YP_562570.1	surface antigen (D15)
Shewanella denitrificans OS217	1.30E-053	Omp85	YP_562513.1	surface antigen (D15)
Shewanella frigidimarina NCIMB 400	2.80E-189	BamB	YP_749810.1	outer membrane protein assembly complex subunit YfgL
Shewanella frigidimarina NCIMB 400	1.20E-030	BamC	YP_750551.1	NlpB/DapX family lipoprotein
Shewanella frigidimarina NCIMB 400	7.60E-157	BamD	YP_749364.1	putative lipoprotein
Shewanella frigidimarina NCIMB 400	1.20E-036	BamE	YP_749788.1	SmpA/OmlA domain-containing protein
Shewanella frigidimarina NCIMB 400	1.10E-209	Omp85	YP_749969.1	surface antigen (D15)
Shewanella frigidimarina NCIMB 400	2.60E-170	Omp85	YP_750134.1	surface antigen (D15)
Shewanella halifaxensis HAW-EB4	3.90E-199	BamB	YP_001673596.1	outer membrane protein assembly complex subunit YfgL
Shewanella halifaxensis HAW-EB4	7.10E-018	BamC	YP_001674538.1	NlpB/DapX family lipoprotein
Shewanella halifaxensis HAW-EB4	2.20E-148	BamD	YP_001675455.1	putative lipoprotein
Shewanella halifaxensis HAW-EB4	1.90E-005	BamD	YP_001673591.1	type IV pilus biogenesis/stability protein PilW
Shewanella halifaxensis HAW-EB4	1.00E-037	BamE	YP_001673574.1	SmpA/OmlA domain-containing protein
Shewanella halifaxensis HAW-EB4	1.30E-220	Omp85	YP_001675181.1	surface antigen (D15)
Shewanella halifaxensis HAW-EB4	3.50E-041	Omp85	YP_001674068.1	surface antigen (D15)
Shewanella loihica PV-4	1.70E-197	BamB	YP_001093422.1	outer membrane protein assembly complex subunit YfgL
Shewanella loihica PV-4	1.70E-019	BamC	YP_001093974.1	NlpB/DapX family lipoprotein
Shewanella loihica PV-4	1.50E-150	BamD	YP_001095076.1	putative lipoprotein
Shewanella loihica PV-4	1.70E-035	BamE	YP_001093400.1	SmpA/OmlA domain-containing protein

Shewanella loihica PV-4	1.20E-216	Omp85	YP_001094752.1	surface antigen (D15)
Shewanella loihica PV-4	3.80E-049	Omp85	YP_001094297.1	surface antigen (D15)
Shewanella oneidensis MR-1	4.50E-225	BamB	NP_718863.1	outer membrane protein assembly complex subunit YfgL
Shewanella oneidensis MR-1	1.20E-032	BamC	NP_717488.1	lipoprotein-34 NlpB
Shewanella oneidensis MR-1	2.10E-173	BamD	NP_719124.1	hypothetical protein SO_3580
Shewanella oneidensis MR-1	1.50E-036	BamE	NP_717092.1	small protein A
Shewanella oneidensis MR-1	8.30E-214	Omp85	NP_717248.1	surface antigen
Shewanella oneidensis MR-1	6.50E-067	Omp85	NP_717717.1	hypothetical protein SO_2114
Shewanella pealeana ATCC 700345	1.20E-198	BamB	YP_001501170.1	outer membrane protein assembly complex subunit YfgL
Shewanella pealeana ATCC 700345	1.80E-018	BamC	YP_001501838.1	NlpB/DapX family lipoprotein
Shewanella pealeana ATCC 700345	8.10E-149	BamD	YP_001503018.1	putative lipoprotein
Shewanella pealeana ATCC 700345	2.00E-005	BamD	YP_001501165.1	type IV pilus biogenesis/stability protein PilW
Shewanella pealeana ATCC 700345	4.60E-005	BamD	YP_001503044.1	diguanylate cyclase
Shewanella pealeana ATCC 700345	2.80E-038	BamE	YP_001501147.1	SmpA/OmlA domain-containing protein
Shewanella pealeana ATCC 700345	1.50E-222	Omp85	YP_001502730.1	surface antigen (D15)
Shewanella pealeana ATCC 700345	7.10E-038	Omp85	YP_001502291.1	surface antigen (D15)
Shewanella piezotolerans WP3	9.60E-203	BamB	YP_002310827.1	outer membrane protein assembly complex subunit YfgL
Shewanella piezotolerans WP3	3.90E-018	BamC	YP_002312051.1	lipoprotein-34 NlpB
Shewanella piezotolerans WP3	4.30E-146	BamD	YP_002313200.1	hypothetical protein swp_3941
Shewanella piezotolerans WP3	3.50E-035	BamE	YP_002310805.1	SmpA/OmlA
Shewanella piezotolerans WP3	7.00E-005	BamE	YP_002314049.1	polysaccharide deacetylase
Shewanella piezotolerans WP3	9.60E-005	BamE	YP_002309463.1	hypothetical protein swp_0028
Shewanella piezotolerans WP3	2.00E-222	Omp85	YP_002312794.1	surface antigen
Shewanella piezotolerans WP3	1.50E-039	Omp85	YP_002312096.1	Surface antigen (D15):Surface antigen variable number
Shewanella putrefaciens CN-32	3.30E-227	BamB	YP_001184169.1	outer membrane protein assembly complex subunit YfgL
Shewanella putrefaciens CN-32	6.50E-030	BamC	YP_001183227.1	NlpB/DapX family lipoprotein
Shewanella putrefaciens CN-32	3.70E-168	BamD	YP_001182549.1	putative lipoprotein
Shewanella putrefaciens CN-32	2.20E-038	BamE	YP_001182759.1	SmpA/OmlA domain-containing protein
Shewanella putrefaciens CN-32	1.30E-224	Omp85	YP_001182883.1	surface antigen (D15)
Shewanella putrefaciens CN-32	6.30E-069	Omp85	YP_001183419.1	surface antigen (D15)
Shewanella sediminis HAW-EB3	3.00E-200	BamB	YP_001473174.1	outer membrane protein assembly complex subunit YfgL
Shewanella sediminis HAW-EB3	1.30E-022	BamC	YP_001474166.1	NlpB/DapX family lipoprotein
Shewanella sediminis HAW-EB3	5.00E-147	BamD	YP_001475232.1	putative lipoprotein
Shewanella sediminis HAW-EB3	4.00E-037	BamE	YP_001473149.1	SmpA/OmlA domain-containing protein
Shewanella sediminis HAW-EB3	2.70E-214	Omp85	YP_001474885.1	surface antigen (D15)
Shewanella sp. ANA-3	8.80E-229	BamB	YP_868873.1	outer membrane protein assembly complex subunit YfgL
Shewanella sp. ANA-3	2.10E-028	BamC	YP_869296.1	NlpB/DapX family lipoprotein
Shewanella sp. ANA-3	6.30E-172	BamD	YP_870802.1	putative lipoprotein

Shewanella sp. ANA-3	8.70E-037	BamE	YP_870581.1	SmpA/OmlA domain-containing protein
Shewanella sp. ANA-3	8.30E-218	Omp85	YP_870440.1	surface antigen (D15)
Shewanella sp. ANA-3	1.10E-055	Omp85	YP_869531.1	surface antigen (D15)
Shewanella sp. MR-4	4.90E-227	BamB	YP_733368.1	outer membrane protein assembly complex subunit YfgL
Shewanella sp. MR-4	2.50E-028	BamC	YP_734467.1	NlpB/DapX family lipoprotein
Shewanella sp. MR-4	6.40E-171	BamD	YP_735122.1	putative lipoprotein
Shewanella sp. MR-4	1.50E-036	BamE	YP_734901.1	SmpA/OmlA domain-containing protein
Shewanella sp. MR-4	2.20E-217	Omp85	YP_734761.1	surface antigen (D15)
Shewanella sp. MR-4	3.00E-052	Omp85	YP_733967.1	surface antigen (D15)
Shewanella sp. MR-7	2.80E-225	BamB	YP_737358.1	outer membrane protein assembly complex subunit YfgL
Shewanella sp. MR-7	2.20E-027	BamC	YP_738453.1	NlpB/DapX family lipoprotein
Shewanella sp. MR-7	6.40E-171	BamD	YP_739116.1	putative lipoprotein
Shewanella sp. MR-7	1.50E-036	BamE	YP_738892.1	SmpA/OmlA domain-containing protein
Shewanella sp. MR-7	2.20E-217	Omp85	YP_738742.1	surface antigen (D15)
Shewanella sp. MR-7	1.90E-052	Omp85	YP_738186.1	surface antigen (D15)
Shewanella sp. W3-18-1	3.30E-227	BamB	YP_962755.1	outer membrane protein assembly complex subunit YfgL
Shewanella sp. W3-18-1	6.50E-030	BamC	YP_963700.1	NlpB/DapX family lipoprotein
Shewanella sp. W3-18-1	3.70E-168	BamD	YP_964513.1	putative lipoprotein
Shewanella sp. W3-18-1	2.20E-038	BamE	YP_964244.1	SmpA/OmlA domain-containing protein
Shewanella sp. W3-18-1	1.30E-224	Omp85	YP_964118.1	surface antigen (D15)
Shewanella sp. W3-18-1	6.30E-069	Omp85	YP_963493.1	surface antigen (D15)
Shewanella violacea DSS12	4.50E-201	BamB	YP_003556127.1	PQQ enzyme repeat domain-containing protein
Shewanella violacea DSS12	4.70E-024	BamC	YP_003556667.1	lipoprotein-34 NlpB
Shewanella violacea DSS12	1.60E-147	BamD	YP_003558176.1	hypothetical protein SVI_3427
Shewanella violacea DSS12	2.90E-040	BamE	YP_003556106.1	small protein A
Shewanella violacea DSS12	5.60E-219	Omp85	YP_003555990.1	surface antigen
Shewanella violacea DSS12	4.20E-027	Omp85	YP_003557206.1	surface antigen
Shewanella woodyi ATCC 51908	8.10E-197	BamB	YP_001759930.1	outer membrane protein assembly complex subunit YfgL
Shewanella woodyi ATCC 51908	1.50E-008	BamB	YP_001760631.1	methanol/ethanol family PQQ-dependent dehydrogenase
Shewanella woodyi ATCC 51908	9.10E-019	BamC	YP_001760555.1	NlpB/DapX family lipoprotein
Shewanella woodyi ATCC 51908	9.70E-148	BamD	YP_001762078.1	putative lipoprotein
Shewanella woodyi ATCC 51908	7.80E-006	BamD	YP_001759002.1	TPR repeat-containing protein
Shewanella woodyi ATCC 51908	4.20E-040	BamE	YP_001759906.1	SmpA/OmlA domain-containing protein
Shewanella woodyi ATCC 51908	1.30E-215	Omp85	YP_001761638.1	outer membrane protein assembly complex, YaeT protein
Shewanella woodyi ATCC 51908	1.40E-041	Omp85	YP_001761058.1	surface antigen (D15)
Shigella boydii CDC 3083-94	1.00E-222	BamB	YP_001881303.1	outer membrane protein assembly complex subunit YfgL
Shigella boydii CDC 3083-94	1.20E-232	BamC	YP_001881269.1	lipoprotein
Shigella boydii CDC 3083-94	1.70E-174	BamD	YP_001881383.1	outer membrane protein assembly complex subunit YfiO

Shigella boydii CDC 3083-94	1.70E-067	BamE	YP_001881407.1	hypothetical protein SbBS512_E3006
Shigella boydii CDC 3083-94	2.30E-005	BamE	YP_001880531.1	DNA-binding transcriptional activator OsmE
Shigella boydii CDC 3083-94	0.00E+000	Omp85	YP_001878979.1	outer membrane protein assembly factor YaeT
Shigella boydii CDC 3083-94	9.30E-007	Omp85	YP_001882992.1	outer membrane protein, OMP85 family
Shigella boydii Sb227	5.10E-224	BamB	YP_408908.1	outer membrane protein assembly complex subunit YfgL
Shigella boydii Sb227	1.30E-005	BamB	YP_406674.1	glucose dehydrogenase
Shigella boydii Sb227	4.70E-233	BamC	YP_408872.1	lipoprotein
Shigella boydii Sb227	4.30E-173	BamD	YP_408995.1	outer membrane protein assembly complex subunit YfiO
Shigella boydii Sb227	1.30E-007	BamE	YP_409107.1	small membrane protein A
Shigella boydii Sb227	2.30E-005	BamE	YP_407805.1	DNA-binding transcriptional activator OsmE
Shigella boydii Sb227	0.00E+000	Omp85	YP_406723.1	outer membrane protein assembly factor YaeT
Shigella boydii Sb227	9.00E-007	Omp85	YP_410476.1	hypothetical protein SBO_4223
Shigella dysenteriae Sd197	6.60E-225	BamB	YP_404246.1	outer membrane protein assembly complex subunit YfgL
Shigella dysenteriae Sd197	9.10E-237	BamC	YP_404208.1	lipoprotein
Shigella dysenteriae Sd197	8.70E-175	BamD	YP_404368.1	outer membrane protein assembly complex subunit YfiO
Shigella dysenteriae Sd197	5.80E-068	BamE	YP_404324.2	hypothetical protein SDY_2790
Shigella dysenteriae Sd197	2.30E-005	BamE	YP_403168.1	DNA-binding transcriptional activator OsmE
Shigella dysenteriae Sd197	0.00E+000	Omp85	YP_401914.1	outer membrane protein assembly factor YaeT
Shigella dysenteriae Sd197	1.30E-006	Omp85	YP_405829.1	hypothetical protein SDY_4457
Shigella flexneri 2a str. 2457T	5.10E-224	BamB	NP_838075.1	outer membrane protein assembly complex subunit YfgL
Shigella flexneri 2a str. 2457T	7.80E-006	BamB	NP_835860.1	glucose dehydrogenase
Shigella flexneri 2a str. 2457T	8.10E-238	BamC	NP_838027.1	lipoprotein
Shigella flexneri 2a str. 2457T	4.30E-174	BamD	NP_838161.1	outer membrane protein assembly complex subunit YfiO
Shigella flexneri 2a str. 2457T	1.30E-007	BamE	NP_838186.1	small membrane protein A
Shigella flexneri 2a str. 2457T	2.30E-005	BamE	NP_837165.1	DNA-binding transcriptional activator OsmE
Shigella flexneri 2a str. 2457T	0.00E+000	Omp85	NP_835905.1	outer membrane protein assembly factor YaeT
Shigella flexneri 2a str. 2457T	1.00E-006	Omp85	NP_839659.1	hypothetical protein S4531
Shigella flexneri 5 str. 8401	6.60E-224	BamB	YP_689963.1	outer membrane protein assembly complex subunit YfgL
Shigella flexneri 5 str. 8401	7.80E-006	BamB	YP_687705.1	glucose dehydrogenase
Shigella flexneri 5 str. 8401	8.10E-238	BamC	YP_689930.1	lipoprotein
Shigella flexneri 5 str. 8401	1.60E-173	BamD	YP_690054.1	outer membrane protein assembly complex subunit YfiO
Shigella flexneri 5 str. 8401	5.80E-068	BamE	YP_690236.2	hypothetical protein SFV_2854
Shigella flexneri 5 str. 8401	2.30E-005	BamE	YP_688973.1	DNA-binding transcriptional activator OsmE
Shigella flexneri 5 str. 8401	0.00E+000	Omp85	YP_687749.1	outer membrane protein assembly factor YaeT
Shigella flexneri 5 str. 8401	1.30E-006	Omp85	YP_691554.1	hypothetical protein SFV_4268
Shigella sonnei Ss046	6.60E-225	BamB	YP_311457.1	outer membrane protein assembly complex subunit YfgL
Shigella sonnei Ss046	8.30E-006	BamB	YP_309161.1	glucose dehydrogenase
Shigella sonnei Ss046	8.10E-238	BamC	YP_311422.1	lipoprotein

Shigella sonnei Ss046	1.70E-174	BamD	YP_311573.1	outer membrane protein assembly complex subunit YfiO
Shigella sonnei Ss046	5.80E-068	BamE	YP_311625.2	hypothetical protein SSON_2773
Shigella sonnei Ss046	2.30E-005	BamE	YP_310361.1	DNA-binding transcriptional activator OsmE
Shigella sonnei Ss046	0.00E+000	Omp85	YP_309216.1	outer membrane protein assembly factor YaeT
Shigella sonnei Ss046	9.60E-007	Omp85	YP_313120.1	hypothetical protein SSON_4405
Sideroxydans lithotrophicus ES-1	5.80E-113	BamB	YP_003524041.1	outer membrane assembly lipoprotein YfgL
Sideroxydans lithotrophicus ES-1	1.20E-099	BamD	YP_003524654.1	outer membrane assembly lipoprotein YfiO
Sideroxydans lithotrophicus ES-1	1.40E-020	BamE	YP_003523675.1	SmpA/OmlA domain protein
Sideroxydans lithotrophicus ES-1	3.00E-212	Omp85	YP_003524263.1	outer membrane protein assembly complex, YaeT protein
Sodalis glossinidius str. 'morsitans'	5.70E-195	BamB	YP_455437.1	outer membrane protein assembly complex subunit YfgL
Sodalis glossinidius str. 'morsitans'	8.10E-242	BamC	YP_455405.1	lipoprotein
Sodalis glossinidius str. 'morsitans'	9.10E-151	BamD	YP_454262.1	outer membrane protein assembly complex subunit YfiO
Sodalis glossinidius str. 'morsitans'	2.20E-058	BamE	YP_455481.1	hypothetical protein SG1801
Sodalis glossinidius str. 'morsitans'	1.60E-005	BamE	YP_455545.1	DNA-binding transcriptional activator OsmE
Sodalis glossinidius str. 'morsitans'	1.90E-304	Omp85	YP_455615.1	outer membrane protein assembly factor YaeT
Sodalis glossinidius str. 'morsitans'	4.10E-007	Omp85	YP_454032.1	hypothetical protein SG0352
Stenotrophomonas maltophilia K279a	1.50E-119	BamB	YP_001971868.1	putative PQQ containing lipoprotein
Stenotrophomonas maltophilia K279a	1.50E-071	BamD	YP_001973447.1	putative competence lipoprotein
Stenotrophomonas maltophilia K279a	4.40E-021	BamE	YP_001971801.1	putative outer membrane lipoprotein
Stenotrophomonas maltophilia K279a	3.90E-008	BamE	YP_001972039.1	putative small protein A homolog
Stenotrophomonas maltophilia K279a	3.60E-005	BamE	YP_001972617.1	putative OmpA family protein
Stenotrophomonas maltophilia K279a	3.60E-193	Omp85	YP_001971339.1	putative outer membrane protein
Stenotrophomonas maltophilia K279a	7.00E-007	Omp85	YP_001974262.1	putative surface antigen exported protein
Stenotrophomonas maltophilia R551-3	6.20E-119	BamB	YP_002028049.1	outer membrane assembly lipoprotein YfgL
Stenotrophomonas maltophilia R551-3	8.50E-070	BamD	YP_002029542.1	outer membrane assembly lipoprotein YfiO
Stenotrophomonas maltophilia R551-3	8.20E-020	BamE	YP_002027981.1	SmpA/OmlA domain-containing protein
Stenotrophomonas maltophilia R551-3	6.00E-005	BamE	YP_002028700.1	OmpA/MotB domain-containing protein
Stenotrophomonas maltophilia R551-3	4.30E-185	Omp85	YP_002027645.1	outer membrane protein assembly complex, YaeT protein
Stenotrophomonas maltophilia R551-3	1.40E-006	Omp85	YP_002030363.1	surface antigen (D15)
Teredinibacter turnerae T7901	3.60E-109	BamB	YP_003074042.1	outer membrane assembly lipoprotein YfgL
Teredinibacter turnerae T7901	1.50E-007	BamC	YP_003072578.1	NlpB/DapX lipoprotein
Teredinibacter turnerae T7901	3.70E-110	BamD	YP_003072648.1	outer membrane assembly lipoprotein YfiO
Teredinibacter turnerae T7901	1.10E-027	BamE	YP_003074657.1	lipoprotein, SmpA/OmlA family
Teredinibacter turnerae T7901	4.40E-152	Omp85	YP_003072609.1	outer membrane protein assembly complex, YaeT protein
Thioalkalimicrobium cyclicum ALM1	2.60E-049	BamB	YP_004536494.1	outer membrane assembly lipoprotein YfgL
Thioalkalimicrobium cyclicum ALM1	3.90E-059	BamD	YP_004536942.1	outer membrane assembly lipoprotein YfiO
Thioalkalimicrobium cyclicum ALM1	4.10E-008	BamE	YP_004537308.1	SmpA/OmlA domain-containing protein
Thioalkalimicrobium cyclicum ALM1	1.80E-184	Omp85	YP_004537210.1	outer membrane protein assembly complex, YaeT protein

Thioalkalimicrobium cyclicum ALM1	5.50E-005	Omp85	YP_004537407.1	surface antigen (D15)
Thioalkalivibrio sp. K90mix	1.00E-096	BamB	YP_003459988.1	outer membrane assembly lipoprotein YfgL
Thioalkalivibrio sp. K90mix	3.70E-005	BamC	YP_003459660.1	lipoprotein
Thioalkalivibrio sp. K90mix	2.20E-103	BamD	YP_003461262.1	outer membrane assembly lipoprotein YfiO
Thioalkalivibrio sp. K90mix	5.30E-005	BamD	YP_003459819.1	tol-pal system protein YbgF
Thioalkalivibrio sp. K90mix	4.90E-018	BamE	YP_003461278.1	SmpA/OmlA domain-containing protein
Thioalkalivibrio sp. K90mix	2.00E-191	Omp85	YP_003460729.1	outer membrane protein assembly complex, YaeT protein
Thioalkalivibrio sp. K90mix	2.30E-007	Omp85	YP_003459547.1	surface antigen (D15)
Thioalkalivibrio sulfidophilus HL-EbGr7	5.40E-135	BamB	YP_002514113.1	hypothetical protein Tgr7_2046
Thioalkalivibrio sulfidophilus HL-EbGr7	1.20E-006	BamC	YP_002512906.1	NlpB/DapX family lipoprotein
Thioalkalivibrio sulfidophilus HL-EbGr7	1.90E-109	BamD	YP_002512892.1	competence lipoprotein
Thioalkalivibrio sulfidophilus HL-EbGr7	1.80E-011	BamD	YP_002512326.1	outer membrane assembly lipoprotein YfiO
Thioalkalivibrio sulfidophilus HL-EbGr7	2.10E-019	BamE	YP_002513041.1	outer membrane lipoprotein
Thioalkalivibrio sulfidophilus HL-EbGr7	2.50E-205	Omp85	YP_002513240.1	outer membrane protein assembly complex, YaeT protein
Thioalkalivibrio sulfidophilus HL-EbGr7	6.20E-007	Omp85	YP_002512533.1	outer membrane protein, OMP85 family
Thiomicrospira crunogena XCL-2	3.00E-056	BamB	YP_390894.1	Pyrrolo-quinoline quinone
Thiomicrospira crunogena XCL-2	5.10E-069	BamD	YP_391640.1	competence lipoprotein ComL
Thiomicrospira crunogena XCL-2	1.20E-010	BamE	YP_391675.1	SmpA/OmlA
Thiomicrospira crunogena XCL-2	3.60E-200	Omp85	YP_391547.1	surface antigen (D15)
Tolumonas auensis DSM 9187	9.80E-155	BamB	YP_002892105.1	outer membrane assembly lipoprotein YfgL
Tolumonas auensis DSM 9187	5.80E-020	BamC	YP_002892339.1	NlpBDapX family lipoprotein
Tolumonas auensis DSM 9187	2.70E-133	BamD	YP_002893951.1	outer membrane assembly lipoprotein YfiO
Tolumonas auensis DSM 9187	9.80E-036	BamE	YP_002893445.1	SmpA/OmlA domain-containing protein
Tolumonas auensis DSM 9187	1.40E-240	Omp85	YP_002893288.1	outer membrane protein assembly complex, YaeT protein
Tolumonas auensis DSM 9187	5.20E-006	Omp85	YP_002892315.1	surface antigen (D15)
Vibrio anguillarum 775	3.80E-148	BamB	YP_004566922.1	PQQ enzyme repeat family protein
Vibrio anguillarum 775	5.50E-178	BamC	YP_004565637.1	Lipoprotein-34
Vibrio anguillarum 775	2.80E-153	BamD	YP_004566969.1	Lipoprotein, ComL family
Vibrio anguillarum 775	4.60E-060	BamE	YP_004566864.1	SmpA
Vibrio anguillarum 775	3.10E-231	Omp85	YP_004565556.1	YaeT
Vibrio anguillarum 775	8.30E-006	Omp85	YP_004565273.1	outer membrane protein
Vibrio cholerae M66-2	7.50E-148	BamB	YP_002809492.1	outer membrane protein assembly complex subunit YfgL
Vibrio cholerae M66-2	2.60E-173	BamC	YP_002810832.1	lipoprotein
Vibrio cholerae M66-2	5.90E-153	BamD	YP_002809439.1	hypothetical protein VCM66_0666
Vibrio cholerae M66-2	3.70E-060	BamE	YP_002809578.1	small protein A
Vibrio cholerae M66-2	1.40E-234	Omp85	YP_002810927.1	outer membrane protein assembly factor YaeT
Vibrio cholerae M66-2	2.80E-006	Omp85	YP_002811217.1	hypothetical protein VCM66_2469
Vibrio cholerae MJ-1236	7.50E-148	BamB	YP_002879290.1	outer membrane protein assembly complex subunit YfgL

Vibrio cholerae MJ-1236	2.60E-173	BamC	YP_002877955.1	lipoprotein
Vibrio cholerae MJ-1236	5.90E-153	BamD	YP_002879340.1	hypothetical protein VCD_003614
Vibrio cholerae MJ-1236	3.70E-060	BamE	YP_002879207.1	lipoprotein SmpA a component of the essential YaeT outer-membrane protein assembly complex
Vibrio cholerae MJ-1236	1.40E-234	Omp85	YP_002877826.1	outer membrane protein assembly factor YaeT
Vibrio cholerae MJ-1236	3.50E-006	Omp85	YP_002877553.1	uncharacterized protein YtfM precursor
Vibrio cholerae O1 biovar El Tor str. N16961	7.50E-148	BamB	NP_230411.1	outer membrane protein assembly complex subunit YfgL
Vibrio cholerae O1 biovar El Tor str. N16961	2.60E-173	BamC	NP_231787.1	lipoprotein
Vibrio cholerae O1 biovar El Tor str. N16961	5.90E-153	BamD	NP_230357.1	hypothetical protein VC0708
Vibrio cholerae O1 biovar El Tor str. N16961	3.70E-060	BamE	NP_230498.1	small protein A
Vibrio cholerae O1 biovar El Tor str. N16961	1.40E-234	Omp85	NP_231883.1	outer membrane protein assembly factor YaeT
Vibrio cholerae O1 biovar El Tor str. N16961	2.80E-006	Omp85	NP_232176.1	hypothetical protein VC2548
Vibrio cholerae O395	7.50E-148	BamB	YP_001216247.1	outer membrane protein assembly complex subunit YfgL
Vibrio cholerae O395	1.40E-172	BamC	YP_001217676.1	lipoprotein
Vibrio cholerae O395	2.70E-153	BamD	YP_001216200.1	putative lipoprotein
Vibrio cholerae O395	3.70E-060	BamE	YP_001216332.1	small protein A
Vibrio cholerae O395	1.40E-234	Omp85	YP_001217767.1	outer membrane protein assembly factor YaeT
Vibrio cholerae O395	2.80E-006	Omp85	YP_001218042.1	hypothetical protein VC0395_A2126
Vibrio fischeri ES114	1.40E-155	BamB	YP_204015.1	outer membrane protein assembly complex subunit YfgL
Vibrio fischeri ES114	2.10E-164	BamC	YP_205300.1	lipoprotein
Vibrio fischeri ES114	6.80E-150	BamD	YP_203946.1	lipoprotein component of outer membrane protein assembly complex
Vibrio fischeri ES114	4.40E-058	BamE	YP_205382.1	small protein A
Vibrio fischeri ES114	2.70E-232	Omp85	YP_205337.1	outer membrane protein assembly factor YaeT
Vibrio fischeri ES114	1.20E-006	Omp85	YP_203715.1	outer membrane protein and surface antigen
Vibrio fischeri MJ11	3.10E-155	BamB	YP_002155389.1	outer membrane assembly lipoprotein YfgL
Vibrio fischeri MJ11	2.00E-164	BamC	YP_002156745.1	lipoprotein-34
Vibrio fischeri MJ11	6.80E-150	BamD	YP_002155321.1	competence lipoprotein ComL
Vibrio fischeri MJ11	1.90E-057	BamE	YP_002156830.1	small protein A
Vibrio fischeri MJ11	6.50E-005	BamE	YP_002157470.1	lipoprotein, putative
Vibrio fischeri MJ11	8.90E-232	Omp85	YP_002156782.1	outer membrane protein assembly complex, YaeT protein
Vibrio fischeri MJ11	2.50E-006	Omp85	YP_002155090.1	outer membrane protein
Vibrio harveyi ATCC BAA-1116	8.90E-144	BamB	YP_001444290.1	outer membrane protein assembly complex subunit YfgL
Vibrio harveyi ATCC BAA-1116	3.70E-176	BamC	YP_001446362.1	lipoprotein
Vibrio harveyi ATCC BAA-1116	1.70E-162	BamD	YP_001444230.1	hypothetical protein VIBHAR_01004
Vibrio harveyi ATCC BAA-1116	1.10E-074	BamE	YP_001444345.1	hypothetical protein VIBHAR_01128
Vibrio harveyi ATCC BAA-1116	6.40E-005	BamE	YP_001444676.1	hypothetical protein VIBHAR_01479
Vibrio harveyi ATCC BAA-1116	2.20E-238	Omp85	YP_001446405.1	outer membrane protein assembly factor YaeT
Vibrio harveyi ATCC BAA-1116	1.10E-006	Omp85	YP_001444007.1	hypothetical protein VIBHAR_00779

Vibrio parahaemolyticus RIMD 2210633	9.50E-144	BamB	NP_796990.1	outer membrane protein assembly complex subunit YfgL
Vibrio parahaemolyticus RIMD 2210633	6.90E-184	BamC	NP_798651.1	lipoprotein
Vibrio parahaemolyticus RIMD 2210633	3.00E-165	BamD	NP_796937.1	hypothetical protein VP0558
Vibrio parahaemolyticus RIMD 2210633	8.00E-069	BamE	NP_797026.1	small protein A
Vibrio parahaemolyticus RIMD 2210633	8.20E-237	Omp85	NP_798689.1	outer membrane protein assembly factor YaeT
Vibrio parahaemolyticus RIMD 2210633	3.40E-006	Omp85	NP_796686.1	hypothetical protein VP0307
Vibrio sp. Ex25	1.30E-141	BamB	YP_003286969.1	YfgL protein
Vibrio sp. Ex25	1.50E-180	BamC	YP_003285421.1	NlpB lipoprotein component of the protein assembly complex
Vibrio sp. Ex25	1.10E-163	BamD	YP_003287018.1	component of the lipoprotein assembly complex
Vibrio sp. Ex25	3.80E-069	BamE	YP_003286920.1	lipoprotein SmpA a component of the essential YaeT outer-membrane protein assembly complex
Vibrio sp. Ex25	1.80E-237	Omp85	YP_003285381.1	outer membrane protein assembly factor YaeT
Vibrio sp. Ex25	3.90E-006	Omp85	YP_003284322.1	hypothetical protein VEA_001694
Vibrio splendidus LGP32	7.90E-157	BamB	YP_002416265.1	outer membrane protein assembly complex subunit YfgL
Vibrio splendidus LGP32	6.30E-176	BamC	YP_002417892.1	lipoprotein
Vibrio splendidus LGP32	5.90E-152	BamD	YP_002416214.1	hypothetical lipoprotein
Vibrio splendidus LGP32	9.30E-065	BamE	YP_002416287.1	small protein A
Vibrio splendidus LGP32	8.80E-005	BamE	YP_002416773.1	hypothetical protein VS_1159
Vibrio splendidus LGP32	2.10E-229	Omp85	YP_002417931.1	outer membrane protein assembly factor YaeT
Vibrio splendidus LGP32	9.10E-007	Omp85	YP_002418315.1	outer membrane protein
Vibrio vulnificus CMCP6	9.50E-146	BamB	NP_759420.1	outer membrane biogenesis protein BamB
Vibrio vulnificus CMCP6	2.70E-223	BamC	NP_760787.1	lipoprotein
Vibrio vulnificus CMCP6	4.20E-156	BamD	NP_759477.1	putative component of the lipoprotein assembly complex
Vibrio vulnificus CMCP6	1.30E-063	BamE	NP_759365.1	outer membrane lipoprotein SmpA
Vibrio vulnificus CMCP6	2.90E-239	Omp85	NP_760743.1	outer membrane protein assembly factor YaeT
Vibrio vulnificus CMCP6	1.90E-006	Omp85	NP_759695.1	hypothetical protein VV1_0712
Vibrio vulnificus MO6-24/O	9.50E-146	BamB	YP_004189650.1	outer membrane protein YfgL
Vibrio vulnificus MO6-24/O	1.40E-221	BamC	YP_004188022.1	outer membrane protein NlpB
Vibrio vulnificus MO6-24/O	2.60E-154	BamD	YP_004189705.1	component of the lipoprotein assembly complex
Vibrio vulnificus MO6-24/O	1.30E-063	BamE	YP_004189620.1	outer membrane lipoprotein SmpA
Vibrio vulnificus MO6-24/O	2.90E-239	Omp85	YP_004187979.1	outer membrane protein assembly factor YaeT
Vibrio vulnificus MO6-24/O	1.90E-006	Omp85	YP_004189932.1	hypothetical protein VVM_04289
Vibrio vulnificus YJ016	9.50E-146	BamB	NP_933562.1	outer membrane protein assembly complex subunit YfgL
Vibrio vulnificus YJ016	4.90E-222	BamC	NP_935296.1	lipoprotein
Vibrio vulnificus YJ016	4.20E-156	BamD	NP_933505.1	lipoprotein
Vibrio vulnificus YJ016	3.80E-062	BamE	NP_933614.1	small protein A
Vibrio vulnificus YJ016	2.90E-239	Omp85	NP_935342.1	outer membrane protein assembly factor YaeT
Vibrio vulnificus YJ016	1.90E-006	Omp85	NP_933221.1	outer membrane protein

Wigglesworthia glossinidia endosymbiont of Glossina brevipalpis	3.20E-013	BamD	NP_871537.1	hypothetical protein WGLp534
Wigglesworthia glossinidia endosymbiont of Glossina brevipalpis	4.80E-005	BamD	NP_871413.1	hypothetical protein WGLp410
Wigglesworthia glossinidia endosymbiont of Glossina brevipalpis	1.20E-053	Omp85	NP_871387.1	hypothetical protein WGLp384
Xanthomonas axonopodis pv. citri str. 306	1.10E-115	BamB	NP_642346.1	hypothetical protein XAC2020
Xanthomonas axonopodis pv. citri str. 306	1.50E-005	BamB	NP_641965.1	glucose dehydrogenase
Xanthomonas axonopodis pv. citri str. 306	6.50E-005	BamB	NP_641001.1	methanol dehydrogenase heavy chain
Xanthomonas axonopodis pv. citri str. 306	7.20E-005	BamB	NP_644498.1	polyvinylalcohol dehydrogenase
Xanthomonas axonopodis pv. citri str. 306	4.90E-074	BamD	NP_643526.1	competence lipoprotein
Xanthomonas axonopodis pv. citri str. 306	3.70E-019	BamE	NP_641849.1	outer membrane protein
Xanthomonas axonopodis pv. citri str. 306	4.10E-185	Omp85	NP_641748.1	outer membrane antigen
Xanthomonas axonopodis pv. citri str. 306	2.40E-006	Omp85	NP_644503.1	hypothetical protein XAC4204
Xanthomonas campestris pv. campestris str. 8004	3.90E-113	BamB	YP_243274.1	hypothetical protein XC_2198
Xanthomonas campestris pv. campestris str. 8004	2.20E-005	BamB	YP_243728.1	glucose dehydrogenase
Xanthomonas campestris pv. campestris str. 8004	2.60E-005	BamB	YP_245229.1	polyvinylalcohol dehydrogenase
Xanthomonas campestris pv. campestris str. 8004	3.50E-005	BamB	YP_241778.1	methanol dehydrogenase heavy chain
Xanthomonas campestris pv. campestris str. 8004	1.10E-073	BamD	YP_242159.1	competence lipoprotein
Xanthomonas campestris pv. campestris str. 8004	2.30E-018	BamE	YP_243837.1	outer membrane protein
Xanthomonas campestris pv. campestris str. 8004	4.10E-197	Omp85	YP_243941.1	outer membrane antigen
Xanthomonas campestris pv. campestris str. 8004	4.30E-007	Omp85	YP_245234.1	hypothetical protein XC_4176
Xanthomonas campestris pv. campestris str. ATCC 33913	3.90E-113	BamB	NP_637351.1	hypothetical protein XCC1986
Xanthomonas campestris pv. campestris str. ATCC 33913	1.60E-005	BamB	NP_636946.1	glucose dehydrogenase
Xanthomonas campestris pv. campestris str. ATCC 33913	3.40E-005	BamB	NP_639419.1	polyvinylalcohol dehydrogenase
Xanthomonas campestris pv. campestris str. ATCC 33913	3.50E-005	BamB	NP_638828.1	methanol dehydrogenase heavy chain
Xanthomonas campestris pv. campestris str. ATCC 33913	1.10E-073	BamD	NP_638436.1	competence lipoprotein
Xanthomonas campestris pv. campestris str. ATCC 33913	2.30E-018	BamE	NP_636841.1	outer membrane protein
Xanthomonas campestris pv. campestris str. ATCC 33913	4.10E-197	Omp85	NP_636739.1	outer membrane antigen
Xanthomonas campestris pv. campestris str. ATCC 33913	4.30E-007	Omp85	NP_639424.1	hypothetical protein XCC4085
Xanthomonas campestris pv. campestris str. B100	3.90E-113	BamB	YP_001903690.1	Putative quinoprotein
Xanthomonas campestris pv. campestris str. B100	1.50E-005	BamB	YP_001905687.1	exported putative alcohol dehydrogenase (acceptor)
Xanthomonas campestris pv. campestris str. B100	2.20E-005	BamB	YP_001904090.1	quinoprotein glucose dehydrogenase
Xanthomonas campestris pv. campestris str. B100	3.50E-005	BamB	YP_001902121.1	exported alcohol dehydrogenase (acceptor)
Xanthomonas campestris pv. campestris str. B100	1.10E-073	BamD	YP_001902508.1	putative outer membrane lipoprotein
Xanthomonas campestris pv. campestris str. B100	2.30E-018	BamE	YP_001904209.1	Putative outer membrane protein
Xanthomonas campestris pv. campestris str. B100	4.00E-198	Omp85	YP_001904336.1	outer membrane protein
Xanthomonas campestris pv. campestris str. B100	1.10E-007	Omp85	YP_001905693.1	Putative outer membrane protein
Xanthomonas campestris pv. vesicatoria str. 85-10	1.10E-114	BamB	YP_363802.1	putative quinoprotein
Xanthomonas campestris pv. vesicatoria str. 85-10	1.90E-005	BamB	YP_366031.1	putative polyvinylalcohol dehydrogenase
Xanthomonas campestris pv. vesicatoria str. 85-10	2.00E-005	BamB	YP_363404.1	glucose dehydrogenase

Xanthomonas campestris pv. vesicatoria str. 85-10	3.10E-005	BamB	YP_362439.1	methanol dehydrogenase heavy chain (fragment)
Xanthomonas campestris pv. vesicatoria str. 85-10	4.90E-074	BamD	YP_365074.1	putative competence lipoprotein
Xanthomonas campestris pv. vesicatoria str. 85-10	8.40E-018	BamE	YP_363290.1	putative outer membrane protein
Xanthomonas campestris pv. vesicatoria str. 85-10	6.40E-185	Omp85	YP_363201.1	outer membrane antigen
Xanthomonas campestris pv. vesicatoria str. 85-10	2.90E-006	Omp85	YP_366037.1	putative outer membrane protein
Xanthomonas oryzae pv. oryzae KACC10331	2.00E-113	BamB	YP_201169.1	hypothetical protein XOO2530
Xanthomonas oryzae pv. oryzae KACC10331	8.50E-005	BamB	YP_198968.1	polyvinylalcohol dehydrogenase
Xanthomonas oryzae pv. oryzae KACC10331	1.10E-074	BamD	YP_200235.1	competence lipoprotein
Xanthomonas oryzae pv. oryzae KACC10331	3.90E-020	BamE	YP_200665.1	outer membrane protein
Xanthomonas oryzae pv. oryzae KACC10331	5.90E-197	Omp85	YP_200607.1	outer membrane antigen
Xanthomonas oryzae pv. oryzae KACC10331	2.60E-006	Omp85	YP_202944.1	hypothetical protein XOO4305
Xanthomonas oryzae pv. oryzae MAFF 311018	2.00E-113	BamB	YP_451417.1	hypothetical protein XOO_2388
Xanthomonas oryzae pv. oryzae MAFF 311018	6.70E-006	BamB	YP_449330.1	polyvinylalcohol dehydrogenase
Xanthomonas oryzae pv. oryzae MAFF 311018	1.10E-074	BamD	YP_450509.1	competence lipoprotein
Xanthomonas oryzae pv. oryzae MAFF 311018	3.90E-020	BamE	YP_450937.1	outer membrane protein
Xanthomonas oryzae pv. oryzae MAFF 311018	5.90E-197	Omp85	YP_450887.1	outer membrane antigen
Xanthomonas oryzae pv. oryzae MAFF 311018	2.60E-006	Omp85	YP_453089.1	putative outer membrane protein
Xanthomonas oryzae pv. oryzae PXO99A	2.00E-113	BamB	YP_001913832.1	serine/threonine protein kinase
Xanthomonas oryzae pv. oryzae PXO99A	2.00E-113	BamB	YP_001913641.1	serine/threonine protein kinase
Xanthomonas oryzae pv. oryzae PXO99A	1.10E-005	BamB	YP_001916000.1	polyvinylalcohol dehydrogenase
Xanthomonas oryzae pv. oryzae PXO99A	2.80E-071	BamD	YP_001912669.1	competence lipoprotein
Xanthomonas oryzae pv. oryzae PXO99A	3.90E-020	BamE	YP_001914042.1	outer membrane protein
Xanthomonas oryzae pv. oryzae PXO99A	1.50E-186	Omp85	YP_001914100.1	outer membrane antigen
Xanthomonas oryzae pv. oryzae PXO99A	4.60E-006	Omp85	YP_001911674.1	pathogenicity protein
Xenorhabdus bovienii SS-2004	4.60E-168	BamB	YP_003468892.1	serine/threonine protein kinase with quinoprotein alcohol dehydrogenase domain
Xenorhabdus bovienii SS-2004	2.00E-005	BamB	YP_003468085.1	hypothetical protein XBJ1_2186
Xenorhabdus bovienii SS-2004	5.00E-174	BamC	YP_003468521.1	lipoprotein-34
Xenorhabdus bovienii SS-2004	1.60E-136	BamD	YP_003469142.1	lipoprotein with tetratricopeptide repeats (TPR) domain
Xenorhabdus bovienii SS-2004	5.90E-057	BamE	YP_003469048.1	small membrane protein A
Xenorhabdus bovienii SS-2004	2.10E-303	Omp85	YP_003466537.1	outer membrane antigen
Xenorhabdus bovienii SS-2004	2.60E-006	Omp85	YP_003466287.1	outer membrane protein
Xenorhabdus nematophila ATCC 19061	1.10E-166	BamB	YP_003713490.1	serine/threonine protein kinase with quinoprotein alcohol dehydrogenase domain
Xenorhabdus nematophila ATCC 19061	3.70E-005	BamB	YP_003710918.1	hypothetical protein XNC1_0612
Xenorhabdus nematophila ATCC 19061	8.90E-194	BamC	YP_003712862.1	lipoprotein-34
Xenorhabdus nematophila ATCC 19061	1.90E-142	BamD	YP_003711555.1	lipoprotein
Xenorhabdus nematophila ATCC 19061	5.30E-059	BamE	YP_003713417.1	small membrane protein A
Xenorhabdus nematophila ATCC 19061	9.10E-005	BamE	YP_003711376.1	hypothetical protein XNC1_1096

Xenorhabdus nematophila ATCC 19061	5.90E-302	Omp85	YP_003714111.1	outer membrane antigen
Xenorhabdus nematophila ATCC 19061	2.60E-006	Omp85	YP_003710786.1	outer membrane protein
Xylella fastidiosa 9a5c	1.60E-094	BamB	NP_297754.1	hypothetical protein XF0464
Xylella fastidiosa 9a5c	5.10E-007	BamB	NP_299538.1	polyvinylalcohol dehydrogenase
Xylella fastidiosa 9a5c	4.90E-068	BamD	NP_298228.1	hypothetical protein XF0938
Xylella fastidiosa 9a5c	2.60E-005	BamD	NP_299448.1	hypothetical protein XF2169
Xylella fastidiosa 9a5c	8.10E-029	BamE	NP_299624.1	outer membrane protein
Xylella fastidiosa 9a5c	1.20E-186	Omp85	NP_298336.1	outer membrane antigen
Xylella fastidiosa 9a5c	4.10E-006	Omp85	NP_298521.1	hypothetical protein XF1231
Xylella fastidiosa M12	2.90E-097	BamB	YP_001776299.1	serine/threonine protein kinase
Xylella fastidiosa M12	1.20E-069	BamD	YP_001776425.1	putative lipoprotein
Xylella fastidiosa M12	7.30E-028	BamE	YP_001776062.1	outer membrane protein
Xylella fastidiosa M12	3.50E-187	Omp85	YP_001775004.1	outer membrane antigen
Xylella fastidiosa M12	4.90E-006	Omp85	YP_001775203.1	pathogenicity protein
Xylella fastidiosa M23	2.20E-096	BamB	YP_001830390.1	outer membrane assembly lipoprotein YfgL
Xylella fastidiosa M23	5.40E-008	BamB	YP_001830076.1	Pyrrolo-quinoline quinone
Xylella fastidiosa M23	5.20E-070	BamD	YP_001830531.1	competence lipoprotein
Xylella fastidiosa M23	7.30E-028	BamE	YP_001830145.1	SmpA/OmlA domain-containing protein
Xylella fastidiosa M23	1.30E-186	Omp85	YP_001829044.1	outer membrane protein assembly complex, YaeT protein
Xylella fastidiosa M23	1.80E-006	Omp85	YP_001829225.1	surface antigen (D15)
Xylella fastidiosa Temecula1	2.20E-096	BamB	NP_779811.1	serine/threonine protein kinase
Xylella fastidiosa Temecula1	6.60E-007	BamB	NP_779499.1	hypothetical protein PD1299
Xylella fastidiosa Temecula1	5.20E-070	BamD	NP_779941.1	hypothetical protein PD1756
Xylella fastidiosa Temecula1	7.30E-028	BamE	NP_779573.1	outer membrane protein
Xylella fastidiosa Temecula1	1.30E-186	Omp85	NP_778561.1	outer membrane antigen
Xylella fastidiosa Temecula1	3.20E-006	Omp85	NP_778733.1	pathogenicity protein
Yersinia enterocolitica subsp. enterocolitica 8081	9.40E-196	BamB	YP_001005408.1	outer membrane protein assembly complex subunit YfgL
Yersinia enterocolitica subsp. enterocolitica 8081	3.80E-231	BamC	YP_001005466.1	lipoprotein
Yersinia enterocolitica subsp. enterocolitica 8081	1.10E-160	BamD	YP_001005231.1	outer membrane protein assembly complex subunit YfiO
Yersinia enterocolitica subsp. enterocolitica 8081	8.10E-064	BamE	YP_001005330.2	hypothetical protein YE0997
Yersinia enterocolitica subsp. enterocolitica 8081	0.00E+000	Omp85	YP_001007450.1	outer membrane protein assembly factor YaeT
Yersinia enterocolitica subsp. enterocolitica 8081	3.10E-006	Omp85	YP_001008367.1	hypothetical protein YE0405
Yersinia enterocolitica subsp. palearctica 105.5R(r)	3.50E-195	BamB	YP_004299210.1	outer membrane protein assembly complex subunit YfgL
Yersinia enterocolitica subsp. palearctica 105.5R(r)	8.90E-232	BamC	YP_004299153.1	lipoprotein
Yersinia enterocolitica subsp. palearctica 105.5R(r)	1.10E-160	BamD	YP_004296857.1	outer membrane protein assembly complex subunit YfiO
Yersinia enterocolitica subsp. palearctica 105.5R(r)	2.10E-005	BamD	YP_004299537.1	putative tight adherance operon protein
Yersinia enterocolitica subsp. palearctica 105.5R(r)	4.10E-005	BamD	YP_004299215.1	putative fimbrial biogenesis protein
Yersinia enterocolitica subsp. palearctica 105.5R(r)	8.10E-064	BamE	YP_004299287.1	hypothetical protein YE105_C3090

Yersinia enterocolitica subsp. palearctica 105.5R(r)	0.00E+000	Omp85	YP_004297177.1	outer membrane protein assembly factor YaeT
Yersinia enterocolitica subsp. palearctica 105.5R(r)	1.50E-006	Omp85	YP_004296627.1	hypothetical protein YE105_C0426
Yersinia pestis Angola	8.50E-197	BamB	YP_001605021.1	outer membrane protein assembly complex subunit YfgL
Yersinia pestis Angola	1.60E-250	BamC	YP_001607495.1	lipoprotein
Yersinia pestis Angola	6.10E-160	BamD	YP_001607808.1	outer membrane protein assembly complex subunit YfiO
Yersinia pestis Angola	1.20E-061	BamE	YP_001605901.1	hypothetical protein YpAngola_A1375
Yersinia pestis Angola	0.00E+000	Omp85	YP_001607764.1	outer membrane protein assembly factor YaeT
Yersinia pestis Angola	3.00E-006	Omp85	YP_001608258.1	OMP85 family outer membrane protein
Yersinia pestis Antiqua	8.50E-197	BamB	YP_652226.1	outer membrane protein assembly complex subunit YfgL
Yersinia pestis Antiqua	1.60E-250	BamC	YP_652162.1	lipoprotein
Yersinia pestis Antiqua	6.10E-160	BamD	YP_652676.1	outer membrane protein assembly complex subunit YfiO
Yersinia pestis Antiqua	1.20E-061	BamE	YP_650495.1	hypothetical protein YPA_0582
Yersinia pestis Antiqua	0.00E+000	Omp85	YP_650441.1	outer membrane protein assembly factor YaeT
Yersinia pestis Antiqua	3.40E-006	Omp85	YP_649988.1	hypothetical protein YPA_0074
Yersinia pestis biovar Microtus str. 91001	8.50E-197	BamB	NP_994053.1	outer membrane protein assembly complex subunit YfgL
Yersinia pestis biovar Microtus str. 91001	1.60E-250	BamC	NP_993996.1	lipoprotein
Yersinia pestis biovar Microtus str. 91001	6.10E-160	BamD	NP_992043.1	outer membrane protein assembly complex subunit YfiO
Yersinia pestis biovar Microtus str. 91001	1.20E-061	BamE	NP_992425.2	hypothetical protein YP_1052
Yersinia pestis biovar Microtus str. 91001	0.00E+000	Omp85	NP_994105.1	outer membrane protein assembly factor YaeT
Yersinia pestis biovar Microtus str. 91001	3.40E-006	Omp85	NP_991952.1	hypothetical protein YP_0559
Yersinia pestis CO92	8.50E-197	BamB	YP_002347811.1	outer membrane protein assembly complex subunit YfgL
Yersinia pestis CO92	1.60E-250	BamC	YP_002347981.1	lipoprotein
Yersinia pestis CO92	6.10E-160	BamD	YP_002348178.1	outer membrane protein assembly complex subunit YfiO
Yersinia pestis CO92	1.20E-061	BamE	YP_002346147.1	hypothetical protein YPO1104
Yersinia pestis CO92	0.00E+000	Omp85	YP_002346095.1	outer membrane protein assembly factor YaeT
Yersinia pestis CO92	3.40E-006	Omp85	YP_002348413.1	hypothetical protein YPO3524
Yersinia pestis KIM 10	8.50E-197	BamB	NP_668678.1	outer membrane protein assembly complex subunit YfgL
Yersinia pestis KIM 10	1.60E-250	BamC	NP_668740.1	lipoprotein
Yersinia pestis KIM 10	6.10E-160	BamD	NP_668242.1	outer membrane protein assembly complex subunit YfiO
Yersinia pestis KIM 10	1.20E-061	BamE	NP_670375.2	hypothetical protein y3076
Yersinia pestis KIM 10	0.00E+000	Omp85	NP_670426.1	outer membrane protein assembly factor YaeT
Yersinia pestis KIM 10	3.40E-006	Omp85	NP_667996.1	hypothetical protein y0659
Yersinia pestis Nepal516	8.50E-197	BamB	YP_647192.1	outer membrane protein assembly complex subunit YfgL
Yersinia pestis Nepal516	1.60E-250	BamC	YP_647254.1	lipoprotein
Yersinia pestis Nepal516	6.10E-160	BamD	YP_646750.1	outer membrane protein assembly complex subunit YfiO
Yersinia pestis Nepal516	1.20E-061	BamE	YP_648821.1	hypothetical protein YPN_2894
Yersinia pestis Nepal516	0.00E+000	Omp85	YP_648875.1	outer membrane protein assembly factor YaeT
Yersinia pestis Nepal516	3.40E-006	Omp85	YP_649195.1	hypothetical protein YPN_3268

Yersinia pestis Pestoides F	8.50E-197	BamB	YP_001163569.1	outer membrane protein assembly complex subunit YfgL
Yersinia pestis Pestoides F	1.60E-250	BamC	YP_001163516.1	lipoprotein
Yersinia pestis Pestoides F	6.10E-160	BamD	YP_001164249.1	outer membrane protein assembly complex subunit YfiO
Yersinia pestis Pestoides F	1.20E-061	BamE	YP_001163934.1	hypothetical protein YPDSF_2593
Yersinia pestis Pestoides F	0.00E+000	Omp85	YP_001163018.1	outer membrane protein assembly factor YaeT
Yersinia pestis Pestoides F	3.40E-006	Omp85	YP_001164896.1	hypothetical protein YPDSF_3573
Yersinia pestis Z176003	8.50E-197	BamB	YP_003568624.1	putative lipoprotein
Yersinia pestis Z176003	1.60E-250	BamC	YP_003568869.1	lipoprotein
Yersinia pestis Z176003	6.10E-160	BamD	YP_003569054.1	putative lipoprotein
Yersinia pestis Z176003	1.20E-061	BamE	YP_003567174.1	hypothetical protein YPZ3_1002
Yersinia pestis Z176003	2.50E-005	BamE	YP_003566489.1	hypothetical protein YPZ3_0317
Yersinia pestis Z176003	0.00E+000	Omp85	YP_003567131.1	putative surface antigen
Yersinia pestis Z176003	3.40E-006	Omp85	YP_003569410.1	hypothetical protein YPZ3_3239
Yersinia pseudotuberculosis IP 31758	1.30E-196	BamB	YP_001400170.1	outer membrane protein assembly complex subunit YfgL
Yersinia pseudotuberculosis IP 31758	5.20E-250	BamC	YP_001400228.1	lipoprotein
Yersinia pseudotuberculosis IP 31758	6.10E-160	BamD	YP_001402172.1	outer membrane protein assembly complex subunit YfiO
Yersinia pseudotuberculosis IP 31758	1.50E-061	BamE	YP_001401853.1	hypothetical protein YpsIP31758_2890
Yersinia pseudotuberculosis IP 31758	0.00E+000	Omp85	YP_001400005.1	outer membrane protein assembly factor YaeT
Yersinia pseudotuberculosis IP 31758	3.40E-006	Omp85	YP_001402579.1	OMP85 family outer membrane protein
Yersinia pseudotuberculosis IP 32953	1.30E-196	BamB	YP_071345.1	outer membrane protein assembly complex subunit YfgL
Yersinia pseudotuberculosis IP 32953	5.20E-250	BamC	YP_071289.1	lipoprotein
Yersinia pseudotuberculosis IP 32953	6.10E-160	BamD	YP_069386.1	outer membrane protein assembly complex subunit YfiO
Yersinia pseudotuberculosis IP 32953	1.20E-061	BamE	YP_069673.2	hypothetical protein YPTB1138
Yersinia pseudotuberculosis IP 32953	0.00E+000	Omp85	YP_071501.1	outer membrane protein assembly factor YaeT
Yersinia pseudotuberculosis IP 32953	3.40E-006	Omp85	YP_068995.1	hypothetical protein YPTB0452
Yersinia pseudotuberculosis PB1/+	1.30E-196	BamB	YP_001873361.1	outer membrane protein assembly complex subunit YfgL
Yersinia pseudotuberculosis PB1/+	5.20E-250	BamC	YP_001873302.1	lipoprotein
Yersinia pseudotuberculosis PB1/+	6.10E-160	BamD	YP_001871325.1	outer membrane protein assembly complex subunit YfiO
Yersinia pseudotuberculosis PB1/+	1.20E-061	BamE	YP_001871647.1	hypothetical protein YPTS_1215
Yersinia pseudotuberculosis PB1/+	0.00E+000	Omp85	YP_001873527.1	outer membrane protein assembly factor YaeT
Yersinia pseudotuberculosis PB1/+	3.40E-006	Omp85	YP_001870925.1	surface antigen (D15)
